# Supplementary material for: Unravelling the reservoirs for colonisation of infants with Campylobacter spp. in rural Ethiopia: protocol for a longitudinal study during a global pandemic and political tensions
Source: BMJ Open. 2022 Oct 5;12(10):e061311. doi: 10.1136/bmjopen-2022-061311 (PMC9535169; doi:10.1136/bmjopen-2022-061311)
Supplement: Supplementary data [file bmjopen-2022-061311supp001.pdf]

Codebook

Codes for Missing Data	
Code / Value	Label
nd	nd

Data Dictionary Codebook

05/14/2022 3:47pm

Collapse all instruments

#	Variable / Field Name	Field Label<br>*Field Note*	Field Attributes (Field Type, Validation, Choices, Calculations, etc.)
Instrument: **Enrollment Questionnaire** (enrollment_questionnaire) Collapse			
1	caged_id	Record ID	text
2	signed_informed_consent	Signed Informed Consent	file<br>Field Annotation: @HIDDEN-APP
3	en_datac_date	Date of data collection (Gregorian calendar)	text (date_dmy)<br>Custom alignment: LV<br>Field Annotation: @HIDEBUTTON
4	en_datac_name	Enter data collector's name	dropdown<br>1 Ibsa<br>2 Abdi<br>3 Jafer<br>Custom alignment: LV
5	en_hh_id	Household ID	text<br>Custom alignment: LV
6	mother_id	Mother ID	text<br>Custom alignment: LV
7	en_hh_contact_name	Household contact name	text<br>Custom alignment: LV
8	en_hh_role	Household contact role (in relation to targeted child)	dropdown<br>1 Mother<br>2 Father<br>3 Grandfather<br>4 Grandmother<br>88 Other<br>Custom alignment: LV
9	en_hh_phone	Household contact phone number	text, Identifier<br>Custom alignment: LV
10	en_child_situation	What is the situation of the mother and child post childbirth? (may be discerned upon approaching the house or in discussion with household member, neighbor, or husband; does not require surveying the mother, but should be completed)	dropdown, Identifier<br>1 Successful delivery (continue)<br>2 Child or mother currently hospitalized (do not continue)<br>3 Child death (do not continue)<br>4 Maternal death (do not continue)<br>Custom alignment: LV
11	end_survey<br>Show the field ONLY if: [en_child_situation] = '2' or [en_child_situation] = '3' or [en_child_situation] = '4'	Do Not Continue - End Survey	descriptive

12	kebele	What is the Kebele?	dropdown<br>1 Negeya<br>2 Damota<br>3 Gobe Challa<br>4 Finqille<br>5 Biftu Geda<br>6 Ifa Oromia<br>7 Amuma<br>8 Kuro<br>9 Fendisha Lencho<br>10 Adele Walta<br>11 Qerensa Dereba<br>12 Bachaqe<br><br>Custom alignment: LV
13	ganda	What is the village/ganda?	text, Identifier<br>Custom alignment: LV
14	en_latitude	Latitude	text, Identifier<br>Custom alignment: LV
15	en_longitude	Longitude	text, Identifier<br>Custom alignment: LV
16	en_mother_quest	Section Header: *Questions for MOTHER*<br>We would like to ask you a number of question, prior to finalizing your enrollment.	descriptive
17	en_mother_fname	What is your (mother's) first name?	text, Identifier<br>Custom alignment: LV
18	en_mother_mname	What is your (mother's) middle name	text, Identifier<br>Custom alignment: LV
19	en_mother_lname	What is your (mother's) last name?	text, Identifier<br>Custom alignment: LV
20	en_mother_dob	What is your date of birth (Gregorian calendar)?<br>*Mother's Date of Birth*	text (date_dmy), Identifier<br>Custom alignment: LV<br>Field Annotation: @HIDEBUTTON
21	en_child_dob	What was the child's date of birth (Gregorian calendar)?	text (date_dmy)<br>Custom alignment: LV<br>Field Annotation: @HIDEBUTTON
22	en_mother_years	Exact age of Mother (years)	calc<br>Calculation: round(datediff([en_mother_dob], [en_child_dob],"y","dmy"),1)
23	en_mother_years_confirm	Please confirm calculated age as accurate.	yesno<br>1 Yes<br>0 No
24	en_mother_eligibility<br>Show the field ONLY if: [en_mother_years] < 16 and [en_mother_years_confirm] = '1'	Mother's calculated current age is less than 16 years of age, child cannot be included and survey should end. NOT ELIGIBLE	descriptive
25	en_father_fname	What is the child's father's first name?	text, Identifier<br>Custom alignment: LV
26	en_father_mname	What is the child's father's middle name?	text, Identifier<br>Custom alignment: LV
27	en_father_lname	What is the child's father's last name?	text, Identifier<br>Custom alignment: LV
28	en_child_sex	What is the child's sex?	dropdown<br>1 Male<br>2 Female<br><br>Custom alignment: LV

29	en_child_birthweight_known	Do you know the child's birthweight?	yesno<br>1 Yes<br>0 No<br><br>Custom alignment: LV
30	en_child_weight_2500<br><br>Show the field ONLY if:<br>[en_child_birthweight_known] = '0'	Does the child visibly appear to weight over 2500 grams?	yesno<br>1 Yes<br>0 No
31	en_child_wt<br><br>Show the field ONLY if:<br>[en_child_birthweight_known] = '1'	What was child's weight at birth? (grams)	text (number)<br>Custom alignment: LV
32	en_child_wt_verify<br><br>Show the field ONLY if:<br>[en_child_birthweight_known] = '1'	Please verify the child's weight in grams, is this correct?	yesno<br>1 Yes<br>0 No<br><br>Custom alignment: LV
33	en_singleton	Was this child delivered as a singleton birth?	yesno<br>1 Yes<br>0 No<br><br>Custom alignment: LV
34	stop_survey_ineligible<br><br>Show the field ONLY if:<br>[en_child_wt] < 2500 and [en_child_wt_verify] = '1' or [en_singleton] = '0' or [en_child_weight_2500] = '0'	STOP SURVEY - Child NOT ELIGIBLE	descriptive
35	en_residence	Did you reside in Haramaya during the last three months of your pregnancy with this child?	dropdown<br>1 Yes<br>0 No (cannot continue in study)<br><br>Custom alignment: LV
36	en_hosp_stay<br><br>Show the field ONLY if:<br>[en_residence] = '1'	Did you (mother) or child stay more than 4 days in hospital after birth?	dropdown<br>1 Yes (cannot continue in study)<br>0 No<br>99 Don't know<br><br>Custom alignment: LV
37	en_child_anom<br><br>Show the field ONLY if:<br>[en_hosp_stay] = '0' or [en_hosp_stay] = '99'	Does the child have visible congenital anomalies?	dropdown<br>1 Yes (cannot continue in study)<br>0 No<br>99 Don't know<br><br>Custom alignment: LV
38	en_enteropathy<br><br>Show the field ONLY if:<br>[en_child_anom] = '0' or [en_child_anom] = '99'	Has child been diagnosed with enteropathy by a medical doctor?	yesno<br>1 Yes<br>0 No<br><br>Custom alignment: LV
39	child_ineligible<br><br>Show the field ONLY if:<br>[en_residence] = '0' or [en_hosp_stay] = '1' or [en_child_anom] = '1' or [en_enteropathy] = '1'	STOP SURVEY - NOT ELIGIBLE	text<br>Custom alignment: LV

40	en_child_illness<br>Show the field ONLY if: [en_residence] = '1' or [en_hosp_stay] = '0' or [en_child_anom] = '0' or [en_hosp_stay] = '99' or [en_child_anom] = '99'	Does the child have any serious known medical illnesses?	yesno<br>1 Yes<br>0 No<br>Custom alignment: LV
41	en_child_illness_y<br>Show the field ONLY if: [en_child_illness] = '1'	If yes, comment	text<br>Custom alignment: LV
42	en_child_caregiver<br>Show the field ONLY if: [en_residence] = '0' or [en_hosp_stay] = '0' or [en_child_anom] = '0' or [en_hosp_stay] = '99' or [en_child_anom] = '99'	Who will be the primary caregiver for the child in his/her first six months of life? (If not mother, child cannot be included)	dropdown<br>1 Mother<br>2 Father<br>3 Grandfather<br>88 Other<br>Custom alignment: LV
43	child_ineligible_2<br>Show the field ONLY if: [en_child_caregiver] = '2' or [en_child_caregiver] = '3' or [en_child_caregiver] = '88' or [en_child_illness] = '1'	STOP SURVEY - NOT ELIGIBLE	text<br>Custom alignment: LV
44	outcome_date	When was the child delivered?	text (date_dmy)<br>Field Annotation: @HIDEBUTTON
45	outcome_type	What was the birth outcome?	dropdown<br>1 Live Birth<br>0 Stillbirth
46	en_outcome	Outcome of eligibility screening	dropdown<br>1 Eligible<br>0 Ineligible<br>Custom alignment: LV
47	en_caged_select<br>Show the field ONLY if: [en_outcome] = '1'	Agree to participate in CAGED and did the parents' consent?	radio<br>1 Yes<br>0 No<br>Custom alignment: LV
48	en_excam_select_2	Do eligible subjects agreed to participate in the EXCAM study & did parents' consent.	radio<br>1 Yes<br>0 No<br>Custom alignment: LV
49	en_refusal<br>Show the field ONLY if: [en_caged_select] = '0'	Eligible but not consented - Consent Refusal Interview	descriptive
50	en_refusal_permission<br>Show the field ONLY if: [en_caged_select] = '0'	1. I understand that you are not interested or are unwilling to participate in the research project we have described. Would you be willing to answer a few questions that help me understand that decision?<br>*Continue only if respondents answer YES above.*	yesno<br>1 Yes<br>0 No
51	en_refusal_reason<br>Show the field ONLY if: [en_refusal_permission] = '1'	2. What is the major reason you are not interested in participating?	text
52	en_refusal_past_research<br>Show the field ONLY if: [en_refusal_permission] = '1'	3. Have you participated in any research studies in the past?	yesno<br>1 Yes<br>0 No

53	en_refusal_past_influence<br><br>Show the field ONLY if:<br>[en_refusal_past_research] = '1'	a. If so, did your participation in that study influence your decision not to participate today? Please explain.	text
54	en_refusal_age_year<br><br>Show the field ONLY if:<br>[en_refusal_permission] = '1'	4. What is your age in years?	text (number)
55	en_refusal_educ<br><br>Show the field ONLY if:<br>[en_refusal_permission] = '1'	5. What is your education level?	dropdown<br>1 No formal education<br>2 Some primary<br>3 Completed primary<br>4 Some secondary<br>5 Completed secondary<br>6 Some university<br>7 Graduated university<br>8 Post graduate/professional training
56	en_refusal_married<br><br>Show the field ONLY if:<br>[en_refusal_permission] = '1'	6. Are you married?	yesno<br>1 Yes<br>0 No
57	en_refusal_children<br><br>Show the field ONLY if:<br>[en_refusal_permission] = '1'	7. How many children do you have?	text (number)
58	en_refusal_income<br><br>Show the field ONLY if:<br>[en_refusal_permission] = '1'	8. What is your family's major source of income?	text
59	enrollment_questionnaire_complete	Section Header: *Form Status*<br>Complete?	dropdown<br>0 Incomplete<br>1 Unverified<br>2 Complete
Instrument: **Participant Dropout Interview** (participant_dropout_interview)			Collapse
60	child_deceased	Child deceased?	yesno<br>1 Yes<br>0 No
61	child_deceased_date<br><br>Show the field ONLY if:<br>[child_deceased] = '1'	Date of child passing	text (date_dmy)
62	dropout_participate<br><br>Show the field ONLY if:<br>[child_deceased] = '0'	1. I understand that you have decided not to participate in our research project any longer. I want to thank you for your engagement up to this point. I would also like to ask you a few questions that will help me understand your decision to leave the study. Would you be willing to answer a few questions? We accept your decision to leave the study and will not try to change your mind; this information will simply help us understand the context of your decision to leave.	yesno<br>1 Yes<br>0 No<br><br>Custom alignment: LV
63	dropout_reason<br><br>Show the field ONLY if:<br>[dropout_participate] = '1'	2. What is the major reason you have decided to no longer participate in the study?	text<br>Custom alignment: LV
64	dropout_done_differently<br><br>Show the field ONLY if:<br>[dropout_participate] = '1'	3. Is there anything we could have done differently as researchers to keep you engaged in the study?	text<br>Custom alignment: LV
65	dropout_experience<br><br>Show the field ONLY if:<br>[dropout_participate] = '1'	4. Is there anything you'd like for us to know or understand about your experience as a participant up to this point?	text<br>Custom alignment: LV

66	dropout_keep_samples	5. We have already collected data and samples while you were participating in the study. Do you allow us to keep these data and samples and analyze them?	yesno<br>1 Yes<br>0 No<br><br>Custom alignment: LV
67	participant_dropout_interview_complete	Section Header: *Form Status*<br>Complete?	dropdown<br>0 Incomplete<br>1 Unverified<br>2 Complete
Instrument: **Short Survey** (short_survey)			Collapse
68	ss_date_int	Date of Interview:	text (date_dmy)<br>Custom alignment: LV<br>Field Annotation: @HIDEBUTTON
69	ss_date_int_2	Date of Interview: Confirmation - enter again	text (date_dmy)<br>Custom alignment: LV<br>Field Annotation: @HIDEBUTTON
70	dates_not_matching<br>Show the field ONLY if:<br>[ss_date_int] <> [ss_date_int_2]	Dates do not match - Please review	descriptive
71	ss_data_collector	Data collector's name:	text<br>Custom alignment: LV
72	ss_lat	Latitude	text, Identifier<br>Custom alignment: LV
73	ss_long	Longitude	text, Identifier<br>Custom alignment: LV
74	ss_verification	Section Header: *Please verify the household ID, mothers name, the child's name and DOB.*<br>Household ID: [enrollment_arm_1][en_hh_id] , Mother's name: [enrollment_arm_1][en_mother_fname] [en_mother_mname] [en_mother_lname] , Child's first name: [month_1_arm_1][en_child_fname] , Child's father's name: [enrollment_arm_1][en_father_fname] [en_father_mname] [en_father_lname] , Child's grandfather's name: [enrollment_arm_1][en_grandfather_fname] , Child's sex: [enrollment_arm_1][en_child_sex] , Child's DOB: [enrollment_arm_1][en_child_dob] , Kebele: [enrollment_arm_1][en_kebele_id] ,Village/Ganda ID: [enrollment_arm_1][en_village_id] ,Latitude: [enrollment_arm_1][en_latitude] , Longitude: [enrollment_arm_1][en_longitude]	radio, Identifier<br>1 Verified<br>0 Unverified
75	ss_ao_new_animal	Section Header: *Animal Ownership*<br>Has your household acquired any new animals in the last month, either through birth or purchase?	yesno<br>1 Yes<br>0 No
76	ss_ao_new_animal_y<br>Show the field ONLY if:<br>[ss_ao_new_animal] = '1'	If yes, what animals have you acquired and how many	checkbox<br>1 ss_ao_new_animal_y___1 Cattle<br>2 ss_ao_new_animal_y___2 Horse, Donkey Mule<br>3 ss_ao_new_animal_y___3 Goat<br>4 ss_ao_new_animal_y___4 Sheep<br>5 ss_ao_new_animal_y___5 Chicken<br>6 ss_ao_new_animal_y___6 Dog<br>7 ss_ao_new_animal_y___7 Cat
77	ss_ao_cattle<br>Show the field ONLY if:<br>[ss_ao_new_animal_y(1)] = '1'	Cattle (number)	text (number)
78	ss_ao_horse<br>Show the field ONLY if:<br>[ss_ao_new_animal_y(2)] = '1'	Horse, Donkey, Mule (number)	text (number)
79	ss_ao_goat<br>Show the field ONLY if:<br>[ss_ao_new_animal_y(3)] = '1'	Goat (number)	text (number)

80	ss_ao_sheep<br>Show the field ONLY if:<br>[ss_ao_new_animal_y(4)] = '1'	Sheep (number)	text (number)
81	ss_ao_chicken<br>Show the field ONLY if:<br>[ss_ao_new_animal_y(5)] = '1'	Chicken (number)	text (number)
82	ss_ao_dog<br>Show the field ONLY if:<br>[ss_ao_new_animal_y(6)] = '1'	Dog (number)	text (number)
83	ss_ao_cat<br>Show the field ONLY if:<br>[ss_ao_new_animal_y(7)] = '1'	Cat (number)	text (number)
84	ss_ao_animals_died	Have any of your animals died in the last month?	yesno<br>1 Yes<br>0 No
85	ss_ao_animals_died_y<br>Show the field ONLY if:<br>[ss_ao_animals_died] = '1'	What animals have died in the past month and how many? (Check all that apply)	checkbox<br>1 ss_ao_animals_died_y___1 Cattle<br>2 ss_ao_animals_died_y___2 Horse, Donkey Mule<br>3 ss_ao_animals_died_y___3 Goat<br>4 ss_ao_animals_died_y___4 Sheep<br>5 ss_ao_animals_died_y___5 Chicken<br>6 ss_ao_animals_died_y___6 Dog<br>7 ss_ao_animals_died_y___7 Cat
86	ss_ao_dead_cattle<br>Show the field ONLY if:<br>[ss_ao_animals_died_y(1)] = '1'	Cattle (number)	text (number)
87	ss_ao_dead_horse<br>Show the field ONLY if:<br>[ss_ao_animals_died_y(2)] = '1'	Horse, Donkey, Mule (number)	text (number)
88	ss_ao_dead_goat<br>Show the field ONLY if:<br>[ss_ao_animals_died_y(3)] = '1'	Goat (number)	text (number)
89	ss_ao_dead_sheep<br>Show the field ONLY if:<br>[ss_ao_animals_died_y(4)] = '1'	Sheep (number)	text (number)
90	ss_ao_dead_chicken<br>Show the field ONLY if:<br>[ss_ao_animals_died_y(5)] = '1'	Chicken (number)	text (number)
91	ss_ao_dead_dog<br>Show the field ONLY if:<br>[ss_ao_animals_died_y(6)] = '1'	Dog (number)	text (number)
92	ss_ao_dead_cat<br>Show the field ONLY if:<br>[ss_ao_animals_died_y(7)] = '1'	Cat (number)	text (number)
93	cl_phy_contact	Section Header: *Child livestock contact*<br>Does [month_1_arm_1][en_child_fname] have physical contact (touch) with household animals?	yesno<br>1 Yes<br>0 No

Havelaar AH, *et al. BMJ Open* 2022; 12:e061311. doi: 10.1136/bmjopen-2022-061311

94	cl_animal_contact_y<br>Show the field ONLY if:<br>[cl_phy_contact] = '1'	Which animals? (check all that apply)	checkbox<br>1 cl_animal_contact_y___1 Cattle<br>2 cl_animal_contact_y___2 Horse, Donkey Mule<br>3 cl_animal_contact_y___3 Goat<br>4 cl_animal_contact_y___4 Sheep<br>5 cl_animal_contact_y___5 Chicken<br>6 cl_animal_contact_y___6 Dog<br>7 cl_animal_contact_y___7 Cat
95	cl_crawl_droppings	Does s/he crawl through areas where animal droppings may be present?	dropdown<br>1 Yes<br>0 No<br>2 No, s/he doesn't crawl yet<br>99 Don't know/No Answer
96	cl_soil_mouth	Does s/he ever put non-food items such as soil or animal feces in their mouth?	yesno<br>1 Yes<br>0 No
97	cl_prevent_soil	Are there things you do to try to prevent this?	dropdown<br>1 Yes<br>0 No<br>99 Don't Know/No Answer
98	cl_prevent_soil_action<br>Show the field ONLY if:<br>[cl_prevent_soil] = '1'	What do you do to prevent this?	dropdown<br>1 Cleaning the house<br>2 Carry the child<br>3 Cleaning the yard<br>88 Other (specify below)
99	cl_prevent_soil_other<br>Show the field ONLY if:<br>[cl_prevent_soil_action] = '88'	Specify other:	text
100	cl_soil_mouth_freq	How often would you estimate that s/he puts non-food item (such as soil or animal feces) in their mouth?	dropdown<br>1 Less than once a day<br>2 Once a day<br>3 More than once a day<br>99 Don't know<br>88 Other (Specify)
101	cl_soil_mouth_freq_o<br>Show the field ONLY if:<br>[cl_soil_mouth_freq] = '88'	Other (specify)	text
102	fs_enough_food	Section Header: *Food Security*<br>1. In the past four weeks, did you worry that your household would not have enough food?	yesno, Required<br>1 Yes<br>0 No
103	fs_enough_food_y<br>Show the field ONLY if:<br>[fs_enough_food] = '1'	If yes, how often did this happen?	dropdown, Required<br>1 Rarely (once or twice in the past four weeks)<br>2 Sometimes (three to ten times in the past four weeks)<br>3 Often (more than ten times in the past four weeks)
104	fs_food_preferred	2. In the past four weeks, were you or any household member not able to eat the kinds of<br>foods you preferred because of a lack of resources?	yesno, Required<br>1 Yes<br>0 No

Havelaar AH, *et al. BMJ Open* 2022; 12:e061311. doi: 10.1136/bmjopen-2022-061311

105	fs_food_preferred_y<br>Show the field ONLY if:<br>[fs_food_preferred] = '1'	If yes, how often did this happen?	dropdown, Required<br>1 Rarely (once or twice in the past four weeks)<br>2 Sometimes (three to ten times in the past four weeks)<br>3 Often (more than ten times in the past four weeks)
106	fs_food_variety	3. In the past four weeks, did you or any household member have to eat a limited variety of foods due to a lack of resources?	yesno, Required<br>1 Yes<br>0 No
107	fs_food_variety_y<br>Show the field ONLY if:<br>[fs_food_variety] = '1'	If yes, how often did this happen?	dropdown, Required<br>1 Rarely (once or twice in the past four weeks)<br>2 Sometimes (three to ten times in the past four weeks)<br>3 Often (more than ten times in the past four weeks)
108	fs_food_same	4. In the past four weeks, did you or any household member have to eat some foods that<br>you really did not want to eat because of a lack of resources to obtain other types of<br>food?	yesno, Required<br>1 Yes<br>0 No
109	fs_food_same_y<br>Show the field ONLY if:<br>[fs_food_same] = '1'	If yes, how often did this happen?	dropdown, Required<br>1 Rarely (once or twice in the past four weeks)<br>2 Sometimes (three to ten times in the past four weeks)<br>3 Often (more than ten times in the past four weeks)
110	fs_food_small	5. In the past four weeks, did you or any household member have to eat a smaller meal than<br>you felt you needed because there was not enough food?	yesno, Required<br>1 Yes<br>0 No
111	fs_food_small_y<br>Show the field ONLY if:<br>[fs_food_small] = '1'	If yes, how often did this happen?	dropdown, Required<br>1 Rarely (once or twice in the past four weeks)<br>2 Sometimes (three to ten times in the past four weeks)<br>3 Often (more than ten times in the past four weeks)
112	fs_food_meals	6. In the past four weeks, did you or any household member have to eat fewer meals in a<br>day because there was not enough food?	yesno, Required<br>1 Yes<br>0 No
113	fs_food_meals_y<br>Show the field ONLY if:<br>[fs_food_meals] = '1'	If yes, how often did this happen?	dropdown, Required<br>1 Rarely (once or twice in the past four weeks)<br>2 Sometimes (three to ten times in the past four weeks)<br>3 Often (more than ten times in the past four weeks)
114	fs_food_none	7. In the past four weeks, was there ever no food to eat of any kind in your household<br>because of lack of resources to get food?	yesno, Required<br>1 Yes<br>0 No
115	fs_food_none_y<br>Show the field ONLY if:<br>[fs_food_none] = '1'	If yes, how often did this happen?	dropdown, Required<br>1 Rarely (once or twice in the past four weeks)<br>2 Sometimes (three to ten times in the past four weeks)<br>3 Often (more than ten times in the past four weeks)
116	fs_food_hungry_nt	8. In the past four weeks, did you or any household member go to sleep at night hungry<br>because there was not enough food?	yesno, Required<br>1 Yes<br>0 No

Havelaar AH, *et al. BMJ Open* 2022; 12:e061311. doi: 10.1136/bmjopen-2022-061311

117	fs_food_hungry_nt_y<br>Show the field ONLY if:<br>[fs_food_hungry_nt] = '1'	If yes, how often did this happen?	dropdown, Required<br>1 Rarely (once or twice in the past four weeks)<br>2 Sometimes (three to ten times in the past four weeks)<br>3 Often (more than ten times in the past four weeks)
118	fs_food_hungry_day	9. In the past four weeks, did you or any household member go a whole day and night<br>without eating anything because there was not enough food?	yesno, Required<br>1 Yes<br>0 No
119	fs_food_hungry_day_y<br>Show the field ONLY if:<br>[fs_food_hungry_day] = '1'	If yes, how often did this happen?	dropdown, Required<br>1 Rarely (once or twice in the past four weeks)<br>2 Sometimes (three to ten times in the past four weeks)<br>3 Often (more than ten times in the past four weeks)
120	ch_diar_script	Section Header: *Child's Health*<br>Diarrhea - Diarrhea is defined by the WHO as the passage of three or more loose or liquid stools per day (or more frequent passage than is normal for the individual).	descriptive
121	ch_diar_today	Did the child have a loose stool/diarrhea today?	dropdown<br>1 Yes<br>0 No<br>99 Don't know
122	ch_diar_yesterday	Did the child have loose stool/diarrhea yesterday?	dropdown<br>1 Yes<br>0 No<br>99 Don't know
123	ch_diar_red<br>Show the field ONLY if:<br>[ch_diar_today] = '1' or [ch_diar_yesterday] = '1'	If yes: Was it red or have blood in it?	yesno<br>1 Yes<br>0 No
124	ch_diar_green<br>Show the field ONLY if:<br>[ch_diar_today] = '1' or [ch_diar_yesterday] = '1'	If yes: Was it green?	yesno<br>1 Yes<br>0 No
125	ch_stool_24h<br>Show the field ONLY if:<br>[ch_diar_today] = '1' or [ch_diar_yesterday] = '1'	About how many stools were passed in 24 hours?	text (number)
126	ch_stool_month	In the past month, since our last visit, has [month_1_arm_1][en_child_fname] had loose stools/diarrhea?	dropdown<br>1 Yes<br>0 No<br>99 Don't know
127	ch_stool_last_day<br>Show the field ONLY if:<br>[ch_stool_month] = '1'	When was the last day s/he suffered from diarrhea (estimated day of last diarrhea)? Days ago (number)	text (number)
128	ch_stool_last_week<br>Show the field ONLY if:<br>[ch_stool_month] = '1'	When was the last day s/he suffered from diarrhea (estimated day of last diarrhea)? Weeks ago (number)	text (number)

129	ch_diar_days_suffer<br>Show the field ONLY if:<br>[ch_stool_month] = '1'	How many days had the child suffered from diarrhea?	dropdown<br>1 1<br>2 2<br>3 3<br>4 4<br>5 5<br>6 6<br>7 More
130	ch_diar_red_month<br>Show the field ONLY if:<br>[ch_stool_month] = '1'	If yes: Was it red or have blood in it?	yesno<br>1 Yes<br>0 No
131	ch_diar_green_month<br>Show the field ONLY if:<br>[ch_stool_month] = '1'	If yes: Was it green?	yesno<br>1 Yes<br>0 No
132	ch_diar_drink	When [month_1_arm_1][en_child_fname] suffered from diarrhea, how much was s/he given to drink, including breastmilk? Was [month_1_arm_1][en_child_fname] given nothing to drink, less than usual to drink, about the same amount, more than usual to drink, or a lot more than usual to drink?	dropdown<br>1 Nothing/a lot less to drink<br>2 Less than usual<br>3 About the same amount<br>4 More than usual<br>5 A lot more than usual
133	ch_diar_food	What about food? When [month_1_arm_1][en_child_fname] suffered from diarrhea, was [month_1_arm_1][en_child_fname] given nothing to eat, less than usual to eat, about the same amount, more than usual, or a lot more to eat?	dropdown<br>1 Nothing/a lot less<br>2 Less<br>3 About the same<br>4 More<br>5 A lot more
134	ch_diar_ors	Was [month_1_arm_1][en_child_fname] given oral rehydration salts (ORS)?	yesno<br>1 Yes<br>0 No
135	ch_diar_fever	Did the child have fever when s/he had diarrhea?	yesno<br>1 Yes<br>0 No
136	ch_diar_health_center	Was the child taken to a health center when s/he had diarrhea?	yesno<br>1 Yes<br>0 No
137	ch_fever	Section Header: *Fever*<br>Does [month_1_arm_1][en_child_fname] have a fever now?	yesno<br>1 Yes<br>0 No
138	ch_fever_yesterday	Did the child have fever yesterday:	yesno<br>1 Yes<br>0 No
139	ch_fever_lastvisit	In the past month, since our last visit, has [month_1_arm_1][en_child_fname] suffered from a fever?	yesno<br>1 Yes<br>0 No
140	ch_fever_days_ago<br>Show the field ONLY if:<br>[ch_fever_lastvisit] = '1'	When was the last time [month_1_arm_1][en_child_fname] had a fever? Days ago (number)	text (number)
141	ch_fever_weeks_ago<br>Show the field ONLY if:<br>[ch_fever_lastvisit] = '1'	When was the last time [month_1_arm_1][en_child_fname] had a fever? Weeks ago (number)	text (number)

142	ch_fever_lasted<br>Show the field ONLY if:<br>[ch_fever_lastvisit] = '1'	For how many days did [month_1_arm_1][en_child_fname] 's fever last?	text (number)
143	ch_fever_drink	When [month_1_arm_1][en_child_fname] had a fever, how much was s/he given to drink, including breastmilk? Was [month_1_arm_1][en_child_fname] given nothing to drink, less than usual to drink, about the same amount, more than usual to drink, or a lot more than usual to drink?	dropdown<br>1 Nothing/a lot less<br>2 Less<br>3 About the same<br>4 More<br>5 A lot more
144	ch_fever_food	What about food? When [month_1_arm_1][en_child_fname] had a fever, was [month_1_arm_1][en_child_fname] given nothing to eat, less than usual to eat, about the same amount, more than usual, or a lot more to eat?	dropdown<br>1 Nothing/a lot less<br>2 Less<br>3 About the same<br>4 More<br>5 A lot more
145	ch_fever_healthcenter	Was the child taken to a health center when s/he had this fever?	yesno<br>1 Yes<br>0 No
146	ch_cough	Section Header: *Cough*<br>Does [month_1_arm_1][en_child_fname] have a cough today?	yesno<br>1 Yes<br>0 No
147	ch_cough_yesterday	Did the child have cough yesterday:	yesno<br>1 Yes<br>0 No
148	ch_cough_lastvisit	Has the child had a cough in the past month, since our last visit?	yesno<br>1 Yes<br>0 No
149	ch_cough_days_ago<br>Show the field ONLY if:<br>[ch_cough_lastvisit] = '1'	When was the last time s/he had a cough? Days ago (number)	text (number)
150	ch_cough_weeks_ago<br>Show the field ONLY if:<br>[ch_cough_lastvisit] = '1'	When was the last time s/he had a cough? Weeks ago (number)	text (number)
151	ch_cough_lasted<br>Show the field ONLY if:<br>[ch_cough_lastvisit] = '1'	How long did the cough last? (days)	text (number)
152	ch_cough_healthcenter	Was the child taken to a health center when s/he had this cough?	yesno<br>1 Yes<br>0 No
153	ch_abx	Section Header: *Antibiotics*<br>Has the child been treated with an antibiotics in the past month, since our last visit?	yesno<br>1 Yes<br>0 No
154	ch_abx_name<br>Show the field ONLY if:<br>[ch_abx] = '1'	Do you know the name of antibiotic?	yesno<br>1 Yes<br>0 No

Havelaar AH, *et al. BMJ Open* 2022; 12:e061311. doi: 10.1136/bmjopen-2022-061311

Supplemental material BMJ Publishing Group Limited (BMJ) disclaims all liability and responsibility arising from any reliance placed on this supplemental material which has been supplied by the author(s) BMJ Open

155	ch_abx_selection<br>Show the field ONLY if:<br>[ch_abx_name] = '1'	Select antibiotic from list below	checkbox<br>1 ch_abx_selection___1 Ampcillin<br>2 ch_abx_selection___2 Amoxicillin<br>3 ch_abx_selection___3 Cotrimoxazole<br>4 ch_abx_selection___4 Ceftriaxone<br>5 ch_abx_selection___5 Ciprofloxacin<br>6 ch_abx_selection___6 Erythromycin<br>7 ch_abx_selection___7 Azithromycin<br>8 ch_abx_selection___8 Cephalexin<br>88 ch_abx_selection___88 Others
156	ch_abx_packaging<br>Show the field ONLY if:<br>[ch_abx_name] = '0'	No, please provide a description of the antibiotic packaging	text
157	ch_abx_long_ago0<br>Show the field ONLY if:<br>[ch_abx_name] = '0'	How long ago was the child treated with this antibiotic?	radio<br>1 Days ago<br>2 Weeks ago
158	ch_abx_days_ago0<br>Show the field ONLY if:<br>[ch_abx_long_ago0] = '1'	When was the last time s/he was treated? Days ago	text (number)
159	ch_abx_weeks_ago0<br>Show the field ONLY if:<br>[ch_abx_long_ago0] = '2'	When was the last time s/he was treated? Weeks ago	text (number)
160	ch_abx_taking0<br>Show the field ONLY if:<br>[ch_abx_name] = '0'	Is s/he still taking antibiotics?	yesno<br>1 Yes<br>0 No
161	ch_abx_symptoms0<br>Show the field ONLY if:<br>[ch_abx_name] = '0'	What were his/her symptoms?	text
162	ch_abx_completed0<br>Show the field ONLY if:<br>[ch_abx_name] = '0'	Did your child complete the antibiotics as advised by a clinician?	yesno<br>1 Yes<br>0 No
163	ch_abx_days_taken0<br>Show the field ONLY if:<br>[ch_abx_name] = '0'	HI14d) How many days did your child take the antibiotics (days)	dropdown<br>1 1<br>2 2<br>3 3<br>4 4<br>5 5<br>6 6<br>7 7<br>8 8<br>9 9<br>10 More
164	ch_abx_days_spec0<br>Show the field ONLY if:<br>[ch_abx_days_taken0] = '10'	Specify number of days	text (number)
165	ch_abx_other<br>Show the field ONLY if:<br>[ch_abx_selection(88)] = '1'	Name of Other antibiotic	text
166	ch_abx_long_ago88<br>Show the field ONLY if:<br>[ch_abx_selection(88)] = '1'	How long ago was the child treated with this Other antibiotic?	radio<br>1 Days ago<br>2 Weeks ago

Havelaar AH, *et al. BMJ Open* 2022; 12:e061311. doi: 10.1136/bmjopen-2022-061311

167	ch_abx_days_ago88<br>Show the field ONLY if:<br>[ch_abx_long_ago88] = '1'	When was the last time s/he was treated? Days ago	text (number)
168	ch_abx_weeks_ago88<br>Show the field ONLY if:<br>[ch_abx_long_ago88] = '2'	When was the last time s/he was treated? Weeks ago	text (number)
169	ch_abx_taking88<br>Show the field ONLY if:<br>[ch_abx_selection(88)] = '1'	Is s/he still taking antibiotics?	yesno<br>1 Yes<br>0 No
170	ch_abx_symptoms88<br>Show the field ONLY if:<br>[ch_abx_selection(88)] = '1'	What were his/her symptoms?	text
171	ch_abx_completed88<br>Show the field ONLY if:<br>[ch_abx_selection(88)] = '1'	Did your child complete the antibiotics as advised by a clinician?	yesno<br>1 Yes<br>0 No
172	ch_abx_days_taken88<br>Show the field ONLY if:<br>[ch_abx_selection(88)] = '1'	HI14d) How many days did your child take the antibiotics (days)	dropdown<br>1 1<br>2 2<br>3 3<br>4 4<br>5 5<br>6 6<br>7 7<br>8 8<br>9 9<br>10 More
173	ch_abx_days_spec88<br>Show the field ONLY if:<br>[ch_abx_days_taken88] = '10'	Specify number of days	text (number)
174	ch_abx_1<br>Show the field ONLY if:<br>[ch_abx_selection(1)] = '1'	Antibiotic 1	descriptive
175	ch_abx_long_ago1<br>Show the field ONLY if:<br>[ch_abx_selection(1)] = '1'	How long ago was the child treated with this antibiotic?	radio<br>1 Days ago<br>2 Weeks ago
176	ch_abx_days_ago1<br>Show the field ONLY if:<br>[ch_abx_long_ago1] = '1'	When was the last time s/he was treated? Days ago	text (number)
177	ch_abx_weeks_ago1<br>Show the field ONLY if:<br>[ch_abx_long_ago1] = '2'	When was the last time s/he was treated? Weeks ago	text (number)
178	ch_abx_taking1<br>Show the field ONLY if:<br>[ch_abx_selection(1)] = '1'	Is s/he still taking antibiotics?	yesno<br>1 Yes<br>0 No
179	ch_abx_symptoms1<br>Show the field ONLY if:<br>[ch_abx_selection(1)] = '1'	What were his/her symptoms?	text
180	ch_abx_completed1<br>Show the field ONLY if:<br>[ch_abx_selection(1)] = '1'	Did your child complete the antibiotics as advised by a clinician?	yesno<br>1 Yes<br>0 No

181	ch_abx_days_taken1<br>Show the field ONLY if:<br>[ch_abx_selection(1)] = '1'	HI14d) How many days did your child take the antibiotics (days)	dropdown<br>1 1<br>2 2<br>3 3<br>4 4<br>5 5<br>6 6<br>7 7<br>8 8<br>9 9<br>10 More
182	ch_abx_days_spec1<br>Show the field ONLY if:<br>[ch_abx_days_taken1] = '10'	Specify number of days	text (number)
183	ch_abx_2<br>Show the field ONLY if:<br>[ch_abx_selection(2)] = '1'	Antibiotic 2	descriptive
184	ch_abx_long_ago2<br>Show the field ONLY if:<br>[ch_abx_selection(2)] = '1'	How long ago was the child treated with this antibiotic?	radio<br>1 Days ago<br>2 Weeks ago
185	ch_abx_days_ago2<br>Show the field ONLY if:<br>[ch_abx_long_ago2] = '1'	When was the last time s/he was treated? Days ago	text (number)
186	ch_abx_weeks_ago2<br>Show the field ONLY if:<br>[ch_abx_long_ago2] = '2'	When was the last time s/he was treated? Weeks ago	text (number)
187	ch_abx_taking2<br>Show the field ONLY if:<br>[ch_abx_selection(2)] = '1'	Is s/he still taking antibiotics?	yesno<br>1 Yes<br>0 No
188	ch_abx_symptoms2<br>Show the field ONLY if:<br>[ch_abx_selection(2)] = '1'	What were his/her symptoms?	text
189	ch_abx_completed2<br>Show the field ONLY if:<br>[ch_abx_selection(2)] = '1'	Did your child complete the antibiotics as advised by a clinician?	yesno<br>1 Yes<br>0 No
190	ch_abx_days_taken2<br>Show the field ONLY if:<br>[ch_abx_selection(2)] = '1'	HI14d) How many days did your child take the antibiotics (days)	dropdown<br>1 1<br>2 2<br>3 3<br>4 4<br>5 5<br>6 6<br>7 7<br>8 8<br>9 9<br>10 More
191	ch_abx_days_spec2<br>Show the field ONLY if:<br>[ch_abx_days_taken2] = '10'	Specify number of days	text (number)
192	ch_abx_3<br>Show the field ONLY if:<br>[ch_abx_selection(3)] = '1'	Antibiotic 3	descriptive

193	ch_abx_long_ago3<br>Show the field ONLY if:<br>[ch_abx_selection(3)] = '1'	How long ago was the child treated with this antibiotic?	radio<br>1 Days ago<br>2 Weeks ago
194	ch_abx_days_ago3<br>Show the field ONLY if:<br>[ch_abx_long_ago3] = '1'	When was the last time s/he was treated? Days ago	text (number)
195	ch_abx_weeks_ago3<br>Show the field ONLY if:<br>[ch_abx_long_ago3] = '2'	When was the last time s/he was treated? Weeks ago	text (number)
196	ch_abx_taking3<br>Show the field ONLY if:<br>[ch_abx_selection(3)] = '1'	Is s/he still taking antibiotics?	yesno<br>1 Yes<br>0 No
197	ch_abx_symptoms3<br>Show the field ONLY if:<br>[ch_abx_selection(3)] = '1'	What were his/her symptoms?	text
198	ch_abx_completed3<br>Show the field ONLY if:<br>[ch_abx_selection(3)] = '1'	Did your child complete the antibiotics as advised by a clinician?	yesno<br>1 Yes<br>0 No
199	ch_abx_days_taken3<br>Show the field ONLY if:<br>[ch_abx_selection(3)] = '1'	HI14d) How many days did your child take the antibiotics (days)	dropdown<br>1 1<br>2 2<br>3 3<br>4 4<br>5 5<br>6 6<br>7 7<br>8 8<br>9 9<br>10 More
200	ch_abx_days_spec3<br>Show the field ONLY if:<br>[ch_abx_days_taken3] = '10'	Specify number of days	text (number)
201	ch_abx_4<br>Show the field ONLY if:<br>[ch_abx_selection(4)] = '1'	Antibiotic 4	descriptive
202	ch_abx_long_ago4<br>Show the field ONLY if:<br>[ch_abx_selection(4)] = '1'	How long ago was the child treated with this antibiotic?	radio<br>1 Days ago<br>2 Weeks ago
203	ch_abx_days_ago4<br>Show the field ONLY if:<br>[ch_abx_long_ago4] = '1'	When was the last time s/he was treated? Days ago	text (number)
204	ch_abx_weeks_ago4<br>Show the field ONLY if:<br>[ch_abx_long_ago4] = '2'	When was the last time s/he was treated? Weeks ago	text (number)
205	ch_abx_taking4<br>Show the field ONLY if:<br>[ch_abx_selection(4)] = '1'	Is s/he still taking antibiotics?	yesno<br>1 Yes<br>0 No
206	ch_abx_symptoms4<br>Show the field ONLY if:<br>[ch_abx_selection(4)] = '1'	What were his/her symptoms?	text

207	ch_abx_completed4<br>Show the field ONLY if:<br>[ch_abx_selection(4)] = '1'	Did your child complete the antibiotics as advised by a clinician?	yesno<br>1 Yes<br>0 No
208	ch_abx_days_taken4<br>Show the field ONLY if:<br>[ch_abx_selection(4)] = '1'	HI14d) How many days did your child take the antibiotics (days)	dropdown<br>1 1<br>2 2<br>3 3<br>4 4<br>5 5<br>6 6<br>7 7<br>8 8<br>9 9<br>10 More
209	ch_abx_days_spec4<br>Show the field ONLY if:<br>[ch_abx_days_taken4] = '10'	Specify number of days	text (number)
210	ch_abx_5<br>Show the field ONLY if:<br>[ch_abx_selection(5)] = '1'	Antibiotic 5	descriptive
211	ch_abx_long_ago5<br>Show the field ONLY if:<br>[ch_abx_selection(5)] = '1'	How long ago was the child treated with this antibiotic?	radio<br>1 Days ago<br>2 Weeks ago
212	ch_abx_days_ago5<br>Show the field ONLY if:<br>[ch_abx_long_ago5] = '1'	When was the last time s/he was treated? Days ago	text (number)
213	ch_abx_weeks_ago5<br>Show the field ONLY if:<br>[ch_abx_long_ago5] = '2'	When was the last time s/he was treated? Weeks ago	text (number)
214	ch_abx_taking5<br>Show the field ONLY if:<br>[ch_abx_selection(5)] = '1'	Is s/he still taking antibiotics?	yesno<br>1 Yes<br>0 No
215	ch_abx_symptoms5<br>Show the field ONLY if:<br>[ch_abx_selection(5)] = '1'	What were his/her symptoms?	text
216	ch_abx_completed5<br>Show the field ONLY if:<br>[ch_abx_selection(5)] = '1'	Did your child complete the antibiotics as advised by a clinician?	yesno<br>1 Yes<br>0 No
217	ch_abx_days_taken5<br>Show the field ONLY if:<br>[ch_abx_selection(5)] = '1'	HI14d) How many days did your child take the antibiotics (days)	dropdown<br>1 1<br>2 2<br>3 3<br>4 4<br>5 5<br>6 6<br>7 7<br>8 8<br>9 9<br>10 More
218	ch_abx_days_spec5<br>Show the field ONLY if:<br>[ch_abx_days_taken5] = '10'	Specify number of days	text (number)

219	ch_abx_6<br>Show the field ONLY if:<br>[ch_abx_selection(6)] = '1'	Antibiotic 6	descriptive
220	ch_abx_long_ago6<br>Show the field ONLY if:<br>[ch_abx_selection(6)] = '1'	How long ago was the child treated with this antibiotic?	radio<br>1 Days ago<br>2 Weeks ago
221	ch_abx_days_ago6<br>Show the field ONLY if:<br>[ch_abx_long_ago6] = '1'	When was the last time s/he was treated? Days ago	text (number)
222	ch_abx_weeks_ago6<br>Show the field ONLY if:<br>[ch_abx_long_ago6] = '2'	When was the last time s/he was treated? Weeks ago	text (number)
223	ch_abx_taking6<br>Show the field ONLY if:<br>[ch_abx_selection(6)] = '1'	Is s/he still taking antibiotics?	yesno<br>1 Yes<br>0 No
224	ch_abx_symptoms6<br>Show the field ONLY if:<br>[ch_abx_selection(6)] = '1'	What were his/her symptoms?	text
225	ch_abx_completed6<br>Show the field ONLY if:<br>[ch_abx_selection(6)] = '1'	Did your child complete the antibiotics as advised by a clinician?	yesno<br>1 Yes<br>0 No
226	ch_abx_days_taken6<br>Show the field ONLY if:<br>[ch_abx_selection(6)] = '1'	HI14d) How many days did your child take the antibiotics (days)	dropdown<br>1 1<br>2 2<br>3 3<br>4 4<br>5 5<br>6 6<br>7 7<br>8 8<br>9 9<br>10 More
227	ch_abx_days_spec6<br>Show the field ONLY if:<br>[ch_abx_days_taken6] = '10'	Specify number of days	text (number)
228	ch_abx_7<br>Show the field ONLY if:<br>[ch_abx_selection(7)] = '1'	Antibiotic 7	descriptive
229	ch_abx_long_ago7<br>Show the field ONLY if:<br>[ch_abx_selection(7)] = '1'	How long ago was the child treated with this antibiotic?	radio<br>1 Days ago<br>2 Weeks ago
230	ch_abx_days_ago7<br>Show the field ONLY if:<br>[ch_abx_long_ago7] = '1'	When was the last time s/he was treated? Days ago	text (number)
231	ch_abx_weeks_ago7<br>Show the field ONLY if:<br>[ch_abx_long_ago7] = '2'	When was the last time s/he was treated? Weeks ago	text (number)
232	ch_abx_taking7<br>Show the field ONLY if:<br>[ch_abx_selection(7)] = '1'	Is s/he still taking antibiotics?	yesno<br>1 Yes<br>0 No

Havelaar AH, *et al. BMJ Open* 2022; 12:e061311. doi: 10.1136/bmjopen-2022-061311

233	ch_abx_symptoms7<br>Show the field ONLY if:<br>[ch_abx_selection(7)] = '1'	What were his/her symptoms?	text
234	ch_abx_completed7<br>Show the field ONLY if:<br>[ch_abx_selection(7)] = '1'	Did your child complete the antibiotics as advised by a clinician?	yesno<br>1 Yes<br>0 No
235	ch_abx_days_taken7<br>Show the field ONLY if:<br>[ch_abx_selection(7)] = '1'	HI14d) How many days did your child take the antibiotics (days)	dropdown<br>1 1<br>2 2<br>3 3<br>4 4<br>5 5<br>6 6<br>7 7<br>8 8<br>9 9<br>10 More
236	ch_abx_days_spec7<br>Show the field ONLY if:<br>[ch_abx_days_taken7] = '10'	Specify number of days	text (number)
237	ch_abx_8<br>Show the field ONLY if:<br>[ch_abx_selection(8)] = '1'	Antibiotic 8	descriptive
238	ch_abx_long_ago8<br>Show the field ONLY if:<br>[ch_abx_selection(8)] = '1'	How long ago was the child treated with this antibiotic?	radio<br>1 Days ago<br>2 Weeks ago
239	ch_abx_days_ago8<br>Show the field ONLY if:<br>[ch_abx_long_ago8] = '1'	When was the last time s/he was treated? Days ago	text (number)
240	ch_abx_weeks_ago8<br>Show the field ONLY if:<br>[ch_abx_long_ago8] = '2'	When was the last time s/he was treated? Weeks ago	text (number)
241	ch_abx_taking8<br>Show the field ONLY if:<br>[ch_abx_selection(8)] = '1'	Is s/he still taking antibiotics?	yesno<br>1 Yes<br>0 No
242	ch_abx_symptoms8<br>Show the field ONLY if:<br>[ch_abx_selection(8)] = '1'	What were his/her symptoms?	text
243	ch_abx_completed8<br>Show the field ONLY if:<br>[ch_abx_selection(8)] = '1'	Did your child complete the antibiotics as advised by a clinician?	yesno<br>1 Yes<br>0 No
244	ch_abx_days_taken8<br>Show the field ONLY if:<br>[ch_abx_selection(8)] = '1'	HI14d) How many days did your child take the antibiotics (days)	dropdown<br>1 1<br>2 2<br>3 3<br>4 4<br>5 5<br>6 6<br>7 7<br>8 8<br>9 9<br>10 More

245	ch_abx_days_spec8<br>Show the field ONLY if:<br>[ch_abx_days_taken8] = '10'	Specify number of days	text (number)
246	ch_tradtional_healer	Was your child treated for an infection by a traditional healer	dropdown<br>1 Yes<br>0 No<br>99 Don't know
247	ch_trad_heal_symptoms<br>Show the field ONLY if:<br>[ch_tradtional_healer] = '1'	If yes, what were his/her symptoms?	text
248	ch_trad_heal_treatment<br>Show the field ONLY if:<br>[ch_tradtional_healer] = '1'	What treatment was provided/proscribed by the traditional healer?	text
249	maln_treated	Section Header: *Malnutrition*<br>Has the child been treated for malnutrition in the past month, since our last visit?	dropdown<br>1 Yes<br>0 No<br>99 Don't know
250	maln_treated_currently<br>Show the field ONLY if:<br>[maln_treated] = '1'	Is the child currently being treated for malnutrition?	dropdown<br>1 Yes<br>0 No<br>99 Don't know
251	maln_treat_type<br>Show the field ONLY if:<br>[maln_treated] = '1'	What type of treatment did the child receive?	dropdown<br>1 Inpatient<br>2 Outpatient supplementary feeding program<br>88 Other (specify)<br>99 I don't know
252	maln_treat_type_other<br>Show the field ONLY if:<br>[maln_treat_type] = '88'	Specify other:	text
253	maln_ready_food<br>Show the field ONLY if:<br>[maln_treated] = '1'	Did the child receive Ready-to-use therapeutic foods, such as Plumpynut, from a clinician?	dropdown<br>1 Yes<br>0 No<br>99 Don't know
254	vit_a_past_mo	Section Header: *Vitamins*<br>Has [month_1_arm_1][en_child_fname] been given a vitamin A dose like [this/any of these] in the past month, since our last visit? ** Note: Show common types of Ampules/capsules/syrups.	dropdown<br>1 Yes<br>0 No<br>99 Don't know
255	vit_iron_7days	In the last seven days, was [month_1_arm_1][en_child_fname] given iron pills, sprinkles with iron, or iron syrup like [this/any of these]? ** Note: Show common types of Pills/sprinkles/syrups	dropdown<br>1 Yes<br>0 No<br>99 Don't know
256	vacc_last_mo	Section Header: *Vaccinations*<br>Has the child received vaccinations in the past month, since our last visit?	dropdown<br>1 Yes<br>0 No<br>99 Don't know
257	vacc_documentation	Do you have a card or other document where [month_1_arm_1][en_child_fname] 's vaccinations are written down?	dropdown<br>1 Yes<br>0 No<br>99 Don't know

258	vacc_documentation_show<br>Show the field ONLY if:<br>[vacc_documentation] = '1'	May I see the card or other document where [month_1_arm_1][en_child_fname] 's vaccinations are written down?	descriptive
259	vacc_bcg	BCG	yesno<br>1 Yes<br>0 No
260	vacc_bcg_date	Date of vaccine	text (date_dmy)<br>Field Annotation: @HIDEBUTTON
261	vacc_polio_penta1	Polio 1 and Penta 1	yesno<br>1 Yes<br>0 No
262	vacc_polio_penta1_date	Date of vaccine	text (date_dmy)<br>Field Annotation: @HIDEBUTTON
263	vacc_polio_penta2	Polio 2 and Penta 2	yesno<br>1 Yes<br>0 No
264	vacc_polio_penta2_date	Date of vaccine	text (date_dmy)<br>Field Annotation: @HIDEBUTTON
265	vacc_polio_penta3	Polio 3 and Penta 3	yesno<br>1 Yes<br>0 No
266	vacc_polio_penta3_date	Date of vaccine	text (date_dmy)<br>Field Annotation: @HIDEBUTTON
267	vacc_measles	Measles	yesno<br>1 Yes<br>0 No
268	vacc_measles_date	Date of vaccine	text (date_dmy)<br>Field Annotation: @HIDEBUTTON
269	vacc_ipv	IPV	yesno<br>1 Yes<br>0 No
270	vacc_ipv_date	Date of vaccine	text (date_dmy)<br>Field Annotation: @HIDEBUTTON
271	vacc_rota	Rotavirus	yesno<br>1 Yes<br>0 No
272	vacc_rota_date	Date of vaccine	text (date_dmy)<br>Field Annotation: @HIDEBUTTON
273	vacc_other	Other (specify)	yesno<br>1 Yes<br>0 No
274	vacc_other_specify<br>Show the field ONLY if:<br>[vacc_other] = '1'	Other (specify)	text
275	vacc_other_date	Date of vaccine	text (date_dmy)<br>Field Annotation: @HIDEBUTTON
276	vacc_additonal	In addition to what is recorded on (this document/these documents), did [month_1_arm_1][en_child_fname] receive any other vaccinations, including vaccinations received in campaigns or immunization days or child health days?	yesno<br>1 Yes<br>0 No
277	vacc_additonal_notes<br>Show the field ONLY if:<br>[vacc_additonal] = '1'	If yes, Comment with date received:	notes

278	bf_currently_feed	Section Header: *Breastfeeding*<br>Are you currently breastfeeding [month_1_arm_1][en_child_fname]?	yesno, Required<br>1 Yes<br>0 No
279	bf_stopped<br>Show the field ONLY if:<br>[bf_currently_feed] = '0'	When did the child stop breastfeeding?	dropdown, Required<br>1 Days ago<br>2 Weeks ago<br>99 I don't know
280	bf_stopped_days<br>Show the field ONLY if:<br>[bf_stopped] = '1'	Days ago (number)	text (number), Required
281	bf_stopped_weeks<br>Show the field ONLY if:<br>[bf_stopped] = '2'	Weeks ago (number)	text (number), Required
282	bf_yesterday<br>Show the field ONLY if:<br>[bf_currently_feed] = '1'	Was [month_1_arm_1][en_child_fname] breastfed (or did s/he consume breast milk) yesterday during the day or at night?	yesno, Required<br>1 Yes<br>0 No
283	bf_eat_solids	Have you given [month_1_arm_1][en_child_fname] something to eat other than breastmilk yet?	dropdown, Required<br>0 No, not yet<br>1 Yes
284	bf_eat_solids_age_day<br>Show the field ONLY if:<br>[bf_eat_solids] = '1'	Child age in days (specify _____)<br>*When did you first give [en_child_fname] something to eat other than breastmilk?*	text (number), Required
285	bf_eat_solids_age_wk<br>Show the field ONLY if:<br>[bf_eat_solids] = '1'	Child age in weeks (specify _____)<br>*When did you first give [en_child_fname] something to eat other than breastmilk?*	text (number), Required
286	bf_eat_solids_age_mo<br>Show the field ONLY if:<br>[bf_eat_solids] = '1'	Child age in months (specify _____)<br>*When did you first give [en_child_fname] something to eat other than breastmilk?*	text (number), Required
287	bf_eat_solids_ago_day<br>Show the field ONLY if:<br>[bf_eat_solids] = '1'	Number of days ago (specify _____)<br>*When did you first give [en_child_fname] something to eat other than breastmilk?*	text (number), Required
288	bf_eat_solids_ago_wk<br>Show the field ONLY if:<br>[bf_eat_solids] = '1'	Number of weeks ago (specify _____)<br>*When did you first give [en_child_fname] something to eat other than breastmilk?*	text (number), Required
289	bf_eat_solids_ago_mo<br>Show the field ONLY if:<br>[bf_eat_solids] = '1'	Number of months ago (specify _____)<br>*When did you first give [en_child_fname] something to eat other than breastmilk?*	text (number), Required
290	bf_eat_solids_type<br>Show the field ONLY if:<br>[bf_eat_solids] = '1'	What food/drink was it that you first gave [month_1_arm_1][en_child_fname]?	text, Required
291	bf_eat_am<br>Show the field ONLY if:<br>[bf_eat_solids] = '1'	Did your child eat this morning?	yesno, Required<br>1 Yes<br>0 No
292	bf_eat_am_food<br>Show the field ONLY if:<br>[bf_eat_am] = '1'	What foods did your child eat this morning?	text, Required
293	cn_fem_cn	Section Header: *Child Nutrition*<br>(Female) Child Nutrition<br>Now I would like to ask you about liquids or foods that your child had yesterday during the<br>day or at night. I am interested in whether they consumed the item even if it was combined<br>with other foods.	descriptive

294	cn_24h_water	Section Header: *In past 24 hours has your child had any of these liquids?*<br>Water	dropdown<br>1 Yes<br>0 No<br>99 Don't know
295	cn_24h_water_quan<br>Show the field ONLY if:<br>[cn_24h_water] = '1'	If yes, How many times in the past 24 hours?	text (number)
296	cn_24h_water_sugar<br>Show the field ONLY if:<br>[cn_24h_water] = '1'	If yes, Was it mixed with sugar	dropdown<br>1 Yes<br>0 No<br>99 Don't know
297	cn_24h_formula	Infant Formula such as (enfamil)?	dropdown<br>1 Yes<br>0 No<br>99 Don't know
298	cn_24h_formula_type<br>Show the field ONLY if:<br>[cn_24h_formula] = '1'	If yes, What type? (specify____________)	text
299	cn_24h_formula_times<br>Show the field ONLY if:<br>[cn_24h_formula] = '1'	If yes, How many times in the past 24 hours? (enter number)	text (number)
300	cn_24h_milk	Milk, such as tinned, powdered, or fresh animal milk (do not include breastmilk)?	dropdown<br>1 Yes<br>0 No<br>99 Don't know
301	cn_24h_milk_type<br>Show the field ONLY if:<br>[cn_24h_milk] = '1'	What type:	dropdown<br>1 Tinned<br>2 Powdered<br>3 Animal milk
302	cn_24h_milk_animal<br>Show the field ONLY if:<br>[cn_24h_milk_type] = '3'	What type of animal milk?	dropdown<br>1 Cow<br>2 Goat<br>3 Camel<br>4 Sheep
303	cn_24h_milk_animal_past<br>Show the field ONLY if:<br>[cn_24h_milk_type] = '3'	Do you pasteurize that milk?	dropdown<br>1 Yes<br>0 No<br>99 Don't know
304	cn_24h_milk_any<br>Show the field ONLY if:<br>[cn_24h_milk] = '1'	If yes, How many times in the past 24 hours have you given the child any type of milk?	text
305	cn_24h_juice	Juice or juice drinks?	dropdown<br>1 Yes<br>0 No<br>99 Don't know
306	cn_24h_juice_quan<br>Show the field ONLY if:<br>[cn_24h_juice] = '1'	If yes, How many times in the past 24 hours?	text (number)

Havelaar AH, *et al. BMJ Open* 2022; 12:e061311. doi: 10.1136/bmjopen-2022-061311

307	cn_24h_broth	Clear broth?	dropdown<br>1 Yes<br>0 No<br>99 Don't know
308	cn_24h_broth_quan<br>Show the field ONLY if:<br>[cn_24h_broth] = '1'	If yes, How many times in the past 24 hours?	text (number)
309	cn_24h_yogurt	Yogurt?	dropdown<br>1 Yes<br>0 No<br>99 Don't know
310	cn_24h_yogurt_quan<br>Show the field ONLY if:<br>[cn_24h_yogurt] = '1'	If yes, how many times?	text (number)
311	cn_24h_porridge	Thin porridge?	dropdown<br>1 Yes<br>0 No<br>99 Don't know
312	cn_24h_porr_quan<br>Show the field ONLY if:<br>[cn_24h_porridge] = '1'	If yes, How many times in the past 24 hours?	text (number)
313	cn_24h_liquids	Any other liquids?	dropdown<br>1 Yes<br>0 No<br>99 Don't know
314	cn_24h_liquids_quan<br>Show the field ONLY if:<br>[cn_24h_liquids] = '1'	If yes, how many times?	text (number)
315	cn_24h_nipple	Did [month_1_arm_1][en_child_fname] drink anything from a bottle with a nipple yesterday during the day or night?	dropdown<br>1 Yes<br>0 No<br>99 Don't know
316	cn_24h_solids	Did [month_1_arm_1][en_child_fname] eat any solid, semi solid or soft foods yesterday during the day or at night?	dropdown<br>1 Yes<br>0 No<br>99 Don't know
317	cn_24h_solids_y<br>Show the field ONLY if:<br>[cn_24h_solids] = '1'	If yes, how many times did they eat solid, semi solid or soft foods, other than liquids, yesterday?	text
318	cn_child_agecalc	Child's current calculated age	calc<br>Calculation: datediff ([ss_date_int], [enrollment_arm_1][en_child_dob],"M","dmy")

319	cn_ate_yesterday<br><br>Show the field ONLY if:<br>[cn_child_agecalc] >= 5	Please describe everything that [month_1_arm_1][en_child_fname] ate yesterday during the day or night, whether at home or outside the home. a) Think about when [month_1_arm_1][en_child_fname] first woke up yesterday. Did [month_1_arm_1][en_child_fname] eat anything at that time? If yes: Please tell me everything [month_1_arm_1][en_child_fname] ate at that time. Probe: Anything else? Until respondent says nothing else. If no, continue to Question b). b) What did [month_1_arm_1][en_child_fname] do after that? Did [month_1_arm_1][en_child_fname] eat anything at that time? If yes: Please tell me everything [month_1_arm_1][en_child_fname] ate at that time. Probe: Anything else? Until respondent says nothing else. Repeat question b) above until respondent says the child went to sleep until the next day. If respondent mentions mixed dishes like a PORRIDGE, sauce or stew, probe: c) What ingredients were in that (MIXEDDISH)? Probe: Anything else? Until respondent says nothing else. As the respondent recalls foods, please indicate the food group associated with the response. If the food is not listed in any of the food groups below, write the food in the box labeled 'other foods'. If foods are used in small amounts for seasoning or as a condiment, include them under the condiments food group.	descriptive
320	cn_ate_food_groups<br><br>Show the field ONLY if:<br>[cn_child_agecalc] >= 5	Select all that apply:	checkbox, Required<br>1 | cn_ate_food_groups___1 | Food Group 1 - Injera, porridge, bread, rice, noodles, or other foods made from grains.<br>2 | cn_ate_food_groups___2 | Food Group 2 - Pumpkin, carrots, squash, or sweet potatoes that are yellow or orange inside.<br>3 | cn_ate_food_groups___3 | Food Group 3 - White potatoes, white yams, manioc, cassava, or any other foods made from roots<br>4 | cn_ate_food_groups___4 | Food Group 4 - Any dark green leafy vegetables<br>5 | cn_ate_food_groups___5 | Food Group 5 - Ripe mangoes, ripe papayas, or (other local vitamin A-rich fruits)<br>6 | cn_ate_food_groups___6 | Food Group 6 - Any other fruits or vegetables<br>7 | cn_ate_food_groups___7 | Food Group 7 - Liver, kidney, heart, or other organ meats<br>8 | cn_ate_food_groups___8 | Food Group 8 - Any meat, such as beef, pork, lamb, goat, chicken, or duck<br>9 | cn_ate_food_groups___9 | Food Group 9 - Eggs<br>10 | cn_ate_food_groups___10 | Food Group 10 - Fresh or dried fish, shellfish, or seafood<br>11 | cn_ate_food_groups___11 | Food Group 11 - Any foods made from beans, peas, or lentils?

			12 | cn_ate_food_groups___12 | Food Group 12 - Any nuts or seeds? Such as Fenugreek, Linseed, and Sesame<br>13 | cn_ate_food_groups___13 | Food Group 13 - Cheese, yogurt, or other milk products<br>14 | cn_ate_food_groups___14 | Food Group 14 - Any oil, fats, or butter, or foods made with any of these<br>15 | cn_ate_food_groups___15 | Food Group 15 - Any sugary foods such as chocolates, sweets, candies, pastries, cakes, or biscuits<br>16 | cn_ate_food_groups___16 | Food Group 16 - Condiments for favor, such as chilies, spices, herbs, or fish powder<br>17 | cn_ate_food_groups___17 | Food Group 17 - Grubs, snails or insects<br>18 | cn_ate_food_groups___18 | Food Group 18 - Foods made with red palm oil, red palm nut, or red palm nut pulp sauce<br>19 | cn_ate_food_groups___19 | Food Group 19 - Breastmilk
321	cn_ate_other_foods<br>Show the field ONLY if: [cn_child_agecalc] >= 5	Other Foods (specify)	notes, Required
322	cn_foods_instruct<br>Show the field ONLY if: [cn_child_agecalc] >= 5	Once the respondent finishes recalling foods eaten and data collector indicates on the list above, read each remaining food group NOT previously selected, and ask the following question:	descriptive
323	cn_no_selection_foods1<br>Show the field ONLY if: [cn_ate_food_groups(1)] = '0' a nd [cn_child_agecalc] >= 5	Yesterday during the day or night, did [month_1_arm_1][en_child_fname] drink/eat any from food group 1?	dropdown, Required<br>1 | Yes<br>0 | No<br>99 | Don't know
324	cn_no_selection_foods2<br>Show the field ONLY if: [cn_ate_food_groups(2)] = '0' a nd [cn_child_agecalc] >= 5	Yesterday during the day or night, did [month_1_arm_1][en_child_fname] drink/eat any from food group 2?	dropdown, Required<br>1 | Yes<br>0 | No<br>99 | Don't know
325	cn_no_selection_foods3<br>Show the field ONLY if: [cn_ate_food_groups(3)] = '0' a nd [cn_child_agecalc] >= 5	Yesterday during the day or night, did [month_1_arm_1][en_child_fname] drink/eat any from food group 3?	dropdown, Required<br>1 | Yes<br>0 | No<br>99 | Don't know
326	cn_no_selection_foods4<br>Show the field ONLY if: [cn_ate_food_groups(4)] = '0' a nd [cn_child_agecalc] >= 5	Yesterday during the day or night, did [month_1_arm_1][en_child_fname] drink/eat any from food group 4?	dropdown, Required<br>1 | Yes<br>0 | No<br>99 | Don't know
327	cn_no_selection_foods5<br>Show the field ONLY if: [cn_ate_food_groups(5)] = '0' a nd [cn_child_agecalc] >= 5	Yesterday during the day or night, did [month_1_arm_1][en_child_fname] drink/eat any from food group 5?	dropdown, Required<br>1 | Yes<br>0 | No<br>99 | Don't know

328	cn_no_selection_foods6<br>Show the field ONLY if:<br>[cn_ate_food_groups(6)] = '0' and [cn_child_agecalc] >= 5	Yesterday during the day or night, did [month_1_arm_1][en_child_fname] drink/eat any from food group 6?	dropdown, Required<br>1 Yes<br>0 No<br>99 Don't know
329	cn_no_selection_foods7<br>Show the field ONLY if:<br>[cn_ate_food_groups(7)] = '0' and [cn_child_agecalc] >= 5	Yesterday during the day or night, did [month_1_arm_1][en_child_fname] drink/eat any from food group 7?	dropdown, Required<br>1 Yes<br>0 No<br>99 Don't know
330	cn_no_selection_foods8<br>Show the field ONLY if:<br>[cn_ate_food_groups(8)] = '0' and [cn_child_agecalc] >= 5	Yesterday during the day or night, did [month_1_arm_1][en_child_fname] drink/eat any from food group 8?	dropdown, Required<br>1 Yes<br>0 No<br>99 Don't know
331	cn_no_selection_foods9<br>Show the field ONLY if:<br>[cn_ate_food_groups(9)] = '0' and [cn_child_agecalc] >= 5	Yesterday during the day or night, did [month_1_arm_1][en_child_fname] drink/eat any from food group 9?	dropdown, Required<br>1 Yes<br>0 No<br>99 Don't know
332	cn_no_selection_foods10<br>Show the field ONLY if:<br>[cn_ate_food_groups(10)] = '0' and [cn_child_agecalc] >= 5	Yesterday during the day or night, did [month_1_arm_1][en_child_fname] drink/eat any from food group 10?	dropdown, Required<br>1 Yes<br>0 No<br>99 Don't know
333	cn_no_selection_foods11<br>Show the field ONLY if:<br>[cn_ate_food_groups(11)] = '0' and [cn_child_agecalc] >= 5	Yesterday during the day or night, did [month_1_arm_1][en_child_fname] drink/eat any from food group 11?	dropdown, Required<br>1 Yes<br>0 No<br>99 Don't know
334	cn_no_selection_foods12<br>Show the field ONLY if:<br>[cn_ate_food_groups(12)] = '0' and [cn_child_agecalc] >= 5	Yesterday during the day or night, did [month_1_arm_1][en_child_fname] drink/eat any from food group 12?	dropdown, Required<br>1 Yes<br>0 No<br>99 Don't know
335	cn_no_selection_foods13<br>Show the field ONLY if:<br>[cn_ate_food_groups(13)] = '0' and [cn_child_agecalc] >= 5	Yesterday during the day or night, did [month_1_arm_1][en_child_fname] drink/eat any from food group 13?	dropdown, Required<br>1 Yes<br>0 No<br>99 Don't know
336	cn_no_selection_foods14<br>Show the field ONLY if:<br>[cn_ate_food_groups(14)] = '0' and [cn_child_agecalc] >= 5	Yesterday during the day or night, did [month_1_arm_1][en_child_fname] drink/eat any from food group 14?	dropdown, Required<br>1 Yes<br>0 No<br>99 Don't know
337	cn_no_selection_foods15<br>Show the field ONLY if:<br>[cn_ate_food_groups(15)] = '0' and [cn_child_agecalc] >= 5	Yesterday during the day or night, did [month_1_arm_1][en_child_fname] drink/eat any from food group 15?	dropdown, Required<br>1 Yes<br>0 No<br>99 Don't know
338	cn_no_selection_foods16<br>Show the field ONLY if:<br>[cn_ate_food_groups(16)] = '0' and [cn_child_agecalc] >= 5	Yesterday during the day or night, did [month_1_arm_1][en_child_fname] drink/eat any from food group 16?	dropdown, Required<br>1 Yes<br>0 No<br>99 Don't know
339	cn_no_selection_foods17<br>Show the field ONLY if:<br>[cn_ate_food_groups(17)] = '0' and [cn_child_agecalc] >= 5	Yesterday during the day or night, did [month_1_arm_1][en_child_fname] drink/eat any from food group 17?	dropdown, Required<br>1 Yes<br>0 No<br>99 Don't know

340	cn_no_selection_foods18<br><br>Show the field ONLY if:<br>[cn_ate_food_groups(18)] = '0' and [cn_child_agecalc] >= 5	Yesterday during the day or night, did [month_1_arm_1] [en_child_fname] drink/eat any from food group 18?	dropdown, Required<br>1 Yes<br>0 No<br>99 Don't know
341	cn_no_selection_foods19<br><br>Show the field ONLY if:<br>[cn_ate_food_groups(19)] = '0' and [cn_child_agecalc] >= 5	Yesterday during the day or night, did [month_1_arm_1] [en_child_fname] drink/eat any from food group 19?	dropdown, Required<br>1 Yes<br>0 No<br>99 Don't know
342	short_survey_complete	Section Header: *Form Status*<br>Complete?	dropdown<br>0 Incomplete<br>1 Unverified<br>2 Complete
Instrument: **Interview Information (All)** (interview_information_all)			Collapse
343	hh_interviewer	Who are you interviewing?<br>*[Note: men and women will be assigned different questions in the survey.*	dropdown<br>1 Father or proxy for father<br>2 Mother
344	interview_information_all_complete	Section Header: *Form Status*<br>Complete?	dropdown<br>0 Incomplete<br>1 Unverified<br>2 Complete
Instrument: **Child's Name** (childs_name)			Collapse
345	child_name	Has this child been named?	yesno<br>1 Yes<br>0 No
346	en_child_fname<br><br>Show the field ONLY if:<br>[child_name] = '1'	What is the child's first name?	text
347	en_child_fname_2<br><br>Show the field ONLY if:<br>[child_name] = '1'	What is the child's middle name?	text
348	en_child_fname_3<br><br>Show the field ONLY if:<br>[child_name] = '1'	What is the child's last name?	text
349	childs_name_complete	Section Header: *Form Status*<br>Complete?	dropdown<br>0 Incomplete<br>1 Unverified<br>2 Complete
Instrument: **General Household Information (Female Only)** (general_household_information_female_only)			Collapse
350	hh_data_collector	Data collector's name:	text<br>Custom alignment: LV
351	hh_date_int	Date of Interview:	text (date_dmy), Required<br>Custom alignment: LV<br>Field Annotation: @HIDEBUTTON
352	hh_father_proxy	If proxy, who?	text

353	hh_mother_verification	Section Header: *Please verify the household ID, the child's name and DOB.*<br>Household ID: [en_hh_id] , Mother's name: [en_mother_fname] [en_mother_mname] [en_mother_lname] , Child's first name: [month_1_arm_1][en_child_fname] [en_child_mname] [en_child_lname] , Child's father's name: [en_father_fname] [en_father_mname] [en_father_lname] , Child's grandfather's name: [en_grandfather_fname] [en_grandfather_mname] [en_grandfather_lname] , Child's sex: [en_child_sex] , Child's DOB: [en_child_dob] , Kebele: [en_kebele_id] ,Village/Ganda ID: [en_village_id] ,Latitude: [en_latitude] , Longitude: [en_longitude] , Altitude: [en_altitude]	radio, Identifier<br>1 Verified<br>0 Unverified
354	hh_mother_script	Section Header: *(Female) Respondent information*<br>(These questions are to be answered by the mother)<br>Explain to the participant: Thank you very much for agreeing to participate in this study and taking the time to answer our questions today. I'd like to start with some basic questions about you.	descriptive
355	hh_mother_fname	Mother's first name [en_mother_fname]	text, Identifier
356	hh_mother_mname	Mother's middle name [en_mother_mname]	text, Identifier
357	hh_mother_lname	Mother's last name [en_mother_lname]	text, Identifier
358	hh_mother_religion	mother's religion	dropdown<br>1 Muslim<br>2 Orthodox Christian<br>3 Protestant Christian<br>4 Catholic Christian<br>5 Traditional believers<br>88 Other (specify)
359	hh_mother_relig_other	Specify other religion:	text
360	hh_mother_educ	Have you ever attended school?	dropdown<br>1 Yes<br>0 No<br>99 Don't know
361	hh_mother_years<br>Show the field ONLY if:<br>[hh_mother_educ] = '1'	What is the highest level of schooling you completed?	dropdown<br>1 No formal education<br>2 Some primary<br>3 Completed primary<br>4 Some secondary<br>5 Completed secondary<br>6 Some university<br>7 Graduated university<br>8 Post graduate/professional training
362	hh_mother_age	Mother's age (years)	text (number)
363	hh_mother_married	How old were you when you got married for the first time?<br>*08-40 (years)*	text (number)
364	hh_mother_birthorder	What number is [month_1_arm_1][en_child_fname] in the birth order of her or his siblings?	dropdown<br>1 First or only child<br>2 Second<br>3 Third<br>4 Fourth<br>5 Fifth<br>6 Sixth<br>7 Seventh<br>8 Eighth<br>9 Ninth<br>10 Tenth or more

365	hh_mother_residents_live	How many individuals live at this residence? (residence to be defined as enumeration household is defined)	text
366	hh_mother_children_live	How many children under five years old live at this residence? (residence to be defined as enumeration household is defined)	text (number)
367	hh_mother_husb_wives	Does your husband have multiple wives?	yesno<br>1 Yes<br>0 No
368	hh_mother_wives_quan<br>Show the field ONLY if:<br>[hh_mother_husb_wives] = '1'	How many?	text (number)
369	hh_mother_wives_number<br>Show the field ONLY if:<br>[hh_mother_husb_wives] = '1'	What number wife are you?	text (number)
370	general_household_information_female_only_complete	Section Header: *Form Status*<br>Complete?	dropdown<br>0 Incomplete<br>1 Unverified<br>2 Complete
Instrument: **General Household Information (Male Only)** (general_household_information_male_only)			
371	hh_f_date_int	Date of Interview:	text (date_dmy), Required<br>Custom alignment: LV<br>Field Annotation: @HIDEBUTTON
372	hh_f_data_collector	Data collector's name:	text<br>Custom alignment: LV
373	hh_f_lat	Latitude	text, Identifier<br>Custom alignment: LV
374	hh_f_long	Longitude	text, Identifier<br>Custom alignment: LV
375	hh_f_father_proxy	If proxy, who?	text
376	hh_father_verification	Section Header: *Please verify the household ID, the child's name and DOB.*<br>Household ID: [en_hh_id] , Mother's name: [en_mother_fname] [en_mother_mname] [en_mother_lname] , Child's first name: [month_1_arm_1][en_child_fname] [en_child_mname] [en_child_lname] , Child's father's name: [en_father_fname] [en_father_mname] [en_father_lname] , Child's grandfather's name: [en_grandfather_fname] [en_grandfather_mname] [en_grandfather_lname] , Child's sex: [en_child_sex] , Child's DOB: [en_child_dob] , Kebele: [en_kebele_id] ,Village/Ganda ID: [en_village_id] ,Latitude: [en_latitude] , Longitude: [en_longitude] , Altitude: [en_altitude]	radio, Identifier<br>1 Verified<br>0 Unverified
377	hh_father_script	Section Header: *(Male) Respondent information*<br>(These questions are to be answered by father or proxy)<br>Explain to the participant: Thank you very much for agreeing to participate in this study and taking the time to answer our questions today. I'd like to start with some basic questions about you.	descriptive
378	hh_father_fname	Father's first name [en_father_fname]	text, Identifier
379	hh_father_mname	Father's middle name [en_father_mname]	text, Identifier
380	hh_father_lname	Father's last name [en_father_lname]	text, Identifier
381	hh_father_religion	Father's religion	dropdown<br>1 Muslim<br>2 Orthodox Christian<br>3 Protestant Christian<br>4 Catholic Christian<br>5 Traditional believers<br>88 Other (specify)

382	hh_father_relig_other<br><br>Show the field ONLY if:<br>[hh_father_religion] = '88'	Specify other religion:	text
383	hh_father_age	Father's age (years)	text
384	hh_father_educ	Have you ever attended school?	dropdown<br>1 Yes<br>0 No<br>99 Don't know
385	hh_father_years<br><br>Show the field ONLY if:<br>[hh_father_educ] = '1'	What is the highest level of schooling you completed?	dropdown<br>1 No formal education<br>2 Some primary<br>3 Completed primary<br>4 Some secondary<br>5 Completed secondary<br>6 Some university<br>7 Graduated university<br>8 Post graduate/professional training
386	general_household_information_male_only_complete	Section Header: *Form Status*<br>Complete?	dropdown<br>0 Incomplete<br>1 Unverified<br>2 Complete
Instrument: **Livelihoods (Male Only)** (livelihoods_male_only)			Collapse
387	ll_father_script	Section Header: *(Male) Livelihoods*<br>Explain to the participant: Now I'm going to ask you some questions relating to your household's livelihood practices, or those things you do to make a living.	descriptive
388	ll_livelihood	Section Header: *Major Cropping Seasons*<br>LL1) In which of the following livelihood activities does your household engage? (Multiple choices allowed)<br>*Check all that apply*	checkbox<br>1 ll_livelihood___1 Animal Production<br>2 ll_livelihood___2 Crop Production (Annual Crops)<br>3 ll_livelihood___3 Chat Production<br>4 ll_livelihood___4 Petty Trade (shops, small trade)<br>5 ll_livelihood___5 Remittances<br>88 ll_livelihood___88 Other (specify below)<br><br>Custom alignment: LV
389	ll_livelihood_other<br><br>Show the field ONLY if:<br>[ll_livelihood(88)] = '1'	LL1a) Specify Other:	text<br>Custom alignment: LV
390	ll_livelihood_primary	LL2) Which would you say is your primary source of livelihood/income (choose one)?	radio<br>1 Animal Production<br>2 Crop Production (Annual Crops)<br>3 Chat Production<br>4 Petty Trade (shops, small trade)<br>5 Remittances<br>88 Other (specify below)<br><br>Custom alignment: LV
391	ll_live_primary_other<br><br>Show the field ONLY if:<br>[ll_livelihood_primary] = '88'	LL2a) Specify Other:	text<br>Custom alignment: LV

Havelaar AH, *et al. BMJ Open* 2022; 12:e061311. doi: 10.1136/bmjopen-2022-061311

392	ll_produce_crop	Do you produce crop?	yesno<br>1 Yes<br>0 No
393	ll_produce<br>Show the field ONLY if:<br>[ll_produce_crop] = '1'	LL3) Which crops do you produce? (Multiple choices allowed)<br>*Check all that apply*	checkbox<br>1 ll_produce___1 Chat<br>2 ll_produce___2 Sorghum<br>3 ll_produce___3 Maize<br>4 ll_produce___4 Teff<br>5 ll_produce___5 Vegetables<br>6 ll_produce___6 Beans<br>88 ll_produce___88 Other (specify below)<br>Custom alignment: LV
394	ll_produce_other<br>Show the field ONLY if:<br>[ll_produce(88)] = '1'	LL3a) Specify Other:	text<br>Custom alignment: LV
395	ll_irrigation	Section Header: *Off-Season (Using Irrigation)*<br>LL4) Do you use irrigation?	yesno<br>1 Yes<br>0 No<br>Custom alignment: LV
396	ll_irrigation_prod<br>Show the field ONLY if:<br>[ll_irrigation] = '1'	LL4a) In the off-season, which crop(s) do you produce using irrigation? (Multiple choices allowed)<br>*Check all that apply*	checkbox<br>1 ll_irrigation_prod___1 Chat<br>2 ll_irrigation_prod___2 Vegetables<br>88 ll_irrigation_prod___88 Other (specify below)<br>Custom alignment: LV
397	ll_irrigation_prod_other<br>Show the field ONLY if:<br>[ll_irrigation_prod(88)] = '1'	LL4b) Specify other:	text<br>Custom alignment: LV
398	livelihoods_male_only_complete	Section Header: *Form Status*<br>Complete?	dropdown<br>0 Incomplete<br>1 Unverified<br>2 Complete

Instrument: **Assets And Wealth (Male Only)** (assets_and_wealth_male_only) Collapse

399	aw_ownership_items	Section Header: *Assets*<br>Now I would like to ask you about your household ownership of a number of items that could be used to generate income. Please indicate if you currently have the following assets:	checkbox<br>1 aw_ownership_items___1 Stores/granary<br>2 aw_ownership_items___2 Poultry houses<br>3 aw_ownership_items___3 Bore hole/well<br>4 aw_ownership_items___4 Motor cycle<br>5 aw_ownership_items___5 Car<br>6 aw_ownership_items___6 Bicycle<br>7 aw_ownership_items___7 Truck/lorry<br>8 aw_ownership_items___8 Tractor<br>9 aw_ownership_items___9 Wheel burrow<br>10 aw_ownership_items___10 Water pump<br>11 aw_ownership_items___11 Hoe<br>12 aw_ownership_items___12 Machete/'mencha'<br>13 aw_ownership_items___13 Crop sheller<br>14 aw_ownership_items___14 Chemical sprayer<br>15 aw_ownership_items___15 Weighing scale<br>16 aw_ownership_items___16 Water tanks<br>17 aw_ownership_items___17 TV<br>18 aw_ownership_items___18 Telephone<br>19 aw_ownership_items___19 Household structures owned<br>20 aw_ownership_items___20 Money in a bank<br>88 aw_ownership_items___88 Other receivables<br>Custom alignment: LV
400	aw_stores_granary_no<br>Show the field ONLY if:<br>[aw_ownership_items(1)] = '1'	AW1a) Indicate the quantity/number of stores/granary owned	text (integer)<br>Custom alignment: LV
401	aw_stores_granary_value<br>Show the field ONLY if:<br>[aw_ownership_items(1)] = '1'	AW1b) Estimate of current value of stores/granary owned (Birr)<br>NOTE (This estimated value is TOTAL estimated value of all stores/granaries. This applies to all future questions).	text (integer)<br>Custom alignment: LV
402	aw_poultry_no<br>Show the field ONLY if:<br>[aw_ownership_items(2)] = '1'	AW2a) Indicate the quantity/number of poultry houses owned	text (integer)<br>Custom alignment: LV
403	aw_poultry_value<br>Show the field ONLY if:<br>[aw_ownership_items(2)] = '1'	AW2b) Estimate of current value of poultry houses owned (Birr)	text (integer)<br>Custom alignment: LV
404	aw_well_no<br>Show the field ONLY if:<br>[aw_ownership_items(3)] = '1'	AW3a) Indicate the quantity/number of bore hole/well owned	text (integer)<br>Custom alignment: LV
405	aw_well_value<br>Show the field ONLY if:<br>[aw_ownership_items(3)] = '1'	AW3b) Estimate of current value of bore hole/well owned (Birr)	text (integer)<br>Custom alignment: LV
406	aw_cycles_no<br>Show the field ONLY if:<br>[aw_ownership_items(4)] = '1'	AW4a) Indicate the quantity/number of motor cycles owned	text (integer)<br>Custom alignment: LV
407	aw_cycles_value<br>Show the field ONLY if:<br>[aw_ownership_items(4)] = '1'	AW4b) Estimate of current value of motor cycles owned (Birr)	text (number)<br>Custom alignment: LV
408	aw_cars_no<br>Show the field ONLY if:<br>[aw_ownership_items(5)] = '1'	AW5a) Indicate the quantity/number of cars owned	text (integer)<br>Custom alignment: LV

409	aw_cars_value<br><br>Show the field ONLY if:<br>[aw_ownership_items(5)] = '1'	AW5b) Estimate of current value of cars owned (Birr)	text (number)<br>Custom alignment: LV
410	aw_bikes_no<br><br>Show the field ONLY if:<br>[aw_ownership_items(6)] = '1'	AW6a) Indicate the quantity/number of bicycles owned	text (integer)<br>Custom alignment: LV
411	aw_bikes_value<br><br>Show the field ONLY if:<br>[aw_ownership_items(6)] = '1'	AW6b) Estimate of current value of bicycles owned (Birr)	text (integer)<br>Custom alignment: LV
412	aw_trucks_no<br><br>Show the field ONLY if:<br>[aw_ownership_items(7)] = '1'	AW7a) Indicate the quantity/number of trucks/lorries owned	text (integer)<br>Custom alignment: LV
413	aw_trucks_value<br><br>Show the field ONLY if:<br>[aw_ownership_items(7)] = '1'	AW7b) Estimate of current value of trucks/lorries owned (Birr)	text (integer)<br>Custom alignment: LV
414	aw_tractor_no<br><br>Show the field ONLY if:<br>[aw_ownership_items(8)] = '1'	AW8a) Indicate the quantity/number of tractors owned	text (integer)<br>Custom alignment: LV
415	aw_tractor_value<br><br>Show the field ONLY if:<br>[aw_ownership_items(8)] = '1'	AW8b) Estimate of current value of tractors owned (Birr)	text (integer)<br>Custom alignment: LV
416	aw_barrow_no<br><br>Show the field ONLY if:<br>[aw_ownership_items(9)] = '1'	AW9a) Indicate the quantity/number of wheelbarrows owned	text (integer)<br>Custom alignment: LV
417	aw_barrow_value<br><br>Show the field ONLY if:<br>[aw_ownership_items(9)] = '1'	AW9b) Estimate of current value of wheelbarrows owned (Birr)	text (integer)<br>Custom alignment: LV
418	aw_water_pump_no<br><br>Show the field ONLY if:<br>[aw_ownership_items(10)] = '1'	AW10a) Indicate the quantity/number of water pumps owned	text (integer)<br>Custom alignment: LV
419	aw_pumps_value<br><br>Show the field ONLY if:<br>[aw_ownership_items(10)] = '1'	AW10b) Estimate of current value of water pumps owned (Birr)	text (number)<br>Custom alignment: LV
420	aw_hoes_no<br><br>Show the field ONLY if:<br>[aw_ownership_items(11)] = '1'	AW11a) Indicate the quantity/number of hoes owned	text (integer)<br>Custom alignment: LV
421	aw_hoes_value<br><br>Show the field ONLY if:<br>[aw_ownership_items(11)] = '1'	AW11b) Estimate of current value of hoes owned (Birr)	text (number)<br>Custom alignment: LV
422	aw_mencha_no<br><br>Show the field ONLY if:<br>[aw_ownership_items(12)] = '1'	AW12a) Indicate the quantity/number of machetes/'mencha' owned	text (integer)<br>Custom alignment: LV
423	aw_mencha_value<br><br>Show the field ONLY if:<br>[aw_ownership_items(12)] = '1'	AW12b) Estimate of current value of machetes/'mencha' owned (Birr)	text (integer)<br>Custom alignment: LV
424	aw_shellers_no<br><br>Show the field ONLY if:<br>[aw_ownership_items(13)] = '1'	AW13a) Indicate the quantity/number of crop shellers owned	text (integer)<br>Custom alignment: LV
425	aw_shellers_value<br><br>Show the field ONLY if:<br>[aw_ownership_items(13)] = '1'	AW13b) Estimate of current value of crop shellers owned (Birr)	text (number)<br>Custom alignment: LV
426	aw_sprayers_no<br><br>Show the field ONLY if:<br>[aw_ownership_items(14)] = '1'	AW14a) Indicate the quantity/number of chemical sprayers owned	text (integer)<br>Custom alignment: LV

427	aw_sprayers_value<br>Show the field ONLY if:<br>[aw_ownership_items(14)] = '1'	AW14b) Estimate of current value of chemical sprayers owned (Birr)	text (number)<br>Custom alignment: LV
428	aw_scales_no<br>Show the field ONLY if:<br>[aw_ownership_items(15)] = '1'	AW15a) Indicate the quantity/number of weighing scales owned	text (integer)<br>Custom alignment: LV
429	aw_scales_value<br>Show the field ONLY if:<br>[aw_ownership_items(15)] = '1'	AW15b) Estimate of current value of weighing scales owned (Birr)	text (integer)<br>Custom alignment: LV
430	aw_tanks_no<br>Show the field ONLY if:<br>[aw_ownership_items(16)] = '1'	AW16a) Indicate the quantity/number of water tanks owned	text (integer)<br>Custom alignment: LV
431	aw_tanks_value<br>Show the field ONLY if:<br>[aw_ownership_items(16)] = '1'	AW16b) Estimate of current value of water tanks owned (Birr)	text (number)<br>Custom alignment: LV
432	aw_tv_no<br>Show the field ONLY if:<br>[aw_ownership_items(17)] = '1'	AW17a) Indicate the quantity/number of TVs owned	text (integer)<br>Custom alignment: LV
433	aw_tv_value<br>Show the field ONLY if:<br>[aw_ownership_items(17)] = '1'	AW17b) Estimate of current value of TVs owned (Birr)	text (integer)<br>Custom alignment: LV
434	aw_phone_no<br>Show the field ONLY if:<br>[aw_ownership_items(18)] = '1'	AW18a) Indicate the quantity/number of telephones/mobiles owned	text (integer)<br>Custom alignment: LV
435	aw_phone_value<br>Show the field ONLY if:<br>[aw_ownership_items(18)] = '1'	AW18b) Estimate of current value of telephones/mobiles owned (Birr)	text (number)<br>Custom alignment: LV
436	aw_house_no<br>Show the field ONLY if:<br>[aw_ownership_items(19)] = '1'	AW19a) Indicate the quantity/number of total household structures owned.	text (integer)<br>Custom alignment: LV
437	aw_house_value<br>Show the field ONLY if:<br>[aw_ownership_items(19)] = '1'	AW19b) Estimate of current value of household structure owned.	text (integer)<br>Custom alignment: LV
438	aw_money_value<br>Show the field ONLY if:<br>[aw_ownership_items(20)] = '1'	AW20a) Estimate of current value of money in bank (birr)	text<br>Custom alignment: LV
439	aw_receivables<br>Show the field ONLY if:<br>[aw_ownership_items(88)] = '1'	Other receivables? How many owned	text<br>Custom alignment: LV
440	aw_receivables_value<br>Show the field ONLY if:<br>[aw_ownership_items(88)] = '1'	Other receivables value	text<br>Custom alignment: LV
441	aw_liabilities	AW21) Any liabilities (e.g. Borrowed from relatives or bank)?	yesno<br>1 Yes<br>0 No<br>Custom alignment: LV
442	aw_liabilities_yes<br>Show the field ONLY if:<br>[aw_liabilities] = '1'	Estimate the current value of all liabilities owed	text<br>Custom alignment: LV
443	aw_total_value	What do you estimate is the of current value of all the items that you own, in Birr?<br>*This estimated value is TOTAL estimated value of all items.*	text (number)
444	aw_cattle_birr	Section Header: *ANNUAL INCOME*<br>AW22) Sale of cattle - Annual income obtained (Birr)	text (number)<br>Custom alignment: LV

445	aw_goats_birr	AW23) Sale of goats and sheep - Annual income obtained (Birr)	text (number)<br>Custom alignment: LV
446	aw_chicken_birr	AW24) Sale of chicken and eggs - Annual income obtained (Birr)	text (number)<br>Custom alignment: LV
447	aw_products_birr	AW25) Sale of other livestock products (milk, yoghurt, butter, cheese, ...) - Annual income obtained (Birr)	text (number)<br>Custom alignment: LV
448	aw_crops_birr	AW26) Sale of annual crops - Annual income obtained (Birr)	text (number)<br>Custom alignment: LV
449	aw_chat_birr	AW27) Sale of chat - Annual income obtained (Birr)	text (number)<br>Custom alignment: LV
450	aw_activities_birr	AW28) Income from off-farm/non-farm activities (petty trade, remittances, handicrafts, casual labor,...) - Annual income obtained (Birr)	text (number)<br>Custom alignment: LV
451	aw_other_sources_type1	Section Header: *Income from other sources*<br>AW29) Income from other sources - specify source type:	text<br>Custom alignment: LV
452	aw_other_source_birr	AW29a) Annual income obtained (Birr) - from other source 1	text (number)<br>Custom alignment: LV
453	aw_other_sources_type2	AW30) Income from other sources - specify source type:	text<br>Custom alignment: LV
454	aw_other_source2_birr	AW30a) Annual income obtained (Birr) - from other source 2	text (number)<br>Custom alignment: LV
455	aw_other_sources_type3	AW31) Income from other sources - specify source type:	text<br>Custom alignment: LV
456	aw_other_source3_birr	AW31a) Annual income obtained (Birr) - from other source 3	text (number)<br>Custom alignment: LV
457	aw_floor_materials	Section Header: *Please observe the following information about the respondent's household:*<br>Main material of the floor (observation)	dropdown<br>1 Earth/sand/clay/mud/dung<br>2 Wood planks<br>3 Parquet or polished wood<br>4 Vinyl or asphalt strips<br>5 Ceramic tiles<br>6 Cement/concrete<br>88 Other
458	aw_roof_material	Main material of the roof (observation)	dropdown<br>1 No roof<br>2 Thatch/palm leaf/reed/grass<br>3 Sod/mud and grass mixture<br>4 Plastic/polythene sheeting<br>5 Rustic mat<br>6 Palm/bamboo<br>7 Unburnt brick<br>8 Loosely packed stone<br>9 Metal/GI<br>10 Wood<br>11 Calamine/cement fiber<br>12 Asbestos sheets<br>13 RCC/RBC/Cement/Concrete<br>14 Roofing shingles<br>15 Tiles<br>16 Slate<br>17 Burnt brick<br>88 Other

459	aw_walls_material	Main material of the exterior walls (observation)	dropdown<br>1 No walls<br>2 Cane/palm/trunks/bamboo<br>3 Mud/sand<br>4 Bamboo with mud<br>5 Stone with mud<br>6 Plywood<br>7 Cardboard<br>8 Raw wood/reused wood<br>9 Cement/concrete<br>10 Stone with lime/cement<br>11 Bricks<br>12 Wood planks/shingles<br>13 Metal/Asbestos sheets<br>88 Other
460	assets_and_wealth_male_only_complete	Section Header: *Form Status*<br>Complete?	dropdown<br>0 Incomplete<br>1 Unverified<br>2 Complete
Instrument: **Animal Husbandry (Male Only)** (animal_husbandry_male_only) Collapse			
461	ao_animal_ownership	Section Header: *Animal ownership*<br>AO1) I am now going to ask you questions relating to your household's animals.<br><br>Does your family currently have any of the following animals:	checkbox<br>1 ao_animal_ownership___1 Cattle<br>2 ao_animal_ownership___2 Donkey<br>3 ao_animal_ownership___3 Goat<br>4 ao_animal_ownership___4 Sheep<br>5 ao_animal_ownership___5 Chicken<br>6 ao_animal_ownership___6 Dog<br>7 ao_animal_ownership___7 Cat<br>8 ao_animal_ownership___8 Camel<br>88 ao_animal_ownership___88 Other domestic animals<br><br>Custom alignment: LV
462	ao_cattle_number<br><br>Show the field ONLY if:<br>[ao_animal_ownership(1)] = '1'	AO2b) How many cattle does your household have?	text (number, Min: 1)<br>Custom alignment: LV
463	ao_cattle_purpose<br><br>Show the field ONLY if:<br>[ao_animal_ownership(1)] = '1'	AO2b) For what purposes do you keep these cattle ? (check all that apply)	checkbox<br>1 ao_cattle_purpose___1 Income (meat)<br>2 ao_cattle_purpose___2 Income (byproduct)<br>3 ao_cattle_purpose___3 Income (livestock)<br>4 ao_cattle_purpose___4 Trade (meat)<br>5 ao_cattle_purpose___5 Trade (byproduct)<br>6 ao_cattle_purpose___6 Trade (livestock)<br>7 ao_cattle_purpose___7 Consumption (meat)<br>8 ao_cattle_purpose___8 Consumption (byproduct)<br>9 ao_cattle_purpose___9 Savings<br>10 ao_cattle_purpose___10 Traction/Transport<br>88 ao_cattle_purpose___88 Others (Specify below)<br><br>Custom alignment: LV

464	ao_cattle_purpose_main<br>Show the field ONLY if:<br>[ao_animal_ownership(1)] = '1'	AO2d) Which of these is the main purpose for which you keep these cattle? (check only one)	radio<br>1 Income (meat)<br>2 Income (byproduct)<br>3 Income (livestock)<br>4 Trade (meat)<br>5 Trade (byproduct)<br>6 Trade (livestock)<br>7 Consumption (meat)<br>8 Consumption (byproduct)<br>9 Savings<br>10 Traction/Transport<br>88 Others (Specify below)<br>Custom alignment: LV
465	ao_cattle_purpose_other<br>Show the field ONLY if:<br>[ao_cattle_purpose(88)] = '1'	AO2c) Specify Other purpose(s):	text<br>Custom alignment: LV
466	ao_cattle_purpose_main_other<br>Show the field ONLY if:<br>[ao_cattle_purpose(88)] = '1'	AO2e) Specify Other main purpose:	text<br>Custom alignment: LV
467	ao_cattle_day<br>Show the field ONLY if:<br>[ao_animal_ownership(1)] = '1'	AO2f) Where are they kept during the day?<br>*Check all that apply*	checkbox<br>1 ao_cattle_day___1 Inside house<br>2 ao_cattle_day___2 Inside homestead (outside house)<br>3 ao_cattle_day___3 Outside homestead<br>Custom alignment: LV
468	ao_cattle_day_in_confine<br>Show the field ONLY if:<br>[ao_cattle_day(1)] = '1'	AO2g) When those cattle are inside the house, are they confined while inside the house?	yesno<br>1 Yes<br>0 No<br>Custom alignment: LV
469	ao_cattle_day_in_how<br>Show the field ONLY if:<br>[ao_cattle_day_in_confine] = '1'	AO2h) How are they confined inside the house?	radio<br>1 Tied<br>2 Confined inside a structure/room<br>3 Cooped<br>4 Corralled<br>88 Other (Specify below)<br>Custom alignment: LV
470	ao_cattle_day_in_confine_other<br>Show the field ONLY if:<br>[ao_cattle_day_in_how] = '88'	AO2i) Specify Other type of confinement:	text<br>Custom alignment: LV
471	ao_cattle_homestead<br>Show the field ONLY if:<br>[ao_cattle_day(2)] = '1'	AO2k) When those cattle are inside the homestead, but not in the house, are the animals confined?	yesno<br>1 Yes<br>0 No<br>Custom alignment: LV

472	ao_cattle_homestead_conf<br>Show the field ONLY if:<br>[ao_cattle_homestead] = '1'	How are they confined inside the homestead?	radio<br>1 Tied<br>2 Confined inside a structure/room<br>3 Cooped<br>4 Corralled<br>88 Other (specify below)<br>Custom alignment: LV
473	ao_cattle_homestead_conf_o<br>Show the field ONLY if:<br>[ao_cattle_homestead_conf] = '88'	Specify other type of confinement	text<br>Custom alignment: LV
474	ao_cattle_out_conf<br>Show the field ONLY if:<br>[ao_cattle_day(3)] = '1'	Are the cattle confined when they are outside homestead?	yesno<br>1 Yes<br>0 No<br>Custom alignment: LV
475	ao_cattle_day_out_how<br>Show the field ONLY if:<br>[ao_cattle_out_conf] = '1'	AO2n) How are they confined outside the homestead?	radio<br>1 Tied<br>2 Confined<br>3 Cooped<br>4 Corralled<br>88 Other (Specify below)<br>Custom alignment: LV
476	ao_cattle_day_out_other<br>Show the field ONLY if:<br>[ao_cattle_day_out_how] = '88'	AO2o) Specify Other confinement:	text<br>Custom alignment: LV
477	ao_cattle_night<br>Show the field ONLY if:<br>[ao_animal_ownership(1)] = '1'	AO2p) Where are they kept/sleep during the night?<br>*Check all that apply*	checkbox<br>1 ao_cattle_night___1 Inside house<br>2 ao_cattle_night___2 Inside homestead (outside house)<br>3 ao_cattle_night___3 Outside homestead<br>Custom alignment: LV
478	ao_cattle_night_home_conf<br>Show the field ONLY if:<br>[ao_cattle_night(1)] = '1'	AO2r) When those cattle are inside the house at night, are they confined while inside the house?	yesno<br>1 Yes<br>0 No<br>Custom alignment: LV
479	ao_cattle_night_in_how<br>Show the field ONLY if:<br>[ao_cattle_night_home_conf] = '1'	AO2r) How are they confined inside house?	radio<br>1 Tied<br>2 Confined inside a structure/room<br>3 Cooped<br>4 Corralled<br>88 Other (Specify below)<br>Custom alignment: LV
480	ao_cattle_night_in_oth<br>Show the field ONLY if:<br>[ao_cattle_night_in_how] = '88'	AO2t) Specify Other confinement:	text<br>Custom alignment: LV
481	ao_cattle_homestead_nt_con<br>Show the field ONLY if:<br>[ao_cattle_night(2)] = '1'	AO2u) When those cattle are inside the homestead but outside the house at night, are the animals confined?	yesno<br>1 Yes<br>0 No<br>Custom alignment: LV

482	ao_cattle_homestead_nt_con_how<br>Show the field ONLY if:<br>[ao_cattle_homestead_nt_con] = '1'	How are they confined inside homestead?	radio<br>1 Tied<br>2 Confined inside a structure/room<br>3 Cooped<br>4 Corralled<br>88 Other (Specify below)<br>Custom alignment: LV
483	ao_cattle_homestead_nt_con_o<br>Show the field ONLY if:<br>[ao_cattle_homestead_nt_con_how] = '88'	AO2w) Specify Other confinement:	text<br>Custom alignment: LV
484	ao_cattle_out_nt_conf<br>Show the field ONLY if:<br>[ao_cattle_night(3)] = '1'	AO2x) Are the cattle confined when they are outside homestead?	yesno<br>1 Yes<br>0 No<br>Custom alignment: LV
485	ao_cattle_out_nt_conf_how<br>Show the field ONLY if:<br>[ao_cattle_out_nt_conf] ='1'	AO2y) How are they confined outside homestead?	radio<br>1 Tied<br>2 Confined inside a structure/room<br>3 Cooped<br>4 Corralled<br>88 Other (Specify below)<br>Custom alignment: LV
486	ao_cattle_night_out_oth<br>Show the field ONLY if:<br>[ao_cattle_out_nt_conf] = '88'	AO2z) Specify Other confinement:	text<br>Custom alignment: LV
487	ao_donkey_number<br>Show the field ONLY if:<br>[ao_animal_ownership(2)] = '1'	AO2b) How many donkey does your household have?	text (number, Min: 1)<br>Custom alignment: LV
488	ao_donkey_purpose<br>Show the field ONLY if:<br>[ao_animal_ownership(2)] = '1'	AO2b) For what purposes do you keep these donkey ? (check all that apply)	checkbox<br>1 ao_donkey_purpose___1 Income (meat)<br>2 ao_donkey_purpose___2 Income (byproduct)<br>3 ao_donkey_purpose___3 Income (livestock)<br>4 ao_donkey_purpose___4 Trade (meat)<br>5 ao_donkey_purpose___5 Trade (byproduct)<br>6 ao_donkey_purpose___6 Trade (livestock)<br>7 ao_donkey_purpose___7 Consumption (meat)<br>8 ao_donkey_purpose___8 Consumption (byproduct)<br>9 ao_donkey_purpose___9 Savings<br>10 ao_donkey_purpose___10 Traction/Transport<br>88 ao_donkey_purpose___88 Others (Specify below)<br>Custom alignment: LV
489	ao_donkey_purpose_other<br>Show the field ONLY if:<br>[ao_donkey_purpose(88)] = '1'	AO2c) Specify Other purpose(s):	text<br>Custom alignment: LV

490	ao_donkey_purpose_main<br>Show the field ONLY if:<br>[ao_animal_ownership(2)] = '1'	AO2d) Which of these is the main purpose for which you keep these donkey? (check only one)	radio<br>1 Income (meat)<br>2 Income (byproduct)<br>3 Income (livestock)<br>4 Trade (meat)<br>5 Trade (byproduct)<br>6 Trade (livestock)<br>7 Consumption (meat)<br>8 Consumption (byproduct)<br>9 Savings<br>10 Traction/Transport<br>88 Others (Specify below)<br>Custom alignment: LV
491	ao_donkey_purpose_main_other<br>Show the field ONLY if:<br>[ao_donkey_purpose(88)] = '1'	AO2e) Specify Other main purpose:	text<br>Custom alignment: LV
492	ao_donkey_day<br>Show the field ONLY if:<br>[ao_animal_ownership(2)] = '1'	AO2f) Where are they kept during the day?<br>*Check all that apply*	checkbox<br>1 ao_donkey_day___1 Inside house<br>2 ao_donkey_day___2 Inside homestead (outside house)<br>3 ao_donkey_day___3 Outside homestead<br>Custom alignment: LV
493	ao_donkey_day_in_confine<br>Show the field ONLY if:<br>[ao_donkey_day(1)] = '1'	AO2g) When those donkey are inside the house, are they confined while inside the house?	yesno<br>1 Yes<br>0 No<br>Custom alignment: LV
494	ao_donkey_day_in_how<br>Show the field ONLY if:<br>[ao_donkey_day_in_confine] = '1'	AO2h) How are they confined inside the house?	radio<br>1 Tied<br>2 Confined inside a structure/room<br>3 Cooped<br>4 Corralled<br>88 Other (Specify below)<br>Custom alignment: LV
495	ao_donkey_day_in_confine_other<br>Show the field ONLY if:<br>[ao_donkey_day_in_how] = '88'	AO2i) Specify Other type of confinement:	text<br>Custom alignment: LV
496	ao_donkey_homestead<br>Show the field ONLY if:<br>[ao_donkey_day(2)] = '1'	AO2k) When those donkey are inside the homestead, but not in the house, are the animals confined?	yesno<br>1 Yes<br>0 No<br>Custom alignment: LV
497	ao_donkey_homestead_conf<br>Show the field ONLY if:<br>[ao_donkey_homestead] = '1'	How are they confined inside the homestead?	radio<br>1 Tied<br>2 Confined inside a structure/room<br>3 Cooped<br>4 Corralled<br>88 Other (specify below)<br>Custom alignment: LV

498	ao_donkey_homestead_conf_o<br>Show the field ONLY if: [ao_donkey_homestead_conf] = '88'	Specify other type of confinement	text<br>Custom alignment: LV
499	ao_donkey_out_conf<br>Show the field ONLY if: [ao_donkey_day(3)] = '1'	Are the donkey confined when they are outside homestead?	yesno<br>1 Yes<br>0 No<br>Custom alignment: LV
500	ao_donkey_day_out_how<br>Show the field ONLY if: [ao_donkey_out_conf] = '1'	AO2n) How are they confined outside the homestead?	radio<br>1 Tied<br>2 Confined<br>3 Cooped<br>4 Corralled<br>88 Other (Specify below)<br>Custom alignment: LV
501	ao_donkey_day_out_other<br>Show the field ONLY if: [ao_donkey_day_out_how] = '88'	AO2o) Specify Other confinement:	text<br>Custom alignment: LV
502	ao_donkey_night<br>Show the field ONLY if: [ao_animal_ownership(2)] = '1'	AO2p) Where are they kept/sleep during the night?<br>*Check all that apply*	checkbox<br>1 ao_donkey_night___1 Inside house<br>2 ao_donkey_night___2 Inside homestead (outside house)<br>3 ao_donkey_night___3 Outside homestead<br>Custom alignment: LV
503	ao_donkey_night_home_conf<br>Show the field ONLY if: [ao_donkey_night(1)] = '1'	AO2r) When those donkey are inside the house at night, are they confined while inside the house?	yesno<br>1 Yes<br>0 No<br>Custom alignment: LV
504	ao_donkey_night_in_how<br>Show the field ONLY if: [ao_donkey_night_home_conf] = '1'	AO2r) How are they confined inside house?	radio<br>1 Tied<br>2 Confined inside a structure/room<br>3 Cooped<br>4 Corralled<br>88 Other (Specify below)<br>Custom alignment: LV
505	ao_donkey_night_in_oth<br>Show the field ONLY if: [ao_donkey_night_in_how] = '88'	AO2t) Specify Other confinement:	text<br>Custom alignment: LV
506	ao_donkey_homestead_nt_con<br>Show the field ONLY if: [ao_donkey_night(2)] = '1'	AO2u) When those donkey are inside the homestead but outside the house at night, are the animals confined?	yesno<br>1 Yes<br>0 No<br>Custom alignment: LV

507	ao_donkey_homestead_nt_con_how<br>Show the field ONLY if: [ao_donkey_homestead_nt_con] = '1'	How are they confined inside homestead?	radio<br>1 Tied<br>2 Confined inside a structure/room<br>3 Cooped<br>4 Corralled<br>88 Other (Specify below)<br>Custom alignment: LV
508	ao_donkey_homestead_nt_con_o<br>Show the field ONLY if: [ao_donkey_homestead_nt_con_how] = '88'	AO2w) Specify Other confinement:	text<br>Custom alignment: LV
509	ao_donkey_out_nt_conf<br>Show the field ONLY if: [ao_donkey_night(3)] = '1'	AO2x) Are the donkey confined when they are outside homestead?	yesno<br>1 Yes<br>0 No<br>Custom alignment: LV
510	ao_donkey_out_nt_conf_how<br>Show the field ONLY if: [ao_donkey_out_nt_conf] ='1'	AO2y) How are they confined outside homestead?	radio<br>1 Tied<br>2 Confined inside a structure/room<br>3 Cooped<br>4 Corralled<br>88 Other (Specify below)<br>Custom alignment: LV
511	ao_donkey_night_out_oth<br>Show the field ONLY if: [ao_donkey_out_nt_conf] = '88'	AO2z) Specify Other confinement:	text<br>Custom alignment: LV
512	ao_goat_number<br>Show the field ONLY if: [ao_animal_ownership(3)] = '1'	AO2b) How many goat does your household have?	text (number, Min: 1)<br>Custom alignment: LV
513	ao_goat_purpose<br>Show the field ONLY if: [ao_animal_ownership(3)] = '1'	AO2b) For what purposes do you keep these goat ? (check all that apply)	checkbox<br>1 ao_goat_purpose___1 Income (meat)<br>2 ao_goat_purpose___2 Income (byproduct)<br>3 ao_goat_purpose___3 Income (livestock)<br>4 ao_goat_purpose___4 Trade (meat)<br>5 ao_goat_purpose___5 Trade (byproduct)<br>6 ao_goat_purpose___6 Trade (livestock)<br>7 ao_goat_purpose___7 Consumption (meat)<br>8 ao_goat_purpose___8 Consumption (byproduct)<br>9 ao_goat_purpose___9 Savings<br>10 ao_goat_purpose___10 Traction/Transport<br>88 ao_goat_purpose___88 Others (Specify below)<br>Custom alignment: LV
514	ao_goat_purpose_other<br>Show the field ONLY if: [ao_goat_purpose(88)] = '1'	AO2c) Specify Other purpose(s):	text<br>Custom alignment: LV

Havelaar AH, *et al. BMJ Open* 2022; 12:e061311. doi: 10.1136/bmjopen-2022-061311

515	ao_goat_purpose_main<br>Show the field ONLY if:<br>[ao_animal_ownership(3)] = '1'	AO2d) Which of these is the main purpose for which you keep these goat? (check only one)	radio<br>1 Income (meat)<br>2 Income (byproduct)<br>3 Income (livestock)<br>4 Trade (meat)<br>5 Trade (byproduct)<br>6 Trade (livestock)<br>7 Consumption (meat)<br>8 Consumption (byproduct)<br>9 Savings<br>10 Traction/Transport<br>88 Others (Specify below)<br>Custom alignment: LV
516	ao_goat_purpose_main_other<br>Show the field ONLY if:<br>[ao_goat_purpose(88)] = '1'	AO2e) Specify Other main purpose:	text<br>Custom alignment: LV
517	ao_goat_day<br>Show the field ONLY if:<br>[ao_animal_ownership(3)] = '1'	AO2f) Where are they kept during the day?<br>*Check all that apply*	checkbox<br>1 ao_goat_day___1 Inside house<br>2 ao_goat_day___2 Inside homestead (outside house)<br>3 ao_goat_day___3 Outside homestead<br>Custom alignment: LV
518	ao_goat_day_in_confine<br>Show the field ONLY if:<br>[ao_goat_day(1)] = '1'	AO2g) When those goat are inside the house, are they confined while inside the house?	yesno<br>1 Yes<br>0 No<br>Custom alignment: LV
519	ao_goat_day_in_how<br>Show the field ONLY if:<br>[ao_goat_day_in_confine] = '1'	AO2h) How are they confined inside the house?	radio<br>1 Tied<br>2 Confined inside a structure/room<br>3 Cooped<br>4 Corralled<br>88 Other (Specify below)<br>Custom alignment: LV
520	ao_goat_day_in_confine_other<br>Show the field ONLY if:<br>[ao_goat_day_in_how] = '88'	AO2i) Specify Other type of confinement:	text<br>Custom alignment: LV
521	ao_goat_homestead<br>Show the field ONLY if:<br>[ao_goat_day(2)] = '1'	AO2k) When those goat are inside the homestead, but not in the house, are the animals confined?	yesno<br>1 Yes<br>0 No<br>Custom alignment: LV
522	ao_goat_homestead_conf<br>Show the field ONLY if:<br>[ao_goat_homestead] = '1'	How are they confined inside the homestead?	radio<br>1 Tied<br>2 Confined inside a structure/room<br>3 Cooped<br>4 Corralled<br>88 Other (specify below)<br>Custom alignment: LV

523	ao_goat_homestead_conf_o<br>Show the field ONLY if:<br>[ao_goat_homestead_conf] = '88'	Specify other type of confinement	text<br>Custom alignment: LV
524	ao_goat_out_conf<br>Show the field ONLY if:<br>[ao_goat_day(3)] = '1'	Are the goat confined when they are outside homestead?	yesno<br>1 Yes<br>0 No<br>Custom alignment: LV
525	ao_goat_day_out_how<br>Show the field ONLY if:<br>[ao_goat_out_conf] = '1'	AO2n) How are they confined outside the homestead?	radio<br>1 Tied<br>2 Confined<br>3 Cooped<br>4 Corralled<br>88 Other (Specify below)<br>Custom alignment: LV
526	ao_goat_day_out_other<br>Show the field ONLY if:<br>[ao_goat_day_out_how] = '88'	AO2o) Specify Other confinement:	text<br>Custom alignment: LV
527	ao_goat_night<br>Show the field ONLY if:<br>[ao_animal_ownership(3)] = '1'	AO2p) Where are they kept/sleep during the night?<br>*Check all that apply*	checkbox<br>1 ao_goat_night___1 Inside house<br>2 ao_goat_night___2 Inside homestead (outside house)<br>3 ao_goat_night___3 Outside homestead<br>Custom alignment: LV
528	ao_goat_night_home_conf<br>Show the field ONLY if:<br>[ao_goat_night(1)] = '1'	AO2r) When those goat are inside the house at night, are they confined while inside the house?	yesno<br>1 Yes<br>0 No<br>Custom alignment: LV
529	ao_goat_night_in_how<br>Show the field ONLY if:<br>[ao_goat_night_home_conf] = '1'	AO2r) How are they confined inside house?	radio<br>1 Tied<br>2 Confined inside a structure/room<br>3 Cooped<br>4 Corralled<br>88 Other (Specify below)<br>Custom alignment: LV
530	ao_goat_night_in_oth<br>Show the field ONLY if:<br>[ao_goat_night_in_how] = '88'	AO2t) Specify Other confinement:	text<br>Custom alignment: LV
531	ao_goat_homestead_nt_con<br>Show the field ONLY if:<br>[ao_goat_night(2)] = '1'	AO2u) When those goat are inside the homestead but outside the house at night, are the animals confined?	yesno<br>1 Yes<br>0 No<br>Custom alignment: LV
532	ao_goat_homestead_nt_con_how<br>Show the field ONLY if:<br>[ao_goat_homestead_nt_con] = '1'	How are they confined inside homestead?	radio<br>1 Tied<br>2 Confined inside a structure/room<br>3 Cooped<br>4 Corralled<br>88 Other (Specify below)<br>Custom alignment: LV

533	ao_goat_homestead_nt_con_o<br>Show the field ONLY if:<br>[ao_goat_homestead_nt_con_how] = '88'	AO2w) Specify Other confinement:	text<br>Custom alignment: LV
534	ao_goat_out_nt_conf<br>Show the field ONLY if:<br>[ao_goat_night(3)] = '1'	AO2x) Are the goat confined when they are outside homestead?	yesno<br>1 Yes<br>0 No<br>Custom alignment: LV
535	ao_goat_out_nt_conf_how<br>Show the field ONLY if:<br>[ao_goat_out_nt_conf] ='1'	AO2y) How are they confined outside homestead?	radio<br>1 Tied<br>2 Confined inside a structure/room<br>3 Cooped<br>4 Corralled<br>88 Other (Specify below)<br>Custom alignment: LV
536	ao_goat_night_out_oth<br>Show the field ONLY if:<br>[ao_goat_out_nt_conf] = '88'	AO2z) Specify Other confinement:	text<br>Custom alignment: LV
537	ao_sheep_number<br>Show the field ONLY if:<br>[ao_animal_ownership(4)] = '1'	AO2b) How many sheep does your household have?	text (number, Min: 1)<br>Custom alignment: LV
538	ao_sheep_purpose<br>Show the field ONLY if:<br>[ao_animal_ownership(4)] = '1'	AO2b) For what purposes do you keep these sheep ? (check all that apply)	checkbox<br>1 ao_sheep_purpose___1 Income (meat)<br>2 ao_sheep_purpose___2 Income (byproduct)<br>3 ao_sheep_purpose___3 Income (livestock)<br>4 ao_sheep_purpose___4 Trade (meat)<br>5 ao_sheep_purpose___5 Trade (byproduct)<br>6 ao_sheep_purpose___6 Trade (livestock)<br>7 ao_sheep_purpose___7 Consumption (meat)<br>8 ao_sheep_purpose___8 Consumption (byproduct)<br>9 ao_sheep_purpose___9 Savings<br>10 ao_sheep_purpose___10 Traction/Transport<br>88 ao_sheep_purpose___88 Others (Specify below)<br>Custom alignment: LV
539	ao_sheep_purpose_other<br>Show the field ONLY if:<br>[ao_sheep_purpose(88)] = '1'	AO2c) Specify Other purpose(s):	text<br>Custom alignment: LV

540	ao_sheep_purpose_main<br><br>Show the field ONLY if:<br>[ao_animal_ownership(4)] = '1'	AO2d) Which of these is the main purpose for which you keep these sheep? (check only one)	radio<br>1 Income (meat)<br>2 Income (byproduct)<br>3 Income (livestock)<br>4 Trade (meat)<br>5 Trade (byproduct)<br>6 Trade (livestock)<br>7 Consumption (meat)<br>8 Consumption (byproduct)<br>9 Savings<br>10 Traction/Transport<br>88 Others (Specify below)<br><br>Custom alignment: LV
541	ao_sheep_purpose_main_other<br><br>Show the field ONLY if:<br>[ao_sheep_purpose(88)] = '1'	AO2e) Specify Other main purpose:	text<br>Custom alignment: LV
542	ao_sheep_day<br><br>Show the field ONLY if:<br>[ao_animal_ownership(4)] = '1'	AO2f) Where are they kept during the day?<br>*Check all that apply*	checkbox<br>1 ao_sheep_day___1 Inside house<br>2 ao_sheep_day___2 Inside homestead (outside house)<br>3 ao_sheep_day___3 Outside homestead<br><br>Custom alignment: LV
543	ao_sheep_day_in_confine<br><br>Show the field ONLY if:<br>[ao_sheep_day(1)] = '1'	AO2g) When those sheep are inside the house, are they confined while inside the house?	yesno<br>1 Yes<br>0 No<br><br>Custom alignment: LV
544	ao_sheep_day_in_how<br><br>Show the field ONLY if:<br>[ao_sheep_day_in_confine] = '1'	AO2h) How are they confined inside the house?	radio<br>1 Tied<br>2 Confined inside a structure/room<br>3 Cooped<br>4 Corralled<br>88 Other (Specify below)<br><br>Custom alignment: LV
545	ao_sheep_day_in_confine_other<br><br>Show the field ONLY if:<br>[ao_sheep_day_in_how] = '88'	AO2i) Specify Other type of confinement:	text<br>Custom alignment: LV
546	ao_sheep_homestead<br><br>Show the field ONLY if:<br>[ao_sheep_day(2)] = '1'	AO2k) When those sheep are inside the homestead, but not in the house, are the animals confined?	yesno<br>1 Yes<br>0 No<br><br>Custom alignment: LV
547	ao_sheep_homestead_conf<br><br>Show the field ONLY if:<br>[ao_sheep_homestead] = '1'	How are they confined inside the homestead?	radio<br>1 Tied<br>2 Confined inside a structure/room<br>3 Cooped<br>4 Corralled<br>88 Other (specify below)<br><br>Custom alignment: LV

548	ao_sheep_homestead_conf_o<br>Show the field ONLY if:<br>[ao_sheep_homestead_conf] = '88'	Specify other type of confinement	text<br>Custom alignment: LV
549	ao_sheep_out_conf<br>Show the field ONLY if:<br>[ao_sheep_day(3)] = '1'	Are the sheep confined when they are outside homestead?	yesno<br>1 Yes<br>0 No<br>Custom alignment: LV
550	ao_sheep_day_out_how<br>Show the field ONLY if:<br>[ao_sheep_out_conf] = '1'	AO2n) How are they confined outside the homestead?	radio<br>1 Tied<br>2 Confined<br>3 Cooped<br>4 Corralled<br>88 Other (Specify below)<br>Custom alignment: LV
551	ao_sheep_day_out_other<br>Show the field ONLY if:<br>[ao_sheep_day_out_how] = '88'	AO2o) Specify Other confinement:	text<br>Custom alignment: LV
552	ao_sheep_night<br>Show the field ONLY if:<br>[ao_animal_ownership(4)] = '1'	AO2p) Where are they kept/sleep during the night?<br>*Check all that apply*	checkbox<br>1 ao_sheep_night___1 Inside house<br>2 ao_sheep_night___2 Inside homestead (outside house)<br>3 ao_sheep_night___3 Outside homestead<br>Custom alignment: LV
553	ao_sheep_night_home_conf<br>Show the field ONLY if:<br>[ao_sheep_night(1)] = '1'	AO2r) When those sheep are inside the house at night, are they confined while inside the house?	yesno<br>1 Yes<br>0 No<br>Custom alignment: LV
554	ao_sheep_night_in_how<br>Show the field ONLY if:<br>[ao_sheep_night_home_conf] = '1'	AO2r) How are they confined inside house?	radio<br>1 Tied<br>2 Confined inside a structure/room<br>3 Cooped<br>4 Corralled<br>88 Other (Specify below)<br>Custom alignment: LV
555	ao_sheep_night_in_oth<br>Show the field ONLY if:<br>[ao_sheep_night_in_how] = '88'	AO2t) Specify Other confinement:	text<br>Custom alignment: LV
556	ao_sheep_homestead_nt_con<br>Show the field ONLY if:<br>[ao_sheep_night(2)] = '1'	AO2u) When those sheep are inside the homestead but outside the house at night, are the animals confined?	yesno<br>1 Yes<br>0 No<br>Custom alignment: LV
557	ao_sheep_homestead_nt_con_how<br>Show the field ONLY if:<br>[ao_sheep_homestead_nt_con] = '1'	How are they confined inside homestead?	radio<br>1 Tied<br>2 Confined inside a structure/room<br>3 Cooped<br>4 Corralled<br>88 Other (Specify below)<br>Custom alignment: LV

558	ao_sheep_homestead_nt_con_o<br>Show the field ONLY if:<br>[ao_sheep_homestead_nt_con_how] = '88'	AO2w) Specify Other confinement:	text<br>Custom alignment: LV
559	ao_sheep_out_nt_conf<br>Show the field ONLY if:<br>[ao_sheep_night(3)] = '1'	AO2x) Are the sheep confined when they are outside homestead?	yesno<br>1 Yes<br>0 No<br>Custom alignment: LV
560	ao_sheep_out_nt_conf_how<br>Show the field ONLY if:<br>[ao_sheep_out_nt_conf] ='1'	AO2y) How are they confined outside homestead?	radio<br>1 Tied<br>2 Confined inside a structure/room<br>3 Cooped<br>4 Corralled<br>88 Other (Specify below)<br>Custom alignment: LV
561	ao_sheep_night_out_oth<br>Show the field ONLY if:<br>[ao_sheep_out_nt_conf] = '88'	AO2z) Specify Other confinement:	text<br>Custom alignment: LV
562	ao_chicken_number<br>Show the field ONLY if:<br>[ao_animal_ownership(5)] = '1'	AO2b) How many chicken does your household have?	text (number, Min: 1)<br>Custom alignment: LV
563	ao_chicken_purpose<br>Show the field ONLY if:<br>[ao_animal_ownership(5)] = '1'	AO2b) For what purposes do you keep these chicken ? (check all that apply)	checkbox<br>1 ao_chicken_purpose___1 Income (meat)<br>2 ao_chicken_purpose___2 Income (byproduct)<br>3 ao_chicken_purpose___3 Income (livestock)<br>4 ao_chicken_purpose___4 Trade (meat)<br>5 ao_chicken_purpose___5 Trade (byproduct)<br>6 ao_chicken_purpose___6 Trade (livestock)<br>7 ao_chicken_purpose___7 Consumption (meat)<br>8 ao_chicken_purpose___8 Consumption (byproduct)<br>9 ao_chicken_purpose___9 Savings<br>10 ao_chicken_purpose___10 Traction/Transport<br>88 ao_chicken_purpose___88 Others (Specify below)<br>Custom alignment: LV
564	ao_chicken_purpose_other<br>Show the field ONLY if:<br>[ao_chicken_purpose(88)] = '1'	AO2c) Specify Other purpose(s):	text<br>Custom alignment: LV

565	ao_chicken_purpose_main<br><br>Show the field ONLY if:<br>[ao_animal_ownership(5)] = '1'	AO2d) Which of these is the main purpose for which you keep these chicken? (check only one)	radio<br>1 Income (meat)<br>2 Income (byproduct)<br>3 Income (livestock)<br>4 Trade (meat)<br>5 Trade (byproduct)<br>6 Trade (livestock)<br>7 Consumption (meat)<br>8 Consumption (byproduct)<br>9 Savings<br>10 Traction/Transport<br>88 Others (Specify below)<br><br>Custom alignment: LV
566	ao_chicken_purpose_main_other<br><br>Show the field ONLY if:<br>[ao_chicken_purpose(88)] = '1'	AO2e) Specify Other main purpose:	text<br>Custom alignment: LV
567	ao_chicken_day<br><br>Show the field ONLY if:<br>[ao_animal_ownership(5)] = '1'	AO2f) Where are they kept during the day?<br>*Check all that apply*	checkbox<br>1 ao_chicken_day___1 Inside house<br>2 ao_chicken_day___2 Inside homestead (outside house)<br>3 ao_chicken_day___3 Outside homestead<br><br>Custom alignment: LV
568	ao_chicken_day_in_confine<br><br>Show the field ONLY if:<br>[ao_chicken_day(1)] = '1'	AO2g) When those chicken are inside the house, are they confined while inside the house?	yesno<br>1 Yes<br>0 No<br><br>Custom alignment: LV
569	ao_chicken_day_in_how<br><br>Show the field ONLY if:<br>[ao_chicken_day_in_confine] = '1'	AO2h) How are they confined inside the house?	radio<br>1 Tied<br>2 Confined inside a structure/room<br>3 Cooped<br>4 Corralled<br>88 Other (Specify below)<br><br>Custom alignment: LV
570	ao_chicken_day_in_confine_other<br><br>Show the field ONLY if:<br>[ao_chicken_day_in_how] = '88'	AO2i) Specify Other type of confinement:	text<br>Custom alignment: LV
571	ao_chicken_homestead<br><br>Show the field ONLY if:<br>[ao_chicken_day(2)] = '1'	AO2k) When those chicken are inside the homestead, but not in the house, are the animals confined?	yesno<br>1 Yes<br>0 No<br><br>Custom alignment: LV
572	ao_chicken_homestead_conf<br><br>Show the field ONLY if:<br>[ao_chicken_homestead] = '1'	How are they confined inside the homestead?	radio<br>1 Tied<br>2 Confined inside a structure/room<br>3 Cooped<br>4 Corralled<br>88 Other (specify below)<br><br>Custom alignment: LV

573	ao_chicken_homestead_conf_o<br>Show the field ONLY if:<br>[ao_chicken_homestead_conf] = '88'	Specify other type of confinement	text<br>Custom alignment: LV
574	ao_chicken_out_conf<br>Show the field ONLY if:<br>[ao_chicken_day(3)] = '1'	Are the chicken confined when they are outside homestead?	yesno<br>1 Yes<br>0 No<br>Custom alignment: LV
575	ao_chicken_day_out_how<br>Show the field ONLY if:<br>[ao_chicken_out_conf] = '1'	AO2n) How are they confined outside the homestead?	radio<br>1 Tied<br>2 Confined<br>3 Cooped<br>4 Corralled<br>88 Other (Specify below)<br>Custom alignment: LV
576	ao_chicken_day_out_other<br>Show the field ONLY if:<br>[ao_chicken_day_out_how] = '88'	AO2o) Specify Other confinement:	text<br>Custom alignment: LV
577	ao_chicken_night<br>Show the field ONLY if:<br>[ao_animal_ownership(5)] = '1'	AO2p) Where are they kept/sleep during the night?<br>*Check all that apply*	checkbox<br>1 ao_chicken_night___1 Inside house<br>2 ao_chicken_night___2 Inside homestead (outside house)<br>3 ao_chicken_night___3 Outside homestead<br>Custom alignment: LV
578	ao_chicken_night_home_conf<br>Show the field ONLY if:<br>[ao_chicken_night(1)] = '1'	AO2r) When those chicken are inside the house at night, are they confined while inside the house?	yesno<br>1 Yes<br>0 No<br>Custom alignment: LV
579	ao_chicken_night_in_how<br>Show the field ONLY if:<br>[ao_chicken_night_home_conf] = '1'	AO2r) How are they confined inside house?	radio<br>1 Tied<br>2 Confined inside a structure/room<br>3 Cooped<br>4 Corralled<br>88 Other (Specify below)<br>Custom alignment: LV
580	ao_chicken_night_in_oth<br>Show the field ONLY if:<br>[ao_chicken_night_in_how] = '88'	AO2t) Specify Other confinement:	text<br>Custom alignment: LV
581	ao_chicken_homestead_nt_con<br>Show the field ONLY if:<br>[ao_chicken_night(2)] = '1'	AO2u) When those chicken are inside the homestead but outside the house at night, are the animals confined?	yesno<br>1 Yes<br>0 No<br>Custom alignment: LV

582	ao_chicken_homestead_nt_con_how<br>Show the field ONLY if:<br>[ao_chicken_homestead_nt_con] = '1'	How are they confined inside homestead?	radio<br>1 Tied<br>2 Confined inside a structure/room<br>3 Cooped<br>4 Corralled<br>88 Other (Specify below)<br>Custom alignment: LV
583	ao_chicken_homestead_nt_con_o<br>Show the field ONLY if:<br>[ao_chicken_homestead_nt_con_how] = '88'	AO2w) Specify Other confinement:	text<br>Custom alignment: LV
584	ao_chicken_out_nt_conf<br>Show the field ONLY if:<br>[ao_chicken_night(3)] = '1'	AO2x) Are the chicken confined when they are outside homestead?	yesno<br>1 Yes<br>0 No<br>Custom alignment: LV
585	ao_chicken_out_nt_conf_how<br>Show the field ONLY if:<br>[ao_chicken_out_nt_conf] ='1'	AO2y) How are they confined outside homestead?	radio<br>1 Tied<br>2 Confined inside a structure/room<br>3 Cooped<br>4 Corralled<br>88 Other (Specify below)<br>Custom alignment: LV
586	ao_chicken_night_out_oth<br>Show the field ONLY if:<br>[ao_chicken_out_nt_conf] = '88'	AO2z) Specify Other confinement:	text<br>Custom alignment: LV
587	ao_dog_number<br>Show the field ONLY if:<br>[ao_animal_ownership(6)] = '1'	AO2b) How many dog does your household have?	text (number, Min: 1)<br>Custom alignment: LV
588	ao_dog_purpose<br>Show the field ONLY if:<br>[ao_animal_ownership(6)] = '1'	AO2b) For what purposes do you keep these dog ? (check all that apply)	checkbox<br>1 ao_dog_purpose___1 Income (meat)<br>2 ao_dog_purpose___2 Income (byproduct)<br>3 ao_dog_purpose___3 Income (livestock)<br>4 ao_dog_purpose___4 Trade (meat)<br>5 ao_dog_purpose___5 Trade (byproduct)<br>6 ao_dog_purpose___6 Trade (livestock)<br>7 ao_dog_purpose___7 Consumption (meat)<br>8 ao_dog_purpose___8 Consumption (byproduct)<br>9 ao_dog_purpose___9 Savings<br>10 ao_dog_purpose___10 Traction/Transport<br>88 ao_dog_purpose___88 Others (Specify below)<br>Custom alignment: LV
589	ao_dog_purpose_other<br>Show the field ONLY if:<br>[ao_dog_purpose(88)] = '1'	AO2c) Specify Other purpose(s):	text<br>Custom alignment: LV

590	ao_dog_purpose_main<br>Show the field ONLY if:<br>[ao_animal_ownership(6)] = '1'	AO2d) Which of these is the main purpose for which you keep these dog? (check only one)	radio<br>1 Income (meat)<br>2 Income (byproduct)<br>3 Income (livestock)<br>4 Trade (meat)<br>5 Trade (byproduct)<br>6 Trade (livestock)<br>7 Consumption (meat)<br>8 Consumption (byproduct)<br>9 Savings<br>10 Traction/Transport<br>88 Others (Specify below)<br>Custom alignment: LV
591	ao_dog_purpose_main_other<br>Show the field ONLY if:<br>[ao_dog_purpose(88)] = '1'	AO2e) Specify Other main purpose:	text<br>Custom alignment: LV
592	ao_dog_day<br>Show the field ONLY if:<br>[ao_animal_ownership(6)] = '1'	AO2f) Where are they kept during the day?<br>*Check all that apply*	checkbox<br>1 ao_dog_day___1 Inside house<br>2 ao_dog_day___2 Inside homestead (outside house)<br>3 ao_dog_day___3 Outside homestead<br>Custom alignment: LV
593	ao_dog_day_in_confine<br>Show the field ONLY if:<br>[ao_dog_day(1)] = '1'	AO2g) When those dog are inside the house, are they confined while inside the house?	yesno<br>1 Yes<br>0 No<br>Custom alignment: LV
594	ao_dog_day_in_how<br>Show the field ONLY if:<br>[ao_dog_day_in_confine] = '1'	AO2h) How are they confined inside the house?	radio<br>1 Tied<br>2 Confined inside a structure/room<br>3 Cooped<br>4 Corralled<br>88 Other (Specify below)<br>Custom alignment: LV
595	ao_dog_day_in_confine_other<br>Show the field ONLY if:<br>[ao_dog_day_in_how] = '88'	AO2i) Specify Other type of confinement:	text<br>Custom alignment: LV
596	ao_dog_homestead<br>Show the field ONLY if:<br>[ao_dog_day(2)] = '1'	AO2k) When those dog are inside the homestead, but not in the house, are the animals confined?	yesno<br>1 Yes<br>0 No<br>Custom alignment: LV
597	ao_dog_homestead_conf<br>Show the field ONLY if:<br>[ao_dog_homestead] = '1'	How are they confined inside the homestead?	radio<br>1 Tied<br>2 Confined inside a structure/room<br>3 Cooped<br>4 Corralled<br>88 Other (specify below)<br>Custom alignment: LV

598	ao_dog_homestead_conf_o<br>Show the field ONLY if:<br>[ao_dog_homestead_conf] = '88'	Specify other type of confinement	text<br>Custom alignment: LV
599	ao_dog_out_conf<br>Show the field ONLY if:<br>[ao_dog_day(3)] = '1'	Are the dog confined when they are outside homestead?	yesno<br>1 Yes<br>0 No<br>Custom alignment: LV
600	ao_dog_day_out_how<br>Show the field ONLY if:<br>[ao_dog_out_conf] = '1'	AO2n) How are they confined outside the homestead?	radio<br>1 Tied<br>2 Confined<br>3 Cooped<br>4 Corralled<br>88 Other (Specify below)<br>Custom alignment: LV
601	ao_dog_day_out_other<br>Show the field ONLY if:<br>[ao_dog_day_out_how] = '88'	AO2o) Specify Other confinement:	text<br>Custom alignment: LV
602	ao_dog_night<br>Show the field ONLY if:<br>[ao_animal_ownership(6)] = '1'	AO2p) Where are they kept/sleep during the night?<br>*Check all that apply*	checkbox<br>1 ao_dog_night___1 Inside house<br>2 ao_dog_night___2 Inside homestead (outside house)<br>3 ao_dog_night___3 Outside homestead<br>Custom alignment: LV
603	ao_dog_night_home_conf<br>Show the field ONLY if:<br>[ao_dog_night(1)] = '1'	AO2r) When those dog are inside the house at night, are they confined while inside the house?	yesno<br>1 Yes<br>0 No<br>Custom alignment: LV
604	ao_dog_night_in_how<br>Show the field ONLY if:<br>[ao_dog_night_home_conf] = '1'	AO2r) How are they confined inside house?	radio<br>1 Tied<br>2 Confined inside a structure/room<br>3 Cooped<br>4 Corralled<br>88 Other (Specify below)<br>Custom alignment: LV
605	ao_dog_night_in_oth<br>Show the field ONLY if:<br>[ao_dog_night_in_how] = '88'	AO2t) Specify Other confinement:	text<br>Custom alignment: LV
606	ao_dog_homestead_nt_con<br>Show the field ONLY if:<br>[ao_dog_night(2)] = '1'	AO2u) When those dog are inside the homestead but outside the house at night, are the animals confined?	yesno<br>1 Yes<br>0 No<br>Custom alignment: LV
607	ao_dog_homestead_nt_con_how<br>Show the field ONLY if:<br>[ao_dog_homestead_nt_con] = '1'	How are they confined inside homestead?	radio<br>1 Tied<br>2 Confined inside a structure/room<br>3 Cooped<br>4 Corralled<br>88 Other (Specify below)<br>Custom alignment: LV

608	ao_dog_homestead_nt_con_o<br>Show the field ONLY if:<br>[ao_dog_homestead_nt_con_how] = '88'	AO2w) Specify Other confinement:	text<br>Custom alignment: LV
609	ao_dog_out_nt_conf<br>Show the field ONLY if:<br>[ao_dog_night(3)] = '1'	AO2x) Are the dog confined when they are outside homestead?	yesno<br>1 Yes<br>0 No<br>Custom alignment: LV
610	ao_dog_out_nt_conf_how<br>Show the field ONLY if:<br>[ao_dog_out_nt_conf] ='1'	AO2y) How are they confined outside homestead?	radio<br>1 Tied<br>2 Confined inside a structure/room<br>3 Cooped<br>4 Corralled<br>88 Other (Specify below)<br>Custom alignment: LV
611	ao_dog_night_out_oth<br>Show the field ONLY if:<br>[ao_dog_out_nt_conf] = '88'	AO2z) Specify Other confinement:	text<br>Custom alignment: LV
612	ao_cat_number<br>Show the field ONLY if:<br>[ao_animal_ownership(7)] = '1'	AO2b) How many cat does your household have?	text (number, Min: 1)<br>Custom alignment: LV
613	ao_cat_purpose<br>Show the field ONLY if:<br>[ao_animal_ownership(7)] = '1'	AO2b) For what purposes do you keep these cat ? (check all that apply)	checkbox<br>1 ao_cat_purpose___1 Income (meat)<br>2 ao_cat_purpose___2 Income (byproduct)<br>3 ao_cat_purpose___3 Income (livestock)<br>4 ao_cat_purpose___4 Trade (meat)<br>5 ao_cat_purpose___5 Trade (byproduct)<br>6 ao_cat_purpose___6 Trade (livestock)<br>7 ao_cat_purpose___7 Consumption (meat)<br>8 ao_cat_purpose___8 Consumption (byproduct)<br>9 ao_cat_purpose___9 Savings<br>10 ao_cat_purpose___10 Traction/Transport<br>88 ao_cat_purpose___88 Others (Specify below)<br>Custom alignment: LV
614	ao_cat_purpose_other<br>Show the field ONLY if:<br>[ao_cat_purpose(88)] = '1'	AO2c) Specify Other purpose(s):	text<br>Custom alignment: LV
615	ao_cat_purpose_main<br>Show the field ONLY if:<br>[ao_animal_ownership(7)] = '1'	AO2d) Which of these is the main purpose for which you keep these cat? (check only one)	radio<br>1 Income (meat)<br>2 Income (byproduct)<br>3 Income (livestock)<br>4 Trade (meat)<br>5 Trade (byproduct)<br>6 Trade (livestock)<br>7 Consumption (meat)<br>8 Consumption (byproduct)<br>9 Savings<br>10 Traction/Transport<br>88 Others (Specify below)<br>Custom alignment: LV

616	ao_cat_purpose_main_other<br>Show the field ONLY if:<br>[ao_cat_purpose(88)] = '1'	AO2e) Specify Other main purpose:	text<br>Custom alignment: LV
617	ao_cat_day<br>Show the field ONLY if:<br>[ao_animal_ownership(7)] = '1'	AO2f) Where are they kept during the day?<br>*Check all that apply*	checkbox<br>1 ao_cat_day___1 Inside house<br>2 ao_cat_day___2 Inside homestead (outside house)<br>3 ao_cat_day___3 Outside homestead<br>Custom alignment: LV
618	ao_cat_day_in_confine<br>Show the field ONLY if:<br>[ao_cat_day(1)] = '1'	AO2g) When those cat are inside the house, are they confined while inside the house?	yesno<br>1 Yes<br>0 No<br>Custom alignment: LV
619	ao_cat_day_in_how<br>Show the field ONLY if:<br>[ao_cat_day_in_confine] = '1'	AO2h) How are they confined inside the house?	radio<br>1 Tied<br>2 Confined inside a structure/room<br>3 Cooped<br>4 Corralled<br>88 Other (Specify below)<br>Custom alignment: LV
620	ao_cat_day_in_confine_other<br>Show the field ONLY if:<br>[ao_cat_day_in_how] = '88'	AO2i) Specify Other type of confinement:	text<br>Custom alignment: LV
621	ao_cat_homestead<br>Show the field ONLY if:<br>[ao_cat_day(2)] = '1'	AO2k) When those cat are inside the homestead, but not in the house, are the animals confined?	yesno<br>1 Yes<br>0 No<br>Custom alignment: LV
622	ao_cat_homestead_conf<br>Show the field ONLY if:<br>[ao_cat_homestead] = '1'	How are they confined inside the homestead?	radio<br>1 Tied<br>2 Confined inside a structure/room<br>3 Cooped<br>4 Corralled<br>88 Other (specify below)<br>Custom alignment: LV
623	ao_cat_homestead_conf_o<br>Show the field ONLY if:<br>[ao_cat_homestead_conf] = '88'	Specify other type of confinement	text<br>Custom alignment: LV
624	ao_cat_out_conf<br>Show the field ONLY if:<br>[ao_cat_day(3)] = '1'	Are the cat confined when they are outside homestead?	yesno<br>1 Yes<br>0 No<br>Custom alignment: LV
625	ao_cat_day_out_how<br>Show the field ONLY if:<br>[ao_cat_out_conf] = '1'	AO2n) How are they confined outside the homestead?	radio<br>1 Tied<br>2 Confined<br>3 Cooped<br>4 Corralled<br>88 Other (Specify below)<br>Custom alignment: LV

626	ao_cat_day_out_other<br>Show the field ONLY if:<br>[ao_cat_day_out_how] = '88'	AO2o) Specify Other confinement:	text<br>Custom alignment: LV
627	ao_cat_night<br>Show the field ONLY if:<br>[ao_animal_ownership(7)] = '1'	AO2p) Where are they kept/sleep during the night?<br>*Check all that apply*	checkbox<br>1 ao_cat_night___1 Inside house<br>2 ao_cat_night___2 Inside homestead (outside house)<br>3 ao_cat_night___3 Outside homestead<br>Custom alignment: LV
628	ao_cat_night_home_conf<br>Show the field ONLY if:<br>[ao_cat_night(1)] = '1'	AO2r) When those cat are inside the house at night, are they confined while inside the house?	yesno<br>1 Yes<br>0 No<br>Custom alignment: LV
629	ao_cat_night_in_how<br>Show the field ONLY if:<br>[ao_cat_night_home_conf] = '1'	AO2r) How are they confined inside house?	radio<br>1 Tied<br>2 Confined inside a structure/room<br>3 Cooped<br>4 Corralled<br>88 Other (Specify below)<br>Custom alignment: LV
630	ao_cat_night_in_oth<br>Show the field ONLY if:<br>[ao_cat_night_in_how] = '88'	AO2t) Specify Other confinement:	text<br>Custom alignment: LV
631	ao_cat_homestead_nt_con<br>Show the field ONLY if:<br>[ao_cat_night(2)] = '1'	AO2u) When those cat are inside the homestead but outside the house at night, are the animals confined?	yesno<br>1 Yes<br>0 No<br>Custom alignment: LV
632	ao_cat_homestead_nt_con_how<br>Show the field ONLY if:<br>[ao_cat_homestead_nt_con] = '1'	How are they confined inside homestead?	radio<br>1 Tied<br>2 Confined inside a structure/room<br>3 Cooped<br>4 Corralled<br>88 Other (Specify below)<br>Custom alignment: LV
633	ao_cat_homestead_nt_con_o<br>Show the field ONLY if:<br>[ao_cat_homestead_nt_con_how] = '88'	AO2w) Specify Other confinement:	text<br>Custom alignment: LV
634	ao_cat_out_nt_conf<br>Show the field ONLY if:<br>[ao_cat_night(3)] = '1'	AO2x) Are the cat confined when they are outside homestead?	yesno<br>1 Yes<br>0 No<br>Custom alignment: LV
635	ao_cat_out_nt_conf_how<br>Show the field ONLY if:<br>[ao_cat_out_nt_conf] ='1'	AO2y) How are they confined outside homestead?	radio<br>1 Tied<br>2 Confined inside a structure/room<br>3 Cooped<br>4 Corralled<br>88 Other (Specify below)<br>Custom alignment: LV

636	ao_cat_night_out_oth<br><br>Show the field ONLY if:<br>[ao_cat_out_nt_conf] = '88'	AO2z) Specify Other confinement:	text<br>Custom alignment: LV
637	ao_camel_number<br><br>Show the field ONLY if:<br>[ao_animal_ownership(8)] = '1'	AO2b) How many camel does your household have?	text (number, Min: 1)<br>Custom alignment: LV
638	ao_camel_purpose<br><br>Show the field ONLY if:<br>[ao_animal_ownership(8)] = '1'	AO2b) For what purposes do you keep these camel ? (check all that apply)	checkbox<br>1 ao_camel_purpose___1 Income (meat)<br>2 ao_camel_purpose___2 Income (byproduct)<br>3 ao_camel_purpose___3 Income (livestock)<br>4 ao_camel_purpose___4 Trade (meat)<br>5 ao_camel_purpose___5 Trade (byproduct)<br>6 ao_camel_purpose___6 Trade (livestock)<br>7 ao_camel_purpose___7 Consumption (meat)<br>8 ao_camel_purpose___8 Consumption (byproduct)<br>9 ao_camel_purpose___9 Savings<br>10 ao_camel_purpose___10 Traction/Transport<br>88 ao_camel_purpose___88 Others (Specify below)<br><br>Custom alignment: LV
639	ao_camel_purpose_other<br><br>Show the field ONLY if:<br>[ao_camel_purpose(88)] = '1'	AO2c) Specify Other purpose(s):	text<br>Custom alignment: LV
640	ao_camel_purpose_main<br><br>Show the field ONLY if:<br>[ao_animal_ownership(8)] = '1'	AO2d) Which of these is the main purpose for which you keep these camel? (check only one)	radio<br>1 Income (meat)<br>2 Income (byproduct)<br>3 Income (livestock)<br>4 Trade (meat)<br>5 Trade (byproduct)<br>6 Trade (livestock)<br>7 Consumption (meat)<br>8 Consumption (byproduct)<br>9 Savings<br>10 Traction/Transport<br>88 Others (Specify below)<br><br>Custom alignment: LV
641	ao_camel_purpose_main_other<br><br>Show the field ONLY if:<br>[ao_camel_purpose(88)] = '1'	AO2e) Specify Other main purpose:	text<br>Custom alignment: LV
642	ao_camel_day<br><br>Show the field ONLY if:<br>[ao_animal_ownership(8)] = '1'	AO2f) Where are they kept during the day?<br>*Check all that apply*	checkbox<br>1 ao_camel_day___1 Inside house<br>2 ao_camel_day___2 Inside homestead (outside house)<br>3 ao_camel_day___3 Outside homestead<br><br>Custom alignment: LV
643	ao_camel_day_in_confine<br><br>Show the field ONLY if:<br>[ao_camel_day(1)] = '1'	AO2g) When those camel are inside the house, are they confined while inside the house?	yesno<br>1 Yes<br>0 No<br><br>Custom alignment: LV

644	ao_camel_day_in_how<br>Show the field ONLY if: [ao_camel_day_in_confine] = '1'	AO2h) How are they confined inside the house?	radio<br>1 Tied<br>2 Confined inside a structure/room<br>3 Cooped<br>4 Corralled<br>88 Other (Specify below)<br>Custom alignment: LV
645	ao_camel_day_in_confine_other<br>Show the field ONLY if: [ao_camel_day_in_how] = '88'	AO2i) Specify Other type of confinement:	text<br>Custom alignment: LV
646	ao_camel_homestead<br>Show the field ONLY if: [ao_camel_day(2)] = '1'	AO2k) When those camel are inside the homestead, but not in the house, are the animals confined?	yesno<br>1 Yes<br>0 No<br>Custom alignment: LV
647	ao_camel_homestead_conf<br>Show the field ONLY if: [ao_camel_homestead] = '1'	How are they confined inside the homestead?	radio<br>1 Tied<br>2 Confined inside a structure/room<br>3 Cooped<br>4 Corralled<br>88 Other (specify below)<br>Custom alignment: LV
648	ao_camel_homestead_conf_o<br>Show the field ONLY if: [ao_camel_homestead_conf] = '88'	Specify other type of confinement	text<br>Custom alignment: LV
649	ao_camel_out_conf<br>Show the field ONLY if: [ao_camel_day(3)] = '1'	Are the camel confined when they are outside homestead?	yesno<br>1 Yes<br>0 No<br>Custom alignment: LV
650	ao_camel_day_out_how<br>Show the field ONLY if: [ao_camel_out_conf] = '1'	AO2n) How are they confined outside the homestead?	radio<br>1 Tied<br>2 Confined<br>3 Cooped<br>4 Corralled<br>88 Other (Specify below)<br>Custom alignment: LV
651	ao_camel_day_out_other<br>Show the field ONLY if: [ao_camel_day_out_how] = '88'	AO2o) Specify Other confinement:	text<br>Custom alignment: LV
652	ao_camel_night<br>Show the field ONLY if: [ao_animal_ownership(8)] = '1'	AO2p) Where are they kept/sleep during the night?<br>*Check all that apply*	checkbox<br>1 ao_camel_night___1 Inside house<br>2 ao_camel_night___2 Inside homestead (outside house)<br>3 ao_camel_night___3 Outside homestead<br>Custom alignment: LV

653	ao_camel_night_home_conf<br><br>Show the field ONLY if:<br>[ao_camel_night(1)] = '1'	AO2r) When those camel are inside the house at night, are they confined while inside the house?	yesno<br>1 Yes<br>0 No<br><br>Custom alignment: LV
654	ao_camel_night_in_how<br><br>Show the field ONLY if:<br>[ao_camel_night_home_conf] = '1'	AO2r) How are they confined inside house?	radio<br>1 Tied<br>2 Confined inside a structure/room<br>3 Cooped<br>4 Corralled<br>88 Other (Specify below)<br><br>Custom alignment: LV
655	ao_camel_night_in_oth<br><br>Show the field ONLY if:<br>[ao_camel_night_in_how] = '88'	AO2t) Specify Other confinement:	text<br>Custom alignment: LV
656	ao_camel_homestead_nt_con<br><br>Show the field ONLY if:<br>[ao_camel_night(2)] = '1'	AO2u) When those camel are inside the homestead but outside the house at night, are the animals confined?	yesno<br>1 Yes<br>0 No<br><br>Custom alignment: LV
657	ao_camel_homestead_nt_con_how<br><br>Show the field ONLY if:<br>[ao_camel_homestead_nt_con] = '1'	How are they confined inside homestead?	radio<br>1 Tied<br>2 Confined inside a structure/room<br>3 Cooped<br>4 Corralled<br>88 Other (Specify below)<br><br>Custom alignment: LV
658	ao_camel_homestead_nt_con_o<br><br>Show the field ONLY if:<br>[ao_camel_homestead_nt_con_how] = '88'	AO2w) Specify Other confinement:	text<br>Custom alignment: LV
659	ao_camel_out_nt_conf<br><br>Show the field ONLY if:<br>[ao_camel_night(3)] = '1'	AO2x) Are the camel confined when they are outside homestead?	yesno<br>1 Yes<br>0 No<br><br>Custom alignment: LV
660	ao_camel_out_nt_conf_how<br><br>Show the field ONLY if:<br>[ao_camel_out_nt_conf] ='1'	AO2y) How are they confined outside homestead?	radio<br>1 Tied<br>2 Confined inside a structure/room<br>3 Cooped<br>4 Corralled<br>88 Other (Specify below)<br><br>Custom alignment: LV
661	ao_camel_night_out_oth<br><br>Show the field ONLY if:<br>[ao_camel_out_nt_conf] = '88'	AO2z) Specify Other confinement:	text<br>Custom alignment: LV
662	ao_other_number_2<br><br>Show the field ONLY if:<br>[ao_animal_ownership(88)] = '1'	AO2b) Specify Other animal	text<br>Custom alignment: LV

663	ao_other_number<br>Show the field ONLY if: [ao_animal_ownership(88)] = '1'	AO2b) How many other (animal) does your household have?	text (number, Min: 1)<br>Custom alignment: LV
664	ao_other_purpose<br>Show the field ONLY if: [ao_animal_ownership(88)] = '1'	AO2b) For what purposes do you keep these other ? (check all that apply)	checkbox<br>1 ao_other_purpose___1 Income (meat)<br>2 ao_other_purpose___2 Income (byproduct)<br>3 ao_other_purpose___3 Income (livestock)<br>4 ao_other_purpose___4 Trade (meat)<br>5 ao_other_purpose___5 Trade (byproduct)<br>6 ao_other_purpose___6 Trade (livestock)<br>7 ao_other_purpose___7 Consumption (meat)<br>8 ao_other_purpose___8 Consumption (byproduct)<br>9 ao_other_purpose___9 Savings<br>10 ao_other_purpose___10 Traction/Transport<br>88 ao_other_purpose___88 Others (Specify below)<br>Custom alignment: LV
665	ao_other_purpose_other<br>Show the field ONLY if: [ao_other_purpose(88)] = '1'	AO2c) Specify Other purpose(s):	text<br>Custom alignment: LV
666	ao_other_purpose_main<br>Show the field ONLY if: [ao_animal_ownership(88)] = '1'	AO2d) Which of these is the main purpose for which you keep these other? (check only one)	radio<br>1 Income (meat)<br>2 Income (byproduct)<br>3 Income (livestock)<br>4 Trade (meat)<br>5 Trade (byproduct)<br>6 Trade (livestock)<br>7 Consumption (meat)<br>8 Consumption (byproduct)<br>9 Savings<br>10 Traction/Transport<br>88 Others (Specify below)<br>Custom alignment: LV
667	ao_other_purpose_main_other<br>Show the field ONLY if: [ao_other_purpose(88)] = '1'	AO2e) Specify Other main purpose:	text<br>Custom alignment: LV
668	ao_other_day<br>Show the field ONLY if: [ao_animal_ownership(88)] = '1'	AO2f) Where are they kept during the day?<br>*Check all that apply*	checkbox<br>1 ao_other_day___1 Inside house<br>2 ao_other_day___2 Inside homestead (outside house)<br>3 ao_other_day___3 Outside homestead<br>Custom alignment: LV
669	ao_other_day_in_confine<br>Show the field ONLY if: [ao_other_day(1)] = '1'	AO2g) When those other are inside the house, are they confined while inside the house?	yesno<br>1 Yes<br>0 No<br>Custom alignment: LV

670	ao_other_day_in_how<br>Show the field ONLY if:<br>[ao_other_day_in_confine] = '1'	AO2h) How are they confined inside the house?	radio<br>1 Tied<br>2 Confined inside a structure/room<br>3 Cooped<br>4 Corralled<br>88 Other (Specify below)<br>Custom alignment: LV
671	ao_other_day_in_confine_other<br>Show the field ONLY if:<br>[ao_other_day_in_how] = '88'	AO2i) Specify Other type of confinement:	text<br>Custom alignment: LV
672	ao_other_homestead<br>Show the field ONLY if:<br>[ao_other_day(2)] = '1'	AO2k) When those other are inside the homestead, but not in the house, are the animals confined?	yesno<br>1 Yes<br>0 No<br>Custom alignment: LV
673	ao_other_homestead_conf<br>Show the field ONLY if:<br>[ao_other_homestead] = '1'	How are they confined inside the homestead?	radio<br>1 Tied<br>2 Confined inside a structure/room<br>3 Cooped<br>4 Corralled<br>88 Other (specify below)<br>Custom alignment: LV
674	ao_other_homestead_conf_o<br>Show the field ONLY if:<br>[ao_other_homestead_conf] = '88'	Specify other type of confinement	text<br>Custom alignment: LV
675	ao_other_out_conf<br>Show the field ONLY if:<br>[ao_other_day(3)] = '1'	Are the other confined when they are outside homestead?	yesno<br>1 Yes<br>0 No<br>Custom alignment: LV
676	ao_other_day_out_how<br>Show the field ONLY if:<br>[ao_other_out_conf] = '1'	AO2n) How are they confined outside the homestead?	radio<br>1 Tied<br>2 Confined<br>3 Cooped<br>4 Corralled<br>88 Other (Specify below)<br>Custom alignment: LV
677	ao_other_day_out_other<br>Show the field ONLY if:<br>[ao_other_day_out_how] = '88'	AO2o) Specify Other confinement:	text<br>Custom alignment: LV
678	ao_other_night<br>Show the field ONLY if:<br>[ao_animal_ownership(88)] = '1'	AO2p) Where are they kept/sleep during the night?<br>*Check all that apply*	checkbox<br>1 ao_other_night___1 Inside house<br>2 ao_other_night___2 Inside homestead (outside house)<br>3 ao_other_night___3 Outside homestead<br>Custom alignment: LV

679	ao_other_night_home_conf<br>Show the field ONLY if:<br>[ao_other_night(1)] = '1'	AO2r) When those other are inside the house at night, are they confined while inside the house?	yesno<br>1 Yes<br>0 No<br>Custom alignment: LV
680	ao_other_night_in_how<br>Show the field ONLY if:<br>[ao_other_night_home_conf] = '1'	AO2r) How are they confined inside house?	radio<br>1 Tied<br>2 Confined inside a structure/room<br>3 Cooped<br>4 Corralled<br>88 Other (Specify below)<br>Custom alignment: LV
681	ao_other_night_in_oth<br>Show the field ONLY if:<br>[ao_other_night_in_how] = '88'	AO2t) Specify Other confinement:	text<br>Custom alignment: LV
682	ao_other_homestead_nt_con<br>Show the field ONLY if:<br>[ao_other_night(2)] = '1'	AO2u) When those other are inside the homestead but outside the house at night, are the animals confined?	yesno<br>1 Yes<br>0 No<br>Custom alignment: LV
683	ao_other_homestead_nt_con_how<br>Show the field ONLY if:<br>[ao_other_homestead_nt_con] = '1'	How are they confined inside homestead?	radio<br>1 Tied<br>2 Confined inside a structure/room<br>3 Cooped<br>4 Corralled<br>88 Other (Specify below)<br>Custom alignment: LV
684	ao_other_homestead_nt_con_o<br>Show the field ONLY if:<br>[ao_other_homestead_nt_con_how] = '88'	AO2w) Specify Other confinement:	text<br>Custom alignment: LV
685	ao_other_out_nt_conf<br>Show the field ONLY if:<br>[ao_other_night(3)] = '1'	AO2x) Are the other confined when they are outside homestead?	yesno<br>1 Yes<br>0 No<br>Custom alignment: LV
686	ao_other_out_nt_conf_how<br>Show the field ONLY if:<br>[ao_other_out_nt_conf] ='1'	AO2y) How are they confined outside homestead?	radio<br>1 Tied<br>2 Confined inside a structure/room<br>3 Cooped<br>4 Corralled<br>88 Other (Specify below)<br>Custom alignment: LV
687	ao_other_night_out_oth<br>Show the field ONLY if:<br>[ao_other_out_nt_conf] = '88'	AO2z) Specify Other confinement:	text<br>Custom alignment: LV
688	note9	Section Header: *Slaughtering*<br>Explain to the participant: I am now going to ask you questions relating to your household's animal slaughter practices. Please let me know if you need me to clarify any of my questions. Feel free to ask any question you may have.	descriptive<br>Custom alignment: LV

689	ao_slaughter	AO11) Do you slaughter your own or your family's animals?	yesno<br>1 Yes<br>0 No<br>Custom alignment: LV
690	ao_slaughter_species<br>Show the field ONLY if:<br>[ao_slaughter] = '1'	AO12) Which of the following species do you slaughter? (check all that apply)	checkbox<br>1 ao_slaughter_species___1 Cattle<br>2 ao_slaughter_species___2 Goat<br>3 ao_slaughter_species___3 Sheep<br>4 ao_slaughter_species___4 Chicken<br>Custom alignment: LV
691	ao_wash_slaughter<br>Show the field ONLY if:<br>[ao_slaughter] = '1'	AO12p) Do you wash your hands, before and/or after slaughtering your (animal)?	dropdown<br>1 Before<br>2 After<br>3 Before and After<br>4 Only sometimes<br>5 Neither
692	ao_slaughter_utensils<br>Show the field ONLY if:<br>[ao_slaughter] = '1'	Do you have designated utensils that you use only for slaughtering (animal)?	dropdown<br>1 Yes<br>2 No, the same utensils are used to slaughter other types of animals<br>3 No, these utensils are used for other, non-food related purposes<br>4 No, these utensils are used for many purposes including other food preparation practices
693	ao_house_droppings_pres	Section Header: *Waste Management*<br>Prior to asking questions, please assess the yard/ground around the household and homestead for presence of any animal waste/droppings. Indicate presence:	radio<br>1 Clean<br>2 Some animal droppings<br>3 Extensive presence of animal droppings
694	note10	Explain to the participant: I am now going to ask you questions relating to your household's waste management. Please let me know if you need me to clarify any of my questions. Feel free to ask any question you may have.	descriptive<br>Custom alignment: LV
695	ao_waste_collect	Do you collect your animal waste/feces?	radio<br>1 Yes<br>0 No<br>99 Don't know/no answer<br>Custom alignment: LV
696	ao_waste_collect_animals<br>Show the field ONLY if:<br>[ao_waste_collect] = '1'	From which animals do you collect?	checkbox<br>1 ao_waste_collect_animals___1 Cattle<br>2 ao_waste_collect_animals___2 Donkey<br>3 ao_waste_collect_animals___3 Goat<br>4 ao_waste_collect_animals___4 Sheep<br>5 ao_waste_collect_animals___5 Chicken<br>6 ao_waste_collect_animals___6 Dog<br>7 ao_waste_collect_animals___7 Cat<br>Custom alignment: LV

Havelaar AH, *et al. BMJ Open* 2022; 12:e061311. doi: 10.1136/bmjopen-2022-061311

697	ao_waste_cattle<br><br>Show the field ONLY if:<br>[ao_waste_collect_animals(1)] = '1'	Cattle - how do you collect these feces?	radio<br>1 By hand<br>2 Brooms<br>3 Using cloth<br>4 Using rake, spade, hoe or other tool<br><br>Custom alignment: LV
698	ao_waste_cattle_reuse<br><br>Show the field ONLY if:<br>[ao_waste_collect_animals(1)] = '1'	Do you use the cattle feces/excrement collected?	radio<br>1 Yes<br>0 No<br>99 Don't know/no answer<br><br>Custom alignment: LV
699	ao_waste_cattle_reuse_how<br><br>Show the field ONLY if:<br>[ao_waste_cattle_reuse] = '1'	What do you do with the animal feces generated from your cattle?<br>*check all that apply*	checkbox<br>1 ao_waste_cattle_reuse_how___1 Use for cooking/fuel<br>2 ao_waste_cattle_reuse_how___2 Use for construction<br>3 ao_waste_cattle_reuse_how___3 Fertilizer/Distribute manure on fields, land, or crops<br>4 ao_waste_cattle_reuse_how___4 For cleaning floor and wall<br>5 ao_waste_cattle_reuse_how___5 As animal feed<br>6 ao_waste_cattle_reuse_how___6 Other household purposes<br>88 ao_waste_cattle_reuse_how___88 Other (comment below)<br><br>Custom alignment: LV
700	ao_waste_cattle_reuse_comment<br><br>Show the field ONLY if:<br>[ao_waste_cattle_reuse_how(88)] = '1'	Other comment regarding use of animal excrement	text<br>Custom alignment: LV
701	ao_waste_donkey<br><br>Show the field ONLY if:<br>[ao_waste_collect_animals(2)] = '1'	Donkey - how do you collect these feces?	radio<br>1 By hand<br>2 Brooms<br>3 Using cloth<br>4 Using rake, spade, hoe or other tool<br><br>Custom alignment: LV
702	ao_waste_donkey_reuse<br><br>Show the field ONLY if:<br>[ao_waste_collect_animals(2)] = '1'	Do you use the donkey feces/excrement collected?	radio<br>1 Yes<br>0 No<br>99 Don't know/no answer<br><br>Custom alignment: LV

703	ao_waste_donkey_reuse_how<br>Show the field ONLY if:<br>[ao_waste_donkey_reuse] = '1'	What do you do with the animal feces generated from your donkey?<br>*check all that apply*	checkbox<br>1 ao_waste_donkey_reuse_how___1 Use for cooking/fuel<br>2 ao_waste_donkey_reuse_how___2 Use for construction<br>3 ao_waste_donkey_reuse_how___3 Fertilizer/Distribu manure on fields land, or crops<br>4 ao_waste_donkey_reuse_how___4 For cleaning floo and wall<br>5 ao_waste_donkey_reuse_how___5 As animal feed<br>6 ao_waste_donkey_reuse_how___6 Other household purposes<br>88 ao_waste_donkey_reuse_how___88 Other (comment below)<br>Custom alignment: LV
704	ao_waste_donkey_reuse_comment<br>Show the field ONLY if:<br>[ao_waste_donkey_reuse_how(88)] = '1'	Other comment regarding use of animal excrement	text<br>Custom alignment: LV
705	ao_waste_goat<br>Show the field ONLY if:<br>[ao_waste_collect_animals(3)] = '1'	Goat - how do you collect these feces?	radio<br>1 By hand<br>2 Brooms<br>3 Using cloth<br>4 Using rake, spade, hoe or other tool<br>Custom alignment: LV
706	ao_waste_goat_reuse<br>Show the field ONLY if:<br>[ao_waste_collect_animals(3)] = '1'	Do you use the goat feces/excrement collected?	radio<br>1 Yes<br>0 No<br>99 Don't know/no answer<br>Custom alignment: LV
707	ao_waste_goat_reuse_how<br>Show the field ONLY if:<br>[ao_waste_goat_reuse] = '1'	What do you do with the animal feces generated from your goat?<br>*check all that apply*	checkbox<br>1 ao_waste_goat_reuse_how___1 Use for cooking/fuel<br>2 ao_waste_goat_reuse_how___2 Use for construction<br>3 ao_waste_goat_reuse_how___3 Fertilizer/Distribute manure on fields, land, or crops<br>4 ao_waste_goat_reuse_how___4 For cleaning floor and wall<br>5 ao_waste_goat_reuse_how___5 As animal feed<br>6 ao_waste_goat_reuse_how___6 Other household purposes<br>88 ao_waste_goat_reuse_how___88 Other (comment below)<br>Custom alignment: LV
708	ao_waste_goat_reuse_comment<br>Show the field ONLY if:<br>[ao_waste_goat_reuse_how(88)] = '1'	Other comment regarding use of animal excrement	text<br>Custom alignment: LV

709	ao_waste_sheep<br>Show the field ONLY if:<br>[ao_waste_collect_animals(4)] = '1'	Sheep - how do you collect these feces?	radio<br>1 By hand<br>2 Brooms<br>3 Using cloth<br>4 Using rake, spade, hoe or other tool<br>Custom alignment: LV
710	ao_waste_sheep_reuse<br>Show the field ONLY if:<br>[ao_waste_collect_animals(4)] = '1'	Do you use the sheep feces/excrement collected?	radio<br>1 Yes<br>0 No<br>99 Don't know/no answer<br>Custom alignment: LV
711	ao_waste_sheep_reuse_how<br>Show the field ONLY if:<br>[ao_waste_sheep_reuse] = '1'	What do you do with the animal feces generated from your sheep?<br>*check all that apply*	checkbox<br>1 ao_waste_sheep_reuse_how___1 Use for cooking/fuel<br>2 ao_waste_sheep_reuse_how___2 Use for construction<br>3 ao_waste_sheep_reuse_how___3 Fertilizer/Distribut manure on fields, land, or crops<br>4 ao_waste_sheep_reuse_how___4 For cleaning floor and wall<br>5 ao_waste_sheep_reuse_how___5 As animal feed<br>6 ao_waste_sheep_reuse_how___6 Other household purposes<br>88 ao_waste_sheep_reuse_how___88 Other (comment below)<br>Custom alignment: LV
712	ao_waste_sheep_reuse_comment<br>Show the field ONLY if:<br>[ao_waste_sheep_reuse_how(88)] = '1'	Other comment regarding use of animal excrement	text<br>Custom alignment: LV
713	ao_waste_chicken<br>Show the field ONLY if:<br>[ao_waste_collect_animals(5)] = '1'	Chicken - how do you collect these feces?	radio<br>1 By hand<br>2 Brooms<br>3 Using cloth<br>4 Using rake, spade, hoe or other tool<br>Custom alignment: LV
714	ao_waste_chicken_reuse<br>Show the field ONLY if:<br>[ao_waste_collect_animals(5)] = '1'	Do you use the chicken feces/excrement collected?	radio<br>1 Yes<br>0 No<br>99 Don't know/no answer<br>Custom alignment: LV

715	ao_waste_chicken_reuse_how<br>Show the field ONLY if: [ao_waste_chicken_reuse] = '1'	What do you do with the animal feces generated from your chicken?<br>*check all that apply*	checkbox<br>1 ao_waste_chicken_reuse_how___1 Use for cooking/fuel<br>2 ao_waste_chicken_reuse_how___2 Use for construction<br>3 ao_waste_chicken_reuse_how___3 Fertilizer/Distribu manure on fields land, or crops<br>4 ao_waste_chicken_reuse_how___4 For cleaning floo and wall<br>5 ao_waste_chicken_reuse_how___5 As animal feed<br>6 ao_waste_chicken_reuse_how___6 Other householc purposes<br>88 ao_waste_chicken_reuse_how___88 Other (comment below)<br>Custom alignment: LV
716	ao_waste_chicken_reuse_comment<br>Show the field ONLY if: [ao_waste_chicken_reuse_how(88)] = '1'	Other comment regarding use of animal excrement	text<br>Custom alignment: LV
717	ao_waste_dog<br>Show the field ONLY if: [ao_waste_collect_animals(6)] = '1'	Dog - how do you collect these feces?	radio<br>1 By hand<br>2 Brooms<br>3 Using cloth<br>4 Using rake, spade, hoe or other tool<br>Custom alignment: LV
718	ao_waste_dog_reuse<br>Show the field ONLY if: [ao_waste_collect_animals(6)] = '1'	Do you use the dog feces/excrement collected?	radio<br>1 Yes<br>0 No<br>99 Don't know/no answer<br>Custom alignment: LV
719	ao_waste_dog_reuse_how<br>Show the field ONLY if: [ao_waste_dog_reuse] = '1'	What do you do with the animal feces generated from your dog?<br>*check all that apply*	checkbox<br>1 ao_waste_dog_reuse_how___1 Use for cooking/fuel<br>2 ao_waste_dog_reuse_how___2 Use for construction<br>3 ao_waste_dog_reuse_how___3 Fertilizer/Distribute manure on fields, land, or crops<br>4 ao_waste_dog_reuse_how___4 For cleaning floor and wall<br>5 ao_waste_dog_reuse_how___5 As animal feed<br>6 ao_waste_dog_reuse_how___6 Other household purposes<br>88 ao_waste_dog_reuse_how___88 Other (comment below)<br>Custom alignment: LV
720	ao_waste_dog_reuse_comment<br>Show the field ONLY if: [ao_waste_dog_reuse_how(88)] = '1'	Other comment regarding use of animal excrement	text<br>Custom alignment: LV

721	ao_waste_cat<br><br>Show the field ONLY if:<br>[ao_waste_collect_animals(7)] = '1'	Cat - how do you collect these feces?	radio<br>1 By hand<br>2 Brooms<br>3 Using cloth<br>4 Using rake, spade, hoe or other tool<br><br>Custom alignment: LV
722	ao_waste_cat_reuse<br><br>Show the field ONLY if:<br>[ao_waste_collect_animals(7)] = '1'	Do you use the cat feces/excrement collected?	radio<br>1 Yes<br>0 No<br>99 Don't know/no answer<br><br>Custom alignment: LV
723	ao_waste_cat_reuse_how<br><br>Show the field ONLY if:<br>[ao_waste_cat_reuse] = '1'	What do you do with the animal feces generated from your cat?<br>*check all that apply*	checkbox<br>1 ao_waste_cat_reuse_how___1 Use for cooking/fuel<br>2 ao_waste_cat_reuse_how___2 Use for construction<br>3 ao_waste_cat_reuse_how___3 Fertilizer/Distribute manure on fields, land, or crops<br>4 ao_waste_cat_reuse_how___4 For cleaning floor and wall<br>5 ao_waste_cat_reuse_how___5 As animal feed<br>6 ao_waste_cat_reuse_how___6 Other household purposes<br>88 ao_waste_cat_reuse_how___88 Other (comment below)<br><br>Custom alignment: LV
724	ao_waste_cat_reuse_comment<br><br>Show the field ONLY if:<br>[ao_waste_cat_reuse_how(88)] = '1'	Other comment regarding use of animal excrement	text<br>Custom alignment: LV
725	ao_waste_garbage	How is garbage produced by this house disposed of?	dropdown<br>1 Household dumps at will<br>2 Burned<br>3 Used as green manure<br>4 Buried<br>5 Periodically collected from household<br>6 Periodically collected from specified dumping points<br>99 Don't know
726	animal_husbandry_male_only_complete	Section Header: *Form Status*<br>Complete?	dropdown<br>0 Incomplete<br>1 Unverified<br>2 Complete
Instrument: **Human WASH Practices (ALL)** (human_wash_practices_all)			Collapse
727	wh_m_wash_hands	WH1) Do you wash your hands after going to the toilet/latrine?	yesno<br>1 Yes<br>0 No<br><br>Custom alignment: LV

728	wh_m_hand_wash_often<br>Show the field ONLY if:<br>[wh_m_wash_hands] = '1'	WH1a) How often?	radio<br>1 Sometimes<br>2 Often<br>3 Everytime<br>Custom alignment: LV
729	wh_m_hand_wash_nappy	WH2) Do you wash your hands after cleaning the baby's bottom/changing a baby's nappy	radio<br>1 Yes<br>0 No<br>99 Not applicable<br>Custom alignment: LV
730	wh_m_hand_wash_nappy_often<br>Show the field ONLY if:<br>[wh_m_hand_wash_nappy] = '1'	WH2a) How often?	radio<br>1 Sometimes<br>2 Often<br>3 Everytime<br>Custom alignment: LV
731	wh_m_wash_hands_food	WH3) Do you wash your hands before preparing/handling food?	radio<br>1 Yes<br>0 No<br>99 Not applicable<br>Custom alignment: LV
732	wh_m_wash_hands_food_often<br>Show the field ONLY if:<br>[wh_m_wash_hands_food] = '1'	WH3a) How often?	radio<br>1 Sometimes<br>2 Often<br>3 Everytime<br>Custom alignment: LV
733	wh_m_wash_hands_feeding	WH4) Do you wash your hands before feeding a child/eating?	radio<br>1 Yes<br>0 No<br>99 Not applicable<br>Custom alignment: LV
734	wh_m_wash_hands_feeding_often<br>Show the field ONLY if:<br>[wh_m_wash_hands_feeding] = '1'	WH4a) How often?	radio<br>1 Sometimes<br>2 Often<br>3 Everytime<br>Custom alignment: LV
735	wh_m_wash_hands_raw	WH5) Do you wash your hands after handling raw food?	radio<br>1 Yes<br>0 No<br>99 Not applicable<br>Custom alignment: LV
736	wh_m_wash_hands_raw_often<br>Show the field ONLY if:<br>[wh_m_wash_hands_raw] = '1'	WH5a) How often?	radio<br>1 Sometimes<br>2 Often<br>3 Everytime<br>Custom alignment: LV

737	wh_m_wash_hands_garbage	WH6) Do you wash your hands after handling garbage?	radio<br>1 Yes<br>0 No<br>99 Not applicable<br><br>Custom alignment: LV
738	wh_m_wash_hands_garbage_often<br><br>Show the field ONLY if:<br>[wh_m_wash_hands_garbage] = '1'	WH6a) How often?	radio<br>1 Sometimes<br>2 Often<br>3 Everytime<br><br>Custom alignment: LV
739	wh_m_wash_hands_field	WH7) Do you wash your hands after field work?	radio<br>1 Yes<br>0 No<br>99 Not applicable<br><br>Custom alignment: LV
740	wh_m_wash_hands_field_often<br><br>Show the field ONLY if:<br>[wh_m_wash_hands_field] = '1'	WH7a) How often?	radio<br>1 Sometimes<br>2 Often<br>3 Everytime<br><br>Custom alignment: LV
741	wh_m_wash_hands_chick	Do you wash your hands after handling livestock, such as milking cattle, managing small stock, or moving chickens?	yesno<br>1 Yes<br>0 No<br><br>Custom alignment: LV
742	wh_m_wash_hands_chick_often<br><br>Show the field ONLY if:<br>[wh_m_wash_hands_chick] = '1'	WH7a) How often?	radio<br>1 Sometimes<br>2 Often<br>3 Everytime<br><br>Custom alignment: LV
743	wa_m_wash_careful	WA81) Are there animals in particular with which you are more careful to wash your hands before/after handling?	yesno<br>1 Yes<br>0 No<br><br>Custom alignment: LV
744	wa_m_wash_careful_list<br><br>Show the field ONLY if:<br>[wa_m_wash_careful] = '1'	WA81a) Which ones?<br>*Check all that apply*	checkbox<br>1 wa_m_wash_careful_list___1 Cattle<br>2 wa_m_wash_careful_list___2 Donkey<br>3 wa_m_wash_careful_list___3 Goat<br>4 wa_m_wash_careful_list___4 Sheep<br>5 wa_m_wash_careful_list___5 Chicken<br>6 wa_m_wash_careful_list___6 Dog<br>7 wa_m_wash_careful_list___7 Cat<br><br>Custom alignment: LV
745	wa_m_wash_caeful_why<br><br>Show the field ONLY if:<br>[wa_m_wash_careful] = '1'	WA81b) Why are you more careful to wash your hands before or after touching/handling [animal species above]?	text<br>Custom alignment: LV

746	wh_m_wash_with	Section Header: *Handwashing technique and soap use*<br>WH8) What do you wash your hands with?<br>*"sand" under our study context belongs to this category.*	radio<br>1 Water<br>2 Soap and water<br>3 Ash/sand<br>4 Disinfectant gels<br>0 Nothing<br>Custom alignment: LV
747	wh_m_wash_soap	WH9) Have you used soap today or yesterday?	yesno<br>1 Yes<br>0 No<br>Custom alignment: LV
748	wh_m_wash_soap_for<br>Show the field ONLY if:<br>[wh_m_wash_soap] = '1'	WH10) What did you use soap for? (Multiple answers possible)<br>*Check all that apply*	checkbox<br>1 wh_m_wash_soap_for___1 Washing clothes<br>2 wh_m_wash_soap_for___2 Washing my body<br>3 wh_m_wash_soap_for___3 Washing child's bottom<br>4 wh_m_wash_soap_for___4 Washing child's hands<br>5 wh_m_wash_soap_for___5 Washing hands after defecating<br>6 wh_m_wash_soap_for___6 Washing hands after cleaning child<br>7 wh_m_wash_soap_for___7 Washing hands before feeding child<br>8 wh_m_wash_soap_for___8 Washing hands after feeding child<br>9 wh_m_wash_soap_for___9 Washing hands before preparing food<br>10 wh_m_wash_soap_for___10 Washing hands before eating<br>11 wh_m_wash_soap_for___11 Washing hands after eating<br>12 wh_m_wash_soap_for___12 Washing hands before going out<br>13 wh_m_wash_soap_for___13 Washing hands before receiving visitors<br>88 wh_m_wash_soap_for___88 Other (specify below)<br>Custom alignment: LV
749	wh_m_wash_soap_for_other<br>Show the field ONLY if:<br>[wh_m_wash_soap_for(88)] = '1'	WH10a) Specify Other	text<br>Custom alignment: LV
750	wh_m_child_defecated_clean	Section Header: *Child stool management*<br>WH15) The last time [month_1_arm_1][en_child_fname] defecated, did you clean [month_1_arm_1][en_child_fname] ?	radio<br>1 Yes<br>0 No<br>99 Not applicable<br>Custom alignment: LV

Supplemental material BMJ Publishing Group Limited (BMJ) disclaims all liability and responsibility arising from any reliance placed on this supplemental material which has been supplied by the author(s) BMJ Open

751	wh_m_child_wash_after<br>Show the field ONLY if: [wh_m_child_defecated_clean] = '1'	WH15a) Did you wash your hands afterwards?	yesno<br>1 Yes<br>0 No<br>Custom alignment: LV
752	wh_m_child_wash_how<br>Show the field ONLY if: [wh_m_child_defecated_clean] = '1'	WH15b) How did you clean [month_1_arm_1][en_child_fname] ?	radio<br>1 Water only<br>2 Water and soap<br>3 Rags/leaves/paper/ash<br>4 Tissue/toilet paper<br>5 Hands only<br>0 None<br>88 Other (specify below)<br>Custom alignment: LV
753	wh_m_child_wash_how_other<br>Show the field ONLY if: [wh_m_child_wash_how] = '88'	WH16c) Specify Other	text<br>Custom alignment: LV
754	wh_m_child_another<br>Show the field ONLY if: [wh_m_child_wash_after] ='0'	WH17) Did another adult clean [month_1_arm_1][en_child_fname] ?	radio<br>1 Yes<br>0 No<br>99 Don't know/no answer<br>Custom alignment: LV
755	wh_m_child_dispose	WH19) The last time [month_1_arm_1][en_child_fname] defecated, what was done to dispose of the stools?	radio<br>1 Child used toilet/latrine<br>2 Put/rinsed into toilet or latrine<br>3 Put/rinsed into drain or ditch<br>4 Thrown into garbage<br>5 Buried<br>6 Left in the open<br>Custom alignment: LV
756	human_wash_practices_all_complete	Section Header: *Form Status*<br>Complete?	dropdown<br>0 Incomplete<br>1 Unverified<br>2 Complete
Instrument: **Child Nutrition (Female Only)** (child_nutrition_female_only)			Collapse
757	nh_c_script	Section Header: *(Female) Child Nutrition*<br>Now I would like to ask you about liquids or foods that your child had yesterday during the day or at night. I am interested in whether they consumed the item even if it was combined with other foods.	descriptive
758	nh_c_water	Section Header: *In past 24 hours has your child had any of these liquids?*<br>Water-	radio, Required<br>1 Yes<br>0 No<br>99 Don't know
759	nh_c_water_freq<br>Show the field ONLY if: [nh_c_water] = '1'	If yes, How many times in the past 24 hours?	text (number, Min: 1), Required

Havelaar AH, *et al. BMJ Open* 2022; 12:e061311. doi: 10.1136/bmjopen-2022-061311

760	nh_c_water_sugar<br>Show the field ONLY if:<br>[nh_c_water] = '1'	If yes, Was it mixed with sugar	radio, Required<br>1 Yes<br>0 No<br>99 Don't know
761	nh_c_formula	Infant Formula such as (enfamil)?	radio, Required<br>1 Yes<br>0 No<br>99 Don't know
762	nh_c_formula_type<br>Show the field ONLY if:<br>[nh_c_formula] = '1'	If yes, What type? (specify____________)	text (number, Min: 1, Max: 7), Required
763	nh_c_formula_freq<br>Show the field ONLY if:<br>[nh_c_formula] = '1'	If yes, How many times in the past 24 hours? (enter number)	text (number, Min: 1, Max: 7), Required
764	nh_c_milk	Milk, such as tinned, powdered, or fresh animal milk?	radio, Required<br>1 Yes<br>0 No<br>99 Don't know
765	nh_c_milk_type<br>Show the field ONLY if:<br>[nh_c_milk] = '1'	What type?	radio, Required<br>1 Tinned<br>2 Powdered<br>3 Animal milk
766	nh_c_milk_animal<br>Show the field ONLY if:<br>[nh_c_milk_type]= '3'	What type of animal milk?	radio, Required<br>1 Cow<br>2 Goat<br>3 Camel<br>4 Sheep
767	nh_c_milk_animal_past<br>Show the field ONLY if:<br>[nh_c_milk_type]= '3'	Do you pasteurize that milk?	radio, Required<br>1 Yes<br>0 No<br>99 Don't know
768	nh_c_milk_quan<br>Show the field ONLY if:<br>[nh_c_milk] = '1'	If yes, How many times in the past 24 hours have you given the child any type of milk?	text (number), Required
769	nh_c_juice	Juice or juice drinks?	radio, Required<br>1 Yes<br>0 No<br>99 Don't know
770	nh_c_clearbroth	Clear broth?	radio, Required<br>1 Yes<br>0 No<br>99 Don't know
771	nh_c_yogurt	Yogurt?	radio, Required<br>1 Yes<br>0 No<br>99 Don't know
772	nh_c_yogurt_freq<br>Show the field ONLY if:<br>[nh_c_yogurt] = '1'	If yes, how many times?	text, Required

773	nh_c_porridge	Thin porridge?	radio, Required<br>1 Yes<br>0 No<br>99 Don't know
774	nh_c_liquids	Any other liquids?	radio, Required<br>1 Yes<br>0 No<br>99 Don't know
775	nh_c_liquids_y<br>Show the field ONLY if:<br>[nh_c_liquids] = '1'	If yes, how many times?	text, Required
776	nh_ate_yest_script	Please describe everything that [month_1_arm_1][en_child_fname] ate yesterday during the day or night, whether at home or outside the home. a) Think about when [month_1_arm_1][en_child_fname] first woke up yesterday. Did [child NAME here] eat anything at that time? If yes: Please tell me everything [month_1_arm_1][en_child_fname]] ate at that time. Probe: Anything else? Until respondent says nothing else. If no, continue to Question b). b) What did [month_1_arm_1][en_child_fname] do after that? Did [month_1_arm_1][en_child_fname] eat anything at that time? If yes: Please tell me everything [month_1_arm_1][en_child_fname] ate at that time. Probe: Anything else? Until respondent says nothing else. Repeat question b) above until respondent says the child went to sleep until the next day. If respondent mentions mixed dishes like a PORRIDGE, sauce or stew, probe: c) What ingredients were in that (MIXEDDISH)? Probe: Anything else? Until respondent says nothing else. As the respondent recalls foods, please indicate the food group associated with the response. If the food is not listed in any of the food groups below, write the food in the box labeled 'other foods'. If foods are used in small amounts for seasoning or as a condiment, include them under the condiments food group.	descriptive

777	nh_ate_yest	Select all that apply:	checkbox, Required<br>1 | nh_ate_yest___1 | Food Group 1 - Injera, porridge, bread, rice, noodles, or other foods made from grains.<br>2 | nh_ate_yest___2 | Food Group 2 - Pumpkin, carrots, squash, or sweet potatoes that are yellow or orange inside.<br>3 | nh_ate_yest___3 | Food Group 3 - White potatoes, white yams, manioc, cassava, or any other foods made from roots<br>4 | nh_ate_yest___4 | Food Group 4 - Any dark green leafy vegetables<br>5 | nh_ate_yest___5 | Food Group 5 - Ripe mangoes, ripe papayas, or (other local vitamin A-rich fruits)<br>6 | nh_ate_yest___6 | Food Group 6 - Any other fruits or vegetables<br>7 | nh_ate_yest___7 | Food Group 7 - Liver, kidney, heart, or other organ meats<br>8 | nh_ate_yest___8 | Food Group 8 - Any meat, such as beef, pork, lamb, goat, chicken, or duck<br>9 | nh_ate_yest___9 | Food Group 9 - Eggs<br>10 | nh_ate_yest___10 | Food Group 10 - Fresh or dried fish, shellfish, or seafood<br>11 | nh_ate_yest___11 | Food Group 11 - Any foods made from beans, peas, or lentils?<br>12 | nh_ate_yest___12 | Food Group 12 - Any nuts or seeds?<br>13 | nh_ate_yest___13 | Food Group 13 - Cheese, yogurt, or other milk products<br>14 | nh_ate_yest___14 | Food Group 14 - Any oil, fats, or butter, or foods made with any of these<br>15 | nh_ate_yest___15 | Food Group 15 - Any sugary foods such as chocolates, sweets, candies, pastries, cakes, or biscuits<br>16 | nh_ate_yest___16 | Food Group 16 - Condiments for favor, such as chilies, spices, herbs, or fish powder<br>17 | nh_ate_yest___17 | Food Group 17 - Grubs, snails or insects<br>18 | nh_ate_yest___18 | Food Group 18 - Foods made with red palm oil, red palm nut, or red palm nut pulp sauce
778	nh_ate_yest_other	Other Foods (specify)	notes, Required
779	nh_no_selection_foods1<br>Show the field ONLY if:<br>[nh_ate_yest(1)] = '0'	Section Header: *Once the respondent finishes recalling foods eaten and data collector indicates on the list above, read each remaining food group NOT previously selected, and ask the following question:*<br>Yesterday during the day or night, did [month_1_arm_1] [en_child_fname] drink/eat any from food group 1?	dropdown, Required<br>1 | Yes<br>0 | No<br>99 | Don't know
780	nh_no_selection_foods2<br>Show the field ONLY if:<br>[nh_ate_yest(2)] = '0'	Yesterday during the day or night, did [month_1_arm_1] [en_child_fname] drink/eat any from food group 2?	dropdown, Required<br>1 | Yes<br>0 | No<br>99 | Don't know

781	nh_no_selection_foods3<br>Show the field ONLY if:<br>[nh_ate_yest(3)] = '0'	Yesterday during the day or night, did [month_1_arm_1][en_child_fname] drink/eat any from food group 3?	dropdown, Required<br>1 Yes<br>0 No<br>99 Don't know
782	nh_no_selection_foods4<br>Show the field ONLY if:<br>[nh_ate_yest(4)] = '0'	Yesterday during the day or night, did [month_1_arm_1][en_child_fname] drink/eat any from food group 4?	dropdown, Required<br>1 Yes<br>0 No<br>99 Don't know
783	nh_no_selection_foods5<br>Show the field ONLY if:<br>[nh_ate_yest(5)] = '0'	Yesterday during the day or night, did [month_1_arm_1][en_child_fname] drink/eat any from food group 5?	dropdown, Required<br>1 Yes<br>0 No<br>99 Don't know
784	nh_no_selection_foods6<br>Show the field ONLY if:<br>[nh_ate_yest(6)] = '0'	Yesterday during the day or night, did [month_1_arm_1][en_child_fname] drink/eat any from food group 6?	dropdown, Required<br>1 Yes<br>0 No<br>99 Don't know
785	nh_no_selection_foods7<br>Show the field ONLY if:<br>[nh_ate_yest(7)] = '0'	Yesterday during the day or night, did [month_1_arm_1][en_child_fname] drink/eat any from food group 7?	dropdown, Required<br>1 Yes<br>0 No<br>99 Don't know
786	nh_no_selection_foods8<br>Show the field ONLY if:<br>[nh_ate_yest(8)] = '0'	Yesterday during the day or night, did [month_1_arm_1][en_child_fname] drink/eat any from food group 8?	dropdown, Required<br>1 Yes<br>0 No<br>99 Don't know
787	nh_no_selection_foods9<br>Show the field ONLY if:<br>[nh_ate_yest(9)] = '0'	Yesterday during the day or night, did [month_1_arm_1][en_child_fname] drink/eat any from food group 9?	dropdown, Required<br>1 Yes<br>0 No<br>99 Don't know
788	nh_no_selection_foods10<br>Show the field ONLY if:<br>[nh_ate_yest(10)] = '0'	Yesterday during the day or night, did [month_1_arm_1][en_child_fname] drink/eat any from food group 10?	dropdown, Required<br>1 Yes<br>0 No<br>99 Don't know
789	nh_no_selection_foods11<br>Show the field ONLY if:<br>[nh_ate_yest(11)] = '0'	Yesterday during the day or night, did [month_1_arm_1][en_child_fname] drink/eat any from food group 11?	dropdown, Required<br>1 Yes<br>0 No<br>99 Don't know
790	nh_no_selection_foods12<br>Show the field ONLY if:<br>[nh_ate_yest(12)] = '0'	Yesterday during the day or night, did [month_1_arm_1][en_child_fname] drink/eat any from food group 12?	dropdown, Required<br>1 Yes<br>0 No<br>99 Don't know
791	nh_no_selection_foods13<br>Show the field ONLY if:<br>[nh_ate_yest(13)] = '0'	Yesterday during the day or night, did [month_1_arm_1][en_child_fname] drink/eat any from food group 13?	dropdown, Required<br>1 Yes<br>0 No<br>99 Don't know
792	nh_no_selection_foods14<br>Show the field ONLY if:<br>[nh_ate_yest(14)] = '0'	Yesterday during the day or night, did [month_1_arm_1][en_child_fname] drink/eat any from food group 14?	dropdown, Required<br>1 Yes<br>0 No<br>99 Don't know

793	nh_no_selection_foods15<br>Show the field ONLY if:<br>[nh_ate_yest(15)] = '0'	Yesterday during the day or night, did [month_1_arm_1] [en_child_fname] drink/eat any from food group 15?	dropdown, Required<br>1 Yes<br>0 No<br>99 Don't know
794	nh_no_selection_foods16<br>Show the field ONLY if:<br>[nh_ate_yest(16)] = '0'	Yesterday during the day or night, did [month_1_arm_1] [en_child_fname] drink/eat any from food group 16?	dropdown, Required<br>1 Yes<br>0 No<br>99 Don't know
795	nh_no_selection_foods17<br>Show the field ONLY if:<br>[nh_ate_yest(17)] = '0'	Yesterday during the day or night, did [month_1_arm_1] [en_child_fname] drink/eat any from food group 17?	dropdown, Required<br>1 Yes<br>0 No<br>99 Don't know
796	nh_no_selection_foods18<br>Show the field ONLY if:<br>[nh_ate_yest(18)] = '0'	Yesterday during the day or night, did [month_1_arm_1] [en_child_fname] drink/eat any from food group 18?	dropdown, Required<br>1 Yes<br>0 No<br>99 Don't know
797	child_nutrition_female_only_complete	Section Header: *Form Status*<br>Complete?	dropdown<br>0 Incomplete<br>1 Unverified<br>2 Complete
Instrument: **Household WASH (Female Only)** (household_wash_female_only)			Collapse
798	ws_main_source	Section Header: *(Female) Household WASH - Explain to the participant: I am now going to ask you questions relating to your household's water and sanitation. Please let me know if you need me to clarify any of my questions. Feel free to ask any question you may have.*<br>WS1) What is the main source of drinking water for members of your household (choose one).	dropdown, Required<br>1 Piped<br>2 Dug well<br>3 Water from spring<br>4 Rainwater collection<br>5 Delivered water<br>6 Water kiosk<br>7 Packaged water<br>8 Surface water (river, stream, dam, lake, pond, canal, irrigation channel)<br>9 Boreholes or Tubewells<br>88 Other (specify below)<br>99 Don't know/No answer
799	ws_main_source_other<br>Show the field ONLY if:<br>[ws_main_source] = '88'	WS1a) Specify Other	text, Required<br>Custom alignment: LV
800	ws_piped<br>Show the field ONLY if:<br>[ws_main_source] = '1'	WS2) Piped - Describe how you access piped water.	radio, Required<br>1 Piped water into dwelling<br>2 Piped water into yard/plot<br>3 Public tap/standpipe<br>Custom alignment: LV
801	ws_dug_well<br>Show the field ONLY if:<br>[ws_main_source] = '2'	WS3) Dug well - Is the dug well protected or unprotected?	radio, Required<br>1 Protected well<br>2 Unprotected well<br>Custom alignment: LV

Supplemental material
BMJ Publishing Group Limited (BMJ) disclaims all liability and responsibility arising from any reliance placed on this supplemental material which has been supplied by the author(s)
BMJ Open

802	ws_spring<br>Show the field ONLY if:<br>[ws_main_source] = '3'	WS4) Water from spring - Is the spring protected or unprotected?	radio, Required<br>1 Protected spring<br>2 Unprotected spring<br>Custom alignment: LV
803	ws_rainws<br>Show the field ONLY if:<br>[ws_main_source] = '4'	WS5) Rainwater - How is your rainwater collected?	radio, Required<br>1 Collected in tank<br>2 Collected as a pond<br>Custom alignment: LV
804	ws_delivered<br>Show the field ONLY if:<br>[ws_main_source] = '5'	WS6) From where is your water delivered?	radio, Required<br>1 Taker-truck<br>2 Cart with small tank/drum<br>Custom alignment: LV
805	ws_packaging<br>Show the field ONLY if:<br>[ws_main_source] = '7'	WS7)From what kind of packaging does your water come?	radio, Required<br>1 Bottled water<br>2 Satchet water
806	ws_surface<br>Show the field ONLY if:<br>[ws_main_source] = '8'	WS8) Surface water - Which surface water do you use?	radio, Required<br>1 River<br>2 Stream<br>3 Dam<br>4 Lake<br>5 Pond<br>6 Canal<br>7 Irrigation Canal<br>Custom alignment: LV
807	ws_access	WS7) Where do you access this drinking water?	radio, Required<br>1 In own dwelling<br>2 In own yard/plot<br>3 Elsewhere<br>Custom alignment: LV
808	ws_water_travel	WS8) How long does it take to go there, get water, and come back?<br>*Note: Record the total time taken for a single round trip including queuing.*	radio, Required<br>1 Household members do not collect drinking water<br>2 Number of minutes<br>99 Do not know<br>Custom alignment: LV
809	ws_water_travel_min<br>Show the field ONLY if:<br>[ws_water_travel] = '2'	How many minutes does it take to get water?	text (number, Min: 1), Required<br>Custom alignment: LV
810	ws_water_insuffice	In the last month, has there been any time when your household did not have sufficient quantities of drinking water when needed?	radio, Required<br>1 Yes, at least once<br>0 No, always sufficient<br>99 Don't know<br>Custom alignment: LV
811	ws_access_other	WS9) Where do you access water for purposes other than drinking, such as cooking or hand-washing?	radio, Required<br>1 In own dwelling<br>2 In own yard/plot<br>3 Elsewhere<br>Custom alignment: LV

Havelaar AH, *et al. BMJ Open* 2022; 12:e061311. doi: 10.1136/bmjopen-2022-061311

812	ws_access_other_distance	WS10) How long does it take to go there, get water, and come back?<br>*Note: Record the total time taken for a single round trip including queuing.*	radio, Required<br>1 Household members do not collect drinking water<br>2 Number of minutes<br>99 Do not know<br>Custom alignment: LV
813	ws_fetch_water	Who usually goes to this source to fetch the water for your household?	radio, Required<br>1 Adult woman<br>2 Adult man<br>3 Female child (under 15 years)<br>4 Male child (under 15 years)<br>99 Do not know<br>Custom alignment: LV
814	ws_safer	WS11) Do you do anything to the water to make it safer to drink?	radio, Required<br>1 Yes<br>0 No<br>99 Don't know/No Answer<br>Custom alignment: LV
815	ws_safer_method<br>Show the field ONLY if: [ws_safer] = '1'	WS12) What do you usually do to make the water safer to drink?<br>*Check all that apply*	checkbox, Required<br>1 ws_safer_method___1 Boil<br>2 ws_safer_method___2 Add bleach/chlorine/water guard/pur/bishan gari/aqua tabs<br>3 ws_safer_method___3 Strain through a cloth<br>4 ws_safer_method___4 Bio sand/composite/ceramic pot filer<br>5 ws_safer_method___5 Solar disinfection<br>6 ws_safer_method___6 Let it stand and settle<br>88 ws_safer_method___88 Other (specify below)<br>99 ws_safer_method___99 Don't know<br>Custom alignment: LV
816	ws_safer_method_other<br>Show the field ONLY if: [ws_safer_method(88)] = '1'	WS12a) Specify Other	text, Required<br>Custom alignment: LV
817	ws_safer_timeframe	WS13) When was the last time your household treated the water using this method?	radio, Required<br>1 Today<br>2 Yesterday<br>3 Less than one week ago<br>4 Less than one month ago<br>5 More than one month ago<br>99 Don't know<br>Custom alignment: LV
818	ws_storage	WS14) Does your household have a water storage container for drinking water?	radio, Required<br>1 Yes<br>0 No<br>99 Don't know/No Answer<br>Custom alignment: LV

Havelaar AH, *et al. BMJ Open* 2022; 12:e061311. doi: 10.1136/bmjopen-2022-061311

819	ws_drinkerwater_container<br>Show the field ONLY if:<br>[ws_storage] = '1'	What type of container (choose the one used for drinking water)?	checkbox, Required<br>1 ws_drinkerwater_container___1 Jerrycan<br>2 ws_drinkerwater_container___2 Bucket<br>3 ws_drinkerwater_container___3 Basin<br>4 ws_drinkerwater_container___4 Bottle<br>5 ws_drinkerwater_container___5 Saucepan<br>6 ws_drinkerwater_container___6 Drums<br>88 ws_drinkerwater_container___88 Other<br>Custom alignment: LV
820	ws_other_container<br>Show the field ONLY if:<br>[ws_drinkerwater_container(88)] = '1'	Specify other container	text, Required<br>Custom alignment: LV
821	ws_container_made<br>Show the field ONLY if:<br>[ws_storage] = '1'	What is this container made out of?	radio, Required<br>1 Plastic<br>2 Metal (aluminum, iron)<br>3 Earthenware (mud, wood)<br>4 Cement<br>88 Other<br>Custom alignment: LV
822	ws_other_container_made<br>Show the field ONLY if:<br>[ws_container_made] = '88'	Specify what the other container is made out of	text, Required<br>Custom alignment: LV
823	ws_container_cover<br>Show the field ONLY if:<br>[ws_storage] = '1'	Is this container covered?	radio, Required<br>1 Yes<br>0 No<br>99 Don't know<br>Custom alignment: LV
824	ws_storage_removal	WS15) How do you remove water from your drinking water storage container?<br>*Check all that apply*	checkbox, Required<br>1 ws_storage_removal___1 Tap<br>2 ws_storage_removal___2 Pitcher<br>3 ws_storage_removal___3 Cup/dipper/ladle/scoop<br>4 ws_storage_removal___4 Pour from the container<br>5 ws_storage_removal___5 With hands<br>6 ws_storage_removal___6 With bottle<br>88 ws_storage_removal___88 Other (specify below)<br>99 ws_storage_removal___99 Don't know/no answer<br>Custom alignment: LV
825	ws_storage_remove_other<br>Show the field ONLY if:<br>[ws_storage_removal(88)] = '1'	WS15a) Specify Other	text, Required<br>Custom alignment: LV

Havelaar AH, *et al. BMJ Open* 2022; 12:e061311. doi: 10.1136/bmjopen-2022-061311

826	ws_retrieved	WS16) How many times per day is water retrieved?	radio, Required<br>0 0<br>1 1<br>2 2<br>3 3<br>4 4<br>5 5<br>6 More<br>Custom alignment: LV
827	ws_latrine_day	Section Header: *Sanitation*<br>WS19) What kind of toilet facility do members of your household who are in and around the homestead during the day usually use?	radio, Required<br>1 Flush or pour toilet<br>2 Pit latrine<br>3 Composting toilet<br>4 Bucket toilet<br>5 Hanging toilet/latrine<br>6 They typically do not use a facility: Bush/Field/open defecation<br>7 Septic tank<br>88 Other (specify below)<br>Custom alignment: LV
828	ws_od_where<br>Show the field ONLY if: [ws_latrine_day] = '1' or [ws_latrine_day] = '2' or [ws_latrine_day] = '3' or [ws_latrine_day] = '4' or [ws_latrine_day] = '5' or [ws_latrine_day] = '88'	Where is this toilet facility located?	radio, Required<br>1 In own dwelling/house<br>2 In own yard/plot/homestead<br>3 Elsewhere<br>Custom alignment: LV
829	ws_latrine_day_bury_feces<br>Show the field ONLY if: [ws_latrine_day] = '6'	WS19b) If so, do household members typically bury their feces?	yesno, Required<br>1 Yes<br>0 No<br>Custom alignment: LV
830	ws_latrine_day_other<br>Show the field ONLY if: [ws_latrine_day] = '88'	WS19c) Specify Other	text, Required<br>Custom alignment: LV
831	ws_latrine_flush<br>Show the field ONLY if: [ws_latrine_day] = '1'	WS20) To where does this flush or pour toilet discharge?	radio, Required<br>1 Flush to piped sewer system<br>2 Flush to septic tank<br>3 Flush to pit latrine<br>4 Flush to somewhere else<br>5 Flush to don't know where<br>Custom alignment: LV
832	ws_latrine_pit<br>Show the field ONLY if: [ws_latrine_day] = '2'	WS21) What type of pit latrine is it?	radio, Required<br>1 Ventilated improved<br>2 Pit latrine with slab<br>3 Pit latrine without slab/open pit<br>Custom alignment: LV

Havelaar AH, *et al. BMJ Open* 2022; 12:e061311. doi: 10.1136/bmjopen-2022-061311

833	ws_latrine_pit_compost<br>Show the field ONLY if:<br>[ws_latrine_day] = '3'	WS21) What type of pit latrine is it?	radio, Required<br>1 Twin pit with slab<br>2 Twin pit without slab<br>3 Other composting toilet<br>Custom alignment: LV
834	ws_latrine_consistent<br>Show the field ONLY if:<br>[ws_latrine_day] = '1' or [ws_latrine_day] = '2' or [ws_latrine_day] = '3' or [ws_latrine_day] = '4' or [ws_latrine_day] = '5' or [ws_latrine_day] = '88'	WS22) Do people within the household consistently use the latrine for defecation?	yesno, Required<br>1 Yes<br>0 No<br>Custom alignment: LV
835	ws_latrine_consistent_not<br>Show the field ONLY if:<br>[ws_latrine_consistent] = '0'	WS23) If not, is it adults or children who do not consistently use latrine for defecation?	radio, Required<br>1 Adults<br>2 Children<br>3 Both<br>Custom alignment: LV
836	ws_latrine_no_households	WS18) Do you share this facility with others who are not members of your household?<br>*Including own household*	yesno, Required<br>1 Yes<br>0 No<br>Custom alignment: LV
837	ws_sani_facil_empty<br>Show the field ONLY if:<br>[ws_od_where] = '1' or [ws_od_where] = '2'	Has your on-site sanitation facility ever been emptied	radio, Required<br>1 Yes emptied<br>0 Never emptied<br>99 Don't know<br>Custom alignment: LV
838	ws_sani_empty_where<br>Show the field ONLY if:<br>[ws_sani_facil_empty] = '1'	The last time it was emptied, where were the contents emptied to?	radio, Required<br>1 Removed by service provider to a treatment plant<br>2 Removed by service provider and buried in a covered pit<br>3 Removed by service provider to don't know where<br>4 Emptied by household and buried in a covered pit<br>5 Emptied by household to uncovered pit, open ground, water body or elsewhere<br>88 Other (specify)<br>99 Don't know<br>Custom alignment: LV
839	ws_sani_empty_other<br>Show the field ONLY if:<br>[ws_sani_empty_where] = '88'	Other (describe)	text, Required<br>Custom alignment: LV
840	household_wash_female_only_complete	Section Header: *Form Status*<br>Complete?	dropdown<br>0 Incomplete<br>1 Unverified<br>2 Complete
Instrument: **Observational Wash Female** (observational_wash_female)			Collapse
841	wh_script	Section Header: *(Female) Observational WASH*<br>Please observe the mother and child for overall cleanliness. If the mother has clean hands, hair, clothes and face, she is classified as fully clean.	descriptive

842	wh_mother_cleanliness	How would you describe the mother?	dropdown, Required<br>1 Fully Clean<br>2 Not fully clean<br>99 I don't know
843	wh_child_cleanliness	How would you describe the child?	dropdown, Required<br>1 Fully Clean<br>2 Not fully clean<br>99 I don't know
844	wh_f_wash_most_where	WH11) Can you show me where you most often wash your hands?<br><br>(Ask to see and observe. Record only one hand washing place. This is the hand washing place that is used most often by the respondent or household.)	radio, Required<br>1 Fixed facility observed (sink/tap) within dwelling/house<br>2 Fixed facility observed (sink/tap) within yard/plot/homestead<br>3 Mobile object observed (tippy-tap/bucket/jug/kettle)<br>4 No handwashing place in dwelling/yard/plot<br>99 No permission to see<br>88 Other reason (specify)<br><br>Custom alignment: LV
845	wh_water_avail_script	Observe availability of water at the place for handwashing. Verify by checking the tap/pump, or basin, bucket, water container or similar objects for presence of water.<br><br>Note: Handwashing facilities may be fixed or mobile and include a sink with tap water, buckets with taps, tippy-taps, and jugs or basins designated for handwashing.<br><br>(If there is a tap or pump present at the specific place for hand washing, open the tap or operate the pump to see if water is coming out. If there is a bucket, basin, or other type of water container, examine it to see whether water is present in the container.)	descriptive
846	wh_water_avail_indication	Indicate if water is available.	radio, Required<br>1 Water is available<br>0 Water is not available
847	wh_soap_avail_script	Observe availability of soap or detergent at the place for handwashing.<br><br>Note: Soap includes bar soap, liquid soap, powder detergent, and soapy water but does not include ash, soil, sand or other handwashing agents.	descriptive
848	wh_soap_avail_indication	Indicate if soap is available.	radio, Required<br>1 Soap is available<br>0 Soap is not available
849	wh_defecate_script	Can you please show me where members of your household most often defecate?<br>(Ask to see and observe. Record only one space for typical defecation of household members. This is the place that is used most often by the respondent or household.)	descriptive
850	wh_defecate_indication	Indicate best description of latrine/place of defecation.	dropdown, Required<br>1 Intact structure (e.g. latrine)<br>2 Structure intact, but abandoned (with indication of non-use, such as being overgrown)<br>3 Structure not intact (e.g. cracked basin, walls caved in, etc.)<br>4 Area for open defecation indicated within the homestead<br>5 Area for open defecation indicated outside the homestead

851	observational_wash_female_complete	Section Header: *Form Status*<br>Complete?	dropdown<br>0 Incomplete<br>1 Unverified<br>2 Complete
Instrument: **Health Information (Female Only)** (health_information_female_only)			Collapse
852	health_intro	Section Header: *HEALTH INFORMATION*<br>Smoking, Alcohol, Chat Use	descriptive
853	hi_smoke	HI1) Do you currently smoke cigarettes?	yesno<br>1 Yes<br>0 No<br>Custom alignment: LV
854	hi_smoke_past_day<br>Show the field ONLY if:<br>[hi_smoke] = '1'	HI1a) If yes, in the last 24 hours, how many cigarettes did you smoke?	text (number)<br>Custom alignment: LV
855	hi_smoke_other_type	HI2) Do you currently smoke or use any other type of tobacco?	yesno<br>1 Yes<br>0 No<br>Custom alignment: LV
856	hi_smoke_other_types<br>Show the field ONLY if:<br>[hi_smoke_other_type] = '1'	HI2a) What other type of tobacco do you currently smoke or use?<br>*Check all that apply*	checkbox<br>1 hi_smoke_other_types___1 Pipe<br>2 hi_smoke_other_types___2 Chewing<br>3 hi_smoke_other_types___3 Snuff/Suret<br>4 hi_smoke_other_types___4 Shisha<br>5 hi_smoke_other_types___5 Gaya<br>88 hi_smoke_other_types___88 Other (Specify below)<br>Custom alignment: LV
857	hi_smoke_other_types_other<br>Show the field ONLY if:<br>[hi_smoke_other_types(88)] = '1'	HI2b) Specify Other	text<br>Custom alignment: LV
858	hi_smoke_ever	HI3) Have you ever smoked cigarette/shisha, etc...?	yesno<br>1 Yes<br>0 No<br>Custom alignment: LV
859	hi_smoke_ever_past_month<br>Show the field ONLY if:<br>[hi_smoke_ever] = '1'	HI3a) If yes, during the last 30 days, how many days did you smoke?	text (number, Min: 0, Max: 30)<br>Custom alignment: LV
860	hi_chat	HI4) Have you ever chewed chat?	yesno<br>1 Yes<br>0 No<br>Custom alignment: LV
861	hi_chat_past_month<br>Show the field ONLY if:<br>[hi_chat] = '1'	HI4a) If yes, during the last 30 days, how many days did you chew chat?	text (number, Min: 0, Max: 30)<br>Custom alignment: LV

Havelaar AH, *et al. BMJ Open* 2022; 12:e061311. doi: 10.1136/bmjopen-2022-061311

862	hi_chat_past_month_2<br>Show the field ONLY if:<br>[hi_chat] = '1'	HI4n) If yes, did you chew chat while you were pregnant with [month_1_arm_1][en_child_fname]?	radio<br>1 Yes<br>0 No<br>99 I don't know<br>Custom alignment: LV
863	hi_alcohol	HI5) Have you ever taken a drink that contains alcohol (Tella/Tegi/ Areke/Beer/Wine, etc...)?	yesno<br>1 Yes<br>0 No<br>Custom alignment: LV
864	hi_alcohol_past_month	HI5a) During the last 30 days, how many days did you take a drink that contains alcohol?	text (number, Min: 0, Max: 30)<br>Custom alignment: LV
865	hi_preg_antenatal	Section Header: *Pregnancy Health*<br>HI6) While pregnant with [month_1_arm_1][en_child_fname] Did you receive ante-natal care?	radio<br>1 Yes<br>0 No<br>99 I don't know<br>Custom alignment: LV
866	hi_preg_antenatal_1<br>Show the field ONLY if:<br>[hi_preg_antenatal] = '1'	Were you taught how to recognize when your baby is hungry?	radio<br>1 Yes<br>0 No<br>99 I don't know
867	hi_preg_antenatal_2<br>Show the field ONLY if:<br>[hi_preg_antenatal] = '1'	Were you encouraged to feed your babies often and for as long as the babies want?	radio<br>1 Yes<br>0 No<br>99 I don't know
868	hi_preg_antenatal_3<br>Show the field ONLY if:<br>[hi_preg_antenatal] = '1'	Were you informed to breastfeed if your breasts became overfull?	radio<br>1 Yes<br>0 No<br>99 I don't know
869	hi_preg_antenatal_4<br>Show the field ONLY if:<br>[hi_preg_antenatal] = '1'	Were you encouraged to breastfeed within one hour of birth?	radio<br>1 Yes<br>0 No<br>99 I don't know
870	hi_preg_antenatal_5<br>Show the field ONLY if:<br>[hi_preg_antenatal] = '1'	Were you shown how to breastfeed and maintain lactation?	radio<br>1 Yes<br>0 No<br>99 I don't know
871	hi_preg_antenatal_6<br>Show the field ONLY if:<br>[hi_preg_antenatal] = '1'	Did you receive information about the benefits of dietary diversity?	radio<br>1 Yes<br>0 No<br>99 I don't know
872	hi_preg_antenatal_7<br>Show the field ONLY if:<br>[hi_preg_antenatal] = '1'	Did you receive information about the benefits of animal source foods, such as eggs, milk, meat or fish?	radio<br>1 Yes<br>0 No<br>99 I don't know
873	hi_preg_antenatal_8<br>Show the field ONLY if:<br>[hi_preg_antenatal] = '1'	How many times during your pregnancy did you receive ante-natal care?	text (number)

Havelaar AH, *et al. BMJ Open* 2022; 12:e061311. doi: 10.1136/bmjopen-2022-061311

874	hi_preg_antenatal_9<br>Show the field ONLY if:<br>[hi_preg_antenatal] = '1'	When was your last ante-natal care visit, in relation to when [month_1_arm_1][en_child_fname] was born?	radio<br>1 Days before birth (enter number)<br>2 Weeks before birth (enter number)<br>3 Months before birth (enter number)<br>99 Do not know
875	hi_preg_antenatal_10<br>Show the field ONLY if:<br>[hi_preg_antenatal_9] = '1' or<br>[hi_preg_antenatal_9] = '2' or<br>[hi_preg_antenatal_9] = '3'	(Enter number) from question above	text
876	hi_preg_antenatal_11<br>Show the field ONLY if:<br>[hi_preg_antenatal] = '1'	Did distance/transportation to ante-natal care visits determine how often you went?	radio<br>1 Yes<br>0 No<br>99 I don't know
877	hi_preg_antenatal_12<br>Show the field ONLY if:<br>[hi_preg_antenatal] = '1'	Where did you receive ante-natal care?	radio<br>1 Government run hospitals<br>2 Private hospitals<br>3 Health clinic<br>4 Health post
878	hi_preg_iron	HI7) Did you take any iron tablets or iron syrup during this pregnancy?	radio<br>1 Yes<br>0 No<br>99 I don't know<br>Custom alignment: LV
879	hi_preg_worms	HI8) Did you take any drug for intestinal worms?	radio<br>1 Yes<br>0 No<br>99 I don't know<br>Custom alignment: LV
880	hi_preg_malaria	HI9) Did you take SP/Fansidar to keep you from getting malaria?	radio<br>1 Yes<br>0 No<br>99 I don't know<br>Custom alignment: LV
881	hi_preg_bednet	HI10) Did you sleep under the bed-net while you were pregnant?	radio<br>1 Yes<br>0 No<br>99 I don't know<br>Custom alignment: LV
882	hi_preg_diet	Did you change your diet in any way when you were pregnant?	radio<br>1 Yes<br>0 No<br>99 I don't know<br>Custom alignment: LV
883	hi_preg_diet_change<br>Show the field ONLY if:<br>[hi_preg_diet] = '1'	How did you change your diet?	text<br>Custom alignment: LV

884	hi_cd_pre_term	Section Header: *Child delivery*<br>Was the child delivered before term?	radio<br>1 Yes<br>0 No<br>99 I don't know
885	hi_cd_pre_term_y<br>Show the field ONLY if:<br>[hi_cd_pre_term] = '1'	If yes, Were you separated from the baby after birth?	radio<br>1 Yes<br>0 No<br>99 I don't know
886	hi_cd_delivered	Where was child delivered?	radio<br>1 At home<br>2 Facility<br>88 Other
887	hi_cd_delivered_home<br>Show the field ONLY if:<br>[hi_cd_delivered] = '1'	If at home: at home, was the birth attended by a skilled/trained birth attendant?	radio<br>1 Yes<br>0 No<br>99 I don't know
888	hi_cd_delivery_method	What was the child's mode of delivery?	radio<br>1 Caesarean<br>2 Vaginal
889	hi_breastfeed	Section Header: *Breastfeeding*<br>HI28) Did you breastfeed [month_1_arm_1][en_child_fname] at birth?	yesno, Required<br>1 Yes<br>0 No<br>Custom alignment: LV
890	hi_breastfeed_start	HI28a) How long after birth did you first put [month_1_arm_1][en_child_fname] to the breast?	radio, Required<br>1 Immediately<br>2 Hours after birth<br>3 Days after birth<br>Custom alignment: LV
891	hi_breastfeed_start_hour<br>Show the field ONLY if:<br>[hi_breastfeed_start] = '2'	HI28b) How many hours after birth?	text (number), Required<br>Custom alignment: LV
892	hi_breastfeed_start_day<br>Show the field ONLY if:<br>[hi_breastfeed_start] = '3'	HI28b) How many days after birth?	text (number), Required<br>Custom alignment: LV
893	hi_breastfeed_colustrum	After [month_1_arm_1][en_child_fname] was born, did you give the baby the first liquid (colostrum) that came from your breast?	radio, Required<br>1 Yes<br>0 No<br>99 I don't know
894	postdelivery_nonmilk	HI28c) In the first three days after delivery, was [month_1_arm_1][en_child_fname] given anything to drink other than breast milk to eat or drink? This might include animal milk, sugar water, or other traditional teas.	yesno, Required<br>1 Yes<br>0 No<br>Custom alignment: LV
895	hi_breastfeed_current	HI28d) Are you currently breastfeeding [month_1_arm_1][en_child_fname]?	yesno, Required<br>1 Yes<br>0 No<br>Custom alignment: LV

896	hi_breastfeed_longer<br>Show the field ONLY if:<br>[hi_breastfeed_current] = '1'	HI28e) For how much longer do you plan on breastfeeding your child?	radio, Required<br>1 For _____ number of months<br>2 Until the child is ______ number of months in age<br>3 Until I am pregnant with another child<br>4 Until the child indicates they no longer are interested<br>88 Other (specify below)<br>Custom alignment: LV
897	hi_bf_longer_num_mo<br>Show the field ONLY if:<br>[hi_breastfeed_longer] = '1'	Indicate - For _____ number of months	text (number), Required<br>Custom alignment: LV
898	hi_bf_longer_num_mo_c<br>Show the field ONLY if:<br>[hi_breastfeed_longer] = '1'	Indicate - Until the child is ______ number of months in age	text (number), Required<br>Custom alignment: LV
899	hi_breastfeed_no_stop<br>Show the field ONLY if:<br>[hi_breastfeed_current] = '0'	At what age did the child stop breastfeeding? (enter child age in number of months)	text (number), Required<br>Custom alignment: LV
900	breastfeed_length_wanted<br>Show the field ONLY if:<br>[hi_breastfeed_current] = '0'	Did you breastfeed for as long as you wanted to?	yesno, Required<br>1 Yes<br>0 No<br>Custom alignment: LV
901	hi_breastfeed_yesterday	HI28j) Was [month_1_arm_1][en_child_fname] breastfed yesterday during the day or at night?	radio, Required<br>1 Yes<br>0 No<br>99 I don't know<br>Custom alignment: LV
902	hi_breastfeed_quantity	Section Header: *Thinking about the past 7 days, please answer the following questions about how your child typically breast feeds:*<br>How many times each day (24 hours) did you typically breastfeed your child?	text (number), Required
903	hi_breastfeed_avg	How long does an average breast feeding last?	dropdown, Required<br>1 Less than 10 minutes<br>2 10-19 minutes<br>3 20-29 minutes<br>4 30-39 minutes<br>5 40-49 minutes<br>6 50 or more minutes
904	hi_breastfeed_24avg_hour	In an average 24-hour period, what is the longest time for you, the mother, between breast feedings? Please count the time from the start of one breastfeeding session to the next. HOURS	text, Required
905	hi_breastfeed_24avg_min	In an average 24-hour period, what is the longest time for you, the mother, between breast feedings? Please count the time from the start of one breastfeeding session to the next. MINUTES	text, Required
906	hi_breathfeed_other_method	Section Header: *Sometimes babies are fed breast milk in different ways, for example by spoon, cup or bottle. This can happen when the mother cannot always be with her baby. Sometimes babies are breastfed by another woman, or given breast milk from another woman by spoon, cup or bottle or some other way. This can happen if a mother cannot breastfeed her own baby.*<br>Did [month_1_arm_1][en_child_fname] consume breast milk in any of these ways yesterday during the day or at night?	radio, Required<br>1 Yes<br>0 No<br>99 I don't know
907	hi_breastfeed_food_liquid	HI29) Have you given [month_1_arm_1][en_child_fname] something to eat or drink other than breastmilk? This might include cow's milk, goat's milk, sugar water, or other liquids	radio, Required<br>1 Yes<br>0 No, not yet

908	hi_food_other_when<br>Show the field ONLY if:<br>[hi_breastfeed_food_liquid] = '1'	HI29) When did you first give [month_1_arm_1][en_child_fname] something to eat other than breastmilk? CHOOSE ONE	radio, Required<br>1 Child age in days (__________)<br>2 Child age in weeks (__________)<br>3 Child age in months (__________)<br>4 Number of days ago (__________)<br>5 Number of weeks ago (__________)<br>6 Number of months ago (__________)<br>Custom alignment: LV
909	hi_food_other_age_days<br>Show the field ONLY if:<br>[hi_breastfeed_food_liquid] = '1'	Section Header: *When did you first give [en_child_fname] something to eat other than breastmilk?*<br>HI29a) Child age in days	text (number), Required<br>Custom alignment: LV
910	hi_food_other_age_weeks<br>Show the field ONLY if:<br>[hi_breastfeed_food_liquid] = '1'	HI29a) Child age in weeks	text (number), Required<br>Custom alignment: LV
911	hi_food_other_age_months<br>Show the field ONLY if:<br>[hi_breastfeed_food_liquid] = '1'	HI29a) Child age in months	text (number), Required<br>Custom alignment: LV
912	hi_other_food_days<br>Show the field ONLY if:<br>[hi_breastfeed_food_liquid] = '1'	HI29a) Number of days ago	text (number), Required<br>Custom alignment: LV
913	hi_other_food_weeks<br>Show the field ONLY if:<br>[hi_breastfeed_food_liquid] = '1'	HI29a) Number of weeks ago	text (number), Required<br>Custom alignment: LV
914	hi_other_food_months<br>Show the field ONLY if:<br>[hi_breastfeed_food_liquid] = '1'	HI29a) Number of months ago	text (number), Required<br>Custom alignment: LV
915	hi_food_first<br>Show the field ONLY if:<br>[hi_breastfeed_food_liquid] = '1'	HI30) What food/drink was it that you first gave [month_1_arm_1][en_child_fname]?	text, Required<br>Custom alignment: LV
916	women_income_self	Section Header: *Women and Chat production*<br>Do you have income from any of your own activity?	radio<br>1 Yes<br>0 No<br>99 I don't know
917	women_income_self_activ<br>Show the field ONLY if:<br>[women_income_self] = '1'	If yes, what activities?	text
918	women_income_amount<br>Show the field ONLY if:<br>[women_income_self] = '1'	If yes, how much income in birr (per week)?	text (number)
919	women_income_use<br>Show the field ONLY if:<br>[women_income_self] = '1'	If yes, what do you do with that income?	radio<br>1 Hand it over to my husband<br>2 Becomes part of shared household income<br>3 I manage it and make decisions about how/when to spend<br>4 Part of it goes to my husband, and part of it is mine<br>Custom alignment: LV

920	chat_involved	Are you involved in chat production?	radio<br>1 Yes<br>0 No<br>99 Don't know
921	chat_own	Do you have your own chat, apart from your husband's fields?	radio<br>1 Yes<br>0 No<br>99 I don't know
922	chat_sell	Do you yourself sell chat at market?	radio<br>1 Yes<br>0 No<br>99 I don't know
923	chat_sell_often<br>Show the field ONLY if:<br>[chat_sell] = '1'	When your household is producing chat, how often are you at market for sale of chat?	radio<br>1 None, I don't travel to market (skip market questions)<br>2 Daily<br>3 2-4 times a week<br>4 Weekly<br>5 1-2 times a month
924	chat_income<br>Show the field ONLY if:<br>[chat_sell] = '1'	When you sell chat at market, what do you do with income generated?	radio<br>1 Give to husband<br>2 Buy food<br>3 Pay school fees<br>88 Other (specify)
925	chat_decisions<br>Show the field ONLY if:<br>[chat_sell] = '1'	Who typically makes decisions about what to do with money generated when you sell chat?	radio<br>1 I do<br>2 My husband does<br>3 My husband and I both do, together<br>88 Other (specify)
926	chat_travel_child<br>Show the field ONLY if:<br>[chat_sell] = '1'	When you travel to market, do you take [month_1_arm_1][en_child_fname] with you?	radio<br>1 Generally yes<br>0 Generally, no<br>2 Sometimes<br>99 I don't know
927	chat_watch_child<br>Show the field ONLY if:<br>[chat_travel_child] = '0'	Who keeps [month_1_arm_1][en_child_fname] if/when you leave him/her behind?	radio<br>1 Neighbor<br>2 Mother/mother in law/non-child relative<br>3 Older child<br>4 Husband
928	chat_fed_child<br>Show the field ONLY if:<br>[chat_sell] = '1'	What is [child name] fed, when you are at market?	text
929	chat_child_breastfed<br>Show the field ONLY if:<br>[chat_sell] = '1'	Is [month_1_arm_1][en_child_fname] breast fed by anyone else when you are at market?	radio<br>1 Yes<br>0 No<br>99 I don't know
930	health_information_female_only_complete	Section Header: *Form Status*<br>Complete?	dropdown<br>0 Incomplete<br>1 Unverified<br>2 Complete

Instrument: **A-WEAI (Female)** (aweai_female) Collapse

931	weai_inter_gender	1) What is the gender of the person being interviewed?	radio<br>1 Male<br>2 Female<br><br>Custom alignment: LV
932	weai_hh_type	2) TYPE OF HOUSEHOLD	radio<br>1 MALE AND FEMALE ADULT<br>2 FEMALE ADULT ONLY<br><br>Custom alignment: LV
933	weai_alone	3) ABILITY TO BE INTERVIEWED ALONE:	radio<br>1 ALONE<br>2 WITH ADULT FEMALES PRESENT<br>3 WITH ADULT MALES PRESENT<br>4 WITH ADULTS MIXED SEX PRESENT<br>5 WITH CHILDREN PRESENT<br>6 WITH ADULTS MIXED SEX AND CHILDREN PRESENT<br><br>Custom alignment: LV
934	weai_crop	Section Header: *"Now I'd like to ask you some questions about your participation in certain types of work activities and on making decisions on various aspects of household life"*<br>4) Did you yourself participate in Food crop farming: These are crops that are grown primarily for household food consumption in the past 12 months (that is, during the last [one/two] cropping seasons), from [PRESENT MONTH] last year to [PRESENT MONTH] this year?	radio<br>1 Yes<br>2 No<br><br>Custom alignment: LV
935	weai_crop_decisions<br><br>Show the field ONLY if:<br>[weai_crop] = '1'	4a) When decisions are made regarding Food crop farming, who is it that normally takes the decision?<br>*Check all that apply*	checkbox<br>1 weai_crop_decisions___1 SELF<br>2 weai_crop_decisions___2 SPOUSE<br>3 weai_crop_decisions___3 OTHER HH MEMBER<br>4 weai_crop_decisions___4 OTHER NON-HH MEMBER<br>98 weai_crop_decisions___98 NOT APPLICABLE<br><br>Custom alignment: LV
936	weai_crop_input<br><br>Show the field ONLY if:<br>[weai_crop_decisions(2)] = '1' or [weai_crop_decisions(3)] = '1' or [weai_crop_decisions(4)] = '1'	4b) How much input did you have in making decisions about food crop farming?	radio<br>1 NO INPUT OR INPUT IN FEW DECISIONS<br>2 INPUT INTO SOME DECISIONS<br>3 INPUT INTO MOST OR ALL DECISIONS<br>98 NO DECISION MADE<br><br>Custom alignment: LV
937	weai_crop_personal<br><br>Show the field ONLY if:<br>([weai_crop_input] = '1' or [weai_crop_input] = '2' or [weai_crop_input] = '3')	4c) To what extent do you feel you can make your own personal decisions regarding food crop farming if you want(ed) to?	radio<br>1 NOT AT ALL<br>2 SMALL EXTENT<br>3 MEDIUM EXTENT<br>4 TO A HIGH EXTENT<br><br>Custom alignment: LV

938	weai_crop_income<br><br>Show the field ONLY if: ([weai_crop_input] = '1' or [weai_crop_input] = '2' or [weai_crop_input] = '3') or ([weai_crop_decisions(1)] = '1' and [weai_crop_decisions(2)] = '0' and [weai_crop_decisions(4)] = '0' and [weai_crop_decisions(98)] = '0')	4d) How much input did you have in decisions on the use of income generated from food crop farming?	radio<br>1 NO INPUT OR INPUT IN FEW DECISIONS<br>2 INPUT INTO SOME DECISIONS<br>3 INPUT INTO MOST OR ALL DECISIONS<br>98 NO DECISION MADE<br><br>Custom alignment: LV
939	weai_cash	5) Did you yourself participate in Cash crop farming: These are crops that are grown primarily for sale in the market in the past 12 months (that is, during the last [one/two] cropping seasons), from [PRESENT MONTH] last year to [PRESENT MONTH] this year?	radio<br>1 Yes<br>2 No<br><br>Custom alignment: LV
940	weai_cash_decisions<br><br>Show the field ONLY if: [weai_cash] = '1'	5a) When decisions are made regarding cash crop farming, who is it that normally takes the decision?<br>*Check all that apply*	checkbox<br>1 weai_cash_decisions___1 SELF<br>2 weai_cash_decisions___2 SPOUSE<br>3 weai_cash_decisions___3 OTHER HH MEMBER<br>4 weai_cash_decisions___4 OTHER NON-HH MEMBER<br>98 weai_cash_decisions___98 NOT APPLICABLE<br><br>Custom alignment: LV
941	weai_cash_input<br><br>Show the field ONLY if: [weai_cash_decisions(2)] = '1' or [weai_cash_decisions(3)] = '1' or [weai_cash_decisions(4)] = '1'	5b) How much input did you have in making decisions about cash crop farming?	radio<br>1 NO INPUT OR INPUT IN FEW DECISIONS<br>2 INPUT INTO SOME DECISIONS<br>3 INPUT INTO MOST OR ALL DECISIONS<br>98 NO DECISION MADE<br><br>Custom alignment: LV
942	weai_cash_personal<br><br>Show the field ONLY if: [weai_cash_input] = '1' or [weai_cash_input] = '2' or [weai_cash_input] = '3'	5c) To what extent do you feel you can make your own personal decisions regarding cash crop farming if you want(ed) to?	radio<br>1 NOT AT ALL<br>2 SMALL EXTENT<br>3 MEDIUM EXTENT<br>4 TO A HIGH EXTENT<br><br>Custom alignment: LV
943	weai_cash_income<br><br>Show the field ONLY if: ([weai_cash_input] = '1' or [weai_cash_input] = '2' or [weai_cash_input] = '3') or ([weai_cash_decisions(1)] = '1' and [weai_cash_decisions(2)] = '0' and [weai_cash_decisions(4)] = '0' and [weai_cash_decisions(98)] = '0')	5d) How much input did you have in decisions on the use of income generated from cash crop farming?	radio<br>1 NO INPUT OR INPUT IN FEW DECISIONS<br>2 INPUT INTO SOME DECISIONS<br>3 INPUT INTO MOST OR ALL DECISIONS<br>98 NO DECISION MADE<br><br>Custom alignment: LV
944	weai_livestock	6) Did you yourself participate in livestock raising in the past 12 months (that is, during the last [one/two] cropping seasons), from [PRESENT MONTH] last year to [PRESENT MONTH] this year?	radio<br>1 Yes<br>2 No<br><br>Custom alignment: LV

945	weai_livestock_decisions<br><br>Show the field ONLY if:<br>[weai_livestock] = '1'	6a) When decisions are made regarding livestock raising, who is it that normally takes the decision?<br>*Check all that apply*	checkbox<br>1 weai_livestock_decisions___1 SELF<br>2 weai_livestock_decisions___2 SPOUSE<br>3 weai_livestock_decisions___3 OTHER HH MEMBER<br>4 weai_livestock_decisions___4 OTHER NON-HH MEMBER<br>98 weai_livestock_decisions___98 NOT APPLICABLE<br><br>Custom alignment: LV
946	weai_livestock_input<br><br>Show the field ONLY if:<br>[weai_livestock_decisions(2)] = '1' or [weai_livestock_decisions(3)] = '1' or [weai_livestock_decisions(4)] = '1'	6b) How much input did you have in making decisions about livestock raising?	radio<br>1 NO INPUT OR INPUT IN FEW DECISIONS<br>2 INPUT INTO SOME DECISIONS<br>3 INPUT INTO MOST OR ALL DECISIONS<br>98 NO DECISION MADE<br><br>Custom alignment: LV
947	weai_livestock_personal<br><br>Show the field ONLY if:<br>[weai_livestock_input] = '1' or [weai_livestock_input] = '2' or [weai_livestock_input] = '3'	6c) To what extent do you feel you can make your own personal decisions regarding livestock raising if you want(ed) to?	radio<br>1 NOT AT ALL<br>2 SMALL EXTENT<br>3 MEDIUM EXTENT<br>4 TO A HIGH EXTENT<br><br>Custom alignment: LV
948	weai_livestock_income<br><br>Show the field ONLY if:<br>([weai_livestock_input] = '1' or [weai_livestock_input] = '2' or [weai_livestock_input] = '3') or ([weai_livestock_decisions(1)] = '1' and [weai_livestock_decisions(2)] = '0' and [weai_livestock_decisions(4)] = '0' and [weai_livestock_decisions(98)] = '0')	6d) How much input did you have in decisions on the use of income generated from livestock raising?	radio<br>1 NO INPUT OR INPUT IN FEW DECISIONS<br>2 INPUT INTO SOME DECISIONS<br>3 INPUT INTO MOST OR ALL DECISIONS<br>98 NO DECISION MADE<br><br>Custom alignment: LV
949	weai_economic	7) Did you yourself participate in Non-farm economic activities: This would include things like running a small business, self-employment, buy-and-sell in the past 12 months (that is, during the last [one/two] cropping seasons), from [PRESENT MONTH] last year to [PRESENT MONTH] this year?	radio<br>1 Yes<br>2 No<br><br>Custom alignment: LV
950	weai_economic_decisions<br><br>Show the field ONLY if:<br>[weai_economic] = '1'	7a) When decisions are made regarding Non-farm economic activities, who is it that normally takes the decision?<br>*Check all that apply*	checkbox<br>1 weai_economic_decisions___1 SELF<br>2 weai_economic_decisions___2 SPOUSE<br>3 weai_economic_decisions___3 OTHER HH MEMBER<br>4 weai_economic_decisions___4 OTHER NON-HH MEMBER<br>98 weai_economic_decisions___98 NOT APPLICABLE<br><br>Custom alignment: LV
951	weai_economic_input<br><br>Show the field ONLY if:<br>[weai_economic_decisions(2)] = '1' or [weai_economic_decisions(3)] = '1' or [weai_economic_decisions(4)] = '1'	7b) How much input did you have in making decisions about Non-farm economic activities?	radio<br>1 NO INPUT OR INPUT IN FEW DECISIONS<br>2 INPUT INTO SOME DECISIONS<br>3 INPUT INTO MOST OR ALL DECISIONS<br>98 NO DECISION MADE<br><br>Custom alignment: LV

952	weai_economic_personal<br><br>Show the field ONLY if:<br>[weai_economic_input] = '1' or [weai_economic_input] = '2' or [weai_economic_input] = '3'	7c) To what extent do you feel you can make your own personal decisions regarding Non-farm economic activities if you want(ed) to?	radio<br>1 NOT AT ALL<br>2 SMALL EXTENT<br>3 MEDIUM EXTENT<br>4 TO A HIGH EXTENT<br><br>Custom alignment: LV
953	weai_economic_income<br><br>Show the field ONLY if:<br>([weai_economic_input] = '1' or [weai_economic_input] = '2' or [weai_economic_input] = '3') or ([weai_economic_decisions(1)] = '1' and [weai_economic_decisions(2)] = '0' and [weai_economic_decisions(4)] = '0' and [weai_economic_decisions(98)] = '0')	7d) How much input did you have in decisions on the use of income generated from non-farm economic activities?	radio<br>1 NO INPUT OR INPUT IN FEW DECISIONS<br>2 INPUT INTO SOME DECISIONS<br>3 INPUT INTO MOST OR ALL DECISIONS<br>98 NO DECISION MADE<br><br>Custom alignment: LV
954	weai_employ	8) Did you yourself participate in Wage and salary employment: This could be work that is paid for in cash or in-kind, including both agriculture and other wage work in the past 12 months (that is, during the last [one/two] cropping seasons), from [PRESENT MONTH] last year to [PRESENT MONTH] this year?	radio<br>1 Yes<br>2 No<br><br>Custom alignment: LV
955	weai_employ_decisions<br><br>Show the field ONLY if:<br>[weai_employ] = '1'	8a) When decisions are made regarding wage and salary employment, who is it that normally takes the decision?<br>*Check all that apply*	checkbox<br>1 weai_employ_decisions___1 SELF<br>2 weai_employ_decisions___2 SPOUSE<br>3 weai_employ_decisions___3 OTHER HH MEMBER<br>4 weai_employ_decisions___4 OTHER NON-HH MEMBER<br>98 weai_employ_decisions___98 NOT APPLICABLE<br><br>Custom alignment: LV
956	weai_employ_input<br><br>Show the field ONLY if:<br>[weai_employ_decisions(2)] = '1' or [weai_employ_decisions(3)] = '1' or [weai_employ_decisions(4)] = '1'	8b) How much input did you have in making decisions about wage and salary employment?	radio<br>1 NO INPUT OR INPUT IN FEW DECISIONS<br>2 INPUT INTO SOME DECISIONS<br>3 INPUT INTO MOST OR ALL DECISIONS<br>98 NO DECISION MADE<br><br>Custom alignment: LV
957	weai_employ_personal<br><br>Show the field ONLY if:<br>[weai_employ_input] = '1' or [weai_employ_input] = '2' or [weai_employ_input] = '3'	8c) To what extent do you feel you can make your own personal decisions regarding wage and salary employment if you want(ed) to?	radio<br>1 NOT AT ALL<br>2 SMALL EXTENT<br>3 MEDIUM EXTENT<br>4 TO A HIGH EXTENT<br><br>Custom alignment: LV
958	weai_employ_income<br><br>Show the field ONLY if:<br>([weai_employ_input] = '1' or [weai_employ_input] = '2' or [weai_employ_input] = '3') or ([weai_employ_decisions(1)] = '1' and [weai_employ_decisions(2)] = '0' and [weai_employ_decisions(4)] = '0' and [weai_employ_decisions(98)] = '0')	8d) How much input did you have in decisions on the use of income generated from wage and salary employment?	radio<br>1 NO INPUT OR INPUT IN FEW DECISIONS<br>2 INPUT INTO SOME DECISIONS<br>3 INPUT INTO MOST OR ALL DECISIONS<br>98 NO DECISION MADE<br><br>Custom alignment: LV

Havelaar AH, *et al. BMJ Open* 2022; 12:e061311. doi: 10.1136/bmjopen-2022-061311

959	weai_fishing	9) Did you yourself participate in Fishing or fishpond culture in the past 12 months (that is, during the last [one/two] cropping seasons), from [PRESENT MONTH] last year to [PRESENT MONTH] this year?	radio<br>1 Yes<br>2 No<br><br>Custom alignment: LV
960	weai_fishing_decisions<br><br>Show the field ONLY if:<br>[weai_fishing] = '1'	9a) When decisions are made regarding Fishing or fishpond culture, who is it that normally takes the decision?<br>*Check all that apply*	checkbox<br>1 weai_fishing_decisions___1 SELF<br>2 weai_fishing_decisions___2 SPOUSE<br>3 weai_fishing_decisions___3 OTHER HH MEMBER<br>4 weai_fishing_decisions___4 OTHER NON-HH MEMBER<br>98 weai_fishing_decisions___98 NOT APPLICABLE<br><br>Custom alignment: LV
961	weai_fishing_input<br><br>Show the field ONLY if:<br>[weai_fishing_decisions(2)] = '1' or [weai_fishing_decisions(3)] = '1' or [weai_fishing_decisions(4)] = '1'	9b) How much input did you have in making decisions about Fishing or fishpond culture?	radio<br>1 NO INPUT OR INPUT IN FEW DECISIONS<br>2 INPUT INTO SOME DECISIONS<br>3 INPUT INTO MOST OR ALL DECISIONS<br>98 NO DECISION MADE<br><br>Custom alignment: LV
962	weai_fishing_personal<br><br>Show the field ONLY if:<br>[weai_fishing_input] = '1' or [weai_fishing_input] = '2' or [weai_fishing_input] = '3'	9c) To what extent do you feel you can make your own personal decisions regarding Fishing or fishpond culture if you want(ed) to?	radio<br>1 NOT AT ALL<br>2 SMALL EXTENT<br>3 MEDIUM EXTENT<br>4 TO A HIGH EXTENT<br><br>Custom alignment: LV
963	weai_fishing_income<br><br>Show the field ONLY if:<br>([weai_fishing_input] = '1' or [weai_fishing_input] = '2' or [weai_fishing_input] = '3') or ([weai_fishing_decisions(1)] = '1' and [weai_fishing_decisions(2)] = '0' and [weai_fishing_decisions(4)] = '0' and [weai_fishing_decisions(98)] = '0')	9d) How much input did you have in decisions on the use of income generated from Fishing or fishpond culture?	radio<br>1 NO INPUT OR INPUT IN FEW DECISIONS<br>2 INPUT INTO SOME DECISIONS<br>3 INPUT INTO MOST OR ALL DECISIONS<br>98 NO DECISION MADE<br><br>Custom alignment: LV
964	weai_house_decisions	10) When decisions are made regarding Major household expenditures (such as bicycles, land, boda boda), who is it that normally takes the decision?<br>*Check all that apply*	checkbox<br>1 weai_house_decisions___1 SELF<br>2 weai_house_decisions___2 SPOUSE<br>3 weai_house_decisions___3 OTHER HH MEMBER<br>4 weai_house_decisions___4 OTHER NON-HH MEMBER<br>98 weai_house_decisions___98 NOT APPLICABLE<br><br>Custom alignment: LV
965	weai_house_input<br><br>Show the field ONLY if:<br>[weai_house_decisions(2)] = '1' or [weai_house_decisions(3)] = '1' or [weai_house_decisions(4)] = '1'	10a) How much input did you have in making decisions about Major household expenditures?	radio<br>1 NO INPUT OR INPUT IN FEW DECISIONS<br>2 INPUT INTO SOME DECISIONS<br>3 INPUT INTO MOST OR ALL DECISIONS<br>98 NO DECISION MADE<br><br>Custom alignment: LV

966	weai_house_personal<br><br>Show the field ONLY if:<br>[weai_house_input] = '1' or [weai_house_input] = '2' or [weai_house_input] = '3'	10b) To what extent do you feel you can make your own personal decisions regarding Major household expenditures if you want(ed) to?	radio<br>1 NOT AT ALL<br>2 SMALL EXTENT<br>3 MEDIUM EXTENT<br>4 TO A HIGH EXTENT<br><br>Custom alignment: LV
967	weai_minor_decisions	11) When decisions are made regarding Minor household expenditures (such as food for daily consumption or other household needs), who is it that normally takes the decision?<br>*Check all that apply*	checkbox<br>1 weai_minor_decisions___1 SELF<br>2 weai_minor_decisions___2 SPOUSE<br>3 weai_minor_decisions___3 OTHER HH MEMBER<br>4 weai_minor_decisions___4 OTHER NON-HH MEMBER<br>98 weai_minor_decisions___98 NOT APPLICABLE<br><br>Custom alignment: LV
968	weai_minor_input<br><br>Show the field ONLY if:<br>[weai_minor_decisions(2)] = '1' or [weai_minor_decisions(3)] = '1' or [weai_minor_decisions(4)] = '1'	11a) How much input did you have in making decisions about Minor household expenditures?	radio<br>1 NO INPUT OR INPUT IN FEW DECISIONS<br>2 INPUT INTO SOME DECISIONS<br>3 INPUT INTO MOST OR ALL DECISIONS<br>98 NO DECISION MADE<br><br>Custom alignment: LV
969	weai_minor_personal<br><br>Show the field ONLY if:<br>[weai_minor_input] = '1' or [weai_minor_input] = '2' or [weai_minor_input] = '3'	11b) To what extent do you feel you can make your own personal decisions regarding Minor household expenditures if you want(ed) to?	radio<br>1 NOT AT ALL<br>2 SMALL EXTENT<br>3 MEDIUM EXTENT<br>4 TO A HIGH EXTENT<br><br>Custom alignment: LV
970	weai_own_agri	Section Header: *"Now I'd like to ask you about your household's access to and ownership of a number of items that could be used to generate income."*<br>12) Does anyone in your household currently have any agricultural land (pieces/plots)?	radio<br>1 Yes<br>2 No<br><br>Custom alignment: LV
971	weai_own_agri_self<br><br>Show the field ONLY if:<br>[weai_own_agri] = '1'	12a) Do you own any of the agricultural land?	radio<br>1 YES, SOLELY<br>2 YES, JOINTLY<br>3 NO<br><br>Custom alignment: LV
972	weai_own_livestock	13) Does anyone in your household currently have any large livestock (oxen, cattle)?	radio<br>1 Yes<br>2 No<br><br>Custom alignment: LV
973	weai_own_livestock_self<br><br>Show the field ONLY if:<br>[weai_own_livestock] = '1'	13a) Do you own any of the large livestock?	radio<br>1 YES, SOLELY<br>2 YES, JOINTLY<br>3 NO<br><br>Custom alignment: LV

974	weai_own_livestock_self_y<br>Show the field ONLY if: [weai_own_livestock_self] = '1' or [weai_own_livestock_self] = '2'	Which large livestock do you own?	checkbox<br>1 weai_own_livestock_self_y___1 Oxen<br>2 weai_own_livestock_self_y___2 Cattle<br>88 weai_own_livestock_self_y___88 Other
975	weai_own_livestock_self_ox<br>Show the field ONLY if: [weai_own_livestock_self_y(1)] = '1'	How many oxen do you own?	text (number)
976	weai_own_livestock_self_cattle<br>Show the field ONLY if: [weai_own_livestock_self_y(2)] = '1'	How many cattle do you own?	text (number)
977	weai_own_livestock_self_o<br>Show the field ONLY if: [weai_own_livestock_self_y(88)] = '1'	Specify other large livestock	text
978	weai_own_livestock_self_o_n<br>Show the field ONLY if: [weai_own_livestock_self_y(88)] = '1'	How many Other large livestock do you own?	text (number)
979	weai_own_smlivestock	14) Does anyone in your household currently have any small livestock (goats, pigs, sheep)?	radio<br>1 Yes<br>2 No<br>Custom alignment: LV
980	weai_own_smlivestock_self<br>Show the field ONLY if: [weai_own_smlivestock] = '1'	14a) Do you own any of the small livestock?	radio<br>1 YES, SOLELY<br>2 YES, JOINTLY<br>3 NO<br>Custom alignment: LV
981	weai_own_smlivestock_self_own<br>Show the field ONLY if: [weai_own_smlivestock_self] = '1' or [weai_own_smlivestock_self] = '2'	Which small livestock do you own?	checkbox<br>1 weai_own_smlivestock_self_own___1 Goats<br>2 weai_own_smlivestock_self_own___2 Pigs<br>3 weai_own_smlivestock_self_own___3 Sheep<br>88 weai_own_smlivestock_self_own___88 Other
982	weai_own_smlivestock_self_goats<br>Show the field ONLY if: [weai_own_smlivestock_self_own(1)] = '1'	How many goats do you own?	text (number)
983	weai_own_smlivestock_self_pigs<br>Show the field ONLY if: [weai_own_smlivestock_self_own(2)] = '1'	How many pigs do you own?	text (number)
984	weai_own_smlivestock_sheep<br>Show the field ONLY if: [weai_own_smlivestock_self_own(3)] = '1'	How many sheep do you own?	text (number)
985	weai_own_smlivestock_other<br>Show the field ONLY if: [weai_own_smlivestock_self_own(88)] = '1'	Specify other small livestock	text

986	weai_own_smlivestock_self_other_n<br>Show the field ONLY if: [weai_own_smlivestock_self_own(88)] = '1'	How many other small livestock do you own?	text (number)
987	weai_own_foul	15) Does anyone in your household currently have any Chickens, Ducks, Turkeys, Pigeons?	radio<br>1 Yes<br>2 No<br>Custom alignment: LV
988	weai_own_foul_self<br>Show the field ONLY if: [weai_own_foul] = '1'	15a) Do you own any of the Chickens, Ducks, Turkeys, Pigeons?	radio<br>1 YES, SOLELY<br>2 YES, JOINTLY<br>3 NO<br>Custom alignment: LV
989	weai_own_foul_self_type<br>Show the field ONLY if: [weai_own_foul_self] = '1' or [weai_own_foul_self] = '2'	Which do you own?	checkbox<br>1 weai_own_foul_self_type___1 Chickens<br>2 weai_own_foul_self_type___2 Ducks<br>3 weai_own_foul_self_type___3 Turkeys<br>4 weai_own_foul_self_type___4 Pigeons<br>88 weai_own_foul_self_type___88 Other foul
990	weai_own_foul_chicken<br>Show the field ONLY if: [weai_own_foul_self_type(1)] = '1'	How many chickens do you own?	text (number)
991	weai_own_foul_duck<br>Show the field ONLY if: [weai_own_foul_self_type(2)] = '1'	How many ducks do you own?	text (number)
992	weai_own_foul_turkey<br>Show the field ONLY if: [weai_own_foul_self_type(3)] = '1'	How many turkeys do you own?	text (number)
993	weai_own_foul_pigeons<br>Show the field ONLY if: [weai_own_foul_self_type(4)] = '1'	How many pigeons do you own?	text (number)
994	weai_own_foul_other<br>Show the field ONLY if: [weai_own_foul_self_type(88)] = '1'	Specify other foul	text
995	weai_own_foul_other_number<br>Show the field ONLY if: [weai_own_foul_self_type(88)] = '1'	How many other foul do you own?	text (number)
996	weai_own_fish	16) Does anyone in your household currently have any fish pond or fishing equipment?	radio<br>1 Yes<br>2 No<br>Custom alignment: LV
997	weai_own_fish_self<br>Show the field ONLY if: [weai_own_fish] = '1'	16a) Do you own any of the fish pond or fishing equipment?	radio<br>1 YES, SOLELY<br>2 YES, JOINTLY<br>3 NO<br>Custom alignment: LV

998	weai_own_nmequip	17) Does anyone in your household currently have any farm equipment (nonmechanized: hand tools, animal-drawn plough)?	radio<br>1 Yes<br>2 No<br>Custom alignment: LV
999	weai_own_nmequip_self<br>Show the field ONLY if:<br>[weai_own_nmequip] = '1'	17a) Do you own any of the farm equipment (nonmechanized: hand tools, animal-drawn plough)?	radio<br>1 YES, SOLELY<br>2 YES, JOINTLY<br>3 NO<br>Custom alignment: LV
1000	weai_own_mequip	18) Does anyone in your household currently have any farm equipment (mechanized: tractor-plough, power tiller, treadle pump)?	radio<br>1 Yes<br>2 No<br>Custom alignment: LV
1001	weai_own_mequip_self<br>Show the field ONLY if:<br>[weai_own_mequip] = '1'	18a) Do you own any of the farm equipment (mechanized: tractor-plough, power tiller, treadle pump)?	radio<br>1 YES, SOLELY<br>2 YES, JOINTLY<br>3 NO<br>Custom alignment: LV
1002	weai_own_buisness	19) Does anyone in your household currently have any nonfarm business equipment (solar panels used for recharging, sewing machine, brewing equipment, fryers)?	radio<br>1 Yes<br>2 No<br>Custom alignment: LV
1003	weai_own_buisness_self<br>Show the field ONLY if:<br>[weai_own_buisness] = '1'	19a) Do you own any of the nonfarm business equipment (solar panels used for recharging, sewing machine, brewing equipment, fryers)?	radio<br>1 YES, SOLELY<br>2 YES, JOINTLY<br>3 NO<br>Custom alignment: LV
1004	weai_own_house	20) Does anyone in your household currently have any houses or other structures?	radio<br>1 Yes<br>2 No<br>Custom alignment: LV
1005	weai_own_house_self<br>Show the field ONLY if:<br>[weai_own_house] = '1'	20a) Do you own any of the houses or other structures?	radio<br>1 YES, SOLELY<br>2 YES, JOINTLY<br>3 NO<br>Custom alignment: LV
1006	weai_own_lgdurables	Section Header: *"Now I'd like to ask you about your household's access to and ownership of a number of items that could be used to generate income."*<br>1) Does anyone in your household currently have any large consumer durables (refrigerator, TV, sofa)?	radio<br>1 Yes<br>2 No<br>Custom alignment: LV
1007	weai_own_lgdurables_self<br>Show the field ONLY if:<br>[weai_own_lgdurables] = '1'	1a) Do you own any of the large consumer durables?	radio<br>1 YES, SOLELY<br>2 YES, JOINTLY<br>3 NO<br>Custom alignment: LV

1008	weai_own_smdurables	2) Does anyone in your household currently have any small consumer durables (radio, cookware)?	radio<br>1 Yes<br>2 No<br>Custom alignment: LV
1009	weai_own_smdurables_self<br>Show the field ONLY if:<br>[weai_own_smdurables] = '1'	2a) Do you own any of the small consumer durables?	radio<br>1 YES, SOLELY<br>2 YES, JOINTLY<br>3 NO<br>Custom alignment: LV
1010	weai_own_cell	3) Does anyone in your household currently have a cell phone?	radio<br>1 Yes<br>2 No<br>Custom alignment: LV
1011	weai_own_cell_self<br>Show the field ONLY if:<br>[weai_own_cell] = '1'	3a) Do you own a cell phone?	radio<br>1 YES, SOLELY<br>2 YES, JOINTLY<br>3 NO<br>Custom alignment: LV
1012	weai_own_land	4) Does anyone in your household currently have any other land not used for agricultural purposes (pieces/plots, residential or commercial land)?	radio<br>1 Yes<br>2 No<br>Custom alignment: LV
1013	weai_own_land_self<br>Show the field ONLY if:<br>[weai_own_land] = '1'	4a) Do you own any of the other land not used for agricultural purposes (pieces/plots, residential or commercial land)?	radio<br>1 YES, SOLELY<br>2 YES, JOINTLY<br>3 NO<br>Custom alignment: LV
1014	weai_own_transport	5) Does anyone in your household currently have any means of transportation (bicycle, motorcycle, car)	radio<br>1 Yes<br>2 No<br>Custom alignment: LV
1015	weai_own_transport_self<br>Show the field ONLY if:<br>[weai_own_transport] = '1'	5a) Do you own any of the means of transportation (bicycle, motorcycle, car)?	radio<br>1 YES, SOLELY<br>2 YES, JOINTLY<br>3 NO<br>Custom alignment: LV
1016	weai_loan_ngo	Section Header: *"Next I'd like to ask about your household's experience with borrowing money or other items in the past 12 months."*<br>1) Would you or anyone in your household be able to take a loan or borrow cash/in-kind from a Non-governmental organization (NGO) if you wanted to?	radio<br>1 Yes<br>2 No<br>3 Maybe<br>Custom alignment: LV

Supplemental material
BMJ Publishing Group Limited (BMJ) disclaims all liability and responsibility arising from any reliance placed on this supplemental material which has been supplied by the author(s)
BMJ Open

1017	weai_loan_ngo_year<br>Show the field ONLY if: [weai_loan_ngo] = '1' or [weai_loan_ngo] = '3'	1a) Has anyone in your household taken any loans or borrowed cash/in-kind from a Non-governmental organization (NGO) in the past 12 months?	radio<br>1 YES, CASH<br>2 YES, IN-KIND<br>3 YES, CASH AND IN-KIND<br>4 NO<br>97 DON'T KNOW<br>Custom alignment: LV
1018	weai_loan_ngo_decision<br>Show the field ONLY if: [weai_loan_ngo_year] = '1' or [weai_loan_ngo_year] = '2' or [weai_loan_ngo_year] = '3'	1b) Who made the decision to borrow from Non- governmental organization (NGO) most of the time?<br>*Check all that apply*	checkbox<br>1 weai_loan_ngo_decision___1 SELF<br>2 weai_loan_ngo_decision___2 SPOUSE<br>3 weai_loan_ngo_decision___3 OTHER HH MEMBER<br>4 weai_loan_ngo_decision___4 OTHER NON-HH MEMBER<br>98 weai_loan_ngo_decision___98 NOT APPLICABLE<br>Custom alignment: LV
1019	weai_loan_ngo_income<br>Show the field ONLY if: [weai_loan_ngo_year] = '1' or [weai_loan_ngo_year] = '2' or [weai_loan_ngo_year] = '3'	1c) Who makes the decision about what to do with the money/ item borrowed from Non- governmental organization (NGO) most of the time?<br>*Check all that apply*	checkbox<br>1 weai_loan_ngo_income___1 SELF<br>2 weai_loan_ngo_income___2 SPOUSE<br>3 weai_loan_ngo_income___3 OTHER HH MEMBER<br>4 weai_loan_ngo_income___4 OTHER NON-HH MEMBER<br>98 weai_loan_ngo_income___98 NOT APPLICABLE<br>Custom alignment: LV
1020	weai_loan_fmlender	2) Would you or anyone in your household be able to take a loan or borrow cash/in-kind from a Formal lender (bank/financial institution) if you wanted to?	radio<br>1 Yes<br>2 No<br>3 Maybe<br>Custom alignment: LV
1021	weai_loan_fmlender_year<br>Show the field ONLY if: [weai_loan_fmlender] = '1' or [weai_loan_fmlender] = '3'	2a) Has anyone in your household taken any loans or borrowed cash/in-kind from a formal lender in the past 12 months?	radio<br>1 YES, CASH<br>2 YES, IN-KIND<br>3 YES, CASH AND IN-KIND<br>4 NO<br>97 DON'T KNOW<br>Custom alignment: LV
1022	weai_loan_fmlender_decision<br>Show the field ONLY if: [weai_loan_fmlender_year] = '1' or [weai_loan_fmlender_year] = '2' or [weai_loan_fmlender_year] = '3'	2b) Who made the decision to borrow from a formal lender most of the time?<br>*Check all that apply*	checkbox<br>1 weai_loan_fmlender_decision___1 SELF<br>2 weai_loan_fmlender_decision___2 SPOUSE<br>3 weai_loan_fmlender_decision___3 OTHER HH MEMBER<br>4 weai_loan_fmlender_decision___4 OTHER NON-HH MEMBER<br>98 weai_loan_fmlender_decision___98 NOT APPLICABLE<br>Custom alignment: LV

Havelaar AH, *et al. BMJ Open* 2022; 12:e061311. doi: 10.1136/bmjopen-2022-061311

1023	weai_loan_fmlender_income<br>Show the field ONLY if: [weai_loan_fmlender_year] = '1' or [weai_loan_fmlender_year] = '2' or [weai_loan_fmlender_year] = '3'	2c) Who makes the decision about what to do with the money/ item borrowed from a formal lender most of the time?<br>*Check all that apply*	checkbox<br>1 weai_loan_fmlender_income___1 SELF<br>2 weai_loan_fmlender_income___2 SPOUSE<br>3 weai_loan_fmlender_income___3 OTHER HH MEMBER<br>4 weai_loan_fmlender_income___4 OTHER NON-HH MEMBER<br>98 weai_loan_fmlender_income___98 NOT APPLICABLE<br>Custom alignment: LV
1024	weai_loan_inflender	3) Would you or anyone in your household be able to take a loan or borrow cash/in-kind from an informal lender if you wanted to?	radio<br>1 Yes<br>2 No<br>3 Maybe<br>Custom alignment: LV
1025	weai_loan_inflender_year<br>Show the field ONLY if: [weai_loan_inflender] = '1' or [weai_loan_inflender] = '3'	3a) Has anyone in your household taken any loans or borrowed cash/in-kind from an informal lender in the past 12 months?	radio<br>1 YES, CASH<br>2 YES, IN-KIND<br>3 YES, CASH AND IN-KIND<br>4 NO<br>97 DON'T KNOW<br>Custom alignment: LV
1026	weai_loan_inflender_decision<br>Show the field ONLY if: [weai_loan_inflender_year] = '1' or [weai_loan_inflender_year] = '2' or [weai_loan_inflender_year] = '3'	3b) Who made the decision to borrow from an informal lender most of the time?<br>*Check all that apply*	checkbox<br>1 weai_loan_inflender_decision___1 SELF<br>2 weai_loan_inflender_decision___2 SPOUSE<br>3 weai_loan_inflender_decision___3 OTHER HH MEMBER<br>4 weai_loan_inflender_decision___4 OTHER NON-HH MEMBER<br>98 weai_loan_inflender_decision___98 NOT APPLICABLE<br>Custom alignment: LV
1027	weai_loan_inflender_income<br>Show the field ONLY if: [weai_loan_inflender_year] = '1' or [weai_loan_inflender_year] = '2' or [weai_loan_inflender_year] = '3'	3c) Who makes the decision about what to do with the money/ item borrowed from an informal lender most of the time?<br>*Check all that apply*	checkbox<br>1 weai_loan_inflender_income___1 SELF<br>2 weai_loan_inflender_income___2 SPOUSE<br>3 weai_loan_inflender_income___3 OTHER HH MEMBER<br>4 weai_loan_inflender_income___4 OTHER NON-HH MEMBER<br>98 weai_loan_inflender_income___98 NOT APPLICABLE<br>Custom alignment: LV
1028	weai_loan_family	4) Would you or anyone in your household be able to take a loan or borrow cash/in-kind from friends or relatives if you wanted to?	radio<br>1 Yes<br>2 No<br>3 Maybe<br>Custom alignment: LV

Havelaar AH, *et al. BMJ Open* 2022; 12:e061311. doi: 10.1136/bmjopen-2022-061311

1029	weai_loan_family_year<br>Show the field ONLY if:<br>[weai_loan_family] = '1' or [weai_loan_family] = '3'	4a) Has anyone in your household taken any loans or borrowed cash/in-kind from friends or relatives in the past 12 months?	radio<br>1 YES, CASH<br>2 YES, IN-KIND<br>3 YES, CASH AND IN-KIND<br>4 NO<br>97 DON'T KNOW<br>Custom alignment: LV
1030	weai_loan_family_decision<br>Show the field ONLY if:<br>[weai_loan_family_year] = '1' or [weai_loan_family_year] = '2' or [weai_loan_family_year] = '3'	4b) Who made the decision to borrow from friends or relatives most of the time?<br>*Check all that apply*	checkbox<br>1 weai_loan_family_decision___1 SELF<br>2 weai_loan_family_decision___2 SPOUSE<br>3 weai_loan_family_decision___3 OTHER HH MEMBER<br>4 weai_loan_family_decision___4 OTHER NON-HH MEMBER<br>98 weai_loan_family_decision___98 NOT APPLICABLE<br>Custom alignment: LV
1031	weai_loan_family_income<br>Show the field ONLY if:<br>[weai_loan_family_year] = '1' or [weai_loan_family_year] = '2' or [weai_loan_family_year] = '3'	4c) Who makes the decision about what to do with the money/item borrowed from friends or relatives most of the time?<br>*Check all that apply*	checkbox<br>1 weai_loan_family_income___1 SELF<br>2 weai_loan_family_income___2 SPOUSE<br>3 weai_loan_family_income___3 OTHER HH MEMBER<br>4 weai_loan_family_income___4 OTHER NON-HH MEMBER<br>98 weai_loan_family_income___98 NOT APPLICABLE<br>Custom alignment: LV
1032	weai_loan_micro	5) Would you or anyone in your household be able to take a loan or borrow cash/in-kind from group based microfinance or lending including VSLAs / SACCOs if you wanted to?	radio<br>1 Yes<br>2 No<br>3 Maybe<br>Custom alignment: LV
1033	weai_loan_micro_year<br>Show the field ONLY if:<br>[weai_loan_micro] = '1' or [weai_loan_micro] = '3'	5a) Has anyone in your household taken any loans or borrowed cash/in-kind from Group based microfinance or lending including VSLAs / SACCOs in the past 12 months?	radio<br>1 YES, CASH<br>2 YES, IN-KIND<br>3 YES, CASH AND IN-KIND<br>4 NO<br>97 DON'T KNOW<br>Custom alignment: LV
1034	weai_loan_micro_decision<br>Show the field ONLY if:<br>[weai_loan_micro_year] = '1' or [weai_loan_micro_year] = '2' or [weai_loan_micro_year] = '3'	5b) Who made the decision to borrow from Group based microfinance or lending including VSLAs / SACCOs most of the time?<br>*Check all that apply*	checkbox<br>1 weai_loan_micro_decision___1 SELF<br>2 weai_loan_micro_decision___2 SPOUSE<br>3 weai_loan_micro_decision___3 OTHER HH MEMBER<br>4 weai_loan_micro_decision___4 OTHER NON-HH MEMBER<br>98 weai_loan_micro_decision___98 NOT APPLICABLE<br>Custom alignment: LV

1035	weai_loan_micro_income<br>Show the field ONLY if:<br>[weai_loan_micro_year] = '1' or [weai_loan_micro_year] = '2' or [weai_loan_micro_year] = '3'	5c) Who makes the decision about what to do with the money/ item borrowed from Group based microfinance or lending including VSLAs / SACCOs most of the time?<br>*Check all that apply*	checkbox<br>1 weai_loan_micro_income___1 SELF<br>2 weai_loan_micro_income___2 SPOUSE<br>3 weai_loan_micro_income___3 OTHER HH MEMBER<br>4 weai_loan_micro_income___4 OTHER NON-HH MEMBER<br>98 weai_loan_micro_income___98 NOT APPLICABLE<br>Custom alignment: LV
1036	weai_loan_credit	6) Would you or anyone in your household be able to take a loan or borrow cash/in-kind from informal credit/savings groups such as merry-go-rounds, tontines, funeral societies, etc. if you wanted to?	radio<br>1 Yes<br>2 No<br>3 Maybe<br>Custom alignment: LV
1037	weai_loan_credit_year<br>Show the field ONLY if:<br>[weai_loan_credit] = '1' or [weai_loan_credit] = '3'	6a) Has anyone in your household taken any loans or borrowed cash/in-kind from informal credit/savings groups such as merry-go- rounds, tontines, funeral societies, etc. in the past 12 months?	radio<br>1 YES, CASH<br>2 YES, IN-KIND<br>3 YES, CASH AND IN-KIND<br>4 NO<br>97 DON'T KNOW<br>Custom alignment: LV
1038	weai_loan_credit_decision<br>Show the field ONLY if:<br>[weai_loan_credit_year] = '1' or [weai_loan_credit_year] = '2' or [weai_loan_credit_year] = '3'	6b) Who made the decision to borrow from informal credit/savings groups such as merry-go-rounds, tontines, funeral societies, etc. most of the time?<br>*Check all that apply*	checkbox<br>1 weai_loan_credit_decision___1 SELF<br>2 weai_loan_credit_decision___2 SPOUSE<br>3 weai_loan_credit_decision___3 OTHER HH MEMBER<br>4 weai_loan_credit_decision___4 OTHER NON-HH MEMBER<br>98 weai_loan_credit_decision___98 NOT APPLICABLE<br>Custom alignment: LV
1039	weai_loan_credit_income<br>Show the field ONLY if:<br>[weai_loan_credit_year] = '1' or [weai_loan_credit_year] = '2' or [weai_loan_credit_year] = '3'	6c) Who makes the decision about what to do with the money/ item borrowed from informal credit/savings groups such as merry-go-rounds, tontines, funeral societies, etc.most of the time?<br>*Check all that apply*	checkbox<br>1 weai_loan_credit_income___1 SELF<br>2 weai_loan_credit_income___2 SPOUSE<br>3 weai_loan_credit_income___3 OTHER HH MEMBER<br>4 weai_loan_credit_income___4 OTHER NON-HH MEMBER<br>98 weai_loan_credit_income___98 NOT APPLICABLE<br>Custom alignment: LV
1040	weai_time	Section Header: *PLEASE RECORD A LOG OF THE ACTIVITIES FOR THE INDIVIDUAL IN THE LAST COMPLETE 24 HOURS (STARTING YESTERDAY MORNING AT 4 AM, FINISHING 3:59 AM OF THE CURRENT DAY). THE TIME INTERVALS ARE MARKED IN 15 MIN INTERVALS AND ONE ACTIVITY CAN BE MARKED FOR EACH TIME PERIOD BY DRAWING A LINE THROUGH THAT ACTIVITY. USE LOCAL TIME*<br>1) "Now I'd like to ask you about how you spent your time during the past 24 hours. We'll begin from yesterday morning, and continue through to this morning. This will be a detailed accounting. I'm interested in everything you do (i.e. resting, eating, personal care, work inside and outside the home, caring for children, cooking, shopping, socializing, etc.), even if it doesn't take you much time."Note: Please check all that apply for all the forms.<br>"Use local time"	descriptive

1041	weai_time_1_sleep	Section Header: *Night/Early Morning*<br>Sleeping and resting	checkbox<br>1 weai_time_1_sleep___1 4:00-4:15<br>2 weai_time_1_sleep___2 4:15-4:30<br>3 weai_time_1_sleep___3 4:30-4:45<br>4 weai_time_1_sleep___4 4:45-5:00<br>5 weai_time_1_sleep___5 5:00-5:15<br>6 weai_time_1_sleep___6 5:15-5:30<br>7 weai_time_1_sleep___7 5:30-5:45<br>8 weai_time_1_sleep___8 5:45-6:00
1042	weai_time_1_eat	Eating and drinking	checkbox<br>1 weai_time_1_eat___1 4:00-4:15<br>2 weai_time_1_eat___2 4:15-4:30<br>3 weai_time_1_eat___3 4:30-4:45<br>4 weai_time_1_eat___4 4:45-5:00<br>5 weai_time_1_eat___5 5:00-5:15<br>6 weai_time_1_eat___6 5:15-5:30<br>7 weai_time_1_eat___7 5:30-5:45<br>8 weai_time_1_eat___8 5:45-6:00
1043	weai_time_1_personal	Personal Care	checkbox<br>1 weai_time_1_personal___1 4:00-4:15<br>2 weai_time_1_personal___2 4:15-4:30<br>3 weai_time_1_personal___3 4:30-4:45<br>4 weai_time_1_personal___4 4:45-5:00<br>5 weai_time_1_personal___5 5:00-5:15<br>6 weai_time_1_personal___6 5:15-5:30<br>7 weai_time_1_personal___7 5:30-5:45<br>8 weai_time_1_personal___8 5:45-6:00
1044	weai_time_1_school	School (also homework)	checkbox<br>1 weai_time_1_school___1 4:00-4:15<br>2 weai_time_1_school___2 4:15-4:30<br>3 weai_time_1_school___3 4:30-4:45<br>4 weai_time_1_school___4 4:45-5:00<br>5 weai_time_1_school___5 5:00-5:15<br>6 weai_time_1_school___6 5:15-5:30<br>7 weai_time_1_school___7 5:30-5:45<br>8 weai_time_1_school___8 5:45-6:00
1045	weai_time_1_work	Work as employed	checkbox<br>1 weai_time_1_work___1 4:00-4:15<br>2 weai_time_1_work___2 4:15-4:30<br>3 weai_time_1_work___3 4:30-4:45<br>4 weai_time_1_work___4 4:45-5:00<br>5 weai_time_1_work___5 5:00-5:15<br>6 weai_time_1_work___6 5:15-5:30<br>7 weai_time_1_work___7 5:30-5:45<br>8 weai_time_1_work___8 5:45-6:00

1046	weai_time_1_buisness	Own business work	checkbox<br>1 weai_time_1_buisness___1 4:00-4:15<br>2 weai_time_1_buisness___2 4:15-4:30<br>3 weai_time_1_buisness___3 4:30-4:45<br>4 weai_time_1_buisness___4 4:45-5:00<br>5 weai_time_1_buisness___5 5:00-5:15<br>6 weai_time_1_buisness___6 5:15-5:30<br>7 weai_time_1_buisness___7 5:30-5:45<br>8 weai_time_1_buisness___8 5:45-6:00
1047	weai_time_1_farm	Farming/livestock/fishing	checkbox<br>1 weai_time_1_farm___1 4:00-4:15<br>2 weai_time_1_farm___2 4:15-4:30<br>3 weai_time_1_farm___3 4:30-4:45<br>4 weai_time_1_farm___4 4:45-5:00<br>5 weai_time_1_farm___5 5:00-5:15<br>6 weai_time_1_farm___6 5:15-5:30<br>7 weai_time_1_farm___7 5:30-5:45<br>8 weai_time_1_farm___8 5:45-6:00
1048	weai_time_1_shop	Shopping/getting service (incl health services)	checkbox<br>1 weai_time_1_shop___1 4:00-4:15<br>2 weai_time_1_shop___2 4:15-4:30<br>3 weai_time_1_shop___3 4:30-4:45<br>4 weai_time_1_shop___4 4:45-5:00<br>5 weai_time_1_shop___5 5:00-5:15<br>6 weai_time_1_shop___6 5:15-5:30<br>7 weai_time_1_shop___7 5:30-5:45<br>8 weai_time_1_shop___8 5:45-6:00
1049	weai_time_1_weave	Weaving, sewing, textile care	checkbox<br>1 weai_time_1_weave___1 4:00-4:15<br>2 weai_time_1_weave___2 4:15-4:30<br>3 weai_time_1_weave___3 4:30-4:45<br>4 weai_time_1_weave___4 4:45-5:00<br>5 weai_time_1_weave___5 5:00-5:15<br>6 weai_time_1_weave___6 5:15-5:30<br>7 weai_time_1_weave___7 5:30-5:45<br>8 weai_time_1_weave___8 5:45-6:00
1050	weai_time_1_cook	Cooking	checkbox<br>1 weai_time_1_cook___1 4:00-4:15<br>2 weai_time_1_cook___2 4:15-4:30<br>3 weai_time_1_cook___3 4:30-4:45<br>4 weai_time_1_cook___4 4:45-5:00<br>5 weai_time_1_cook___5 5:00-5:15<br>6 weai_time_1_cook___6 5:15-5:30<br>7 weai_time_1_cook___7 5:30-5:45<br>8 weai_time_1_cook___8 5:45-6:00

Havelaar AH, *et al. BMJ Open* 2022; 12:e061311. doi: 10.1136/bmjopen-2022-061311

1051	weai_time_1_domestic	Domestic work (incl fetching wood and water)	checkbox<br>1 weai_time_1_domestic___1 4:00-4:15<br>2 weai_time_1_domestic___2 4:15-4:30<br>3 weai_time_1_domestic___3 4:30-4:45<br>4 weai_time_1_domestic___4 4:45-5:00<br>5 weai_time_1_domestic___5 5:00-5:15<br>6 weai_time_1_domestic___6 5:15-5:30<br>7 weai_time_1_domestic___7 5:30-5:45<br>8 weai_time_1_domestic___8 5:45-6:00
1052	weai_time_1_care	Care for children/adults/elderly	checkbox<br>1 weai_time_1_care___1 4:00-4:15<br>2 weai_time_1_care___2 4:15-4:30<br>3 weai_time_1_care___3 4:30-4:45<br>4 weai_time_1_care___4 4:45-5:00<br>5 weai_time_1_care___5 5:00-5:15<br>6 weai_time_1_care___6 5:15-5:30<br>7 weai_time_1_care___7 5:30-5:45<br>8 weai_time_1_care___8 5:45-6:00
1053	weai_time_1_travel	Travelling and communiting	checkbox<br>1 weai_time_1_travel___1 4:00-4:15<br>2 weai_time_1_travel___2 4:15-4:30<br>3 weai_time_1_travel___3 4:30-4:45<br>4 weai_time_1_travel___4 4:45-5:00<br>5 weai_time_1_travel___5 5:00-5:15<br>6 weai_time_1_travel___6 5:15-5:30<br>7 weai_time_1_travel___7 5:30-5:45<br>8 weai_time_1_travel___8 5:45-6:00
1054	weai_time_1_tv	Watching TV/listening to radio/reading	checkbox<br>1 weai_time_1_tv___1 4:00-4:15<br>2 weai_time_1_tv___2 4:15-4:30<br>3 weai_time_1_tv___3 4:30-4:45<br>4 weai_time_1_tv___4 4:45-5:00<br>5 weai_time_1_tv___5 5:00-5:15<br>6 weai_time_1_tv___6 5:15-5:30<br>7 weai_time_1_tv___7 5:30-5:45<br>8 weai_time_1_tv___8 5:45-6:00
1055	weai_time_1_exercising	Exercising	checkbox<br>1 weai_time_1_exercising___1 4:00-4:15<br>2 weai_time_1_exercising___2 4:15-4:30<br>3 weai_time_1_exercising___3 4:30-4:45<br>4 weai_time_1_exercising___4 4:45-5:00<br>5 weai_time_1_exercising___5 5:00-5:15<br>6 weai_time_1_exercising___6 5:15-5:30<br>7 weai_time_1_exercising___7 5:30-5:45<br>8 weai_time_1_exercising___8 5:45-6:00

Havelaar AH, *et al. BMJ Open* 2022; 12:e061311. doi: 10.1136/bmjopen-2022-061311

Supplemental material BMJ Publishing Group Limited (BMJ) disclaims all liability and responsibility arising from any reliance placed on this supplemental material which has been supplied by the author(s) *BMJ Open*

1056	weai_time_1_social	Social activities and hobbies	checkbox<br>1 weai_time_1_social___1 4:00-4:15<br>2 weai_time_1_social___2 4:15-4:30<br>3 weai_time_1_social___3 4:30-4:45<br>4 weai_time_1_social___4 4:45-5:00<br>5 weai_time_1_social___5 5:00-5:15<br>6 weai_time_1_social___6 5:15-5:30<br>7 weai_time_1_social___7 5:30-5:45<br>8 weai_time_1_social___8 5:45-6:00
1057	weai_time_1_religious	Religious activities	checkbox<br>1 weai_time_1_religious___1 4:00-4:15<br>2 weai_time_1_religious___2 4:15-4:30<br>3 weai_time_1_religious___3 4:30-4:45<br>4 weai_time_1_religious___4 4:45-5:00<br>5 weai_time_1_religious___5 5:00-5:15<br>6 weai_time_1_religious___6 5:15-5:30<br>7 weai_time_1_religious___7 5:30-5:45<br>8 weai_time_1_religious___8 5:45-6:00
1058	weai_time_1_other	Other (specify below)	checkbox<br>1 weai_time_1_other___1 4:00-4:15<br>2 weai_time_1_other___2 4:15-4:30<br>3 weai_time_1_other___3 4:30-4:45<br>4 weai_time_1_other___4 4:45-5:00<br>5 weai_time_1_other___5 5:00-5:15<br>6 weai_time_1_other___6 5:15-5:30<br>7 weai_time_1_other___7 5:30-5:45<br>8 weai_time_1_other___8 5:45-6:00
1059	weai_time_1_other_specify<br>Show the field ONLY if: [weai_time_1_other(1)] or [weai_time_1_other(2)] or [weai_time_1_other(3)] or [weai_time_1_other(4)] or [weai_time_1_other(5)] or [weai_time_1_other(6)] or [weai_time_1_other(7)] or [weai_time_1_other(8)]	Specify Other	text<br>Custom alignment: LV
1060	weai_time_2_sleep	Section Header: *Morning*<br>Sleeping and resting	checkbox<br>1 weai_time_2_sleep___1 6:00-6:15<br>2 weai_time_2_sleep___2 6:15-6:30<br>3 weai_time_2_sleep___3 6:30-6:45<br>4 weai_time_2_sleep___4 6:45-7:00<br>5 weai_time_2_sleep___5 7:00-7:15<br>6 weai_time_2_sleep___6 7:15-7:30<br>7 weai_time_2_sleep___7 7:30-7:45<br>8 weai_time_2_sleep___8 7:45-8:00

Havelaar AH, *et al. BMJ Open* 2022; 12:e061311. doi: 10.1136/bmjopen-2022-061311

1061	weai_time_2_eat	Eating and drinking	checkbox<br>1 weai_time_2_eat___1 6:00-6:15<br>2 weai_time_2_eat___2 6:15-6:30<br>3 weai_time_2_eat___3 6:30-6:45<br>4 weai_time_2_eat___4 6:45-7:00<br>5 weai_time_2_eat___5 7:00-7:15<br>6 weai_time_2_eat___6 7:15-7:30<br>7 weai_time_2_eat___7 7:30-7:45<br>8 weai_time_2_eat___8 7:45-8:00
1062	weai_time_2_personal	Personal Care	checkbox<br>1 weai_time_2_personal___1 6:00-6:15<br>2 weai_time_2_personal___2 6:15-6:30<br>3 weai_time_2_personal___3 6:30-6:45<br>4 weai_time_2_personal___4 6:45-7:00<br>5 weai_time_2_personal___5 7:00-7:15<br>6 weai_time_2_personal___6 7:15-7:30<br>7 weai_time_2_personal___7 7:30-7:45<br>8 weai_time_2_personal___8 7:45-8:00
1063	weai_time_2_school	School (also homework)	checkbox<br>1 weai_time_2_school___1 6:00-6:15<br>2 weai_time_2_school___2 6:15-6:30<br>3 weai_time_2_school___3 6:30-6:45<br>4 weai_time_2_school___4 6:45-7:00<br>5 weai_time_2_school___5 7:00-7:15<br>6 weai_time_2_school___6 7:15-7:30<br>7 weai_time_2_school___7 7:30-7:45<br>8 weai_time_2_school___8 7:45-8:00
1064	weai_time_2_work	Work as employed	checkbox<br>1 weai_time_2_work___1 6:00-6:15<br>2 weai_time_2_work___2 6:15-6:30<br>3 weai_time_2_work___3 6:30-6:45<br>4 weai_time_2_work___4 6:45-7:00<br>5 weai_time_2_work___5 7:00-7:15<br>6 weai_time_2_work___6 7:15-7:30<br>7 weai_time_2_work___7 7:30-7:45<br>8 weai_time_2_work___8 7:45-8:00
1065	weai_time_2_buisness	Own business work	checkbox<br>1 weai_time_2_buisness___1 6:00-6:15<br>2 weai_time_2_buisness___2 6:15-6:30<br>3 weai_time_2_buisness___3 6:30-6:45<br>4 weai_time_2_buisness___4 6:45-7:00<br>5 weai_time_2_buisness___5 7:00-7:15<br>6 weai_time_2_buisness___6 7:15-7:30<br>7 weai_time_2_buisness___7 7:30-7:45<br>8 weai_time_2_buisness___8 7:45-8:00

Havelaar AH, *et al. BMJ Open* 2022; 12:e061311. doi: 10.1136/bmjopen-2022-061311

1066	weai_time_2_farm	Farming/livestock/fishing	checkbox<br>1 weai_time_2_farm___1 6:00-6:15<br>2 weai_time_2_farm___2 6:15-6:30<br>3 weai_time_2_farm___3 6:30-6:45<br>4 weai_time_2_farm___4 6:45-7:00<br>5 weai_time_2_farm___5 7:00-7:15<br>6 weai_time_2_farm___6 7:15-7:30<br>7 weai_time_2_farm___7 7:30-7:45<br>8 weai_time_2_farm___8 7:45-8:00
1067	weai_time_2_shop	Shopping/getting service (incl health services)	checkbox<br>1 weai_time_2_shop___1 6:00-6:15<br>2 weai_time_2_shop___2 6:15-6:30<br>3 weai_time_2_shop___3 6:30-6:45<br>4 weai_time_2_shop___4 6:45-7:00<br>5 weai_time_2_shop___5 7:00-7:15<br>6 weai_time_2_shop___6 7:15-7:30<br>7 weai_time_2_shop___7 7:30-7:45<br>8 weai_time_2_shop___8 7:45-8:00
1068	weai_time_2_weave	Weaving, sewing, textile care	checkbox<br>1 weai_time_2_weave___1 6:00-6:15<br>2 weai_time_2_weave___2 6:15-6:30<br>3 weai_time_2_weave___3 6:30-6:45<br>4 weai_time_2_weave___4 6:45-7:00<br>5 weai_time_2_weave___5 7:00-7:15<br>6 weai_time_2_weave___6 7:15-7:30<br>7 weai_time_2_weave___7 7:30-7:45<br>8 weai_time_2_weave___8 7:45-8:00
1069	weai_time_2_cook	Cooking	checkbox<br>1 weai_time_2_cook___1 6:00-6:15<br>2 weai_time_2_cook___2 6:15-6:30<br>3 weai_time_2_cook___3 6:30-6:45<br>4 weai_time_2_cook___4 6:45-7:00<br>5 weai_time_2_cook___5 7:00-7:15<br>6 weai_time_2_cook___6 7:15-7:30<br>7 weai_time_2_cook___7 7:30-7:45<br>8 weai_time_2_cook___8 7:45-8:00
1070	weai_time_2_domestic	Domestic work (incl fetching wood and water)	checkbox<br>1 weai_time_2_domestic___1 6:00-6:15<br>2 weai_time_2_domestic___2 6:15-6:30<br>3 weai_time_2_domestic___3 6:30-6:45<br>4 weai_time_2_domestic___4 6:45-7:00<br>5 weai_time_2_domestic___5 7:00-7:15<br>6 weai_time_2_domestic___6 7:15-7:30<br>7 weai_time_2_domestic___7 7:30-7:45<br>8 weai_time_2_domestic___8 7:45-8:00

1071	weai_time_2_care	Care for children/adults/elderly	checkbox<br>1 weai_time_2_care___1 6:00-6:15<br>2 weai_time_2_care___2 6:15-6:30<br>3 weai_time_2_care___3 6:30-6:45<br>4 weai_time_2_care___4 6:45-7:00<br>5 weai_time_2_care___5 7:00-7:15<br>6 weai_time_2_care___6 7:15-7:30<br>7 weai_time_2_care___7 7:30-7:45<br>8 weai_time_2_care___8 7:45-8:00
1072	weai_time_2_travel	Travelling and communiting	checkbox<br>1 weai_time_2_travel___1 6:00-6:15<br>2 weai_time_2_travel___2 6:15-6:30<br>3 weai_time_2_travel___3 6:30-6:45<br>4 weai_time_2_travel___4 6:45-7:00<br>5 weai_time_2_travel___5 7:00-7:15<br>6 weai_time_2_travel___6 7:15-7:30<br>7 weai_time_2_travel___7 7:30-7:45<br>8 weai_time_2_travel___8 7:45-8:00
1073	weai_time_2_tv	Watching TV/listening to radio/reading	checkbox<br>1 weai_time_2_tv___1 6:00-6:15<br>2 weai_time_2_tv___2 6:15-6:30<br>3 weai_time_2_tv___3 6:30-6:45<br>4 weai_time_2_tv___4 6:45-7:00<br>5 weai_time_2_tv___5 7:00-7:15<br>6 weai_time_2_tv___6 7:15-7:30<br>7 weai_time_2_tv___7 7:30-7:45<br>8 weai_time_2_tv___8 7:45-8:00
1074	weai_time_2_exercising	Exercising	checkbox<br>1 weai_time_2_exercising___1 6:00-6:15<br>2 weai_time_2_exercising___2 6:15-6:30<br>3 weai_time_2_exercising___3 6:30-6:45<br>4 weai_time_2_exercising___4 6:45-7:00<br>5 weai_time_2_exercising___5 7:00-7:15<br>6 weai_time_2_exercising___6 7:15-7:30<br>7 weai_time_2_exercising___7 7:30-7:45<br>8 weai_time_2_exercising___8 7:45-8:00
1075	weai_time_2_social	Social activities and hobbies	checkbox<br>1 weai_time_2_social___1 6:00-6:15<br>2 weai_time_2_social___2 6:15-6:30<br>3 weai_time_2_social___3 6:30-6:45<br>4 weai_time_2_social___4 6:45-7:00<br>5 weai_time_2_social___5 7:00-7:15<br>6 weai_time_2_social___6 7:15-7:30<br>7 weai_time_2_social___7 7:30-7:45<br>8 weai_time_2_social___8 7:45-8:00

Havelaar AH, *et al. BMJ Open* 2022; 12:e061311. doi: 10.1136/bmjopen-2022-061311

Supplemental material BMJ Publishing Group Limited (BMJ) disclaims all liability and responsibility arising from any reliance placed on this supplemental material which has been supplied by the author(s) BMJ Open

1076	weai_time_2_religious	Religious activities	checkbox<br>1 weai_time_2_religious___1 6:00-6:15<br>2 weai_time_2_religious___2 6:15-6:30<br>3 weai_time_2_religious___3 6:30-6:45<br>4 weai_time_2_religious___4 6:45-7:00<br>5 weai_time_2_religious___5 7:00-7:15<br>6 weai_time_2_religious___6 7:15-7:30<br>7 weai_time_2_religious___7 7:30-7:45<br>8 weai_time_2_religious___8 7:45-8:00
1077	weai_time_2_other	Other (specify below)	checkbox<br>1 weai_time_2_other___1 6:00-6:15<br>2 weai_time_2_other___2 6:15-6:30<br>3 weai_time_2_other___3 6:30-6:45<br>4 weai_time_2_other___4 6:45-7:00<br>5 weai_time_2_other___5 7:00-7:15<br>6 weai_time_2_other___6 7:15-7:30<br>7 weai_time_2_other___7 7:30-7:45<br>8 weai_time_2_other___8 7:45-8:00
1078	weai_time_2_other_specify<br>Show the field ONLY if: [weai_time_2_other(1)] or [weai_time_2_other(2)] or [weai_time_2_other(3)] or [weai_time_2_other(4)] or [weai_time_2_other(5)] or [weai_time_2_other(6)] or [weai_time_2_other(7)] or [weai_time_2_other(8)]	Specify Other	text<br>Custom alignment: LV
1079	weai_time_3_sleep	Section Header: *Early Day*<br>Sleeping and resting	checkbox<br>1 weai_time_3_sleep___1 8:00-8:15<br>2 weai_time_3_sleep___2 8:15-8:30<br>3 weai_time_3_sleep___3 8:30-8:45<br>4 weai_time_3_sleep___4 8:45-9:00<br>5 weai_time_3_sleep___5 9:00-9:15<br>6 weai_time_3_sleep___6 9:15-9:30<br>7 weai_time_3_sleep___7 9:30-9:45<br>8 weai_time_3_sleep___8 9:45-10:00
1080	weai_time_3_eat	Eating and drinking	checkbox<br>1 weai_time_3_eat___1 8:00-8:15<br>2 weai_time_3_eat___2 8:15-8:30<br>3 weai_time_3_eat___3 8:30-8:45<br>4 weai_time_3_eat___4 8:45-9:00<br>5 weai_time_3_eat___5 9:00-9:15<br>6 weai_time_3_eat___6 9:15-9:30<br>7 weai_time_3_eat___7 9:30-9:45<br>8 weai_time_3_eat___8 9:45-10:00

Havelaar AH, *et al. BMJ Open* 2022; 12:e061311. doi: 10.1136/bmjopen-2022-061311

1081	weai_time_3_personal	Personal Care	checkbox<br>1 weai_time_3_personal___1 8:00-8:15<br>2 weai_time_3_personal___2 8:15-8:30<br>3 weai_time_3_personal___3 8:30-8:45<br>4 weai_time_3_personal___4 8:45-9:00<br>5 weai_time_3_personal___5 9:00-9:15<br>6 weai_time_3_personal___6 9:15-9:30<br>7 weai_time_3_personal___7 9:30-9:45<br>8 weai_time_3_personal___8 9:45-10:00
1082	weai_time_3_school	School (also homework)	checkbox<br>1 weai_time_3_school___1 8:00-8:15<br>2 weai_time_3_school___2 8:15-8:30<br>3 weai_time_3_school___3 8:30-8:45<br>4 weai_time_3_school___4 8:45-9:00<br>5 weai_time_3_school___5 9:00-9:15<br>6 weai_time_3_school___6 9:15-9:30<br>7 weai_time_3_school___7 9:30-9:45<br>8 weai_time_3_school___8 9:45-10:00
1083	weai_time_3_work	Work as employed	checkbox<br>1 weai_time_3_work___1 8:00-8:15<br>2 weai_time_3_work___2 8:15-8:30<br>3 weai_time_3_work___3 8:30-8:45<br>4 weai_time_3_work___4 8:45-9:00<br>5 weai_time_3_work___5 9:00-9:15<br>6 weai_time_3_work___6 9:15-9:30<br>7 weai_time_3_work___7 9:30-9:45<br>8 weai_time_3_work___8 9:45-10:00
1084	weai_time_3_buisness	Own business work	checkbox<br>1 weai_time_3_buisness___1 8:00-8:15<br>2 weai_time_3_buisness___2 8:15-8:30<br>3 weai_time_3_buisness___3 8:30-8:45<br>4 weai_time_3_buisness___4 8:45-9:00<br>5 weai_time_3_buisness___5 9:00-9:15<br>6 weai_time_3_buisness___6 9:15-9:30<br>7 weai_time_3_buisness___7 9:30-9:45<br>8 weai_time_3_buisness___8 9:45-10:00
1085	weai_time_3_farm	Farming/livestock/fishing	checkbox<br>1 weai_time_3_farm___1 8:00-8:15<br>2 weai_time_3_farm___2 8:15-8:30<br>3 weai_time_3_farm___3 8:30-8:45<br>4 weai_time_3_farm___4 8:45-9:00<br>5 weai_time_3_farm___5 9:00-9:15<br>6 weai_time_3_farm___6 9:15-9:30<br>7 weai_time_3_farm___7 9:30-9:45<br>8 weai_time_3_farm___8 9:45-10:00

1086	weai_time_3_shop	Shopping/getting service (incl health services)	checkbox<br>1 weai_time_3_shop___1 8:00-8:15<br>2 weai_time_3_shop___2 8:15-8:30<br>3 weai_time_3_shop___3 8:30-8:45<br>4 weai_time_3_shop___4 8:45-9:00<br>5 weai_time_3_shop___5 9:00-9:15<br>6 weai_time_3_shop___6 9:15-9:30<br>7 weai_time_3_shop___7 9:30-9:45<br>8 weai_time_3_shop___8 9:45-10:00
1087	weai_time_3_weave	Weaving, sewing, textile care	checkbox<br>1 weai_time_3_weave___1 8:00-8:15<br>2 weai_time_3_weave___2 8:15-8:30<br>3 weai_time_3_weave___3 8:30-8:45<br>4 weai_time_3_weave___4 8:45-9:00<br>5 weai_time_3_weave___5 9:00-9:15<br>6 weai_time_3_weave___6 9:15-9:30<br>7 weai_time_3_weave___7 9:30-9:45<br>8 weai_time_3_weave___8 9:45-10:00
1088	weai_time_3_cook	Cooking	checkbox<br>1 weai_time_3_cook___1 8:00-8:15<br>2 weai_time_3_cook___2 8:15-8:30<br>3 weai_time_3_cook___3 8:30-8:45<br>4 weai_time_3_cook___4 8:45-9:00<br>5 weai_time_3_cook___5 9:00-9:15<br>6 weai_time_3_cook___6 9:15-9:30<br>7 weai_time_3_cook___7 9:30-9:45<br>8 weai_time_3_cook___8 9:45-10:00
1089	weai_time_3_domestic	Domestic work (incl fetching wood and water)	checkbox<br>1 weai_time_3_domestic___1 8:00-8:15<br>2 weai_time_3_domestic___2 8:15-8:30<br>3 weai_time_3_domestic___3 8:30-8:45<br>4 weai_time_3_domestic___4 8:45-9:00<br>5 weai_time_3_domestic___5 9:00-9:15<br>6 weai_time_3_domestic___6 9:15-9:30<br>7 weai_time_3_domestic___7 9:30-9:45<br>8 weai_time_3_domestic___8 9:45-10:00
1090	weai_time_3_care	Care for children/adults/elderly	checkbox<br>1 weai_time_3_care___1 8:00-8:15<br>2 weai_time_3_care___2 8:15-8:30<br>3 weai_time_3_care___3 8:30-8:45<br>4 weai_time_3_care___4 8:45-9:00<br>5 weai_time_3_care___5 9:00-9:15<br>6 weai_time_3_care___6 9:15-9:30<br>7 weai_time_3_care___7 9:30-9:45<br>8 weai_time_3_care___8 9:45-10:00

Havelaar AH, *et al. BMJ Open* 2022; 12:e061311. doi: 10.1136/bmjopen-2022-061311

1091	weai_time_3_travel	Travelling and communiting	checkbox<br>1 weai_time_3_travel___1 8:00-8:15<br>2 weai_time_3_travel___2 8:15-8:30<br>3 weai_time_3_travel___3 8:30-8:45<br>4 weai_time_3_travel___4 8:45-9:00<br>5 weai_time_3_travel___5 9:00-9:15<br>6 weai_time_3_travel___6 9:15-9:30<br>7 weai_time_3_travel___7 9:30-9:45<br>8 weai_time_3_travel___8 9:45-10:00
1092	weai_time_3_tv	Watching TV/listening to radio/reading	checkbox<br>1 weai_time_3_tv___1 8:00-8:15<br>2 weai_time_3_tv___2 8:15-8:30<br>3 weai_time_3_tv___3 8:30-8:45<br>4 weai_time_3_tv___4 8:45-9:00<br>5 weai_time_3_tv___5 9:00-9:15<br>6 weai_time_3_tv___6 9:15-9:30<br>7 weai_time_3_tv___7 9:30-9:45<br>8 weai_time_3_tv___8 9:45-10:00
1093	weai_time_3_exercising	Exercising	checkbox<br>1 weai_time_3_exercising___1 8:00-8:15<br>2 weai_time_3_exercising___2 8:15-8:30<br>3 weai_time_3_exercising___3 8:30-8:45<br>4 weai_time_3_exercising___4 8:45-9:00<br>5 weai_time_3_exercising___5 9:00-9:15<br>6 weai_time_3_exercising___6 9:15-9:30<br>7 weai_time_3_exercising___7 9:30-9:45<br>8 weai_time_3_exercising___8 9:45-10:00
1094	weai_time_3_social	Social activities and hobbies	checkbox<br>1 weai_time_3_social___1 8:00-8:15<br>2 weai_time_3_social___2 8:15-8:30<br>3 weai_time_3_social___3 8:30-8:45<br>4 weai_time_3_social___4 8:45-9:00<br>5 weai_time_3_social___5 9:00-9:15<br>6 weai_time_3_social___6 9:15-9:30<br>7 weai_time_3_social___7 9:30-9:45<br>8 weai_time_3_social___8 9:45-10:00
1095	weai_time_3_religious	Religious activities	checkbox<br>1 weai_time_3_religious___1 8:00-8:15<br>2 weai_time_3_religious___2 8:15-8:30<br>3 weai_time_3_religious___3 8:30-8:45<br>4 weai_time_3_religious___4 8:45-9:00<br>5 weai_time_3_religious___5 9:00-9:15<br>6 weai_time_3_religious___6 9:15-9:30<br>7 weai_time_3_religious___7 9:30-9:45<br>8 weai_time_3_religious___8 9:45-10:00

Havelaar AH, *et al. BMJ Open* 2022; 12:e061311. doi: 10.1136/bmjopen-2022-061311

1096	weai_time_3_other	Other (specify below)	checkbox<br>1 weai_time_3_other___1 8:00-8:15<br>2 weai_time_3_other___2 8:15-8:30<br>3 weai_time_3_other___3 8:30-8:45<br>4 weai_time_3_other___4 8:45-9:00<br>5 weai_time_3_other___5 9:00-9:15<br>6 weai_time_3_other___6 9:15-9:30<br>7 weai_time_3_other___7 9:30-9:45<br>8 weai_time_3_other___8 9:45-10:00
1097	weai_time_3_other_specify<br>Show the field ONLY if:<br>[weai_time_3_other(1)] or [weai_time_3_other(2)] or [weai_time_3_other(3)] or [weai_time_3_other(4)] or [weai_time_3_other(5)] or [weai_time_3_other(6)] or [weai_time_3_other(7)] or [weai_time_3_other(8)]	Specify Other	text<br>Custom alignment: LV
1098	weai_time_4_sleep	Section Header: *Late Day*<br>Sleeping and resting	checkbox<br>1 weai_time_4_sleep___1 10:00-10:15<br>2 weai_time_4_sleep___2 10:15-10:30<br>3 weai_time_4_sleep___3 10:30-10:45<br>4 weai_time_4_sleep___4 10:45-11:00<br>5 weai_time_4_sleep___5 11:00-11:15<br>6 weai_time_4_sleep___6 11:15-11:30<br>7 weai_time_4_sleep___7 11:30-11:45<br>8 weai_time_4_sleep___8 11:45-12:00
1099	weai_time_4_eat	Eating and drinking	checkbox<br>1 weai_time_4_eat___1 10:00-10:15<br>2 weai_time_4_eat___2 10:15-10:30<br>3 weai_time_4_eat___3 10:30-10:45<br>4 weai_time_4_eat___4 10:45-11:00<br>5 weai_time_4_eat___5 11:00-11:15<br>6 weai_time_4_eat___6 11:15-11:30<br>7 weai_time_4_eat___7 11:30-11:45<br>8 weai_time_4_eat___8 11:45-12:00
1100	weai_time_4_personal	Personal Care	checkbox<br>1 weai_time_4_personal___1 10:00-10:15<br>2 weai_time_4_personal___2 10:15-10:30<br>3 weai_time_4_personal___3 10:30-10:45<br>4 weai_time_4_personal___4 10:45-11:00<br>5 weai_time_4_personal___5 11:00-11:15<br>6 weai_time_4_personal___6 11:15-11:30<br>7 weai_time_4_personal___7 11:30-11:45<br>8 weai_time_4_personal___8 11:45-12:00

1101	weai_time_4_school	School (also homework)	checkbox	1	weai_time_4_school___1	10:00-10:15
				2	weai_time_4_school___2	10:15-10:30
				3	weai_time_4_school___3	10:30-10:45
				4	weai_time_4_school___4	10:45-11:00
				5	weai_time_4_school___5	11:00-11:15
				6	weai_time_4_school___6	11:15-11:30
				7	weai_time_4_school___7	11:30-11:45
				8	weai_time_4_school___8	11:45-12:00
1102	weai_time_4_work	Work as employed	checkbox	1	weai_time_4_work___1	10:00-10:15
				2	weai_time_4_work___2	10:15-10:30
				3	weai_time_4_work___3	10:30-10:45
				4	weai_time_4_work___4	10:45-11:00
				5	weai_time_4_work___5	11:00-11:15
				6	weai_time_4_work___6	11:15-11:30
				7	weai_time_4_work___7	11:30-11:45
				8	weai_time_4_work___8	11:45-12:00
1103	weai_time_4_buisness	Own business work	checkbox	1	weai_time_4_buisness___1	10:00-10:15
				2	weai_time_4_buisness___2	10:15-10:30
				3	weai_time_4_buisness___3	10:30-10:45
				4	weai_time_4_buisness___4	10:45-11:00
				5	weai_time_4_buisness___5	11:00-11:15
				6	weai_time_4_buisness___6	11:15-11:30
				7	weai_time_4_buisness___7	11:30-11:45
				8	weai_time_4_buisness___8	11:45-12:00
1104	weai_time_4_farm	Farming/livestock/fishing	checkbox	1	weai_time_4_farm___1	10:00-10:15
				2	weai_time_4_farm___2	10:15-10:30
				3	weai_time_4_farm___3	10:30-10:45
				4	weai_time_4_farm___4	10:45-11:00
				5	weai_time_4_farm___5	11:00-11:15
				6	weai_time_4_farm___6	11:15-11:30
				7	weai_time_4_farm___7	11:30-11:45
				8	weai_time_4_farm___8	11:45-12:00
1105	weai_time_4_shop	Shopping/getting service (incl health services)	checkbox	1	weai_time_4_shop___1	10:00-10:15
				2	weai_time_4_shop___2	10:15-10:30
				3	weai_time_4_shop___3	10:30-10:45
				4	weai_time_4_shop___4	10:45-11:00
				5	weai_time_4_shop___5	11:00-11:15
				6	weai_time_4_shop___6	11:15-11:30
				7	weai_time_4_shop___7	11:30-11:45
				8	weai_time_4_shop___8	11:45-12:00

Supplemental material BMJ Publishing Group Limited (BMJ) disclaims all liability and responsibility arising from any reliance placed on this supplemental material which has been supplied by the author(s) BMJ Open

1106	weai_time_4_weave	Weaving, sewing, textile care	checkbox<br>1 weai_time_4_weave___1 10:00-10:15<br>2 weai_time_4_weave___2 10:15-10:30<br>3 weai_time_4_weave___3 10:30-10:45<br>4 weai_time_4_weave___4 10:45-11:00<br>5 weai_time_4_weave___5 11:00-11:15<br>6 weai_time_4_weave___6 11:15-11:30<br>7 weai_time_4_weave___7 11:30-11:45<br>8 weai_time_4_weave___8 11:45-12:00
1107	weai_time_4_cook	Cooking	checkbox<br>1 weai_time_4_cook___1 10:00-10:15<br>2 weai_time_4_cook___2 10:15-10:30<br>3 weai_time_4_cook___3 10:30-10:45<br>4 weai_time_4_cook___4 10:45-11:00<br>5 weai_time_4_cook___5 11:00-11:15<br>6 weai_time_4_cook___6 11:15-11:30<br>7 weai_time_4_cook___7 11:30-11:45<br>8 weai_time_4_cook___8 11:45-12:00
1108	weai_time_4_domestic	Domestic work (incl fetching wood and water)	checkbox<br>1 weai_time_4_domestic___1 10:00-10:15<br>2 weai_time_4_domestic___2 10:15-10:30<br>3 weai_time_4_domestic___3 10:30-10:45<br>4 weai_time_4_domestic___4 10:45-11:00<br>5 weai_time_4_domestic___5 11:00-11:15<br>6 weai_time_4_domestic___6 11:15-11:30<br>7 weai_time_4_domestic___7 11:30-11:45<br>8 weai_time_4_domestic___8 11:45-12:00
1109	weai_time_4_care	Care for children/adults/elderly	checkbox<br>1 weai_time_4_care___1 10:00-10:15<br>2 weai_time_4_care___2 10:15-10:30<br>3 weai_time_4_care___3 10:30-10:45<br>4 weai_time_4_care___4 10:45-11:00<br>5 weai_time_4_care___5 11:00-11:15<br>6 weai_time_4_care___6 11:15-11:30<br>7 weai_time_4_care___7 11:30-11:45<br>8 weai_time_4_care___8 11:45-12:00
1110	weai_time_4_travel	Travelling and communiting	checkbox<br>1 weai_time_4_travel___1 10:00-10:15<br>2 weai_time_4_travel___2 10:15-10:30<br>3 weai_time_4_travel___3 10:30-10:45<br>4 weai_time_4_travel___4 10:45-11:00<br>5 weai_time_4_travel___5 11:00-11:15<br>6 weai_time_4_travel___6 11:15-11:30<br>7 weai_time_4_travel___7 11:30-11:45<br>8 weai_time_4_travel___8 11:45-12:00

Havelaar AH, *et al. BMJ Open* 2022; 12:e061311. doi: 10.1136/bmjopen-2022-061311

<table>
<tr><td>1111</td><td>weai_time_4_tv</td><td>Watching TV/listening to radio/reading</td><td>checkbox<table>
<tr><td>1</td><td>weai_time_4_tv___1</td><td>10:00-10:15</td></tr>
<tr><td>2</td><td>weai_time_4_tv___2</td><td>10:15-10:30</td></tr>
<tr><td>3</td><td>weai_time_4_tv___3</td><td>10:30-10:45</td></tr>
<tr><td>4</td><td>weai_time_4_tv___4</td><td>10:45-11:00</td></tr>
<tr><td>5</td><td>weai_time_4_tv___5</td><td>11:00-11:15</td></tr>
<tr><td>6</td><td>weai_time_4_tv___6</td><td>11:15-11:30</td></tr>
<tr><td>7</td><td>weai_time_4_tv___7</td><td>11:30-11:45</td></tr>
<tr><td>8</td><td>weai_time_4_tv___8</td><td>11:45-12:00</td></tr>
</table></td></tr>
<tr><td>1112</td><td>weai_time_4_exercising</td><td>Exercising</td><td>checkbox<table>
<tr><td>1</td><td>weai_time_4_exercising___1</td><td>10:00-10:15</td></tr>
<tr><td>2</td><td>weai_time_4_exercising___2</td><td>10:15-10:30</td></tr>
<tr><td>3</td><td>weai_time_4_exercising___3</td><td>10:30-10:45</td></tr>
<tr><td>4</td><td>weai_time_4_exercising___4</td><td>10:45-11:00</td></tr>
<tr><td>5</td><td>weai_time_4_exercising___5</td><td>11:00-11:15</td></tr>
<tr><td>6</td><td>weai_time_4_exercising___6</td><td>11:15-11:30</td></tr>
<tr><td>7</td><td>weai_time_4_exercising___7</td><td>11:30-11:45</td></tr>
<tr><td>8</td><td>weai_time_4_exercising___8</td><td>11:45-12:00</td></tr>
</table></td></tr>
<tr><td>1113</td><td>weai_time_4_social</td><td>Social activities and hobbies</td><td>checkbox<table>
<tr><td>1</td><td>weai_time_4_social___1</td><td>10:00-10:15</td></tr>
<tr><td>2</td><td>weai_time_4_social___2</td><td>10:15-10:30</td></tr>
<tr><td>3</td><td>weai_time_4_social___3</td><td>10:30-10:45</td></tr>
<tr><td>4</td><td>weai_time_4_social___4</td><td>10:45-11:00</td></tr>
<tr><td>5</td><td>weai_time_4_social___5</td><td>11:00-11:15</td></tr>
<tr><td>6</td><td>weai_time_4_social___6</td><td>11:15-11:30</td></tr>
<tr><td>7</td><td>weai_time_4_social___7</td><td>11:30-11:45</td></tr>
<tr><td>8</td><td>weai_time_4_social___8</td><td>11:45-12:00</td></tr>
</table></td></tr>
<tr><td>1114</td><td>weai_time_4_religious</td><td>Religious activities</td><td>checkbox<table>
<tr><td>1</td><td>weai_time_4_religious___1</td><td>10:00-10:15</td></tr>
<tr><td>2</td><td>weai_time_4_religious___2</td><td>10:15-10:30</td></tr>
<tr><td>3</td><td>weai_time_4_religious___3</td><td>10:30-10:45</td></tr>
<tr><td>4</td><td>weai_time_4_religious___4</td><td>10:45-11:00</td></tr>
<tr><td>5</td><td>weai_time_4_religious___5</td><td>11:00-11:15</td></tr>
<tr><td>6</td><td>weai_time_4_religious___6</td><td>11:15-11:30</td></tr>
<tr><td>7</td><td>weai_time_4_religious___7</td><td>11:30-11:45</td></tr>
<tr><td>8</td><td>weai_time_4_religious___8</td><td>11:45-12:00</td></tr>
</table></td></tr>
<tr><td>1115</td><td>weai_time_4_other</td><td>Other (specify below)</td><td>checkbox<table>
<tr><td>1</td><td>weai_time_4_other___1</td><td>10:00-10:15</td></tr>
<tr><td>2</td><td>weai_time_4_other___2</td><td>10:15-10:30</td></tr>
<tr><td>3</td><td>weai_time_4_other___3</td><td>10:30-10:45</td></tr>
<tr><td>4</td><td>weai_time_4_other___4</td><td>10:45-11:00</td></tr>
<tr><td>5</td><td>weai_time_4_other___5</td><td>11:00-11:15</td></tr>
<tr><td>6</td><td>weai_time_4_other___6</td><td>11:15-11:30</td></tr>
<tr><td>7</td><td>weai_time_4_other___7</td><td>11:30-11:45</td></tr>
<tr><td>8</td><td>weai_time_4_other___8</td><td>11:45-12:00</td></tr>
</table></td></tr>
</table>

Havelaar AH, et al. BMJ Open 2022; 12:e061311. doi: 10.1136/bmjopen-2022-061311

1116	weai_time_4_other_specify<br><br>Show the field ONLY if: [weai_time_4_other(1)] or [weai_time_4_other(2)] or [weai_time_4_other(3)] or [weai_time_4_other(4)] or [weai_time_4_other(5)] or [weai_time_4_other(6)] or [weai_time_4_other(7)] or [weai_time_4_other(8)]	Specify Other	text<br>Custom alignment: LV
1117	weai_time_5_sleep	Section Header: *Early Evening*<br>Sleeping and resting	checkbox<br>1 weai_time_5_sleep___1 12:00-12:15<br>2 weai_time_5_sleep___2 12:15-12:30<br>3 weai_time_5_sleep___3 12:30-12:45<br>4 weai_time_5_sleep___4 12:45-13:00<br>5 weai_time_5_sleep___5 13:00-13:15<br>6 weai_time_5_sleep___6 13:15-13:30<br>7 weai_time_5_sleep___7 13:30-13:45<br>8 weai_time_5_sleep___8 13:45-14:00
1118	weai_time_5_eat	Eating and drinking	checkbox<br>1 weai_time_5_eat___1 12:00-12:15<br>2 weai_time_5_eat___2 12:15-12:30<br>3 weai_time_5_eat___3 12:30-12:45<br>4 weai_time_5_eat___4 12:45-13:00<br>5 weai_time_5_eat___5 13:00-13:15<br>6 weai_time_5_eat___6 13:15-13:30<br>7 weai_time_5_eat___7 13:30-13:45<br>8 weai_time_5_eat___8 13:45-14:00
1119	weai_time_5_personal	Personal Care	checkbox<br>1 weai_time_5_personal___1 12:00-12:15<br>2 weai_time_5_personal___2 12:15-12:30<br>3 weai_time_5_personal___3 12:30-12:45<br>4 weai_time_5_personal___4 12:45-13:00<br>5 weai_time_5_personal___5 13:00-13:15<br>6 weai_time_5_personal___6 13:15-13:30<br>7 weai_time_5_personal___7 13:30-13:45<br>8 weai_time_5_personal___8 13:45-14:00
1120	weai_time_5_school	School (also homework)	checkbox<br>1 weai_time_5_school___1 12:00-12:15<br>2 weai_time_5_school___2 12:15-12:30<br>3 weai_time_5_school___3 12:30-12:45<br>4 weai_time_5_school___4 12:45-13:00<br>5 weai_time_5_school___5 13:00-13:15<br>6 weai_time_5_school___6 13:15-13:30<br>7 weai_time_5_school___7 13:30-13:45<br>8 weai_time_5_school___8 13:45-14:00

1121	weai_time_5_work	Work as employed	checkbox<br>1 weai_time_5_work___1 12:00-12:15<br>2 weai_time_5_work___2 12:15-12:30<br>3 weai_time_5_work___3 12:30-12:45<br>4 weai_time_5_work___4 12:45-13:00<br>5 weai_time_5_work___5 13:00-13:15<br>6 weai_time_5_work___6 13:15-13:30<br>7 weai_time_5_work___7 13:30-13:45<br>8 weai_time_5_work___8 13:45-14:00
1122	weai_time_5_buisness	Own business work	checkbox<br>1 weai_time_5_buisness___1 12:00-12:15<br>2 weai_time_5_buisness___2 12:15-12:30<br>3 weai_time_5_buisness___3 12:30-12:45<br>4 weai_time_5_buisness___4 12:45-13:00<br>5 weai_time_5_buisness___5 13:00-13:15<br>6 weai_time_5_buisness___6 13:15-13:30<br>7 weai_time_5_buisness___7 13:30-13:45<br>8 weai_time_5_buisness___8 13:45-14:00
1123	weai_time_5_farm	Farming/livestock/fishing	checkbox<br>1 weai_time_5_farm___1 12:00-12:15<br>2 weai_time_5_farm___2 12:15-12:30<br>3 weai_time_5_farm___3 12:30-12:45<br>4 weai_time_5_farm___4 12:45-13:00<br>5 weai_time_5_farm___5 13:00-13:15<br>6 weai_time_5_farm___6 13:15-13:30<br>7 weai_time_5_farm___7 13:30-13:45<br>8 weai_time_5_farm___8 13:45-14:00
1124	weai_time_5_shop	Shopping/getting service (incl health services)	checkbox<br>1 weai_time_5_shop___1 12:00-12:15<br>2 weai_time_5_shop___2 12:15-12:30<br>3 weai_time_5_shop___3 12:30-12:45<br>4 weai_time_5_shop___4 12:45-13:00<br>5 weai_time_5_shop___5 13:00-13:15<br>6 weai_time_5_shop___6 13:15-13:30<br>7 weai_time_5_shop___7 13:30-13:45<br>8 weai_time_5_shop___8 13:45-14:00
1125	weai_time_5_weave	Weaving, sewing, textile care	checkbox<br>1 weai_time_5_weave___1 12:00-12:15<br>2 weai_time_5_weave___2 12:15-12:30<br>3 weai_time_5_weave___3 12:30-12:45<br>4 weai_time_5_weave___4 12:45-13:00<br>5 weai_time_5_weave___5 13:00-13:15<br>6 weai_time_5_weave___6 13:15-13:30<br>7 weai_time_5_weave___7 13:30-13:45<br>8 weai_time_5_weave___8 13:45-14:00

1126	weai_time_5_cook	Cooking	checkbox<br>1 weai_time_5_cook___1 12:00-12:15<br>2 weai_time_5_cook___2 12:15-12:30<br>3 weai_time_5_cook___3 12:30-12:45<br>4 weai_time_5_cook___4 12:45-13:00<br>5 weai_time_5_cook___5 13:00-13:15<br>6 weai_time_5_cook___6 13:15-13:30<br>7 weai_time_5_cook___7 13:30-13:45<br>8 weai_time_5_cook___8 13:45-14:00
1127	weai_time_5_domestic	Domestic work (incl fetching wood and water)	checkbox<br>1 weai_time_5_domestic___1 12:00-12:15<br>2 weai_time_5_domestic___2 12:15-12:30<br>3 weai_time_5_domestic___3 12:30-12:45<br>4 weai_time_5_domestic___4 12:45-13:00<br>5 weai_time_5_domestic___5 13:00-13:15<br>6 weai_time_5_domestic___6 13:15-13:30<br>7 weai_time_5_domestic___7 13:30-13:45<br>8 weai_time_5_domestic___8 13:45-14:00
1128	weai_time_5_care	Care for children/adults/elderly	checkbox<br>1 weai_time_5_care___1 12:00-12:15<br>2 weai_time_5_care___2 12:15-12:30<br>3 weai_time_5_care___3 12:30-12:45<br>4 weai_time_5_care___4 12:45-13:00<br>5 weai_time_5_care___5 13:00-13:15<br>6 weai_time_5_care___6 13:15-13:30<br>7 weai_time_5_care___7 13:30-13:45<br>8 weai_time_5_care___8 13:45-14:00
1129	weai_time_5_travel	Travelling and communiting	checkbox<br>1 weai_time_5_travel___1 12:00-12:15<br>2 weai_time_5_travel___2 12:15-12:30<br>3 weai_time_5_travel___3 12:30-12:45<br>4 weai_time_5_travel___4 12:45-13:00<br>5 weai_time_5_travel___5 13:00-13:15<br>6 weai_time_5_travel___6 13:15-13:30<br>7 weai_time_5_travel___7 13:30-13:45<br>8 weai_time_5_travel___8 13:45-14:00
1130	weai_time_5_tv	Watching TV/listening to radio/reading	checkbox<br>1 weai_time_5_tv___1 12:00-12:15<br>2 weai_time_5_tv___2 12:15-12:30<br>3 weai_time_5_tv___3 12:30-12:45<br>4 weai_time_5_tv___4 12:45-13:00<br>5 weai_time_5_tv___5 13:00-13:15<br>6 weai_time_5_tv___6 13:15-13:30<br>7 weai_time_5_tv___7 13:30-13:45<br>8 weai_time_5_tv___8 13:45-14:00

1131	weai_time_5_exercising	Exercising	checkbox<br>1 weai_time_5_exercising___1 12:00-12:15<br>2 weai_time_5_exercising___2 12:15-12:30<br>3 weai_time_5_exercising___3 12:30-12:45<br>4 weai_time_5_exercising___4 12:45-13:00<br>5 weai_time_5_exercising___5 13:00-13:15<br>6 weai_time_5_exercising___6 13:15-13:30<br>7 weai_time_5_exercising___7 13:30-13:45<br>8 weai_time_5_exercising___8 13:45-14:00
1132	weai_time_5_social	Social activities and hobbies	checkbox<br>1 weai_time_5_social___1 12:00-12:15<br>2 weai_time_5_social___2 12:15-12:30<br>3 weai_time_5_social___3 12:30-12:45<br>4 weai_time_5_social___4 12:45-13:00<br>5 weai_time_5_social___5 13:00-13:15<br>6 weai_time_5_social___6 13:15-13:30<br>7 weai_time_5_social___7 13:30-13:45<br>8 weai_time_5_social___8 13:45-14:00
1133	weai_time_5_religious	Religious activities	checkbox<br>1 weai_time_5_religious___1 12:00-12:15<br>2 weai_time_5_religious___2 12:15-12:30<br>3 weai_time_5_religious___3 12:30-12:45<br>4 weai_time_5_religious___4 12:45-13:00<br>5 weai_time_5_religious___5 13:00-13:15<br>6 weai_time_5_religious___6 13:15-13:30<br>7 weai_time_5_religious___7 13:30-13:45<br>8 weai_time_5_religious___8 13:45-14:00
1134	weai_time_5_other	Other (specify below)	checkbox<br>1 weai_time_5_other___1 12:00-12:15<br>2 weai_time_5_other___2 12:15-12:30<br>3 weai_time_5_other___3 12:30-12:45<br>4 weai_time_5_other___4 12:45-13:00<br>5 weai_time_5_other___5 13:00-13:15<br>6 weai_time_5_other___6 13:15-13:30<br>7 weai_time_5_other___7 13:30-13:45<br>8 weai_time_5_other___8 13:45-14:00
1135	weai_time_5_other_specify<br>Show the field ONLY if: [weai_time_5_other(1)] or [weai_time_5_other(2)] or [weai_time_5_other(3)] or [weai_time_5_other(4)] or [weai_time_5_other(5)] or [weai_time_5_other(6)] or [weai_time_5_other(7)] or [weai_time_5_other(8)]	Specify Other	text<br>Custom alignment: LV

1136	weai_time_6_sleep	Section Header: *Late Evening/Night*<br>Sleeping and resting	checkbox<br>1 weai_time_6_sleep___1 14:00-14:15<br>2 weai_time_6_sleep___2 14:15-14:30<br>3 weai_time_6_sleep___3 14:30-14:45<br>4 weai_time_6_sleep___4 14:45-15:00<br>5 weai_time_6_sleep___5 15:00-15:15<br>6 weai_time_6_sleep___6 15:15-15:30<br>7 weai_time_6_sleep___7 15:30-15:45<br>8 weai_time_6_sleep___8 15:45-16:00
1137	weai_time_6_eat	Eating and drinking	checkbox<br>1 weai_time_6_eat___1 14:00-14:15<br>2 weai_time_6_eat___2 14:15-14:30<br>3 weai_time_6_eat___3 14:30-14:45<br>4 weai_time_6_eat___4 14:45-15:00<br>5 weai_time_6_eat___5 15:00-15:15<br>6 weai_time_6_eat___6 15:15-15:30<br>7 weai_time_6_eat___7 15:30-15:45<br>8 weai_time_6_eat___8 15:45-16:00
1138	weai_time_6_personal	Personal Care	checkbox<br>1 weai_time_6_personal___1 14:00-14:15<br>2 weai_time_6_personal___2 14:15-14:30<br>3 weai_time_6_personal___3 14:30-14:45<br>4 weai_time_6_personal___4 14:45-15:00<br>5 weai_time_6_personal___5 15:00-15:15<br>6 weai_time_6_personal___6 15:15-15:30<br>7 weai_time_6_personal___7 15:30-15:45<br>8 weai_time_6_personal___8 15:45-16:00
1139	weai_time_6_school	School (also homework)	checkbox<br>1 weai_time_6_school___1 14:00-14:15<br>2 weai_time_6_school___2 14:15-14:30<br>3 weai_time_6_school___3 14:30-14:45<br>4 weai_time_6_school___4 14:45-15:00<br>5 weai_time_6_school___5 15:00-15:15<br>6 weai_time_6_school___6 15:15-15:30<br>7 weai_time_6_school___7 15:30-15:45<br>8 weai_time_6_school___8 15:45-16:00
1140	weai_time_6_work	Work as employed	checkbox<br>1 weai_time_6_work___1 14:00-14:15<br>2 weai_time_6_work___2 14:15-14:30<br>3 weai_time_6_work___3 14:30-14:45<br>4 weai_time_6_work___4 14:45-15:00<br>5 weai_time_6_work___5 15:00-15:15<br>6 weai_time_6_work___6 15:15-15:30<br>7 weai_time_6_work___7 15:30-15:45<br>8 weai_time_6_work___8 15:45-16:00

Havelaar AH, *et al. BMJ Open* 2022; 12:e061311. doi: 10.1136/bmjopen-2022-061311

Supplemental material
BMJ Publishing Group Limited (BMJ) disclaims all liability and responsibility arising from any reliance placed on this supplemental material which has been supplied by the author(s)
BMJ Open

1141	weai_time_6_buisness	Own business work	checkbox<br>1 weai_time_6_buisness___1 14:00-14:15<br>2 weai_time_6_buisness___2 14:15-14:30<br>3 weai_time_6_buisness___3 14:30-14:45<br>4 weai_time_6_buisness___4 14:45-15:00<br>5 weai_time_6_buisness___5 15:00-15:15<br>6 weai_time_6_buisness___6 15:15-15:30<br>7 weai_time_6_buisness___7 15:30-15:45<br>8 weai_time_6_buisness___8 15:45-16:00
1142	weai_time_6_farm	Farming/livestock/fishing	checkbox<br>1 weai_time_6_farm___1 14:00-14:15<br>2 weai_time_6_farm___2 14:15-14:30<br>3 weai_time_6_farm___3 14:30-14:45<br>4 weai_time_6_farm___4 14:45-15:00<br>5 weai_time_6_farm___5 15:00-15:15<br>6 weai_time_6_farm___6 15:15-15:30<br>7 weai_time_6_farm___7 15:30-15:45<br>8 weai_time_6_farm___8 15:45-16:00
1143	weai_time_6_shop	Shopping/getting service (incl health services)	checkbox<br>1 weai_time_6_shop___1 14:00-14:15<br>2 weai_time_6_shop___2 14:15-14:30<br>3 weai_time_6_shop___3 14:30-14:45<br>4 weai_time_6_shop___4 14:45-15:00<br>5 weai_time_6_shop___5 15:00-15:15<br>6 weai_time_6_shop___6 15:15-15:30<br>7 weai_time_6_shop___7 15:30-15:45<br>8 weai_time_6_shop___8 15:45-16:00
1144	weai_time_6_weave	Weaving, sewing, textile care	checkbox<br>1 weai_time_6_weave___1 14:00-14:15<br>2 weai_time_6_weave___2 14:15-14:30<br>3 weai_time_6_weave___3 14:30-14:45<br>4 weai_time_6_weave___4 14:45-15:00<br>5 weai_time_6_weave___5 15:00-15:15<br>6 weai_time_6_weave___6 15:15-15:30<br>7 weai_time_6_weave___7 15:30-15:45<br>8 weai_time_6_weave___8 15:45-16:00
1145	weai_time_6_cook	Cooking	checkbox<br>1 weai_time_6_cook___1 14:00-14:15<br>2 weai_time_6_cook___2 14:15-14:30<br>3 weai_time_6_cook___3 14:30-14:45<br>4 weai_time_6_cook___4 14:45-15:00<br>5 weai_time_6_cook___5 15:00-15:15<br>6 weai_time_6_cook___6 15:15-15:30<br>7 weai_time_6_cook___7 15:30-15:45<br>8 weai_time_6_cook___8 15:45-16:00

Havelaar AH, *et al. BMJ Open* 2022; 12:e061311. doi: 10.1136/bmjopen-2022-061311

1146	weai_time_6_domestic	Domestic work (incl fetching wood and water)	checkbox<br>1 weai_time_6_domestic___1 14:00-14:15<br>2 weai_time_6_domestic___2 14:15-14:30<br>3 weai_time_6_domestic___3 14:30-14:45<br>4 weai_time_6_domestic___4 14:45-15:00<br>5 weai_time_6_domestic___5 15:00-15:15<br>6 weai_time_6_domestic___6 15:15-15:30<br>7 weai_time_6_domestic___7 15:30-15:45<br>8 weai_time_6_domestic___8 15:45-16:00
1147	weai_time_6_care	Care for children/adults/elderly	checkbox<br>1 weai_time_6_care___1 14:00-14:15<br>2 weai_time_6_care___2 14:15-14:30<br>3 weai_time_6_care___3 14:30-14:45<br>4 weai_time_6_care___4 14:45-15:00<br>5 weai_time_6_care___5 15:00-15:15<br>6 weai_time_6_care___6 15:15-15:30<br>7 weai_time_6_care___7 15:30-15:45<br>8 weai_time_6_care___8 15:45-16:00
1148	weai_time_6_travel	Travelling and communiting	checkbox<br>1 weai_time_6_travel___1 14:00-14:15<br>2 weai_time_6_travel___2 14:15-14:30<br>3 weai_time_6_travel___3 14:30-14:45<br>4 weai_time_6_travel___4 14:45-15:00<br>5 weai_time_6_travel___5 15:00-15:15<br>6 weai_time_6_travel___6 15:15-15:30<br>7 weai_time_6_travel___7 15:30-15:45<br>8 weai_time_6_travel___8 15:45-16:00
1149	weai_time_6_tv	Watching TV/listening to radio/reading	checkbox<br>1 weai_time_6_tv___1 14:00-14:15<br>2 weai_time_6_tv___2 14:15-14:30<br>3 weai_time_6_tv___3 14:30-14:45<br>4 weai_time_6_tv___4 14:45-15:00<br>5 weai_time_6_tv___5 15:00-15:15<br>6 weai_time_6_tv___6 15:15-15:30<br>7 weai_time_6_tv___7 15:30-15:45<br>8 weai_time_6_tv___8 15:45-16:00
1150	weai_time_6_exercising	Exercising	checkbox<br>1 weai_time_6_exercising___1 14:00-14:15<br>2 weai_time_6_exercising___2 14:15-14:30<br>3 weai_time_6_exercising___3 14:30-14:45<br>4 weai_time_6_exercising___4 14:45-15:00<br>5 weai_time_6_exercising___5 15:00-15:15<br>6 weai_time_6_exercising___6 15:15-15:30<br>7 weai_time_6_exercising___7 15:30-15:45<br>8 weai_time_6_exercising___8 15:45-16:00

Havelaar AH, *et al. BMJ Open* 2022; 12:e061311. doi: 10.1136/bmjopen-2022-061311

1151	weai_time_6_social	Social activities and hobbies	checkbox<br>1 weai_time_6_social___1 14:00-14:15<br>2 weai_time_6_social___2 14:15-14:30<br>3 weai_time_6_social___3 14:30-14:45<br>4 weai_time_6_social___4 14:45-15:00<br>5 weai_time_6_social___5 15:00-15:15<br>6 weai_time_6_social___6 15:15-15:30<br>7 weai_time_6_social___7 15:30-15:45<br>8 weai_time_6_social___8 15:45-16:00
1152	weai_time_6_religious	Religious activities	checkbox<br>1 weai_time_6_religious___1 14:00-14:15<br>2 weai_time_6_religious___2 14:15-14:30<br>3 weai_time_6_religious___3 14:30-14:45<br>4 weai_time_6_religious___4 14:45-15:00<br>5 weai_time_6_religious___5 15:00-15:15<br>6 weai_time_6_religious___6 15:15-15:30<br>7 weai_time_6_religious___7 15:30-15:45<br>8 weai_time_6_religious___8 15:45-16:00
1153	weai_time_6_other	Other (specify below)	checkbox<br>1 weai_time_6_other___1 14:00-14:15<br>2 weai_time_6_other___2 14:15-14:30<br>3 weai_time_6_other___3 14:30-14:45<br>4 weai_time_6_other___4 14:45-15:00<br>5 weai_time_6_other___5 15:00-15:15<br>6 weai_time_6_other___6 15:15-15:30<br>7 weai_time_6_other___7 15:30-15:45<br>8 weai_time_6_other___8 15:45-16:00
1154	weai_time_6_other_specify<br>Show the field ONLY if: [weai_time_6_other(1)] or [weai_time_6_other(2)] or [weai_time_6_other(3)] or [weai_time_6_other(4)] or [weai_time_6_other(5)] or [weai_time_6_other(6)] or [weai_time_6_other(7)] or [weai_time_6_other(8)]	Specify Other	text<br>Custom alignment: LV
1155	weai_time_7_sleep	Section Header: *Night/Early morning*<br>Sleeping and resting	checkbox<br>1 weai_time_7_sleep___1 16:00-16:15<br>2 weai_time_7_sleep___2 16:15-16:30<br>3 weai_time_7_sleep___3 16:30-16:45<br>4 weai_time_7_sleep___4 16:45-17:00<br>5 weai_time_7_sleep___5 17:00-17:15<br>6 weai_time_7_sleep___6 17:15-17:30<br>7 weai_time_7_sleep___7 17:30-17:45<br>8 weai_time_7_sleep___8 17:45-18:00

Havelaar AH, *et al. BMJ Open* 2022; 12:e061311. doi: 10.1136/bmjopen-2022-061311

1156	weai_time_7_eat	Eating and drinking	checkbox<br>1 weai_time_7_eat___1 16:00-16:15<br>2 weai_time_7_eat___2 16:15-16:30<br>3 weai_time_7_eat___3 16:30-16:45<br>4 weai_time_7_eat___4 16:45-17:00<br>5 weai_time_7_eat___5 17:00-17:15<br>6 weai_time_7_eat___6 17:15-17:30<br>7 weai_time_7_eat___7 17:30-17:45<br>8 weai_time_7_eat___8 17:45-18:00
1157	weai_time_7_personal	Personal Care	checkbox<br>1 weai_time_7_personal___1 16:00-16:15<br>2 weai_time_7_personal___2 16:15-16:30<br>3 weai_time_7_personal___3 16:30-16:45<br>4 weai_time_7_personal___4 16:45-17:00<br>5 weai_time_7_personal___5 17:00-17:15<br>6 weai_time_7_personal___6 17:15-17:30<br>7 weai_time_7_personal___7 17:30-17:45<br>8 weai_time_7_personal___8 17:45-18:00
1158	weai_time_7_school	School (also homework)	checkbox<br>1 weai_time_7_school___1 16:00-16:15<br>2 weai_time_7_school___2 16:15-16:30<br>3 weai_time_7_school___3 16:30-16:45<br>4 weai_time_7_school___4 16:45-17:00<br>5 weai_time_7_school___5 17:00-17:15<br>6 weai_time_7_school___6 17:15-17:30<br>7 weai_time_7_school___7 17:30-17:45<br>8 weai_time_7_school___8 17:45-18:00
1159	weai_time_7_work	Work as employed	checkbox<br>1 weai_time_7_work___1 16:00-16:15<br>2 weai_time_7_work___2 16:15-16:30<br>3 weai_time_7_work___3 16:30-16:45<br>4 weai_time_7_work___4 16:45-17:00<br>5 weai_time_7_work___5 17:00-17:15<br>6 weai_time_7_work___6 17:15-17:30<br>7 weai_time_7_work___7 17:30-17:45<br>8 weai_time_7_work___8 17:45-18:00
1160	weai_time_7_buisness	Own business work	checkbox<br>1 weai_time_7_buisness___1 16:00-16:15<br>2 weai_time_7_buisness___2 16:15-16:30<br>3 weai_time_7_buisness___3 16:30-16:45<br>4 weai_time_7_buisness___4 16:45-17:00<br>5 weai_time_7_buisness___5 17:00-17:15<br>6 weai_time_7_buisness___6 17:15-17:30<br>7 weai_time_7_buisness___7 17:30-17:45<br>8 weai_time_7_buisness___8 17:45-18:00

Havelaar AH, *et al. BMJ Open* 2022; 12:e061311. doi: 10.1136/bmjopen-2022-061311

Supplemental material
BMJ Publishing Group Limited (BMJ) disclaims all liability and responsibility arising from any reliance placed on this supplemental material which has been supplied by the author(s)
BMJ Open

1161	weai_time_7_farm	Farming/livestock/fishing	checkbox<br>1 weai_time_7_farm___1 16:00-16:15<br>2 weai_time_7_farm___2 16:15-16:30<br>3 weai_time_7_farm___3 16:30-16:45<br>4 weai_time_7_farm___4 16:45-17:00<br>5 weai_time_7_farm___5 17:00-17:15<br>6 weai_time_7_farm___6 17:15-17:30<br>7 weai_time_7_farm___7 17:30-17:45<br>8 weai_time_7_farm___8 17:45-18:00
1162	weai_time_7_shop	Shopping/getting service (incl health services)	checkbox<br>1 weai_time_7_shop___1 16:00-16:15<br>2 weai_time_7_shop___2 16:15-16:30<br>3 weai_time_7_shop___3 16:30-16:45<br>4 weai_time_7_shop___4 16:45-17:00<br>5 weai_time_7_shop___5 17:00-17:15<br>6 weai_time_7_shop___6 17:15-17:30<br>7 weai_time_7_shop___7 17:30-17:45<br>8 weai_time_7_shop___8 17:45-18:00
1163	weai_time_7_weave	Weaving, sewing, textile care	checkbox<br>1 weai_time_7_weave___1 16:00-16:15<br>2 weai_time_7_weave___2 16:15-16:30<br>3 weai_time_7_weave___3 16:30-16:45<br>4 weai_time_7_weave___4 16:45-17:00<br>5 weai_time_7_weave___5 17:00-17:15<br>6 weai_time_7_weave___6 17:15-17:30<br>7 weai_time_7_weave___7 17:30-17:45<br>8 weai_time_7_weave___8 17:45-18:00
1164	weai_time_7_cook	Cooking	checkbox<br>1 weai_time_7_cook___1 16:00-16:15<br>2 weai_time_7_cook___2 16:15-16:30<br>3 weai_time_7_cook___3 16:30-16:45<br>4 weai_time_7_cook___4 16:45-17:00<br>5 weai_time_7_cook___5 17:00-17:15<br>6 weai_time_7_cook___6 17:15-17:30<br>7 weai_time_7_cook___7 17:30-17:45<br>8 weai_time_7_cook___8 17:45-18:00
1165	weai_time_7_domestic	Domestic work (incl fetching wood and water)	checkbox<br>1 weai_time_7_domestic___1 16:00-16:15<br>2 weai_time_7_domestic___2 16:15-16:30<br>3 weai_time_7_domestic___3 16:30-16:45<br>4 weai_time_7_domestic___4 16:45-17:00<br>5 weai_time_7_domestic___5 17:00-17:15<br>6 weai_time_7_domestic___6 17:15-17:30<br>7 weai_time_7_domestic___7 17:30-17:45<br>8 weai_time_7_domestic___8 17:45-18:00

Havelaar AH, *et al. BMJ Open* 2022; 12:e061311. doi: 10.1136/bmjopen-2022-061311

1166	weai_time_7_care	Care for children/adults/elderly	checkbox<br>1 weai_time_7_care___1 16:00-16:15<br>2 weai_time_7_care___2 16:15-16:30<br>3 weai_time_7_care___3 16:30-16:45<br>4 weai_time_7_care___4 16:45-17:00<br>5 weai_time_7_care___5 17:00-17:15<br>6 weai_time_7_care___6 17:15-17:30<br>7 weai_time_7_care___7 17:30-17:45<br>8 weai_time_7_care___8 17:45-18:00
1167	weai_time_7_travel	Travelling and communiting	checkbox<br>1 weai_time_7_travel___1 16:00-16:15<br>2 weai_time_7_travel___2 16:15-16:30<br>3 weai_time_7_travel___3 16:30-16:45<br>4 weai_time_7_travel___4 16:45-17:00<br>5 weai_time_7_travel___5 17:00-17:15<br>6 weai_time_7_travel___6 17:15-17:30<br>7 weai_time_7_travel___7 17:30-17:45<br>8 weai_time_7_travel___8 17:45-18:00
1168	weai_time_7_tv	Watching TV/listening to radio/reading	checkbox<br>1 weai_time_7_tv___1 16:00-16:15<br>2 weai_time_7_tv___2 16:15-16:30<br>3 weai_time_7_tv___3 16:30-16:45<br>4 weai_time_7_tv___4 16:45-17:00<br>5 weai_time_7_tv___5 17:00-17:15<br>6 weai_time_7_tv___6 17:15-17:30<br>7 weai_time_7_tv___7 17:30-17:45<br>8 weai_time_7_tv___8 17:45-18:00
1169	weai_time_7_exercising	Exercising	checkbox<br>1 weai_time_7_exercising___1 16:00-16:15<br>2 weai_time_7_exercising___2 16:15-16:30<br>3 weai_time_7_exercising___3 16:30-16:45<br>4 weai_time_7_exercising___4 16:45-17:00<br>5 weai_time_7_exercising___5 17:00-17:15<br>6 weai_time_7_exercising___6 17:15-17:30<br>7 weai_time_7_exercising___7 17:30-17:45<br>8 weai_time_7_exercising___8 17:45-18:00
1170	weai_time_7_social	Social activities and hobbies	checkbox<br>1 weai_time_7_social___1 16:00-16:15<br>2 weai_time_7_social___2 16:15-16:30<br>3 weai_time_7_social___3 16:30-16:45<br>4 weai_time_7_social___4 16:45-17:00<br>5 weai_time_7_social___5 17:00-17:15<br>6 weai_time_7_social___6 17:15-17:30<br>7 weai_time_7_social___7 17:30-17:45<br>8 weai_time_7_social___8 17:45-18:00

Havelaar AH, *et al. BMJ Open* 2022; 12:e061311. doi: 10.1136/bmjopen-2022-061311

1171	weai_time_7_religious	Religious activities	checkbox<br>1 weai_time_7_religious___1 16:00-16:15<br>2 weai_time_7_religious___2 16:15-16:30<br>3 weai_time_7_religious___3 16:30-16:45<br>4 weai_time_7_religious___4 16:45-17:00<br>5 weai_time_7_religious___5 17:00-17:15<br>6 weai_time_7_religious___6 17:15-17:30<br>7 weai_time_7_religious___7 17:30-17:45<br>8 weai_time_7_religious___8 17:45-18:00
1172	weai_time_7_other	Other (specify below)	checkbox<br>1 weai_time_7_other___1 16:00-16:15<br>2 weai_time_7_other___2 16:15-16:30<br>3 weai_time_7_other___3 16:30-16:45<br>4 weai_time_7_other___4 16:45-17:00<br>5 weai_time_7_other___5 17:00-17:15<br>6 weai_time_7_other___6 17:15-17:30<br>7 weai_time_7_other___7 17:30-17:45<br>8 weai_time_7_other___8 17:45-18:00
1173	weai_time_7_other_specify<br>Show the field ONLY if:<br>[weai_time_7_other(1)] or [weai_time_7_other(2)] or [weai_time_7_other(3)] or [weai_time_7_other(4)] or [weai_time_7_other(5)] or [weai_time_7_other(6)] or [weai_time_7_other(7)] or [weai_time_7_other(8)]	Specify Other	text<br>Custom alignment: LV
1174	weai_time_8_sleep	Section Header: *Night/Early morning*<br>Sleeping and resting	checkbox<br>1 weai_time_8_sleep___1 18:00-18:15<br>2 weai_time_8_sleep___2 18:15-18:30<br>3 weai_time_8_sleep___3 18:30-18:45<br>4 weai_time_8_sleep___4 18:45-19:00<br>5 weai_time_8_sleep___5 19:00-19:15<br>6 weai_time_8_sleep___6 19:15-19:30<br>7 weai_time_8_sleep___7 19:30-19:45<br>8 weai_time_8_sleep___8 19:45-20:00
1175	weai_time_8_eat	Eating and drinking	checkbox<br>1 weai_time_8_eat___1 18:00-18:15<br>2 weai_time_8_eat___2 18:15-18:30<br>3 weai_time_8_eat___3 18:30-18:45<br>4 weai_time_8_eat___4 18:45-19:00<br>5 weai_time_8_eat___5 19:00-19:15<br>6 weai_time_8_eat___6 19:15-19:30<br>7 weai_time_8_eat___7 19:30-19:45<br>8 weai_time_8_eat___8 19:45-20:00

Havelaar AH, *et al. BMJ Open* 2022; 12:e061311. doi: 10.1136/bmjopen-2022-061311

1176	weai_time_8_personal	Personal Care	checkbox<br>1 weai_time_8_personal___1 18:00-18:15<br>2 weai_time_8_personal___2 18:15-18:30<br>3 weai_time_8_personal___3 18:30-18:45<br>4 weai_time_8_personal___4 18:45-19:00<br>5 weai_time_8_personal___5 19:00-19:15<br>6 weai_time_8_personal___6 19:15-19:30<br>7 weai_time_8_personal___7 19:30-19:45<br>8 weai_time_8_personal___8 19:45-20:00
1177	weai_time_8_school	School (also homework)	checkbox<br>1 weai_time_8_school___1 18:00-18:15<br>2 weai_time_8_school___2 18:15-18:30<br>3 weai_time_8_school___3 18:30-18:45<br>4 weai_time_8_school___4 18:45-19:00<br>5 weai_time_8_school___5 19:00-19:15<br>6 weai_time_8_school___6 19:15-19:30<br>7 weai_time_8_school___7 19:30-19:45<br>8 weai_time_8_school___8 19:45-20:00
1178	weai_time_8_work	Work as employed	checkbox<br>1 weai_time_8_work___1 18:00-18:15<br>2 weai_time_8_work___2 18:15-18:30<br>3 weai_time_8_work___3 18:30-18:45<br>4 weai_time_8_work___4 18:45-19:00<br>5 weai_time_8_work___5 19:00-19:15<br>6 weai_time_8_work___6 19:15-19:30<br>7 weai_time_8_work___7 19:30-19:45<br>8 weai_time_8_work___8 19:45-20:00
1179	weai_time_8_buisness	Own business work	checkbox<br>1 weai_time_8_buisness___1 18:00-18:15<br>2 weai_time_8_buisness___2 18:15-18:30<br>3 weai_time_8_buisness___3 18:30-18:45<br>4 weai_time_8_buisness___4 18:45-19:00<br>5 weai_time_8_buisness___5 19:00-19:15<br>6 weai_time_8_buisness___6 19:15-19:30<br>7 weai_time_8_buisness___7 19:30-19:45<br>8 weai_time_8_buisness___8 19:45-20:00
1180	weai_time_8_farm	Farming/livestock/fishing	checkbox<br>1 weai_time_8_farm___1 18:00-18:15<br>2 weai_time_8_farm___2 18:15-18:30<br>3 weai_time_8_farm___3 18:30-18:45<br>4 weai_time_8_farm___4 18:45-19:00<br>5 weai_time_8_farm___5 19:00-19:15<br>6 weai_time_8_farm___6 19:15-19:30<br>7 weai_time_8_farm___7 19:30-19:45<br>8 weai_time_8_farm___8 19:45-20:00

1181	weai_time_8_shop	Shopping/getting service (incl health services)	checkbox<br>1 weai_time_8_shop___1 18:00-18:15<br>2 weai_time_8_shop___2 18:15-18:30<br>3 weai_time_8_shop___3 18:30-18:45<br>4 weai_time_8_shop___4 18:45-19:00<br>5 weai_time_8_shop___5 19:00-19:15<br>6 weai_time_8_shop___6 19:15-19:30<br>7 weai_time_8_shop___7 19:30-19:45<br>8 weai_time_8_shop___8 19:45-20:00
1182	weai_time_8_weave	Weaving, sewing, textile care	checkbox<br>1 weai_time_8_weave___1 18:00-18:15<br>2 weai_time_8_weave___2 18:15-18:30<br>3 weai_time_8_weave___3 18:30-18:45<br>4 weai_time_8_weave___4 18:45-19:00<br>5 weai_time_8_weave___5 19:00-19:15<br>6 weai_time_8_weave___6 19:15-19:30<br>7 weai_time_8_weave___7 19:30-19:45<br>8 weai_time_8_weave___8 19:45-20:00
1183	weai_time_8_cook	Cooking	checkbox<br>1 weai_time_8_cook___1 18:00-18:15<br>2 weai_time_8_cook___2 18:15-18:30<br>3 weai_time_8_cook___3 18:30-18:45<br>4 weai_time_8_cook___4 18:45-19:00<br>5 weai_time_8_cook___5 19:00-19:15<br>6 weai_time_8_cook___6 19:15-19:30<br>7 weai_time_8_cook___7 19:30-19:45<br>8 weai_time_8_cook___8 19:45-20:00
1184	weai_time_8_domestic	Domestic work (incl fetching wood and water)	checkbox<br>1 weai_time_8_domestic___1 18:00-18:15<br>2 weai_time_8_domestic___2 18:15-18:30<br>3 weai_time_8_domestic___3 18:30-18:45<br>4 weai_time_8_domestic___4 18:45-19:00<br>5 weai_time_8_domestic___5 19:00-19:15<br>6 weai_time_8_domestic___6 19:15-19:30<br>7 weai_time_8_domestic___7 19:30-19:45<br>8 weai_time_8_domestic___8 19:45-20:00
1185	weai_time_8_care	Care for children/adults/elderly	checkbox<br>1 weai_time_8_care___1 18:00-18:15<br>2 weai_time_8_care___2 18:15-18:30<br>3 weai_time_8_care___3 18:30-18:45<br>4 weai_time_8_care___4 18:45-19:00<br>5 weai_time_8_care___5 19:00-19:15<br>6 weai_time_8_care___6 19:15-19:30<br>7 weai_time_8_care___7 19:30-19:45<br>8 weai_time_8_care___8 19:45-20:00

Havelaar AH, *et al. BMJ Open* 2022; 12:e061311. doi: 10.1136/bmjopen-2022-061311

1186	weai_time_8_travel	Travelling and communiting	checkbox<br>1 weai_time_8_travel___1 18:00-18:15<br>2 weai_time_8_travel___2 18:15-18:30<br>3 weai_time_8_travel___3 18:30-18:45<br>4 weai_time_8_travel___4 18:45-19:00<br>5 weai_time_8_travel___5 19:00-19:15<br>6 weai_time_8_travel___6 19:15-19:30<br>7 weai_time_8_travel___7 19:30-19:45<br>8 weai_time_8_travel___8 19:45-20:00
1187	weai_time_8_tv	Watching TV/listening to radio/reading	checkbox<br>1 weai_time_8_tv___1 18:00-18:15<br>2 weai_time_8_tv___2 18:15-18:30<br>3 weai_time_8_tv___3 18:30-18:45<br>4 weai_time_8_tv___4 18:45-19:00<br>5 weai_time_8_tv___5 19:00-19:15<br>6 weai_time_8_tv___6 19:15-19:30<br>7 weai_time_8_tv___7 19:30-19:45<br>8 weai_time_8_tv___8 19:45-20:00
1188	weai_time_8_exercising	Exercising	checkbox<br>1 weai_time_8_exercising___1 18:00-18:15<br>2 weai_time_8_exercising___2 18:15-18:30<br>3 weai_time_8_exercising___3 18:30-18:45<br>4 weai_time_8_exercising___4 18:45-19:00<br>5 weai_time_8_exercising___5 19:00-19:15<br>6 weai_time_8_exercising___6 19:15-19:30<br>7 weai_time_8_exercising___7 19:30-19:45<br>8 weai_time_8_exercising___8 19:45-20:00
1189	weai_time_8_social	Social activities and hobbies	checkbox<br>1 weai_time_8_social___1 18:00-18:15<br>2 weai_time_8_social___2 18:15-18:30<br>3 weai_time_8_social___3 18:30-18:45<br>4 weai_time_8_social___4 18:45-19:00<br>5 weai_time_8_social___5 19:00-19:15<br>6 weai_time_8_social___6 19:15-19:30<br>7 weai_time_8_social___7 19:30-19:45<br>8 weai_time_8_social___8 19:45-20:00
1190	weai_time_8_religious	Religious activities	checkbox<br>1 weai_time_8_religious___1 18:00-18:15<br>2 weai_time_8_religious___2 18:15-18:30<br>3 weai_time_8_religious___3 18:30-18:45<br>4 weai_time_8_religious___4 18:45-19:00<br>5 weai_time_8_religious___5 19:00-19:15<br>6 weai_time_8_religious___6 19:15-19:30<br>7 weai_time_8_religious___7 19:30-19:45<br>8 weai_time_8_religious___8 19:45-20:00

1191	weai_time_8_other	Other (specify below)	checkbox<br>1 weai_time_8_other___1 18:00-18:15<br>2 weai_time_8_other___2 18:15-18:30<br>3 weai_time_8_other___3 18:30-18:45<br>4 weai_time_8_other___4 18:45-19:00<br>5 weai_time_8_other___5 19:00-19:15<br>6 weai_time_8_other___6 19:15-19:30<br>7 weai_time_8_other___7 19:30-19:45<br>8 weai_time_8_other___8 19:45-20:00
1192	weai_time_8_other_specify<br>Show the field ONLY if:<br>[weai_time_8_other(1)] or [weai_time_8_other(2)] or [weai_time_8_other(3)] or [weai_time_8_other(4)] or [weai_time_8_other(5)] or [weai_time_8_other(6)] or [weai_time_8_other(7)] or [weai_time_8_other(8)]	Specify Other	text<br>Custom alignment: LV
1193	weai_time_9_sleep	Section Header: *Night/Early morning*<br>Sleeping and resting	checkbox<br>1 weai_time_9_sleep___1 20:00-20:15<br>2 weai_time_9_sleep___2 20:15-20:30<br>3 weai_time_9_sleep___3 20:30-20:45<br>4 weai_time_9_sleep___4 20:45-21:00<br>5 weai_time_9_sleep___5 21:00-21:15<br>6 weai_time_9_sleep___6 21:15-21:30<br>7 weai_time_9_sleep___7 21:30-21:45<br>8 weai_time_9_sleep___8 21:45-22:00
1194	weai_time_9_eat	Eating and drinking	checkbox<br>1 weai_time_9_eat___1 20:00-20:15<br>2 weai_time_9_eat___2 20:15-20:30<br>3 weai_time_9_eat___3 20:30-20:45<br>4 weai_time_9_eat___4 20:45-21:00<br>5 weai_time_9_eat___5 21:00-21:15<br>6 weai_time_9_eat___6 21:15-21:30<br>7 weai_time_9_eat___7 21:30-21:45<br>8 weai_time_9_eat___8 21:45-22:00
1195	weai_time_9_personal	Personal Care	checkbox<br>1 weai_time_9_personal___1 20:00-20:15<br>2 weai_time_9_personal___2 20:15-20:30<br>3 weai_time_9_personal___3 20:30-20:45<br>4 weai_time_9_personal___4 20:45-21:00<br>5 weai_time_9_personal___5 21:00-21:15<br>6 weai_time_9_personal___6 21:15-21:30<br>7 weai_time_9_personal___7 21:30-21:45<br>8 weai_time_9_personal___8 21:45-22:00

Havelaar AH, *et al. BMJ Open* 2022; 12:e061311. doi: 10.1136/bmjopen-2022-061311

1196	weai_time_9_school	School (also homework)	checkbox<br>1 | weai_time_9_school___1 | 20:00-20:15<br>2 | weai_time_9_school___2 | 20:15-20:30<br>3 | weai_time_9_school___3 | 20:30-20:45<br>4 | weai_time_9_school___4 | 20:45-21:00<br>5 | weai_time_9_school___5 | 21:00-21:15<br>6 | weai_time_9_school___6 | 21:15-21:30<br>7 | weai_time_9_school___7 | 21:30-21:45<br>8 | weai_time_9_school___8 | 21:45-22:00
1197	weai_time_9_work	Work as employed	checkbox<br>1 | weai_time_9_work___1 | 20:00-20:15<br>2 | weai_time_9_work___2 | 20:15-20:30<br>3 | weai_time_9_work___3 | 20:30-20:45<br>4 | weai_time_9_work___4 | 20:45-21:00<br>5 | weai_time_9_work___5 | 21:00-21:15<br>6 | weai_time_9_work___6 | 21:15-21:30<br>7 | weai_time_9_work___7 | 21:30-21:45<br>8 | weai_time_9_work___8 | 21:45-22:00
1198	weai_time_9_buisness	Own business work	checkbox<br>1 | weai_time_9_buisness___1 | 20:00-20:15<br>2 | weai_time_9_buisness___2 | 20:15-20:30<br>3 | weai_time_9_buisness___3 | 20:30-20:45<br>4 | weai_time_9_buisness___4 | 20:45-21:00<br>5 | weai_time_9_buisness___5 | 21:00-21:15<br>6 | weai_time_9_buisness___6 | 21:15-21:30<br>7 | weai_time_9_buisness___7 | 21:30-21:45<br>8 | weai_time_9_buisness___8 | 21:45-22:00
1199	weai_time_9_farm	Farming/livestock/fishing	checkbox<br>1 | weai_time_9_farm___1 | 20:00-20:15<br>2 | weai_time_9_farm___2 | 20:15-20:30<br>3 | weai_time_9_farm___3 | 20:30-20:45<br>4 | weai_time_9_farm___4 | 20:45-21:00<br>5 | weai_time_9_farm___5 | 21:00-21:15<br>6 | weai_time_9_farm___6 | 21:15-21:30<br>7 | weai_time_9_farm___7 | 21:30-21:45<br>8 | weai_time_9_farm___8 | 21:45-22:00
1200	weai_time_9_shop	Shopping/getting service (incl health services)	checkbox<br>1 | weai_time_9_shop___1 | 20:00-20:15<br>2 | weai_time_9_shop___2 | 20:15-20:30<br>3 | weai_time_9_shop___3 | 20:30-20:45<br>4 | weai_time_9_shop___4 | 20:45-21:00<br>5 | weai_time_9_shop___5 | 21:00-21:15<br>6 | weai_time_9_shop___6 | 21:15-21:30<br>7 | weai_time_9_shop___7 | 21:30-21:45<br>8 | weai_time_9_shop___8 | 21:45-22:00

1201	weai_time_9_weave	Weaving, sewing, textile care	checkbox<br>1 | weai_time_9_weave___1 | 20:00-20:15<br>2 | weai_time_9_weave___2 | 20:15-20:30<br>3 | weai_time_9_weave___3 | 20:30-20:45<br>4 | weai_time_9_weave___4 | 20:45-21:00<br>5 | weai_time_9_weave___5 | 21:00-21:15<br>6 | weai_time_9_weave___6 | 21:15-21:30<br>7 | weai_time_9_weave___7 | 21:30-21:45<br>8 | weai_time_9_weave___8 | 21:45-22:00
1202	weai_time_9_cook	Cooking	checkbox<br>1 | weai_time_9_cook___1 | 20:00-20:15<br>2 | weai_time_9_cook___2 | 20:15-20:30<br>3 | weai_time_9_cook___3 | 20:30-20:45<br>4 | weai_time_9_cook___4 | 20:45-21:00<br>5 | weai_time_9_cook___5 | 21:00-21:15<br>6 | weai_time_9_cook___6 | 21:15-21:30<br>7 | weai_time_9_cook___7 | 21:30-21:45<br>8 | weai_time_9_cook___8 | 21:45-22:00
1203	weai_time_9_domestic	Domestic work (incl fetching wood and water)	checkbox<br>1 | weai_time_9_domestic___1 | 20:00-20:15<br>2 | weai_time_9_domestic___2 | 20:15-20:30<br>3 | weai_time_9_domestic___3 | 20:30-20:45<br>4 | weai_time_9_domestic___4 | 20:45-21:00<br>5 | weai_time_9_domestic___5 | 21:00-21:15<br>6 | weai_time_9_domestic___6 | 21:15-21:30<br>7 | weai_time_9_domestic___7 | 21:30-21:45<br>8 | weai_time_9_domestic___8 | 21:45-22:00
1204	weai_time_9_care	Care for children/adults/elderly	checkbox<br>1 | weai_time_9_care___1 | 20:00-20:15<br>2 | weai_time_9_care___2 | 20:15-20:30<br>3 | weai_time_9_care___3 | 20:30-20:45<br>4 | weai_time_9_care___4 | 20:45-21:00<br>5 | weai_time_9_care___5 | 21:00-21:15<br>6 | weai_time_9_care___6 | 21:15-21:30<br>7 | weai_time_9_care___7 | 21:30-21:45<br>8 | weai_time_9_care___8 | 21:45-22:00
1205	weai_time_9_travel	Travelling and communiting	checkbox<br>1 | weai_time_9_travel___1 | 20:00-20:15<br>2 | weai_time_9_travel___2 | 20:15-20:30<br>3 | weai_time_9_travel___3 | 20:30-20:45<br>4 | weai_time_9_travel___4 | 20:45-21:00<br>5 | weai_time_9_travel___5 | 21:00-21:15<br>6 | weai_time_9_travel___6 | 21:15-21:30<br>7 | weai_time_9_travel___7 | 21:30-21:45<br>8 | weai_time_9_travel___8 | 21:45-22:00

1206	weai_time_9_tv	Watching TV/listening to radio/reading	checkbox<br>1 weai_time_9_tv___1 20:00-20:15<br>2 weai_time_9_tv___2 20:15-20:30<br>3 weai_time_9_tv___3 20:30-20:45<br>4 weai_time_9_tv___4 20:45-21:00<br>5 weai_time_9_tv___5 21:00-21:15<br>6 weai_time_9_tv___6 21:15-21:30<br>7 weai_time_9_tv___7 21:30-21:45<br>8 weai_time_9_tv___8 21:45-22:00
1207	weai_time_9_exercising	Exercising	checkbox<br>1 weai_time_9_exercising___1 20:00-20:15<br>2 weai_time_9_exercising___2 20:15-20:30<br>3 weai_time_9_exercising___3 20:30-20:45<br>4 weai_time_9_exercising___4 20:45-21:00<br>5 weai_time_9_exercising___5 21:00-21:15<br>6 weai_time_9_exercising___6 21:15-21:30<br>7 weai_time_9_exercising___7 21:30-21:45<br>8 weai_time_9_exercising___8 21:45-22:00
1208	weai_time_9_social	Social activities and hobbies	checkbox<br>1 weai_time_9_social___1 20:00-20:15<br>2 weai_time_9_social___2 20:15-20:30<br>3 weai_time_9_social___3 20:30-20:45<br>4 weai_time_9_social___4 20:45-21:00<br>5 weai_time_9_social___5 21:00-21:15<br>6 weai_time_9_social___6 21:15-21:30<br>7 weai_time_9_social___7 21:30-21:45<br>8 weai_time_9_social___8 21:45-22:00
1209	weai_time_9_religious	Religious activities	checkbox<br>1 weai_time_9_religious___1 20:00-20:15<br>2 weai_time_9_religious___2 20:15-20:30<br>3 weai_time_9_religious___3 20:30-20:45<br>4 weai_time_9_religious___4 20:45-21:00<br>5 weai_time_9_religious___5 21:00-21:15<br>6 weai_time_9_religious___6 21:15-21:30<br>7 weai_time_9_religious___7 21:30-21:45<br>8 weai_time_9_religious___8 21:45-22:00
1210	weai_time_9_other	Other (specify below)	checkbox<br>1 weai_time_9_other___1 20:00-20:15<br>2 weai_time_9_other___2 20:15-20:30<br>3 weai_time_9_other___3 20:30-20:45<br>4 weai_time_9_other___4 20:45-21:00<br>5 weai_time_9_other___5 21:00-21:15<br>6 weai_time_9_other___6 21:15-21:30<br>7 weai_time_9_other___7 21:30-21:45<br>8 weai_time_9_other___8 21:45-22:00

Havelaar AH, *et al. BMJ Open* 2022; 12:e061311. doi: 10.1136/bmjopen-2022-061311

1211	weai_time_9_other_specify<br><br>Show the field ONLY if: [weai_time_9_other(1)] or [weai_time_9_other(2)] or [weai_time_9_other(3)] or [weai_time_9_other(4)] or [weai_time_9_other(5)] or [weai_time_9_other(6)] or [weai_time_9_other(7)] or [weai_time_9_other(8)]	Specify Other	text<br>Custom alignment: LV
1212	weai_time_10_sleep	Section Header: *Night/Early morning*<br>Sleeping and resting	checkbox<br>1 weai_time_10_sleep___1 22:00-22:15<br>2 weai_time_10_sleep___2 22:15-22:30<br>3 weai_time_10_sleep___3 22:30-22:45<br>4 weai_time_10_sleep___4 22:45-23:00<br>5 weai_time_10_sleep___5 23:00-23:15<br>6 weai_time_10_sleep___6 23:15-23:30<br>7 weai_time_10_sleep___7 23:30-23:45<br>8 weai_time_10_sleep___8 23:45-00:00
1213	weai_time_10_eat	Eating and drinking	checkbox<br>1 weai_time_10_eat___1 22:00-22:15<br>2 weai_time_10_eat___2 22:15-22:30<br>3 weai_time_10_eat___3 22:30-22:45<br>4 weai_time_10_eat___4 22:45-23:00<br>5 weai_time_10_eat___5 23:00-23:15<br>6 weai_time_10_eat___6 23:15-23:30<br>7 weai_time_10_eat___7 23:30-23:45<br>8 weai_time_10_eat___8 23:45-00:00
1214	weai_time_10_personal	Personal Care	checkbox<br>1 weai_time_10_personal___1 22:00-22:15<br>2 weai_time_10_personal___2 22:15-22:30<br>3 weai_time_10_personal___3 22:30-22:45<br>4 weai_time_10_personal___4 22:45-23:00<br>5 weai_time_10_personal___5 23:00-23:15<br>6 weai_time_10_personal___6 23:15-23:30<br>7 weai_time_10_personal___7 23:30-23:45<br>8 weai_time_10_personal___8 23:45-00:00
1215	weai_time_10_school	School (also homework)	checkbox<br>1 weai_time_10_school___1 22:00-22:15<br>2 weai_time_10_school___2 22:15-22:30<br>3 weai_time_10_school___3 22:30-22:45<br>4 weai_time_10_school___4 22:45-23:00<br>5 weai_time_10_school___5 23:00-23:15<br>6 weai_time_10_school___6 23:15-23:30<br>7 weai_time_10_school___7 23:30-23:45<br>8 weai_time_10_school___8 23:45-00:00

Havelaar AH, *et al. BMJ Open* 2022; 12:e061311. doi: 10.1136/bmjopen-2022-061311

1216	weai_time_10_work	Work as employed	checkbox<br>1 weai_time_10_work___1 22:00-22:15<br>2 weai_time_10_work___2 22:15-22:30<br>3 weai_time_10_work___3 22:30-22:45<br>4 weai_time_10_work___4 22:45-23:00<br>5 weai_time_10_work___5 23:00-23:15<br>6 weai_time_10_work___6 23:15-23:30<br>7 weai_time_10_work___7 23:30-23:45<br>8 weai_time_10_work___8 23:45-00:00
1217	weai_time_10_buisness	Own business work	checkbox<br>1 weai_time_10_buisness___1 22:00-22:15<br>2 weai_time_10_buisness___2 22:15-22:30<br>3 weai_time_10_buisness___3 22:30-22:45<br>4 weai_time_10_buisness___4 22:45-23:00<br>5 weai_time_10_buisness___5 23:00-23:15<br>6 weai_time_10_buisness___6 23:15-23:30<br>7 weai_time_10_buisness___7 23:30-23:45<br>8 weai_time_10_buisness___8 23:45-00:00
1218	weai_time_10_farm	Farming/livestock/fishing	checkbox<br>1 weai_time_10_farm___1 22:00-22:15<br>2 weai_time_10_farm___2 22:15-22:30<br>3 weai_time_10_farm___3 22:30-22:45<br>4 weai_time_10_farm___4 22:45-23:00<br>5 weai_time_10_farm___5 23:00-23:15<br>6 weai_time_10_farm___6 23:15-23:30<br>7 weai_time_10_farm___7 23:30-23:45<br>8 weai_time_10_farm___8 23:45-00:00
1219	weai_time_10_shop	Shopping/getting service (incl health services)	checkbox<br>1 weai_time_10_shop___1 22:00-22:15<br>2 weai_time_10_shop___2 22:15-22:30<br>3 weai_time_10_shop___3 22:30-22:45<br>4 weai_time_10_shop___4 22:45-23:00<br>5 weai_time_10_shop___5 23:00-23:15<br>6 weai_time_10_shop___6 23:15-23:30<br>7 weai_time_10_shop___7 23:30-23:45<br>8 weai_time_10_shop___8 23:45-00:00
1220	weai_time_10_weave	Weaving, sewing, textile care	checkbox<br>1 weai_time_10_weave___1 22:00-22:15<br>2 weai_time_10_weave___2 22:15-22:30<br>3 weai_time_10_weave___3 22:30-22:45<br>4 weai_time_10_weave___4 22:45-23:00<br>5 weai_time_10_weave___5 23:00-23:15<br>6 weai_time_10_weave___6 23:15-23:30<br>7 weai_time_10_weave___7 23:30-23:45<br>8 weai_time_10_weave___8 23:45-00:00

Havelaar AH, *et al. BMJ Open* 2022; 12:e061311. doi: 10.1136/bmjopen-2022-061311

1221	weai_time_10_cook	Cooking	checkbox<br>1 weai_time_10_cook___1 22:00-22:15<br>2 weai_time_10_cook___2 22:15-22:30<br>3 weai_time_10_cook___3 22:30-22:45<br>4 weai_time_10_cook___4 22:45-23:00<br>5 weai_time_10_cook___5 23:00-23:15<br>6 weai_time_10_cook___6 23:15-23:30<br>7 weai_time_10_cook___7 23:30-23:45<br>8 weai_time_10_cook___8 23:45-00:00
1222	weai_time_10_domestic	Domestic work (incl fetching wood and water)	checkbox<br>1 weai_time_10_domestic___1 22:00-22:15<br>2 weai_time_10_domestic___2 22:15-22:30<br>3 weai_time_10_domestic___3 22:30-22:45<br>4 weai_time_10_domestic___4 22:45-23:00<br>5 weai_time_10_domestic___5 23:00-23:15<br>6 weai_time_10_domestic___6 23:15-23:30<br>7 weai_time_10_domestic___7 23:30-23:45<br>8 weai_time_10_domestic___8 23:45-00:00
1223	weai_time_10_care	Care for children/adults/elderly	checkbox<br>1 weai_time_10_care___1 22:00-22:15<br>2 weai_time_10_care___2 22:15-22:30<br>3 weai_time_10_care___3 22:30-22:45<br>4 weai_time_10_care___4 22:45-23:00<br>5 weai_time_10_care___5 23:00-23:15<br>6 weai_time_10_care___6 23:15-23:30<br>7 weai_time_10_care___7 23:30-23:45<br>8 weai_time_10_care___8 23:45-00:00
1224	weai_time_10_travel	Travelling and communiting	checkbox<br>1 weai_time_10_travel___1 22:00-22:15<br>2 weai_time_10_travel___2 22:15-22:30<br>3 weai_time_10_travel___3 22:30-22:45<br>4 weai_time_10_travel___4 22:45-23:00<br>5 weai_time_10_travel___5 23:00-23:15<br>6 weai_time_10_travel___6 23:15-23:30<br>7 weai_time_10_travel___7 23:30-23:45<br>8 weai_time_10_travel___8 23:45-00:00
1225	weai_time_10_tv	Watching TV/listening to radio/reading	checkbox<br>1 weai_time_10_tv___1 22:00-22:15<br>2 weai_time_10_tv___2 22:15-22:30<br>3 weai_time_10_tv___3 22:30-22:45<br>4 weai_time_10_tv___4 22:45-23:00<br>5 weai_time_10_tv___5 23:00-23:15<br>6 weai_time_10_tv___6 23:15-23:30<br>7 weai_time_10_tv___7 23:30-23:45<br>8 weai_time_10_tv___8 23:45-00:00

1226	weai_time_10_exercising	Exercising	checkbox<br>1 weai_time_10_exercising___1 22:00-22:15<br>2 weai_time_10_exercising___2 22:15-22:30<br>3 weai_time_10_exercising___3 22:30-22:45<br>4 weai_time_10_exercising___4 22:45-23:00<br>5 weai_time_10_exercising___5 23:00-23:15<br>6 weai_time_10_exercising___6 23:15-23:30<br>7 weai_time_10_exercising___7 23:30-23:45<br>8 weai_time_10_exercising___8 23:45-00:00
1227	weai_time_10_social	Social activities and hobbies	checkbox<br>1 weai_time_10_social___1 22:00-22:15<br>2 weai_time_10_social___2 22:15-22:30<br>3 weai_time_10_social___3 22:30-22:45<br>4 weai_time_10_social___4 22:45-23:00<br>5 weai_time_10_social___5 23:00-23:15<br>6 weai_time_10_social___6 23:15-23:30<br>7 weai_time_10_social___7 23:30-23:45<br>8 weai_time_10_social___8 23:45-00:00
1228	weai_time_10_religious	Religious activities	checkbox<br>1 weai_time_10_religious___1 22:00-22:15<br>2 weai_time_10_religious___2 22:15-22:30<br>3 weai_time_10_religious___3 22:30-22:45<br>4 weai_time_10_religious___4 22:45-23:00<br>5 weai_time_10_religious___5 23:00-23:15<br>6 weai_time_10_religious___6 23:15-23:30<br>7 weai_time_10_religious___7 23:30-23:45<br>8 weai_time_10_religious___8 23:45-00:00
1229	weai_time_10_other	Other (specify below)	checkbox<br>1 weai_time_10_other___1 22:00-22:15<br>2 weai_time_10_other___2 22:15-22:30<br>3 weai_time_10_other___3 22:30-22:45<br>4 weai_time_10_other___4 22:45-23:00<br>5 weai_time_10_other___5 23:00-23:15<br>6 weai_time_10_other___6 23:15-23:30<br>7 weai_time_10_other___7 23:30-23:45<br>8 weai_time_10_other___8 23:45-00:00
1230	weai_time_10_other_specify<br>Show the field ONLY if: [weai_time_10_other(1)] or [weai_time_10_other(2)] or [weai_time_10_other(3)] or [weai_time_10_other(4)] or [weai_time_10_other(5)] or [weai_time_10_other(6)] or [weai_time_10_other(7)] or [weai_time_10_other(8)]	Specify Other	text<br>Custom alignment: LV

Supplemental material BMJ Publishing Group Limited (BMJ) disclaims all liability and responsibility arising from any reliance placed on this supplemental material which has been supplied by the author(s) BMJ Open

1231	weai_time_11_sleep	Section Header: *Night/Early morning*<br>Sleeping and resting	checkbox<br>1 weai_time_11_sleep___1 00:00-00:15<br>2 weai_time_11_sleep___2 00:15-00:30<br>3 weai_time_11_sleep___3 00:30-00:45<br>4 weai_time_11_sleep___4 00:45-01:00<br>5 weai_time_11_sleep___5 01:00-01:15<br>6 weai_time_11_sleep___6 01:15-01:30<br>7 weai_time_11_sleep___7 01:30-01:45<br>8 weai_time_11_sleep___8 01:45-02:00
1232	weai_time_11_eat	Eating and drinking	checkbox<br>1 weai_time_11_eat___1 00:00-00:15<br>2 weai_time_11_eat___2 00:15-00:30<br>3 weai_time_11_eat___3 00:30-00:45<br>4 weai_time_11_eat___4 00:45-01:00<br>5 weai_time_11_eat___5 01:00-01:15<br>6 weai_time_11_eat___6 01:15-01:30<br>7 weai_time_11_eat___7 01:30-01:45<br>8 weai_time_11_eat___8 01:45-02:00
1233	weai_time_11_personal	Personal Care	checkbox<br>1 weai_time_11_personal___1 00:00-00:15<br>2 weai_time_11_personal___2 00:15-00:30<br>3 weai_time_11_personal___3 00:30-00:45<br>4 weai_time_11_personal___4 00:45-01:00<br>5 weai_time_11_personal___5 01:00-01:15<br>6 weai_time_11_personal___6 01:15-01:30<br>7 weai_time_11_personal___7 01:30-01:45<br>8 weai_time_11_personal___8 01:45-02:00
1234	weai_time_11_school	School (also homework)	checkbox<br>1 weai_time_11_school___1 00:00-00:15<br>2 weai_time_11_school___2 00:15-00:30<br>3 weai_time_11_school___3 00:30-00:45<br>4 weai_time_11_school___4 00:45-01:00<br>5 weai_time_11_school___5 01:00-01:15<br>6 weai_time_11_school___6 01:15-01:30<br>7 weai_time_11_school___7 01:30-01:45<br>8 weai_time_11_school___8 01:45-02:00
1235	weai_time_11_work	Work as employed	checkbox<br>1 weai_time_11_work___1 00:00-00:15<br>2 weai_time_11_work___2 00:15-00:30<br>3 weai_time_11_work___3 00:30-00:45<br>4 weai_time_11_work___4 00:45-01:00<br>5 weai_time_11_work___5 01:00-01:15<br>6 weai_time_11_work___6 01:15-01:30<br>7 weai_time_11_work___7 01:30-01:45<br>8 weai_time_11_work___8 01:45-02:00

Havelaar AH, *et al. BMJ Open* 2022; 12:e061311. doi: 10.1136/bmjopen-2022-061311

1236	weai_time_11_buisness	Own business work	checkbox<br>1 weai_time_11_buisness___1 00:00-00:15<br>2 weai_time_11_buisness___2 00:15-00:30<br>3 weai_time_11_buisness___3 00:30-00:45<br>4 weai_time_11_buisness___4 00:45-01:00<br>5 weai_time_11_buisness___5 01:00-01:15<br>6 weai_time_11_buisness___6 01:15-01:30<br>7 weai_time_11_buisness___7 01:30-01:45<br>8 weai_time_11_buisness___8 01:45-02:00
1237	weai_time_11_farm	Farming/livestock/fishing	checkbox<br>1 weai_time_11_farm___1 00:00-00:15<br>2 weai_time_11_farm___2 00:15-00:30<br>3 weai_time_11_farm___3 00:30-00:45<br>4 weai_time_11_farm___4 00:45-01:00<br>5 weai_time_11_farm___5 01:00-01:15<br>6 weai_time_11_farm___6 01:15-01:30<br>7 weai_time_11_farm___7 01:30-01:45<br>8 weai_time_11_farm___8 01:45-02:00
1238	weai_time_11_shop	Shopping/getting service (incl health services)	checkbox<br>1 weai_time_11_shop___1 00:00-00:15<br>2 weai_time_11_shop___2 00:15-00:30<br>3 weai_time_11_shop___3 00:30-00:45<br>4 weai_time_11_shop___4 00:45-01:00<br>5 weai_time_11_shop___5 01:00-01:15<br>6 weai_time_11_shop___6 01:15-01:30<br>7 weai_time_11_shop___7 01:30-01:45<br>8 weai_time_11_shop___8 01:45-02:00
1239	weai_time_11_weave	Weaving, sewing, textile care	checkbox<br>1 weai_time_11_weave___1 00:00-00:15<br>2 weai_time_11_weave___2 00:15-00:30<br>3 weai_time_11_weave___3 00:30-00:45<br>4 weai_time_11_weave___4 00:45-01:00<br>5 weai_time_11_weave___5 01:00-01:15<br>6 weai_time_11_weave___6 01:15-01:30<br>7 weai_time_11_weave___7 01:30-01:45<br>8 weai_time_11_weave___8 01:45-02:00
1240	weai_time_11_cook	Cooking	checkbox<br>1 weai_time_11_cook___1 00:00-00:15<br>2 weai_time_11_cook___2 00:15-00:30<br>3 weai_time_11_cook___3 00:30-00:45<br>4 weai_time_11_cook___4 00:45-01:00<br>5 weai_time_11_cook___5 01:00-01:15<br>6 weai_time_11_cook___6 01:15-01:30<br>7 weai_time_11_cook___7 01:30-01:45<br>8 weai_time_11_cook___8 01:45-02:00

1241	weai_time_11_domestic	Domestic work (incl fetching wood and water)	checkbox<br>1 weai_time_11_domestic___1 00:00-00:15<br>2 weai_time_11_domestic___2 00:15-00:30<br>3 weai_time_11_domestic___3 00:30-00:45<br>4 weai_time_11_domestic___4 00:45-01:00<br>5 weai_time_11_domestic___5 01:00-01:15<br>6 weai_time_11_domestic___6 01:15-01:30<br>7 weai_time_11_domestic___7 01:30-01:45<br>8 weai_time_11_domestic___8 01:45-02:00
1242	weai_time_11_care	Care for children/adults/elderly	checkbox<br>1 weai_time_11_care___1 00:00-00:15<br>2 weai_time_11_care___2 00:15-00:30<br>3 weai_time_11_care___3 00:30-00:45<br>4 weai_time_11_care___4 00:45-01:00<br>5 weai_time_11_care___5 01:00-01:15<br>6 weai_time_11_care___6 01:15-01:30<br>7 weai_time_11_care___7 01:30-01:45<br>8 weai_time_11_care___8 01:45-02:00
1243	weai_time_11_travel	Travelling and communiting	checkbox<br>1 weai_time_11_travel___1 00:00-00:15<br>2 weai_time_11_travel___2 00:15-00:30<br>3 weai_time_11_travel___3 00:30-00:45<br>4 weai_time_11_travel___4 00:45-01:00<br>5 weai_time_11_travel___5 01:00-01:15<br>6 weai_time_11_travel___6 01:15-01:30<br>7 weai_time_11_travel___7 01:30-01:45<br>8 weai_time_11_travel___8 01:45-02:00
1244	weai_time_11_tv	Watching TV/listening to radio/reading	checkbox<br>1 weai_time_11_tv___1 00:00-00:15<br>2 weai_time_11_tv___2 00:15-00:30<br>3 weai_time_11_tv___3 00:30-00:45<br>4 weai_time_11_tv___4 00:45-01:00<br>5 weai_time_11_tv___5 01:00-01:15<br>6 weai_time_11_tv___6 01:15-01:30<br>7 weai_time_11_tv___7 01:30-01:45<br>8 weai_time_11_tv___8 01:45-02:00
1245	weai_time_11_exercising	Exercising	checkbox<br>1 weai_time_11_exercising___1 00:00-00:15<br>2 weai_time_11_exercising___2 00:15-00:30<br>3 weai_time_11_exercising___3 00:30-00:45<br>4 weai_time_11_exercising___4 00:45-01:00<br>5 weai_time_11_exercising___5 01:00-01:15<br>6 weai_time_11_exercising___6 01:15-01:30<br>7 weai_time_11_exercising___7 01:30-01:45<br>8 weai_time_11_exercising___8 01:45-02:00

Havelaar AH, *et al. BMJ Open* 2022; 12:e061311. doi: 10.1136/bmjopen-2022-061311

1246	weai_time_11_social	Social activities and hobbies	checkbox<br>1 weai_time_11_social___1 00:00-00:15<br>2 weai_time_11_social___2 00:15-00:30<br>3 weai_time_11_social___3 00:30-00:45<br>4 weai_time_11_social___4 00:45-01:00<br>5 weai_time_11_social___5 01:00-01:15<br>6 weai_time_11_social___6 01:15-01:30<br>7 weai_time_11_social___7 01:30-01:45<br>8 weai_time_11_social___8 01:45-02:00
1247	weai_time_11_religious	Religious activities	checkbox<br>1 weai_time_11_religious___1 00:00-00:15<br>2 weai_time_11_religious___2 00:15-00:30<br>3 weai_time_11_religious___3 00:30-00:45<br>4 weai_time_11_religious___4 00:45-01:00<br>5 weai_time_11_religious___5 01:00-01:15<br>6 weai_time_11_religious___6 01:15-01:30<br>7 weai_time_11_religious___7 01:30-01:45<br>8 weai_time_11_religious___8 01:45-02:00
1248	weai_time_11_other	Other (specify below)	checkbox<br>1 weai_time_11_other___1 00:00-00:15<br>2 weai_time_11_other___2 00:15-00:30<br>3 weai_time_11_other___3 00:30-00:45<br>4 weai_time_11_other___4 00:45-01:00<br>5 weai_time_11_other___5 01:00-01:15<br>6 weai_time_11_other___6 01:15-01:30<br>7 weai_time_11_other___7 01:30-01:45<br>8 weai_time_11_other___8 01:45-02:00
1249	weai_time_11_other_specify<br>Show the field ONLY if:<br>[weai_time_11_other(1)] or [weai_time_11_other(2)] or [weai_time_11_other(3)] or [weai_time_11_other(4)] or [weai_time_11_other(5)] or [weai_time_11_other(6)] or [weai_time_11_other(7)] or [weai_time_11_other(8)]	Specify Other	text<br>Custom alignment: LV
1250	weai_time_12_sleep	Section Header: *Night/Early morning*<br>Sleeping and resting	checkbox<br>1 weai_time_12_sleep___1 02:00-02:15<br>2 weai_time_12_sleep___2 02:15-02:30<br>3 weai_time_12_sleep___3 02:30-02:45<br>4 weai_time_12_sleep___4 02:45-03:00<br>5 weai_time_12_sleep___5 03:00-03:15<br>6 weai_time_12_sleep___6 03:15-03:30<br>7 weai_time_12_sleep___7 03:30-03:45<br>8 weai_time_12_sleep___8 03:45-04:00

Havelaar AH, *et al. BMJ Open* 2022; 12:e061311. doi: 10.1136/bmjopen-2022-061311

1251	weai_time_12_eat	Eating and drinking	checkbox<br>1 weai_time_12_eat___1 02:00-02:15<br>2 weai_time_12_eat___2 02:15-02:30<br>3 weai_time_12_eat___3 02:30-02:45<br>4 weai_time_12_eat___4 02:45-03:00<br>5 weai_time_12_eat___5 03:00-03:15<br>6 weai_time_12_eat___6 03:15-03:30<br>7 weai_time_12_eat___7 03:30-03:45<br>8 weai_time_12_eat___8 03:45-04:00
1252	weai_time_12_personal	Personal Care	checkbox<br>1 weai_time_12_personal___1 02:00-02:15<br>2 weai_time_12_personal___2 02:15-02:30<br>3 weai_time_12_personal___3 02:30-02:45<br>4 weai_time_12_personal___4 02:45-03:00<br>5 weai_time_12_personal___5 03:00-03:15<br>6 weai_time_12_personal___6 03:15-03:30<br>7 weai_time_12_personal___7 03:30-03:45<br>8 weai_time_12_personal___8 03:45-04:00
1253	weai_time_12_school	School (also homework)	checkbox<br>1 weai_time_12_school___1 02:00-02:15<br>2 weai_time_12_school___2 02:15-02:30<br>3 weai_time_12_school___3 02:30-02:45<br>4 weai_time_12_school___4 02:45-03:00<br>5 weai_time_12_school___5 03:00-03:15<br>6 weai_time_12_school___6 03:15-03:30<br>7 weai_time_12_school___7 03:30-03:45<br>8 weai_time_12_school___8 03:45-04:00
1254	weai_time_12_work	Work as employed	checkbox<br>1 weai_time_12_work___1 02:00-02:15<br>2 weai_time_12_work___2 02:15-02:30<br>3 weai_time_12_work___3 02:30-02:45<br>4 weai_time_12_work___4 02:45-03:00<br>5 weai_time_12_work___5 03:00-03:15<br>6 weai_time_12_work___6 03:15-03:30<br>7 weai_time_12_work___7 03:30-03:45<br>8 weai_time_12_work___8 03:45-04:00
1255	weai_time_12_buisness	Own business work	checkbox<br>1 weai_time_12_buisness___1 02:00-02:15<br>2 weai_time_12_buisness___2 02:15-02:30<br>3 weai_time_12_buisness___3 02:30-02:45<br>4 weai_time_12_buisness___4 02:45-03:00<br>5 weai_time_12_buisness___5 03:00-03:15<br>6 weai_time_12_buisness___6 03:15-03:30<br>7 weai_time_12_buisness___7 03:30-03:45<br>8 weai_time_12_buisness___8 03:45-04:00

1256	weai_time_12_farm	Farming/livestock/fishing	checkbox<br>1 weai_time_12_farm___1 02:00-02:15<br>2 weai_time_12_farm___2 02:15-02:30<br>3 weai_time_12_farm___3 02:30-02:45<br>4 weai_time_12_farm___4 02:45-03:00<br>5 weai_time_12_farm___5 03:00-03:15<br>6 weai_time_12_farm___6 03:15-03:30<br>7 weai_time_12_farm___7 03:30-03:45<br>8 weai_time_12_farm___8 03:45-04:00
1257	weai_time_12_shop	Shopping/getting service (incl health services)	checkbox<br>1 weai_time_12_shop___1 02:00-02:15<br>2 weai_time_12_shop___2 02:15-02:30<br>3 weai_time_12_shop___3 02:30-02:45<br>4 weai_time_12_shop___4 02:45-03:00<br>5 weai_time_12_shop___5 03:00-03:15<br>6 weai_time_12_shop___6 03:15-03:30<br>7 weai_time_12_shop___7 03:30-03:45<br>8 weai_time_12_shop___8 03:45-04:00
1258	weai_time_12_weave	Weaving, sewing, textile care	checkbox<br>1 weai_time_12_weave___1 02:00-02:15<br>2 weai_time_12_weave___2 02:15-02:30<br>3 weai_time_12_weave___3 02:30-02:45<br>4 weai_time_12_weave___4 02:45-03:00<br>5 weai_time_12_weave___5 03:00-03:15<br>6 weai_time_12_weave___6 03:15-03:30<br>7 weai_time_12_weave___7 03:30-03:45<br>8 weai_time_12_weave___8 03:45-04:00
1259	weai_time_12_cook	Cooking	checkbox<br>1 weai_time_12_cook___1 02:00-02:15<br>2 weai_time_12_cook___2 02:15-02:30<br>3 weai_time_12_cook___3 02:30-02:45<br>4 weai_time_12_cook___4 02:45-03:00<br>5 weai_time_12_cook___5 03:00-03:15<br>6 weai_time_12_cook___6 03:15-03:30<br>7 weai_time_12_cook___7 03:30-03:45<br>8 weai_time_12_cook___8 03:45-04:00
1260	weai_time_12_domestic	Domestic work (incl fetching wood and water)	checkbox<br>1 weai_time_12_domestic___1 02:00-02:15<br>2 weai_time_12_domestic___2 02:15-02:30<br>3 weai_time_12_domestic___3 02:30-02:45<br>4 weai_time_12_domestic___4 02:45-03:00<br>5 weai_time_12_domestic___5 03:00-03:15<br>6 weai_time_12_domestic___6 03:15-03:30<br>7 weai_time_12_domestic___7 03:30-03:45<br>8 weai_time_12_domestic___8 03:45-04:00

1261	weai_time_12_care	Care for children/adults/elderly	checkbox			
				1	weai_time_12_care___1	02:00-02:15
				2	weai_time_12_care___2	02:15-02:30
				3	weai_time_12_care___3	02:30-02:45
				4	weai_time_12_care___4	02:45-03:00
				5	weai_time_12_care___5	03:00-03:15
				6	weai_time_12_care___6	03:15-03:30
				7	weai_time_12_care___7	03:30-03:45
				8	weai_time_12_care___8	03:45-04:00
1262	weai_time_12_travel	Travelling and communiting	checkbox			
				1	weai_time_12_travel___1	02:00-02:15
				2	weai_time_12_travel___2	02:15-02:30
				3	weai_time_12_travel___3	02:30-02:45
				4	weai_time_12_travel___4	02:45-03:00
				5	weai_time_12_travel___5	03:00-03:15
				6	weai_time_12_travel___6	03:15-03:30
				7	weai_time_12_travel___7	03:30-03:45
				8	weai_time_12_travel___8	03:45-04:00
1263	weai_time_12_tv	Watching TV/listening to radio/reading	checkbox			
				1	weai_time_12_tv___1	02:00-02:15
				2	weai_time_12_tv___2	02:15-02:30
				3	weai_time_12_tv___3	02:30-02:45
				4	weai_time_12_tv___4	02:45-03:00
				5	weai_time_12_tv___5	03:00-03:15
				6	weai_time_12_tv___6	03:15-03:30
				7	weai_time_12_tv___7	03:30-03:45
				8	weai_time_12_tv___8	03:45-04:00
1264	weai_time_12_exercising	Exercising	checkbox			
				1	weai_time_12_exercising___1	02:00-02:15
				2	weai_time_12_exercising___2	02:15-02:30
				3	weai_time_12_exercising___3	02:30-02:45
				4	weai_time_12_exercising___4	02:45-03:00
				5	weai_time_12_exercising___5	03:00-03:15
				6	weai_time_12_exercising___6	03:15-03:30
				7	weai_time_12_exercising___7	03:30-03:45
				8	weai_time_12_exercising___8	03:45-04:00
1265	weai_time_12_social	Social activities and hobbies	checkbox			
				1	weai_time_12_social___1	02:00-02:15
				2	weai_time_12_social___2	02:15-02:30
				3	weai_time_12_social___3	02:30-02:45
				4	weai_time_12_social___4	02:45-03:00
				5	weai_time_12_social___5	03:00-03:15
				6	weai_time_12_social___6	03:15-03:30
				7	weai_time_12_social___7	03:30-03:45
				8	weai_time_12_social___8	03:45-04:00

Havelaar AH, *et al. BMJ Open* 2022; 12:e061311. doi: 10.1136/bmjopen-2022-061311

1266	weai_time_12_religious	Religious activities	checkbox<br>1 weai_time_12_religious___1 02:00-02:15<br>2 weai_time_12_religious___2 02:15-02:30<br>3 weai_time_12_religious___3 02:30-02:45<br>4 weai_time_12_religious___4 02:45-03:00<br>5 weai_time_12_religious___5 03:00-03:15<br>6 weai_time_12_religious___6 03:15-03:30<br>7 weai_time_12_religious___7 03:30-03:45<br>8 weai_time_12_religious___8 03:45-04:00
1267	weai_time_12_other	Other (specify below)	checkbox<br>1 weai_time_12_other___1 02:00-02:15<br>2 weai_time_12_other___2 02:15-02:30<br>3 weai_time_12_other___3 02:30-02:45<br>4 weai_time_12_other___4 02:45-03:00<br>5 weai_time_12_other___5 03:00-03:15<br>6 weai_time_12_other___6 03:15-03:30<br>7 weai_time_12_other___7 03:30-03:45<br>8 weai_time_12_other___8 03:45-04:00
1268	weai_time_12_other_specify<br>Show the field ONLY if: [weai_time_12_other(1)] or [weai_time_12_other(2)] or [weai_time_12_other(3)] or [weai_time_12_other(4)] or [weai_time_12_other(5)] or [weai_time_12_other(6)] or [weai_time_12_other(7)] or [weai_time_12_other(8)]	Specify Other	text<br>Custom alignment: LV
1269	weai_24hrs_work	2) In the last 24 hours did you work (at home or outside of the home) more than usual, about the same as usual, or less than usual?	radio<br>1 More than usual<br>2 About the same as usual<br>3 Less than usual<br>Custom alignment: LV
1270	weai_group_ag	Section Header: *"Now I'm going to ask you about groups in the community. These can be either formal or informal and customary groups."*<br>3) Is there an Agricultural / livestock/ fisheries producer's group (including marketing groups) in your community?	radio<br>1 Yes<br>2 No<br>97 Don't know<br>Custom alignment: LV
1271	weai_group_ag_active<br>Show the field ONLY if: [weai_group_ag] = '1'	3a) Are you an active member of this [Agricultural / livestock/ fisheries producer's group (including marketing groups)]?	radio<br>1 Yes<br>2 No<br>Custom alignment: LV
1272	weai_group_water	4) Is there a Water users' group in your community?	radio<br>1 Yes<br>2 No<br>97 Don't know<br>Custom alignment: LV
1273	weai_group_water_active<br>Show the field ONLY if: [weai_group_water] = '1'	4a) Are you an active member of this Water users' group?	radio<br>1 Yes<br>2 No<br>Custom alignment: LV

1274	weai_group_forest	5) Is there a Forest users' group in your community?	radio<br>1 Yes<br>2 No<br>97 Don't know<br><br>Custom alignment: LV
1275	weai_group_forest_active<br><br>Show the field ONLY if:<br>[weai_group_forest] = '1'	5a) Are you an active member of this Forest users' group?	radio<br>1 Yes<br>2 No<br><br>Custom alignment: LV
1276	weai_group_credit	6) Is there a Credit or microfinance group (including SACCOs/merry-go-rounds/ VSLAs) in your community?	radio<br>1 Yes<br>2 No<br>97 Don't know<br><br>Custom alignment: LV
1277	weai_group_credit_active<br><br>Show the field ONLY if:<br>[weai_group_credit] = '1'	6a) Are you an active member of this Credit or microfinance group (including SACCOs/merry-go-rounds/ VSLAs)?	radio<br>1 Yes<br>2 No<br><br>Custom alignment: LV
1278	weai_group_mutual	7) Is there a Mutual help or insurance group (including burial societies) in your community?	radio<br>1 Yes<br>2 No<br>97 Don't know<br><br>Custom alignment: LV
1279	weai_group_mutual_active<br><br>Show the field ONLY if:<br>[weai_group_mutual] = '1'	7a) Are you an active member of this Mutual help or insurance group (including burial societies)?	radio<br>1 Yes<br>2 No<br><br>Custom alignment: LV
1280	weai_group_trade	8) Is there a Trade and business association group in your community?	radio<br>1 Yes<br>2 No<br>97 Don't know<br><br>Custom alignment: LV
1281	weai_group_trade_active<br><br>Show the field ONLY if:<br>[weai_group_trade] = '1'	8a) Are you an active member of this Trade and business association group?	radio<br>1 Yes<br>2 No<br><br>Custom alignment: LV
1282	weai_group_civics	9) Is there a Civic groups (improving community) or charitable group (helping others) in your community?	radio<br>1 Yes<br>2 No<br>97 Don't know<br><br>Custom alignment: LV
1283	weai_group_civics_active<br><br>Show the field ONLY if:<br>[weai_group_civics] = '1'	9a) Are you an active member of this Civic groups (improving community) or charitable group (helping others)?	radio<br>1 Yes<br>2 No<br><br>Custom alignment: LV

1284	weai_group_religious	10) Is there a Religious group in your community?	radio<br>1 Yes<br>2 No<br>97 Don't know<br><br>Custom alignment: LV
1285	weai_group_religion_active<br><br>Show the field ONLY if:<br>[weai_group_religious] = '1'	10a) Are you an active member of this Religious group?	radio<br>1 Yes<br>2 No<br><br>Custom alignment: LV
1286	weai_group_other	11) Is there an Other [women's/men's] group (only if it does not fit into one of the other categories) in your community?	radio<br>1 Yes<br>2 No<br>97 Don't know<br><br>Custom alignment: LV
1287	weai_group_other_active<br><br>Show the field ONLY if:<br>[weai_group_other] = '1'	11a) Are you an active member of this Other [women's/men's] group?	radio<br>1 Yes<br>2 No<br><br>Custom alignment: LV
1288	weai_group_other2	12) Is there an Other [specify type below] group in your community?	radio<br>1 Yes<br>2 No<br>97 Don't know<br><br>Custom alignment: LV
1289	weai_group_other2_specify<br><br>Show the field ONLY if:<br>[weai_group_other2] = '1'	12a) Specify Other group:	text<br>Custom alignment: LV
1290	weai_group_other2_active<br><br>Show the field ONLY if:<br>[weai_group_other2] = '1'	12b) Are you an active member of this Other group?	radio<br>1 Yes<br>2 No<br><br>Custom alignment: LV
1291	weai_outcome	2) OUTCOME OF INTERVIEW:	radio<br>1 COMPLETED<br>2 HOUSEHOLD MEMBER TOO ILL TO RESPOND/COGNITIVELY IMPAIRED<br>3 RESPONDENT NOT AT HOME/TEMPORARILY UNAVAILABLE<br>4 RESPONDENT NOT AT HOME/EXTENDED ABSENCE<br>5 REFUSED<br>6 COULD NOT LOCATE<br><br>Custom alignment: LV
1292	aweai_female_complete	Section Header: *Form Status*<br><br>Complete?	dropdown<br>0 Incomplete<br>1 Unverified<br>2 Complete

Instrument: **A-WEAI (Male)** (aweai_male)

Collapse

1293	weai_inter_gender_m	1) What is the gender of the person being interviewed?	radio<br>1 Male<br>2 Female<br><br>Custom alignment: LV
1294	weai_hh_type_m	2) TYPE OF HOUSEHOLD	radio<br>1 MALE AND FEMALE ADULT<br>2 FEMALE ADULT ONLY<br><br>Custom alignment: LV
1295	weai_alone_m	3) ABILITY TO BE INTERVIEWED ALONE:	radio<br>1 ALONE<br>2 WITH ADULT FEMALES PRESENT<br>3 WITH ADULT MALES PRESENT<br>4 WITH ADULTS MIXED SEX PRESENT<br>5 WITH CHILDREN PRESENT<br>6 WITH ADULTS MIXED SEX AND CHILDREN PRESENT<br><br>Custom alignment: LV
1296	weai_crop_m	Section Header: *"Now I'd like to ask you some questions about your participation in certain types of work activities and on making decisions on various aspects of household life"*<br>4) Did you yourself participate in Food crop farming: These are crops that are grown primarily for household food consumption in the past 12 months (that is, during the last [one/two] cropping seasons), from [PRESENT MONTH] last year to [PRESENT MONTH] this year?	radio<br>1 Yes<br>2 No<br><br>Custom alignment: LV
1297	weai_crop_decisions_m<br>Show the field ONLY if:<br>[weai_crop_m] = '1'	4a) When decisions are made regarding Food crop farming, who is it that normally takes the decision?<br>*Check all that apply*	checkbox<br>1 weai_crop_decisions_m___1 SELF<br>2 weai_crop_decisions_m___2 SPOUSE<br>3 weai_crop_decisions_m___3 OTHER HH MEMBER<br>4 weai_crop_decisions_m___4 OTHER NON-HH MEMBER<br>98 weai_crop_decisions_m___98 NOT APPLICABLE<br><br>Custom alignment: LV
1298	weai_crop_input_m<br>Show the field ONLY if:<br>[weai_crop_decisions_m(2)] = '1' or [weai_crop_decisions_m(3)] = '1' or [weai_crop_decisions_m(4)] = '1'	4b) How much input did you have in making decisions about food crop farming?	radio<br>1 NO INPUT OR INPUT IN FEW DECISIONS<br>2 INPUT INTO SOME DECISIONS<br>3 INPUT INTO MOST OR ALL DECISIONS<br>98 NO DECISION MADE<br><br>Custom alignment: LV
1299	weai_crop_personal_m<br>Show the field ONLY if:<br>([weai_crop_input_m] = '1' or [weai_crop_input_m] = '2' or [weai_crop_input_m] = '3')	4c) To what extent do you feel you can make your own personal decisions regarding food crop farming if you want(ed) to?	radio<br>1 NOT AT ALL<br>2 SMALL EXTENT<br>3 MEDIUM EXTENT<br>4 TO A HIGH EXTENT<br><br>Custom alignment: LV

1300	weai_crop_income_m<br><br>Show the field ONLY if:<br>([weai_crop_input_m] = '1' or [weai_crop_input_m] = '2' or [weai_crop_input_m] = '3') or ([weai_crop_decisions_m(1)] = '1' and [weai_crop_decisions_m(2)] = '0' and [weai_crop_decisions_m(4)] = '0' and [weai_crop_decisions_m(98)] = '0')	4d) How much input did you have in decisions on the use of income generated from food crop farming?	radio<br>1 NO INPUT OR INPUT IN FEW DECISIONS<br>2 INPUT INTO SOME DECISIONS<br>3 INPUT INTO MOST OR ALL DECISIONS<br>98 NO DECISION MADE<br><br>Custom alignment: LV
1301	weai_cash_m	5) Did you yourself participate in Cash crop farming: These are crops that are grown primarily for sale in the market in the past 12 months (that is, during the last [one/two] cropping seasons), from [PRESENT MONTH] last year to [PRESENT MONTH] this year?	radio<br>1 Yes<br>2 No<br><br>Custom alignment: LV
1302	weai_cash_decisions_m<br><br>Show the field ONLY if:<br>[weai_cash_m] = '1'	5a) When decisions are made regarding cash crop farming, who is it that normally takes the decision?<br>*Check all that apply*	checkbox<br>1 weai_cash_decisions_m___1 SELF<br>2 weai_cash_decisions_m___2 SPOUSE<br>3 weai_cash_decisions_m___3 OTHER HH MEMBER<br>4 weai_cash_decisions_m___4 OTHER NON-HH MEMBER<br>98 weai_cash_decisions_m___98 NOT APPLICABLE<br><br>Custom alignment: LV
1303	weai_cash_input_m<br><br>Show the field ONLY if:<br>[weai_cash_decisions_m(2)] = '1' or [weai_cash_decisions_m(3)] = '1' or [weai_cash_decisions_m(4)] = '1'	5b) How much input did you have in making decisions about cash crop farming?	radio<br>1 NO INPUT OR INPUT IN FEW DECISIONS<br>2 INPUT INTO SOME DECISIONS<br>3 INPUT INTO MOST OR ALL DECISIONS<br>98 NO DECISION MADE<br><br>Custom alignment: LV
1304	weai_cash_personal_m<br><br>Show the field ONLY if:<br>[weai_cash_input_m] = '1' or [weai_cash_input_m] = '2' or [weai_cash_input_m] = '3'	5c) To what extent do you feel you can make your own personal decisions regarding cash crop farming if you want(ed) to?	radio<br>1 NOT AT ALL<br>2 SMALL EXTENT<br>3 MEDIUM EXTENT<br>4 TO A HIGH EXTENT<br><br>Custom alignment: LV
1305	weai_cash_income_m<br><br>Show the field ONLY if:<br>([weai_cash_input_m] = '1' or [weai_cash_input_m] = '2' or [weai_cash_input_m] = '3') or ([weai_cash_decisions_m(1)] = '1' and [weai_cash_decisions_m(2)] = '0' and [weai_cash_decisions_m(4)] = '0' and [weai_cash_decisions_m(98)] = '0')	5d) How much input did you have in decisions on the use of income generated from cash crop farming?	radio<br>1 NO INPUT OR INPUT IN FEW DECISIONS<br>2 INPUT INTO SOME DECISIONS<br>3 INPUT INTO MOST OR ALL DECISIONS<br>98 NO DECISION MADE<br><br>Custom alignment: LV
1306	weai_livestock_m	6) Did you yourself participate in livestock raising in the past 12 months (that is, during the last [one/two] cropping seasons), from [PRESENT MONTH] last year to [PRESENT MONTH] this year?	radio<br>1 Yes<br>2 No<br><br>Custom alignment: LV

1307	weai_livestock_decisions_m<br>Show the field ONLY if:<br>[weai_livestock_m] = '1'	6a) When decisions are made regarding livestock raising, who is it that normally takes the decision?<br>*Check all that apply*	checkbox<br>1 weai_livestock_decisions_m___1 SELF<br>2 weai_livestock_decisions_m___2 SPOUSE<br>3 weai_livestock_decisions_m___3 OTHER HH MEMBER<br>4 weai_livestock_decisions_m___4 OTHER NON-HH MEMBER<br>98 weai_livestock_decisions_m___98 NOT APPLICABLE<br>Custom alignment: LV
1308	weai_livestock_input_m<br>Show the field ONLY if:<br>[weai_livestock_decisions_m(2)] = '1' or [weai_livestock_decisions_m(3)] = '1' or [weai_livestock_decisions_m(4)] = '1'	6b) How much input did you have in making decisions about livestock raising?	radio<br>1 NO INPUT OR INPUT IN FEW DECISIONS<br>2 INPUT INTO SOME DECISIONS<br>3 INPUT INTO MOST OR ALL DECISIONS<br>98 NO DECISION MADE<br>Custom alignment: LV
1309	weai_livestock_personal_m<br>Show the field ONLY if:<br>[weai_livestock_input_m] = '1' or [weai_livestock_input_m] = '2' or [weai_livestock_input_m] = '3'	6c) To what extent do you feel you can make your own personal decisions regarding livestock raising if you want(ed) to?	radio<br>1 NOT AT ALL<br>2 SMALL EXTENT<br>3 MEDIUM EXTENT<br>4 TO A HIGH EXTENT<br>Custom alignment: LV
1310	weai_livestock_income_m<br>Show the field ONLY if:<br>([weai_livestock_input_m] = '1' or [weai_livestock_input_m] = '2' or [weai_livestock_input_m] = '3') or ([weai_livestock_decisions_m(1)] = '1' and [weai_livestock_decisions_m(2)] = '0' and [weai_livestock_decisions_m(4)] = '0' and [weai_livestock_decisions_m(98)] = '0')	6d) How much input did you have in decisions on the use of income generated from livestock raising?	radio<br>1 NO INPUT OR INPUT IN FEW DECISIONS<br>2 INPUT INTO SOME DECISIONS<br>3 INPUT INTO MOST OR ALL DECISIONS<br>98 NO DECISION MADE<br>Custom alignment: LV
1311	weai_economic_m	7) Did you yourself participate in Non-farm economic activities: This would include things like running a small business, self-employment, buy-and-sell in the past 12 months (that is, during the last [one/two] cropping seasons), from [PRESENT MONTH] last year to [PRESENT MONTH] this year?	radio<br>1 Yes<br>2 No<br>Custom alignment: LV
1312	weai_economic_decisions_m<br>Show the field ONLY if:<br>[weai_economic_m] = '1'	7a) When decisions are made regarding Non-farm economic activities, who is it that normally takes the decision?<br>*Check all that apply*	checkbox<br>1 weai_economic_decisions_m___1 SELF<br>2 weai_economic_decisions_m___2 SPOUSE<br>3 weai_economic_decisions_m___3 OTHER HH MEMBER<br>4 weai_economic_decisions_m___4 OTHER NON-HH MEMBER<br>98 weai_economic_decisions_m___98 NOT APPLICABLE<br>Custom alignment: LV
1313	weai_economic_input_m<br>Show the field ONLY if:<br>[weai_economic_decisions_m(2)] = '1' or [weai_economic_decisions_m(3)] = '1' or [weai_economic_decisions_m(4)] = '1'	7b) How much input did you have in making decisions about Non-farm economic activities?	radio<br>1 NO INPUT OR INPUT IN FEW DECISIONS<br>2 INPUT INTO SOME DECISIONS<br>3 INPUT INTO MOST OR ALL DECISIONS<br>98 NO DECISION MADE<br>Custom alignment: LV

1314	weai_economic_personal_m<br>Show the field ONLY if: [weai_economic_input_m] = '1' or [weai_economic_input_m] = '2' or [weai_economic_input_m] = '3'	7c) To what extent do you feel you can make your own personal decisions regarding Non-farm economic activities if you want(ed) to?	radio<br>1 NOT AT ALL<br>2 SMALL EXTENT<br>3 MEDIUM EXTENT<br>4 TO A HIGH EXTENT<br>Custom alignment: LV
1315	weai_economic_income_m<br>Show the field ONLY if: ([weai_economic_input_m] = '1' or [weai_economic_input_m] = '2' or [weai_economic_input_m] = '3') or ([weai_economic_decisions_m(1)] = '1' and [weai_economic_decisions_m(2)] = '0' and [weai_economic_decisions_m(4)] = '0' and [weai_economic_decisions_m(98)] = '0')	7d) How much input did you have in decisions on the use of income generated from non-farm economic activities?	radio<br>1 NO INPUT OR INPUT IN FEW DECISIONS<br>2 INPUT INTO SOME DECISIONS<br>3 INPUT INTO MOST OR ALL DECISIONS<br>98 NO DECISION MADE<br>Custom alignment: LV
1316	weai_employ_m	8) Did you yourself participate in Wage and salary employment: This could be work that is paid for in cash or in-kind, including both agriculture and other wage work in the past 12 months (that is, during the last [one/two] cropping seasons), from [PRESENT MONTH] last year to [PRESENT MONTH] this year?	radio<br>1 Yes<br>2 No<br>Custom alignment: LV
1317	weai_employ_decisions_m<br>Show the field ONLY if: [weai_employ_m] = '1'	8a) When decisions are made regarding wage and salary employment, who is it that normally takes the decision?<br>*Check all that apply*	checkbox<br>1 weai_employ_decisions_m___1 SELF<br>2 weai_employ_decisions_m___2 SPOUSE<br>3 weai_employ_decisions_m___3 OTHER HH MEMBER<br>4 weai_employ_decisions_m___4 OTHER NON-HH MEMBER<br>98 weai_employ_decisions_m___98 NOT APPLICABLE<br>Custom alignment: LV
1318	weai_employ_input_m<br>Show the field ONLY if: [weai_employ_decisions_m(2)] = '1' or [weai_employ_decisions_m(3)] = '1' or [weai_employ_decisions_m(4)] = '1'	8b) How much input did you have in making decisions about wage and salary employment?	radio<br>1 NO INPUT OR INPUT IN FEW DECISIONS<br>2 INPUT INTO SOME DECISIONS<br>3 INPUT INTO MOST OR ALL DECISIONS<br>98 NO DECISION MADE<br>Custom alignment: LV
1319	weai_employ_personal_m<br>Show the field ONLY if: [weai_employ_input_m] = '1' or [weai_employ_input_m] = '2' or [weai_employ_input_m] = '3'	8c) To what extent do you feel you can make your own personal decisions regarding wage and salary employment if you want(ed) to?	radio<br>1 NOT AT ALL<br>2 SMALL EXTENT<br>3 MEDIUM EXTENT<br>4 TO A HIGH EXTENT<br>Custom alignment: LV
1320	weai_employ_income_m<br>Show the field ONLY if: ([weai_employ_input_m] = '1' or [weai_employ_input_m] = '2' or [weai_employ_input_m] = '3') or ([weai_employ_decisions_m(1)] = '1' and [weai_employ_decisions_m(2)] = '0' and [weai_employ_decisions_m(4)] = '0' and [weai_employ_decisions_m(98)] = '0')	8d) How much input did you have in decisions on the use of income generated from wage and salary employment?	radio<br>1 NO INPUT OR INPUT IN FEW DECISIONS<br>2 INPUT INTO SOME DECISIONS<br>3 INPUT INTO MOST OR ALL DECISIONS<br>98 NO DECISION MADE<br>Custom alignment: LV

1321	weai_fishing_m	9) Did you yourself participate in Fishing or fishpond culture in the past 12 months (that is, during the last [one/two] cropping seasons), from [PRESENT MONTH] last year to [PRESENT MONTH] this year?	radio<br>1 Yes<br>2 No<br>Custom alignment: LV
1322	weai_fishing_decisions_m<br>Show the field ONLY if:<br>[weai_fishing_m] = '1'	9a) When decisions are made regarding Fishing or fishpond culture, who is it that normally takes the decision?<br>*Check all that apply*	checkbox<br>1 weai_fishing_decisions_m___1 SELF<br>2 weai_fishing_decisions_m___2 SPOUSE<br>3 weai_fishing_decisions_m___3 OTHER HH MEMBER<br>4 weai_fishing_decisions_m___4 OTHER NON-HH MEMBER<br>98 weai_fishing_decisions_m___98 NOT APPLICABLE<br>Custom alignment: LV
1323	weai_fishing_input_m<br>Show the field ONLY if:<br>[weai_fishing_decisions_m(2)] = '1' or [weai_fishing_decisions_m(3)] = '1' or [weai_fishing_decisions_m(4)] = '1'	9b) How much input did you have in making decisions about Fishing or fishpond culture?	radio<br>1 NO INPUT OR INPUT IN FEW DECISIONS<br>2 INPUT INTO SOME DECISIONS<br>3 INPUT INTO MOST OR ALL DECISIONS<br>98 NO DECISION MADE<br>Custom alignment: LV
1324	weai_fishing_personal_m<br>Show the field ONLY if:<br>[weai_fishing_input_m] = '1' or [weai_fishing_input_m] = '2' or [weai_fishing_input_m] = '3'	9c) To what extent do you feel you can make your own personal decisions regarding Fishing or fishpond culture if you want(ed) to?	radio<br>1 NOT AT ALL<br>2 SMALL EXTENT<br>3 MEDIUM EXTENT<br>4 TO A HIGH EXTENT<br>Custom alignment: LV
1325	weai_fishing_income_m<br>Show the field ONLY if:<br>([weai_fishing_input_m] = '1' or [weai_fishing_input_m] = '2' or [weai_fishing_input_m] = '3') or ([weai_fishing_decisions_m(1)] = '1' and [weai_fishing_decisions_m(2)] = '0' and [weai_fishing_decisions_m(4)] = '0' and [weai_fishing_decisions_m(98)] = '0')	9d) How much input did you have in decisions on the use of income generated from Fishing or fishpond culture?	radio<br>1 NO INPUT OR INPUT IN FEW DECISIONS<br>2 INPUT INTO SOME DECISIONS<br>3 INPUT INTO MOST OR ALL DECISIONS<br>98 NO DECISION MADE<br>Custom alignment: LV
1326	weai_house_decisions_m	10) When decisions are made regarding Major household expenditures (such as bicycles, land, boda boda), who is it that normally takes the decision?<br>*Check all that apply*	checkbox<br>1 weai_house_decisions_m___1 SELF<br>2 weai_house_decisions_m___2 SPOUSE<br>3 weai_house_decisions_m___3 OTHER HH MEMBER<br>4 weai_house_decisions_m___4 OTHER NON-HH MEMBER<br>98 weai_house_decisions_m___98 NOT APPLICABLE<br>Custom alignment: LV
1327	weai_house_input_m<br>Show the field ONLY if:<br>[weai_house_decisions_m(2)] = '1' or [weai_house_decisions_m(3)] = '1' or [weai_house_decisions_m(4)] = '1'	10a) How much input did you have in making decisions about Major household expenditures?	radio<br>1 NO INPUT OR INPUT IN FEW DECISIONS<br>2 INPUT INTO SOME DECISIONS<br>3 INPUT INTO MOST OR ALL DECISIONS<br>98 NO DECISION MADE<br>Custom alignment: LV

1328	weai_house_personal_m<br>Show the field ONLY if:<br>[weai_house_input_m] = '1' or [weai_house_input_m] = '2' or [weai_house_input_m] = '3'	10b) To what extent do you feel you can make your own personal decisions regarding Major household expenditures if you want(ed) to?	radio<br>1 NOT AT ALL<br>2 SMALL EXTENT<br>3 MEDIUM EXTENT<br>4 TO A HIGH EXTENT<br>Custom alignment: LV
1329	weai_minor_decisions_m	11) When decisions are made regarding Minor household expenditures (such as food for daily consumption or other household needs), who is it that normally takes the decision?<br>*Check all that apply*	checkbox<br>1 weai_minor_decisions_m___1 SELF<br>2 weai_minor_decisions_m___2 SPOUSE<br>3 weai_minor_decisions_m___3 OTHER HH MEMBER<br>4 weai_minor_decisions_m___4 OTHER NON-HH MEMBER<br>98 weai_minor_decisions_m___98 NOT APPLICABLE<br>Custom alignment: LV
1330	weai_minor_input_m<br>Show the field ONLY if:<br>[weai_minor_decisions_m(2)] = '1' or [weai_minor_decisions_m(3)] = '1' or [weai_minor_decisions_m(4)] = '1'	11a) How much input did you have in making decisions about Minor household expenditures?	radio<br>1 NO INPUT OR INPUT IN FEW DECISIONS<br>2 INPUT INTO SOME DECISIONS<br>3 INPUT INTO MOST OR ALL DECISIONS<br>98 NO DECISION MADE<br>Custom alignment: LV
1331	weai_minor_personal_m<br>Show the field ONLY if:<br>[weai_minor_input_m] = '1' or [weai_minor_input_m] = '2' or [weai_minor_input_m] = '3'	11b) To what extent do you feel you can make your own personal decisions regarding Minor household expenditures if you want(ed) to?	radio<br>1 NOT AT ALL<br>2 SMALL EXTENT<br>3 MEDIUM EXTENT<br>4 TO A HIGH EXTENT<br>Custom alignment: LV
1332	weai_own_agri_m	Section Header: *"Now I'd like to ask you about your household's access to and ownership of a number of items that could be used to generate income."*<br>12) Does anyone in your household currently have any agricultural land (pieces/plots)?	radio<br>1 Yes<br>2 No<br>Custom alignment: LV
1333	weai_own_agri_self_m<br>Show the field ONLY if:<br>[weai_own_agri_m] = '1'	12a) Do you own any of the agricultural land?	radio<br>1 YES, SOLELY<br>2 YES, JOINTLY<br>3 NO<br>Custom alignment: LV
1334	weai_own_livestock_m	13) Does anyone in your household currently have any large livestock (oxen, cattle)?	radio<br>1 Yes<br>2 No<br>Custom alignment: LV
1335	weai_own_livestock_self_m<br>Show the field ONLY if:<br>[weai_own_livestock_m] = '1'	13a) Do you own any of the large livestock?	radio<br>1 YES, SOLELY<br>2 YES, JOINTLY<br>3 NO<br>Custom alignment: LV

Havelaar AH, *et al. BMJ Open* 2022; 12:e061311. doi: 10.1136/bmjopen-2022-061311

1336	weai_own_smlivestock_m	14) Does anyone in your household currently have any small livestock (goats, pigs, sheep)?	radio<br>1 Yes<br>2 No<br>Custom alignment: LV
1337	weai_own_smlivestock_self_m<br>Show the field ONLY if:<br>[weai_own_smlivestock_m] = '1'	14a) Do you own any of the small livestock?	radio<br>1 YES, SOLELY<br>2 YES, JOINTLY<br>3 NO<br>Custom alignment: LV
1338	weai_own_foul_m	15) Does anyone in your household currently have any Chickens, Ducks, Turkeys, Pigeons?	radio<br>1 Yes<br>2 No<br>Custom alignment: LV
1339	weai_own_foul_self_m<br>Show the field ONLY if:<br>[weai_own_foul_m] = '1'	15a) Do you own any of the Chickens, Ducks, Turkeys, Pigeons?	radio<br>1 YES, SOLELY<br>2 YES, JOINTLY<br>3 NO<br>Custom alignment: LV
1340	weai_own_fish_m	16) Does anyone in your household currently have any fish pond or fishing equipment?	radio<br>1 Yes<br>2 No<br>Custom alignment: LV
1341	weai_own_fish_self_m<br>Show the field ONLY if:<br>[weai_own_fish_m] = '1'	16a) Do you own any of the fish pond or fishing equipment?	radio<br>1 YES, SOLELY<br>2 YES, JOINTLY<br>3 NO<br>Custom alignment: LV
1342	weai_own_nmequip_m	17) Does anyone in your household currently have any farm equipment (nonmechanized: hand tools, animal-drawn plough)?	radio<br>1 Yes<br>2 No<br>Custom alignment: LV
1343	weai_own_nmequip_self_m<br>Show the field ONLY if:<br>[weai_own_nmequip_m] = '1'	17a) Do you own any of the farm equipment (nonmechanized: hand tools, animal-drawn plough)?	radio<br>1 YES, SOLELY<br>2 YES, JOINTLY<br>3 NO<br>Custom alignment: LV
1344	weai_own_mequip_m	18) Does anyone in your household currently have any farm equipment (mechanized: tractor-plough, power tiller, treadle pump)?	radio<br>1 Yes<br>2 No<br>Custom alignment: LV
1345	weai_own_mequip_self_m<br>Show the field ONLY if:<br>[weai_own_mequip_m] = '1'	18a) Do you own any of the farm equipment (mechanized: tractor-plough, power tiller, treadle pump)?	radio<br>1 YES, SOLELY<br>2 YES, JOINTLY<br>3 NO<br>Custom alignment: LV

1346	weai_own_buisness_m	19) Does anyone in your household currently have any nonfarm business equipment (solar panels used for recharging, sewing machine, brewing equipment, fryers)?	radio<br>1 Yes<br>2 No<br>Custom alignment: LV
1347	weai_own_buisness_self_m<br>Show the field ONLY if:<br>[weai_own_buisness_m] = '1'	19a) Do you own any of the nonfarm business equipment (mechanized: tractor-plough, power tiller, treadle pump)?	radio<br>1 YES, SOLELY<br>2 YES, JOINTLY<br>3 NO<br>Custom alignment: LV
1348	weai_own_house_m	20) Does anyone in your household currently have any houses or other structures?	radio<br>1 Yes<br>2 No<br>Custom alignment: LV
1349	weai_own_house_self_m<br>Show the field ONLY if:<br>[weai_own_house_m] = '1'	20a) Do you own any of the houses or other structures?	radio<br>1 YES, SOLELY<br>2 YES, JOINTLY<br>3 NO<br>Custom alignment: LV
1350	weai_own_lgdurables_m	Section Header: *"Now I'd like to ask you about your household's access to and ownership of a number of items that could be used to generate income."*<br>1) Does anyone in your household currently have any large consumer durables (refrigerator, TV, sofa)?	radio<br>1 Yes<br>2 No<br>Custom alignment: LV
1351	weai_own_lgdurables_self_m<br>Show the field ONLY if:<br>[weai_own_lgdurables_m] = '1'	1a) Do you own any of the large consumer durables?	radio<br>1 YES, SOLELY<br>2 YES, JOINTLY<br>3 NO<br>Custom alignment: LV
1352	weai_own_smdurables_m	2) Does anyone in your household currently have any small consumer durables (radio, cookware)?	radio<br>1 Yes<br>2 No<br>Custom alignment: LV
1353	weai_own_smdurables_self_m<br>Show the field ONLY if:<br>[weai_own_smdurables_m] = '1'	2a) Do you own any of the small consumer durables?	radio<br>1 YES, SOLELY<br>2 YES, JOINTLY<br>3 NO<br>Custom alignment: LV
1354	weai_own_cell_m	3) Does anyone in your household currently have a cell phone?	radio<br>1 Yes<br>2 No<br>Custom alignment: LV
1355	weai_own_cell_self_m<br>Show the field ONLY if:<br>[weai_own_cell_m] = '1'	3a) Do you own a cell phone?	radio<br>1 YES, SOLELY<br>2 YES, JOINTLY<br>3 NO<br>Custom alignment: LV

1356	weai_own_land_m	4) Does anyone in your household currently have any other land not used for agricultural purposes (pieces/plots, residential or commercial land)?	radio<br>1 Yes<br>2 No<br>Custom alignment: LV
1357	weai_own_land_self_m<br>Show the field ONLY if:<br>[weai_own_land_m] = '1'	4a) Do you own any of the other land not used for agricultural purposes (pieces/plots, residential or commercial land)?	radio<br>1 YES, SOLELY<br>2 YES, JOINTLY<br>3 NO<br>Custom alignment: LV
1358	weai_own_transport_m	5) Does anyone in your household currently have any means of transportation (bicycle, motorcycle, car)	radio<br>1 Yes<br>2 No<br>Custom alignment: LV
1359	weai_own_transport_self_m<br>Show the field ONLY if:<br>[weai_own_transport_m] = '1'	5a) Do you own any of the means of transportation (bicycle, motorcycle, car)?	radio<br>1 YES, SOLELY<br>2 YES, JOINTLY<br>3 NO<br>Custom alignment: LV
1360	weai_loan_ngo_m	Section Header: *"Next I'd like to ask about your household's experience with borrowing money or other items in the past 12 months."*<br>1) Would you or anyone in your household be able to take a loan or borrow cash/in-kind from a Non-governmental organization (NGO) if you wanted to?	radio<br>1 Yes<br>2 No<br>3 Maybe<br>Custom alignment: LV
1361	weai_loan_ngo_year_m<br>Show the field ONLY if:<br>[weai_loan_ngo_m] = '1' or [weai_loan_ngo_m] = '3'	1a) Has anyone in your household taken any loans or borrowed cash/in-kind from a Non-governmental organization (NGO) in the past 12 months?	radio<br>1 YES, CASH<br>2 YES, IN-KIND<br>3 YES, CASH AND IN-KIND<br>4 NO<br>97 DON'T KNOW<br>Custom alignment: LV
1362	weai_loan_ngo_decision_m<br>Show the field ONLY if:<br>[weai_loan_ngo_year_m] = '1' or [weai_loan_ngo_year_m] = '2' or [weai_loan_ngo_year_m] = '3'	1b) Who made the decision to borrow from Non- governmental organization (NGO) most of the time?<br>*Check all that apply*	checkbox<br>1 weai_loan_ngo_decision_m___1 SELF<br>2 weai_loan_ngo_decision_m___2 SPOUSE<br>3 weai_loan_ngo_decision_m___3 OTHER HH MEMBER<br>4 weai_loan_ngo_decision_m___4 OTHER NON-HH MEMBER<br>98 weai_loan_ngo_decision_m___98 NOT APPLICABLE<br>Custom alignment: LV

1363	weai_loan_ngo_income_m<br>Show the field ONLY if:<br>[weai_loan_ngo_year_m] = '1' or [weai_loan_ngo_year_m] = '2' or [weai_loan_ngo_year_m] = '3'	1c) Who makes the decision about what to do with the money/ item borrowed from Non- governmental organization (NGO) most of the time?<br>*Check all that apply*	checkbox<br>1 weai_loan_ngo_income_m___1 SELF<br>2 weai_loan_ngo_income_m___2 SPOUSE<br>3 weai_loan_ngo_income_m___3 OTHER HH MEMBER<br>4 weai_loan_ngo_income_m___4 OTHER NON-HH MEMBER<br>98 weai_loan_ngo_income_m___98 NOT APPLICABLE<br>Custom alignment: LV
1364	weai_loan_fmlender_m	2) Would you or anyone in your household be able to take a loan or borrow cash/in-kind from a Formal lender (bank/financial institution) if you wanted to?	radio<br>1 Yes<br>2 No<br>3 Maybe<br>Custom alignment: LV
1365	weai_loan_fmlender_year_m<br>Show the field ONLY if:<br>[weai_loan_fmlender_m] = '1' or [weai_loan_fmlender_m] = '3'	2a) Has anyone in your household taken any loans or borrowed cash/in-kind from a formal lender in the past 12 months?	radio<br>1 YES, CASH<br>2 YES, IN-KIND<br>3 YES, CASH AND IN-KIND<br>4 NO<br>97 DON'T KNOW<br>Custom alignment: LV
1366	weai_loan_fmlender_decision_m<br>Show the field ONLY if:<br>[weai_loan_fmlender_year_m] = '1' or [weai_loan_fmlender_year_m] = '2' or [weai_loan_fmlender_year_m] = '3'	2b) Who made the decision to borrow from a formal lender most of the time?<br>*Check all that apply*	checkbox<br>1 weai_loan_fmlender_decision_m___1 SELF<br>2 weai_loan_fmlender_decision_m___2 SPOUSE<br>3 weai_loan_fmlender_decision_m___3 OTHER HH MEMBER<br>4 weai_loan_fmlender_decision_m___4 OTHER NON-HH MEMBER<br>98 weai_loan_fmlender_decision_m___98 NOT APPLICABLE<br>Custom alignment: LV
1367	weai_loan_fmlender_income_m<br>Show the field ONLY if:<br>[weai_loan_fmlender_year_m] = '1' or [weai_loan_fmlender_year_m] = '2' or [weai_loan_fmlender_year_m] = '3'	2c) Who makes the decision about what to do with the money/ item borrowed from a formal lender most of the time?<br>*Check all that apply*	checkbox<br>1 weai_loan_fmlender_income_m___1 SELF<br>2 weai_loan_fmlender_income_m___2 SPOUSE<br>3 weai_loan_fmlender_income_m___3 OTHER HH MEMBER<br>4 weai_loan_fmlender_income_m___4 OTHER NON-HH MEMBER<br>98 weai_loan_fmlender_income_m___98 NOT APPLICABLE<br>Custom alignment: LV
1368	weai_loan_inflender_m	3) Would you or anyone in your household be able to take a loan or borrow cash/in-kind from an informal lender if you wanted to?	radio<br>1 Yes<br>2 No<br>3 Maybe<br>Custom alignment: LV

Havelaar AH, *et al. BMJ Open* 2022; 12:e061311. doi: 10.1136/bmjopen-2022-061311

1369	weai_loan_inflender_year_m<br><br>Show the field ONLY if: [weai_loan_inflender_m] = '1' or [weai_loan_inflender_m] = '3'	3a) Has anyone in your household taken any loans or borrowed cash/in-kind from an informal lender in the past 12 months?	radio<br>1 YES, CASH<br>2 YES, IN-KIND<br>3 YES, CASH AND IN-KIND<br>4 NO<br>97 DON'T KNOW<br><br>Custom alignment: LV
1370	weai_loan_inflender_decision_m<br><br>Show the field ONLY if: [weai_loan_inflender_year_m] = '1' or [weai_loan_inflender_year_m] = '2' or [weai_loan_inflender_year_m] = '3'	3b) Who made the decision to borrow from an informal lender most of the time?<br>*Check all that apply*	checkbox<br>1 weai_loan_inflender_decision_m___1 SELF<br>2 weai_loan_inflender_decision_m___2 SPOUSE<br>3 weai_loan_inflender_decision_m___3 OTHER HH MEMBER<br>4 weai_loan_inflender_decision_m___4 OTHER NON-HH MEMBER<br>98 weai_loan_inflender_decision_m___98 NOT APPLICABLE<br><br>Custom alignment: LV
1371	weai_loan_inflender_income_m<br><br>Show the field ONLY if: [weai_loan_inflender_year_m] = '1' or [weai_loan_inflender_year_m] = '2' or [weai_loan_inflender_year_m] = '3'	3c) Who makes the decision about what to do with the money/ item borrowed from an informal lender most of the time?<br>*Check all that apply*	checkbox<br>1 weai_loan_inflender_income_m___1 SELF<br>2 weai_loan_inflender_income_m___2 SPOUSE<br>3 weai_loan_inflender_income_m___3 OTHER HH MEMBER<br>4 weai_loan_inflender_income_m___4 OTHER NON-HH MEMBER<br>98 weai_loan_inflender_income_m___98 NOT APPLICABLE<br><br>Custom alignment: LV
1372	weai_loan_family_m	4) Would you or anyone in your household be able to take a loan or borrow cash/in-kind from friends or relatives if you wanted to?	radio<br>1 Yes<br>2 No<br>3 Maybe<br><br>Custom alignment: LV
1373	weai_loan_family_year_m<br><br>Show the field ONLY if: [weai_loan_family_m] = '1' or [weai_loan_family_m] = '3'	4a) Has anyone in your household taken any loans or borrowed cash/in-kind from friends or relatives in the past 12 months?	radio<br>1 YES, CASH<br>2 YES, IN-KIND<br>3 YES, CASH AND IN-KIND<br>4 NO<br>97 DON'T KNOW<br><br>Custom alignment: LV
1374	weai_loan_family_decision_m<br><br>Show the field ONLY if: [weai_loan_family_year_m] = '1' or [weai_loan_family_year_m] = '2' or [weai_loan_family_year_m] = '3'	4b) Who made the decision to borrow from friends or relatives most of the time?<br>*Check all that apply*	checkbox<br>1 weai_loan_family_decision_m___1 SELF<br>2 weai_loan_family_decision_m___2 SPOUSE<br>3 weai_loan_family_decision_m___3 OTHER HH MEMBER<br>4 weai_loan_family_decision_m___4 OTHER NON-HH MEMBER<br>98 weai_loan_family_decision_m___98 NOT APPLICABLE<br><br>Custom alignment: LV

1375	weai_loan_family_income_m<br><br>Show the field ONLY if:<br>[weai_loan_family_year_m] = '1' or [weai_loan_family_year_m] = '2' or [weai_loan_family_year_m] = '3'	4c) Who makes the decision about what to do with the money/item borrowed from friends or relatives most of the time?<br>*Check all that apply*	checkbox<br>1 weai_loan_family_income_m___1 SELF<br>2 weai_loan_family_income_m___2 SPOUSE<br>3 weai_loan_family_income_m___3 OTHER HH MEMBER<br>4 weai_loan_family_income_m___4 OTHER NON-HH MEMBER<br>98 weai_loan_family_income_m___98 NOT APPLICABLE<br><br>Custom alignment: LV
1376	weai_loan_micro_m	5) Would you or anyone in your household be able to take a loan or borrow cash/in-kind from group based microfinance or lending including VSLAs / SACCOs if you wanted to?	radio<br>1 Yes<br>2 No<br>3 Maybe<br><br>Custom alignment: LV
1377	weai_loan_micro_year_m<br><br>Show the field ONLY if:<br>[weai_loan_micro_m] = '1' or [weai_loan_micro_m] = '3'	5a) Has anyone in your household taken any loans or borrowed cash/in-kind from Group based microfinance or lending including VSLAs / SACCOs in the past 12 months?	radio<br>1 YES, CASH<br>2 YES, IN-KIND<br>3 YES, CASH AND IN-KIND<br>4 NO<br>97 DON'T KNOW<br><br>Custom alignment: LV
1378	weai_loan_micro_decision_m<br><br>Show the field ONLY if:<br>[weai_loan_micro_year_m] = '1' or [weai_loan_micro_year_m] = '2' or [weai_loan_micro_year_m] = '3'	5b) Who made the decision to borrow from Group based microfinance or lending including VSLAs / SACCOs most of the time?<br>*Check all that apply*	checkbox<br>1 weai_loan_micro_decision_m___1 SELF<br>2 weai_loan_micro_decision_m___2 SPOUSE<br>3 weai_loan_micro_decision_m___3 OTHER HH MEMBER<br>4 weai_loan_micro_decision_m___4 OTHER NON-HH MEMBER<br>98 weai_loan_micro_decision_m___98 NOT APPLICABLE<br><br>Custom alignment: LV
1379	weai_loan_micro_income_m<br><br>Show the field ONLY if:<br>[weai_loan_micro_year_m] = '1' or [weai_loan_micro_year_m] = '2' or [weai_loan_micro_year_m] = '3'	5c) Who makes the decision about what to do with the money/item borrowed from Group based microfinance or lending including VSLAs / SACCOs most of the time?<br>*Check all that apply*	checkbox<br>1 weai_loan_micro_income_m___1 SELF<br>2 weai_loan_micro_income_m___2 SPOUSE<br>3 weai_loan_micro_income_m___3 OTHER HH MEMBER<br>4 weai_loan_micro_income_m___4 OTHER NON-HH MEMBER<br>98 weai_loan_micro_income_m___98 NOT APPLICABLE<br><br>Custom alignment: LV
1380	weai_loan_credit_m	6) Would you or anyone in your household be able to take a loan or borrow cash/in-kind from informal credit/savings groups such as merry-go-rounds, tontines, funeral societies, etc. if you wanted to?	radio<br>1 Yes<br>2 No<br>3 Maybe<br><br>Custom alignment: LV

1381	weai_loan_credit_year_m<br>Show the field ONLY if: [weai_loan_credit_m] = '1' or [weai_loan_credit_m] = '3'	6a) Has anyone in your household taken any loans or borrowed cash/in-kind from informal credit/savings groups such as merry-go- rounds, tontines, funeral societies, etc. in the past 12 months?	radio<br>1 YES, CASH<br>2 YES, IN-KIND<br>3 YES, CASH AND IN-KIND<br>4 NO<br>97 DON'T KNOW<br>Custom alignment: LV
1382	weai_loan_credit_decision_m<br>Show the field ONLY if: [weai_loan_credit_year_m] = '1' or [weai_loan_credit_year_m] = '2' or [weai_loan_credit_year_m] = '3'	6b) Who made the decision to borrow from informal credit/savings groups such as merry-go-rounds, tontines, funeral societies, etc. most of the time?<br>*Check all that apply*	checkbox<br>1 weai_loan_credit_decision_m___1 SELF<br>2 weai_loan_credit_decision_m___2 SPOUSE<br>3 weai_loan_credit_decision_m___3 OTHER HH MEMBER<br>4 weai_loan_credit_decision_m___4 OTHER NON-HH MEMBER<br>98 weai_loan_credit_decision_m___98 NOT APPLICABLE<br>Custom alignment: LV
1383	weai_loan_credit_income_m<br>Show the field ONLY if: [weai_loan_credit_year_m] = '1' or [weai_loan_credit_year_m] = '2' or [weai_loan_credit_year_m] = '3'	6c) Who makes the decision about what to do with the money/ item borrowed from informal credit/savings groups such as merry-go-rounds, tontines, funeral societies, etc.most of the time?<br>*Check all that apply*	checkbox<br>1 weai_loan_credit_income_m___1 SELF<br>2 weai_loan_credit_income_m___2 SPOUSE<br>3 weai_loan_credit_income_m___3 OTHER HH MEMBER<br>4 weai_loan_credit_income_m___4 OTHER NON-HH MEMBER<br>98 weai_loan_credit_income_m___98 NOT APPLICABLE<br>Custom alignment: LV
1384	weai_time_m	Section Header: *PLEASE RECORD A LOG OF THE ACTIVITIES FOR THE INDIVIDUAL IN THE LAST COMPLETE 24 HOURS (STARTING YESTERDAY MORNING AT 4 AM, FINISHING 3:59 AM OF THE CURRENT DAY). THE TIME INTERVALS ARE MARKED IN 15 MIN INTERVALS AND ONE ACTIVITY CAN BE MARKED FOR EACH TIME PERIOD BY DRAWING A LINE THROUGH THAT ACTIVITY.*<br>1) "Now I'd like to ask you about how you spent your time during the past 24 hours. We'll begin from yesterday morning, and continue through to this morning. This will be a detailed accounting. I'm interested in everything you do (i.e. resting, eating, personal care, work inside and outside the home, caring for children, cooking, shopping, socializing, etc.), even if it doesn't take you much time."<br>Note: Please check all that apply for all the forms.	descriptive
1385	weai_time_1_sleep_m	Section Header: *Night/Early Morning*<br>Sleeping and resting	checkbox<br>1 weai_time_1_sleep_m___1 4:00-4:15<br>2 weai_time_1_sleep_m___2 4:15-4:30<br>3 weai_time_1_sleep_m___3 4:30-4:45<br>4 weai_time_1_sleep_m___4 4:45-5:00<br>5 weai_time_1_sleep_m___5 5:00-5:15<br>6 weai_time_1_sleep_m___6 5:15-5:30<br>7 weai_time_1_sleep_m___7 5:30-5:45<br>8 weai_time_1_sleep_m___8 5:45-6:00

1386	weai_time_1_eat_m	Eating and drinking	checkbox<br>1 weai_time_1_eat_m___1 4:00-4:15<br>2 weai_time_1_eat_m___2 4:15-4:30<br>3 weai_time_1_eat_m___3 4:30-4:45<br>4 weai_time_1_eat_m___4 4:45-5:00<br>5 weai_time_1_eat_m___5 5:00-5:15<br>6 weai_time_1_eat_m___6 5:15-5:30<br>7 weai_time_1_eat_m___7 5:30-5:45<br>8 weai_time_1_eat_m___8 5:45-6:00
1387	weai_time_1_personal_m	Personal Care	checkbox<br>1 weai_time_1_personal_m___1 4:00-4:15<br>2 weai_time_1_personal_m___2 4:15-4:30<br>3 weai_time_1_personal_m___3 4:30-4:45<br>4 weai_time_1_personal_m___4 4:45-5:00<br>5 weai_time_1_personal_m___5 5:00-5:15<br>6 weai_time_1_personal_m___6 5:15-5:30<br>7 weai_time_1_personal_m___7 5:30-5:45<br>8 weai_time_1_personal_m___8 5:45-6:00
1388	weai_time_1_school_m	School (also homework)	checkbox<br>1 weai_time_1_school_m___1 4:00-4:15<br>2 weai_time_1_school_m___2 4:15-4:30<br>3 weai_time_1_school_m___3 4:30-4:45<br>4 weai_time_1_school_m___4 4:45-5:00<br>5 weai_time_1_school_m___5 5:00-5:15<br>6 weai_time_1_school_m___6 5:15-5:30<br>7 weai_time_1_school_m___7 5:30-5:45<br>8 weai_time_1_school_m___8 5:45-6:00
1389	weai_time_1_work_m	Work as employed	checkbox<br>1 weai_time_1_work_m___1 4:00-4:15<br>2 weai_time_1_work_m___2 4:15-4:30<br>3 weai_time_1_work_m___3 4:30-4:45<br>4 weai_time_1_work_m___4 4:45-5:00<br>5 weai_time_1_work_m___5 5:00-5:15<br>6 weai_time_1_work_m___6 5:15-5:30<br>7 weai_time_1_work_m___7 5:30-5:45<br>8 weai_time_1_work_m___8 5:45-6:00
1390	weai_time_1_buisness_m	Own business work	checkbox<br>1 weai_time_1_buisness_m___1 4:00-4:15<br>2 weai_time_1_buisness_m___2 4:15-4:30<br>3 weai_time_1_buisness_m___3 4:30-4:45<br>4 weai_time_1_buisness_m___4 4:45-5:00<br>5 weai_time_1_buisness_m___5 5:00-5:15<br>6 weai_time_1_buisness_m___6 5:15-5:30<br>7 weai_time_1_buisness_m___7 5:30-5:45<br>8 weai_time_1_buisness_m___8 5:45-6:00

Supplemental material BMJ Publishing Group Limited (BMJ) disclaims all liability and responsibility arising from any reliance placed on this supplemental material which has been supplied by the author(s) BMJ Open

1391	weai_time_1_farm_m	Farming/livestock/fishing	checkbox<br>1 weai_time_1_farm_m___1 4:00-4:15<br>2 weai_time_1_farm_m___2 4:15-4:30<br>3 weai_time_1_farm_m___3 4:30-4:45<br>4 weai_time_1_farm_m___4 4:45-5:00<br>5 weai_time_1_farm_m___5 5:00-5:15<br>6 weai_time_1_farm_m___6 5:15-5:30<br>7 weai_time_1_farm_m___7 5:30-5:45<br>8 weai_time_1_farm_m___8 5:45-6:00
1392	weai_time_1_shop_m	Shopping/getting service (incl health services)	checkbox<br>1 weai_time_1_shop_m___1 4:00-4:15<br>2 weai_time_1_shop_m___2 4:15-4:30<br>3 weai_time_1_shop_m___3 4:30-4:45<br>4 weai_time_1_shop_m___4 4:45-5:00<br>5 weai_time_1_shop_m___5 5:00-5:15<br>6 weai_time_1_shop_m___6 5:15-5:30<br>7 weai_time_1_shop_m___7 5:30-5:45<br>8 weai_time_1_shop_m___8 5:45-6:00
1393	weai_time_1_weave_m	Weaving, sewing, textile care	checkbox<br>1 weai_time_1_weave_m___1 4:00-4:15<br>2 weai_time_1_weave_m___2 4:15-4:30<br>3 weai_time_1_weave_m___3 4:30-4:45<br>4 weai_time_1_weave_m___4 4:45-5:00<br>5 weai_time_1_weave_m___5 5:00-5:15<br>6 weai_time_1_weave_m___6 5:15-5:30<br>7 weai_time_1_weave_m___7 5:30-5:45<br>8 weai_time_1_weave_m___8 5:45-6:00
1394	weai_time_1_cook_m	Cooking	checkbox<br>1 weai_time_1_cook_m___1 4:00-4:15<br>2 weai_time_1_cook_m___2 4:15-4:30<br>3 weai_time_1_cook_m___3 4:30-4:45<br>4 weai_time_1_cook_m___4 4:45-5:00<br>5 weai_time_1_cook_m___5 5:00-5:15<br>6 weai_time_1_cook_m___6 5:15-5:30<br>7 weai_time_1_cook_m___7 5:30-5:45<br>8 weai_time_1_cook_m___8 5:45-6:00
1395	weai_time_1_domestic_m	Domestic work (incl fetching wood and water)	checkbox<br>1 weai_time_1_domestic_m___1 4:00-4:15<br>2 weai_time_1_domestic_m___2 4:15-4:30<br>3 weai_time_1_domestic_m___3 4:30-4:45<br>4 weai_time_1_domestic_m___4 4:45-5:00<br>5 weai_time_1_domestic_m___5 5:00-5:15<br>6 weai_time_1_domestic_m___6 5:15-5:30<br>7 weai_time_1_domestic_m___7 5:30-5:45<br>8 weai_time_1_domestic_m___8 5:45-6:00

Havelaar AH, *et al. BMJ Open* 2022; 12:e061311. doi: 10.1136/bmjopen-2022-061311

1396	weai_time_1_care_m	Care for children/adults/elderly	checkbox<br>1 weai_time_1_care_m___1 4:00-4:15<br>2 weai_time_1_care_m___2 4:15-4:30<br>3 weai_time_1_care_m___3 4:30-4:45<br>4 weai_time_1_care_m___4 4:45-5:00<br>5 weai_time_1_care_m___5 5:00-5:15<br>6 weai_time_1_care_m___6 5:15-5:30<br>7 weai_time_1_care_m___7 5:30-5:45<br>8 weai_time_1_care_m___8 5:45-6:00
1397	weai_time_1_travel_m	Travelling and communiting	checkbox<br>1 weai_time_1_travel_m___1 4:00-4:15<br>2 weai_time_1_travel_m___2 4:15-4:30<br>3 weai_time_1_travel_m___3 4:30-4:45<br>4 weai_time_1_travel_m___4 4:45-5:00<br>5 weai_time_1_travel_m___5 5:00-5:15<br>6 weai_time_1_travel_m___6 5:15-5:30<br>7 weai_time_1_travel_m___7 5:30-5:45<br>8 weai_time_1_travel_m___8 5:45-6:00
1398	weai_time_1_tv_m	Watching TV/listening to radio/reading	checkbox<br>1 weai_time_1_tv_m___1 4:00-4:15<br>2 weai_time_1_tv_m___2 4:15-4:30<br>3 weai_time_1_tv_m___3 4:30-4:45<br>4 weai_time_1_tv_m___4 4:45-5:00<br>5 weai_time_1_tv_m___5 5:00-5:15<br>6 weai_time_1_tv_m___6 5:15-5:30<br>7 weai_time_1_tv_m___7 5:30-5:45<br>8 weai_time_1_tv_m___8 5:45-6:00
1399	weai_time_1_exercising_m	Exercising	checkbox<br>1 weai_time_1_exercising_m___1 4:00-4:15<br>2 weai_time_1_exercising_m___2 4:15-4:30<br>3 weai_time_1_exercising_m___3 4:30-4:45<br>4 weai_time_1_exercising_m___4 4:45-5:00<br>5 weai_time_1_exercising_m___5 5:00-5:15<br>6 weai_time_1_exercising_m___6 5:15-5:30<br>7 weai_time_1_exercising_m___7 5:30-5:45<br>8 weai_time_1_exercising_m___8 5:45-6:00
1400	weai_time_1_social_m	Social activities and hobbies	checkbox<br>1 weai_time_1_social_m___1 4:00-4:15<br>2 weai_time_1_social_m___2 4:15-4:30<br>3 weai_time_1_social_m___3 4:30-4:45<br>4 weai_time_1_social_m___4 4:45-5:00<br>5 weai_time_1_social_m___5 5:00-5:15<br>6 weai_time_1_social_m___6 5:15-5:30<br>7 weai_time_1_social_m___7 5:30-5:45<br>8 weai_time_1_social_m___8 5:45-6:00

Havelaar AH, *et al. BMJ Open* 2022; 12:e061311. doi: 10.1136/bmjopen-2022-061311

1401	weai_time_1_religious_m	Religious activities	checkbox<br>1 weai_time_1_religious_m___1 4:00-4:15<br>2 weai_time_1_religious_m___2 4:15-4:30<br>3 weai_time_1_religious_m___3 4:30-4:45<br>4 weai_time_1_religious_m___4 4:45-5:00<br>5 weai_time_1_religious_m___5 5:00-5:15<br>6 weai_time_1_religious_m___6 5:15-5:30<br>7 weai_time_1_religious_m___7 5:30-5:45<br>8 weai_time_1_religious_m___8 5:45-6:00
1402	weai_time_1_other_m	Other (specify below)	checkbox<br>1 weai_time_1_other_m___1 4:00-4:15<br>2 weai_time_1_other_m___2 4:15-4:30<br>3 weai_time_1_other_m___3 4:30-4:45<br>4 weai_time_1_other_m___4 4:45-5:00<br>5 weai_time_1_other_m___5 5:00-5:15<br>6 weai_time_1_other_m___6 5:15-5:30<br>7 weai_time_1_other_m___7 5:30-5:45<br>8 weai_time_1_other_m___8 5:45-6:00
1403	weai_time_1_other_specify_m<br>Show the field ONLY if: [weai_time_1_other_m(1)] or [weai_time_1_other_m(2)] or [weai_time_1_other_m(3)] or [weai_time_1_other_m(4)] or [weai_time_1_other_m(5)] or [weai_time_1_other_m(6)] or [weai_time_1_other_m(7)] or [weai_time_1_other_m(8)]	Specify Other	text<br>Custom alignment: LV
1404	weai_time_2_sleep_m	Section Header: *Morning*<br>Sleeping and resting	checkbox<br>1 weai_time_2_sleep_m___1 6:00-6:15<br>2 weai_time_2_sleep_m___2 6:15-6:30<br>3 weai_time_2_sleep_m___3 6:30-6:45<br>4 weai_time_2_sleep_m___4 6:45-7:00<br>5 weai_time_2_sleep_m___5 7:00-7:15<br>6 weai_time_2_sleep_m___6 7:15-7:30<br>7 weai_time_2_sleep_m___7 7:30-7:45<br>8 weai_time_2_sleep_m___8 7:45-8:00
1405	weai_time_2_eat_m	Eating and drinking	checkbox<br>1 weai_time_2_eat_m___1 6:00-6:15<br>2 weai_time_2_eat_m___2 6:15-6:30<br>3 weai_time_2_eat_m___3 6:30-6:45<br>4 weai_time_2_eat_m___4 6:45-7:00<br>5 weai_time_2_eat_m___5 7:00-7:15<br>6 weai_time_2_eat_m___6 7:15-7:30<br>7 weai_time_2_eat_m___7 7:30-7:45<br>8 weai_time_2_eat_m___8 7:45-8:00

Havelaar AH, *et al. BMJ Open* 2022; 12:e061311. doi: 10.1136/bmjopen-2022-061311

Supplemental material BMJ Publishing Group Limited (BMJ) disclaims all liability and responsibility arising from any reliance placed on this supplemental material which has been supplied by the author(s) BMJ Open

1406	weai_time_2_personal_m	Personal Care	checkbox<br>1 weai_time_2_personal_m___1 6:00-6:15<br>2 weai_time_2_personal_m___2 6:15-6:30<br>3 weai_time_2_personal_m___3 6:30-6:45<br>4 weai_time_2_personal_m___4 6:45-7:00<br>5 weai_time_2_personal_m___5 7:00-7:15<br>6 weai_time_2_personal_m___6 7:15-7:30<br>7 weai_time_2_personal_m___7 7:30-7:45<br>8 weai_time_2_personal_m___8 7:45-8:00
1407	weai_time_2_school_m	School (also homework)	checkbox<br>1 weai_time_2_school_m___1 6:00-6:15<br>2 weai_time_2_school_m___2 6:15-6:30<br>3 weai_time_2_school_m___3 6:30-6:45<br>4 weai_time_2_school_m___4 6:45-7:00<br>5 weai_time_2_school_m___5 7:00-7:15<br>6 weai_time_2_school_m___6 7:15-7:30<br>7 weai_time_2_school_m___7 7:30-7:45<br>8 weai_time_2_school_m___8 7:45-8:00
1408	weai_time_2_work_m	Work as employed	checkbox<br>1 weai_time_2_work_m___1 6:00-6:15<br>2 weai_time_2_work_m___2 6:15-6:30<br>3 weai_time_2_work_m___3 6:30-6:45<br>4 weai_time_2_work_m___4 6:45-7:00<br>5 weai_time_2_work_m___5 7:00-7:15<br>6 weai_time_2_work_m___6 7:15-7:30<br>7 weai_time_2_work_m___7 7:30-7:45<br>8 weai_time_2_work_m___8 7:45-8:00
1409	weai_time_2_buisness_m	Own business work	checkbox<br>1 weai_time_2_buisness_m___1 6:00-6:15<br>2 weai_time_2_buisness_m___2 6:15-6:30<br>3 weai_time_2_buisness_m___3 6:30-6:45<br>4 weai_time_2_buisness_m___4 6:45-7:00<br>5 weai_time_2_buisness_m___5 7:00-7:15<br>6 weai_time_2_buisness_m___6 7:15-7:30<br>7 weai_time_2_buisness_m___7 7:30-7:45<br>8 weai_time_2_buisness_m___8 7:45-8:00
1410	weai_time_2_farm_m	Farming/livestock/fishing	checkbox<br>1 weai_time_2_farm_m___1 6:00-6:15<br>2 weai_time_2_farm_m___2 6:15-6:30<br>3 weai_time_2_farm_m___3 6:30-6:45<br>4 weai_time_2_farm_m___4 6:45-7:00<br>5 weai_time_2_farm_m___5 7:00-7:15<br>6 weai_time_2_farm_m___6 7:15-7:30<br>7 weai_time_2_farm_m___7 7:30-7:45<br>8 weai_time_2_farm_m___8 7:45-8:00

Havelaar AH, *et al. BMJ Open* 2022; 12:e061311. doi: 10.1136/bmjopen-2022-061311

1411	weai_time_2_shop_m	Shopping/getting service (incl health services)	checkbox<br>1 weai_time_2_shop_m___1 6:00-6:15<br>2 weai_time_2_shop_m___2 6:15-6:30<br>3 weai_time_2_shop_m___3 6:30-6:45<br>4 weai_time_2_shop_m___4 6:45-7:00<br>5 weai_time_2_shop_m___5 7:00-7:15<br>6 weai_time_2_shop_m___6 7:15-7:30<br>7 weai_time_2_shop_m___7 7:30-7:45<br>8 weai_time_2_shop_m___8 7:45-8:00
1412	weai_time_2_weave_m	Weaving, sewing, textile care	checkbox<br>1 weai_time_2_weave_m___1 6:00-6:15<br>2 weai_time_2_weave_m___2 6:15-6:30<br>3 weai_time_2_weave_m___3 6:30-6:45<br>4 weai_time_2_weave_m___4 6:45-7:00<br>5 weai_time_2_weave_m___5 7:00-7:15<br>6 weai_time_2_weave_m___6 7:15-7:30<br>7 weai_time_2_weave_m___7 7:30-7:45<br>8 weai_time_2_weave_m___8 7:45-8:00
1413	weai_time_2_cook_m	Cooking	checkbox<br>1 weai_time_2_cook_m___1 6:00-6:15<br>2 weai_time_2_cook_m___2 6:15-6:30<br>3 weai_time_2_cook_m___3 6:30-6:45<br>4 weai_time_2_cook_m___4 6:45-7:00<br>5 weai_time_2_cook_m___5 7:00-7:15<br>6 weai_time_2_cook_m___6 7:15-7:30<br>7 weai_time_2_cook_m___7 7:30-7:45<br>8 weai_time_2_cook_m___8 7:45-8:00
1414	weai_time_2_domestic_m	Domestic work (incl fetching wood and water)	checkbox<br>1 weai_time_2_domestic_m___1 6:00-6:15<br>2 weai_time_2_domestic_m___2 6:15-6:30<br>3 weai_time_2_domestic_m___3 6:30-6:45<br>4 weai_time_2_domestic_m___4 6:45-7:00<br>5 weai_time_2_domestic_m___5 7:00-7:15<br>6 weai_time_2_domestic_m___6 7:15-7:30<br>7 weai_time_2_domestic_m___7 7:30-7:45<br>8 weai_time_2_domestic_m___8 7:45-8:00
1415	weai_time_2_care_m	Care for children/adults/elderly	checkbox<br>1 weai_time_2_care_m___1 6:00-6:15<br>2 weai_time_2_care_m___2 6:15-6:30<br>3 weai_time_2_care_m___3 6:30-6:45<br>4 weai_time_2_care_m___4 6:45-7:00<br>5 weai_time_2_care_m___5 7:00-7:15<br>6 weai_time_2_care_m___6 7:15-7:30<br>7 weai_time_2_care_m___7 7:30-7:45<br>8 weai_time_2_care_m___8 7:45-8:00

Havelaar AH, *et al. BMJ Open* 2022; 12:e061311. doi: 10.1136/bmjopen-2022-061311

1416	weai_time_2_travel_m	Travelling and communiting	checkbox<br>1 weai_time_2_travel_m___1 6:00-6:15<br>2 weai_time_2_travel_m___2 6:15-6:30<br>3 weai_time_2_travel_m___3 6:30-6:45<br>4 weai_time_2_travel_m___4 6:45-7:00<br>5 weai_time_2_travel_m___5 7:00-7:15<br>6 weai_time_2_travel_m___6 7:15-7:30<br>7 weai_time_2_travel_m___7 7:30-7:45<br>8 weai_time_2_travel_m___8 7:45-8:00
1417	weai_time_2_tv_m	Watching TV/listening to radio/reading	checkbox<br>1 weai_time_2_tv_m___1 6:00-6:15<br>2 weai_time_2_tv_m___2 6:15-6:30<br>3 weai_time_2_tv_m___3 6:30-6:45<br>4 weai_time_2_tv_m___4 6:45-7:00<br>5 weai_time_2_tv_m___5 7:00-7:15<br>6 weai_time_2_tv_m___6 7:15-7:30<br>7 weai_time_2_tv_m___7 7:30-7:45<br>8 weai_time_2_tv_m___8 7:45-8:00
1418	weai_time_2_exercising_m	Exercising	checkbox<br>1 weai_time_2_exercising_m___1 6:00-6:15<br>2 weai_time_2_exercising_m___2 6:15-6:30<br>3 weai_time_2_exercising_m___3 6:30-6:45<br>4 weai_time_2_exercising_m___4 6:45-7:00<br>5 weai_time_2_exercising_m___5 7:00-7:15<br>6 weai_time_2_exercising_m___6 7:15-7:30<br>7 weai_time_2_exercising_m___7 7:30-7:45<br>8 weai_time_2_exercising_m___8 7:45-8:00
1419	weai_time_2_social_m	Social activities and hobbies	checkbox<br>1 weai_time_2_social_m___1 6:00-6:15<br>2 weai_time_2_social_m___2 6:15-6:30<br>3 weai_time_2_social_m___3 6:30-6:45<br>4 weai_time_2_social_m___4 6:45-7:00<br>5 weai_time_2_social_m___5 7:00-7:15<br>6 weai_time_2_social_m___6 7:15-7:30<br>7 weai_time_2_social_m___7 7:30-7:45<br>8 weai_time_2_social_m___8 7:45-8:00
1420	weai_time_2_religious_m	Religious activities	checkbox<br>1 weai_time_2_religious_m___1 6:00-6:15<br>2 weai_time_2_religious_m___2 6:15-6:30<br>3 weai_time_2_religious_m___3 6:30-6:45<br>4 weai_time_2_religious_m___4 6:45-7:00<br>5 weai_time_2_religious_m___5 7:00-7:15<br>6 weai_time_2_religious_m___6 7:15-7:30<br>7 weai_time_2_religious_m___7 7:30-7:45<br>8 weai_time_2_religious_m___8 7:45-8:00

1421	weai_time_2_other_m	Other (specify below)	checkbox<br>1 weai_time_2_other_m___1 6:00-6:15<br>2 weai_time_2_other_m___2 6:15-6:30<br>3 weai_time_2_other_m___3 6:30-6:45<br>4 weai_time_2_other_m___4 6:45-7:00<br>5 weai_time_2_other_m___5 7:00-7:15<br>6 weai_time_2_other_m___6 7:15-7:30<br>7 weai_time_2_other_m___7 7:30-7:45<br>8 weai_time_2_other_m___8 7:45-8:00
1422	weai_time_2_other_specify_m<br>Show the field ONLY if: [weai_time_2_other_m(1)] or [weai_time_2_other_m(2)] or [weai_time_2_other_m(3)] or [weai_time_2_other_m(4)] or [weai_time_2_other_m(5)] or [weai_time_2_other_m(6)] or [weai_time_2_other_m(7)] or [weai_time_2_other_m(8)]	Specify Other	text<br>Custom alignment: LV
1423	weai_time_3_sleep_m	Section Header: *Early Day*<br>Sleeping and resting	checkbox<br>1 weai_time_3_sleep_m___1 8:00-8:15<br>2 weai_time_3_sleep_m___2 8:15-8:30<br>3 weai_time_3_sleep_m___3 8:30-8:45<br>4 weai_time_3_sleep_m___4 8:45-9:00<br>5 weai_time_3_sleep_m___5 9:00-9:15<br>6 weai_time_3_sleep_m___6 9:15-9:30<br>7 weai_time_3_sleep_m___7 9:30-9:45<br>8 weai_time_3_sleep_m___8 9:45-10:00
1424	weai_time_3_eat_m	Eating and drinking	checkbox<br>1 weai_time_3_eat_m___1 8:00-8:15<br>2 weai_time_3_eat_m___2 8:15-8:30<br>3 weai_time_3_eat_m___3 8:30-8:45<br>4 weai_time_3_eat_m___4 8:45-9:00<br>5 weai_time_3_eat_m___5 9:00-9:15<br>6 weai_time_3_eat_m___6 9:15-9:30<br>7 weai_time_3_eat_m___7 9:30-9:45<br>8 weai_time_3_eat_m___8 9:45-10:00
1425	weai_time_3_personal_m	Personal Care	checkbox<br>1 weai_time_3_personal_m___1 8:00-8:15<br>2 weai_time_3_personal_m___2 8:15-8:30<br>3 weai_time_3_personal_m___3 8:30-8:45<br>4 weai_time_3_personal_m___4 8:45-9:00<br>5 weai_time_3_personal_m___5 9:00-9:15<br>6 weai_time_3_personal_m___6 9:15-9:30<br>7 weai_time_3_personal_m___7 9:30-9:45<br>8 weai_time_3_personal_m___8 9:45-10:00

1426	weai_time_3_school_m	School (also homework)	checkbox<br>1 weai_time_3_school_m___1 8:00-8:15<br>2 weai_time_3_school_m___2 8:15-8:30<br>3 weai_time_3_school_m___3 8:30-8:45<br>4 weai_time_3_school_m___4 8:45-9:00<br>5 weai_time_3_school_m___5 9:00-9:15<br>6 weai_time_3_school_m___6 9:15-9:30<br>7 weai_time_3_school_m___7 9:30-9:45<br>8 weai_time_3_school_m___8 9:45-10:00
1427	weai_time_3_work_m	Work as employed	checkbox<br>1 weai_time_3_work_m___1 8:00-8:15<br>2 weai_time_3_work_m___2 8:15-8:30<br>3 weai_time_3_work_m___3 8:30-8:45<br>4 weai_time_3_work_m___4 8:45-9:00<br>5 weai_time_3_work_m___5 9:00-9:15<br>6 weai_time_3_work_m___6 9:15-9:30<br>7 weai_time_3_work_m___7 9:30-9:45<br>8 weai_time_3_work_m___8 9:45-10:00
1428	weai_time_3_buisness_m	Own business work	checkbox<br>1 weai_time_3_buisness_m___1 8:00-8:15<br>2 weai_time_3_buisness_m___2 8:15-8:30<br>3 weai_time_3_buisness_m___3 8:30-8:45<br>4 weai_time_3_buisness_m___4 8:45-9:00<br>5 weai_time_3_buisness_m___5 9:00-9:15<br>6 weai_time_3_buisness_m___6 9:15-9:30<br>7 weai_time_3_buisness_m___7 9:30-9:45<br>8 weai_time_3_buisness_m___8 9:45-10:00
1429	weai_time_3_farm_m	Farming/livestock/fishing	checkbox<br>1 weai_time_3_farm_m___1 8:00-8:15<br>2 weai_time_3_farm_m___2 8:15-8:30<br>3 weai_time_3_farm_m___3 8:30-8:45<br>4 weai_time_3_farm_m___4 8:45-9:00<br>5 weai_time_3_farm_m___5 9:00-9:15<br>6 weai_time_3_farm_m___6 9:15-9:30<br>7 weai_time_3_farm_m___7 9:30-9:45<br>8 weai_time_3_farm_m___8 9:45-10:00
1430	weai_time_3_shop_m	Shopping/getting service (incl health services)	checkbox<br>1 weai_time_3_shop_m___1 8:00-8:15<br>2 weai_time_3_shop_m___2 8:15-8:30<br>3 weai_time_3_shop_m___3 8:30-8:45<br>4 weai_time_3_shop_m___4 8:45-9:00<br>5 weai_time_3_shop_m___5 9:00-9:15<br>6 weai_time_3_shop_m___6 9:15-9:30<br>7 weai_time_3_shop_m___7 9:30-9:45<br>8 weai_time_3_shop_m___8 9:45-10:00

Havelaar AH, *et al. BMJ Open* 2022; 12:e061311. doi: 10.1136/bmjopen-2022-061311

1431	weai_time_3_weave_m	Weaving, sewing, textile care	checkbox<br>1 weai_time_3_weave_m___1 8:00-8:15<br>2 weai_time_3_weave_m___2 8:15-8:30<br>3 weai_time_3_weave_m___3 8:30-8:45<br>4 weai_time_3_weave_m___4 8:45-9:00<br>5 weai_time_3_weave_m___5 9:00-9:15<br>6 weai_time_3_weave_m___6 9:15-9:30<br>7 weai_time_3_weave_m___7 9:30-9:45<br>8 weai_time_3_weave_m___8 9:45-10:00
1432	weai_time_3_cook_m	Cooking	checkbox<br>1 weai_time_3_cook_m___1 8:00-8:15<br>2 weai_time_3_cook_m___2 8:15-8:30<br>3 weai_time_3_cook_m___3 8:30-8:45<br>4 weai_time_3_cook_m___4 8:45-9:00<br>5 weai_time_3_cook_m___5 9:00-9:15<br>6 weai_time_3_cook_m___6 9:15-9:30<br>7 weai_time_3_cook_m___7 9:30-9:45<br>8 weai_time_3_cook_m___8 9:45-10:00
1433	weai_time_3_domestic_m	Domestic work (incl fetching wood and water)	checkbox<br>1 weai_time_3_domestic_m___1 8:00-8:15<br>2 weai_time_3_domestic_m___2 8:15-8:30<br>3 weai_time_3_domestic_m___3 8:30-8:45<br>4 weai_time_3_domestic_m___4 8:45-9:00<br>5 weai_time_3_domestic_m___5 9:00-9:15<br>6 weai_time_3_domestic_m___6 9:15-9:30<br>7 weai_time_3_domestic_m___7 9:30-9:45<br>8 weai_time_3_domestic_m___8 9:45-10:00
1434	weai_time_3_care_m	Care for children/adults/elderly	checkbox<br>1 weai_time_3_care_m___1 8:00-8:15<br>2 weai_time_3_care_m___2 8:15-8:30<br>3 weai_time_3_care_m___3 8:30-8:45<br>4 weai_time_3_care_m___4 8:45-9:00<br>5 weai_time_3_care_m___5 9:00-9:15<br>6 weai_time_3_care_m___6 9:15-9:30<br>7 weai_time_3_care_m___7 9:30-9:45<br>8 weai_time_3_care_m___8 9:45-10:00
1435	weai_time_3_travel_m	Travelling and communiting	checkbox<br>1 weai_time_3_travel_m___1 8:00-8:15<br>2 weai_time_3_travel_m___2 8:15-8:30<br>3 weai_time_3_travel_m___3 8:30-8:45<br>4 weai_time_3_travel_m___4 8:45-9:00<br>5 weai_time_3_travel_m___5 9:00-9:15<br>6 weai_time_3_travel_m___6 9:15-9:30<br>7 weai_time_3_travel_m___7 9:30-9:45<br>8 weai_time_3_travel_m___8 9:45-10:00

Havelaar AH, et al. BMJ Open 2022; 12:e061311. doi: 10.1136/bmjopen-2022-061311

Supplemental material BMJ Publishing Group Limited (BMJ) disclaims all liability and responsibility arising from any reliance placed on this supplemental material which has been supplied by the author(s) BMJ Open

1436	weai_time_3_tv_m	Watching TV/listening to radio/reading	checkbox<br>1 weai_time_3_tv_m___1 8:00-8:15<br>2 weai_time_3_tv_m___2 8:15-8:30<br>3 weai_time_3_tv_m___3 8:30-8:45<br>4 weai_time_3_tv_m___4 8:45-9:00<br>5 weai_time_3_tv_m___5 9:00-9:15<br>6 weai_time_3_tv_m___6 9:15-9:30<br>7 weai_time_3_tv_m___7 9:30-9:45<br>8 weai_time_3_tv_m___8 9:45-10:00
1437	weai_time_3_exercising_m	Exercising	checkbox<br>1 weai_time_3_exercising_m___1 8:00-8:15<br>2 weai_time_3_exercising_m___2 8:15-8:30<br>3 weai_time_3_exercising_m___3 8:30-8:45<br>4 weai_time_3_exercising_m___4 8:45-9:00<br>5 weai_time_3_exercising_m___5 9:00-9:15<br>6 weai_time_3_exercising_m___6 9:15-9:30<br>7 weai_time_3_exercising_m___7 9:30-9:45<br>8 weai_time_3_exercising_m___8 9:45-10:00
1438	weai_time_3_social_m	Social activities and hobbies	checkbox<br>1 weai_time_3_social_m___1 8:00-8:15<br>2 weai_time_3_social_m___2 8:15-8:30<br>3 weai_time_3_social_m___3 8:30-8:45<br>4 weai_time_3_social_m___4 8:45-9:00<br>5 weai_time_3_social_m___5 9:00-9:15<br>6 weai_time_3_social_m___6 9:15-9:30<br>7 weai_time_3_social_m___7 9:30-9:45<br>8 weai_time_3_social_m___8 9:45-10:00
1439	weai_time_3_religious_m	Religious activities	checkbox<br>1 weai_time_3_religious_m___1 8:00-8:15<br>2 weai_time_3_religious_m___2 8:15-8:30<br>3 weai_time_3_religious_m___3 8:30-8:45<br>4 weai_time_3_religious_m___4 8:45-9:00<br>5 weai_time_3_religious_m___5 9:00-9:15<br>6 weai_time_3_religious_m___6 9:15-9:30<br>7 weai_time_3_religious_m___7 9:30-9:45<br>8 weai_time_3_religious_m___8 9:45-10:00
1440	weai_time_3_other_m	Other (specify below)	checkbox<br>1 weai_time_3_other_m___1 8:00-8:15<br>2 weai_time_3_other_m___2 8:15-8:30<br>3 weai_time_3_other_m___3 8:30-8:45<br>4 weai_time_3_other_m___4 8:45-9:00<br>5 weai_time_3_other_m___5 9:00-9:15<br>6 weai_time_3_other_m___6 9:15-9:30<br>7 weai_time_3_other_m___7 9:30-9:45<br>8 weai_time_3_other_m___8 9:45-10:00

Havelaar AH, *et al. BMJ Open* 2022; 12:e061311. doi: 10.1136/bmjopen-2022-061311

1441	weai_time_3_other_specify_m<br>Show the field ONLY if:<br>[weai_time_3_other_m(1)] or<br>[weai_time_3_other_m(2)] or<br>[weai_time_3_other_m(3)] or<br>[weai_time_3_other_m(4)] or<br>[weai_time_3_other_m(5)] or<br>[weai_time_3_other_m(6)] or<br>[weai_time_3_other_m(7)] or<br>[weai_time_3_other_m(8)]	Specify Other	text<br>Custom alignment: LV
1442	weai_time_4_sleep_m	Section Header: *Late Day*<br>Sleeping and resting	checkbox<br>1 weai_time_4_sleep_m___1 10:00-10:15<br>2 weai_time_4_sleep_m___2 10:15-10:30<br>3 weai_time_4_sleep_m___3 10:30-10:45<br>4 weai_time_4_sleep_m___4 10:45-11:00<br>5 weai_time_4_sleep_m___5 11:00-11:15<br>6 weai_time_4_sleep_m___6 11:15-11:30<br>7 weai_time_4_sleep_m___7 11:30-11:45<br>8 weai_time_4_sleep_m___8 11:45-12:00
1443	weai_time_4_eat_m	Eating and drinking	checkbox<br>1 weai_time_4_eat_m___1 10:00-10:15<br>2 weai_time_4_eat_m___2 10:15-10:30<br>3 weai_time_4_eat_m___3 10:30-10:45<br>4 weai_time_4_eat_m___4 10:45-11:00<br>5 weai_time_4_eat_m___5 11:00-11:15<br>6 weai_time_4_eat_m___6 11:15-11:30<br>7 weai_time_4_eat_m___7 11:30-11:45<br>8 weai_time_4_eat_m___8 11:45-12:00
1444	weai_time_4_personal_m	Personal Care	checkbox<br>1 weai_time_4_personal_m___1 10:00-10:15<br>2 weai_time_4_personal_m___2 10:15-10:30<br>3 weai_time_4_personal_m___3 10:30-10:45<br>4 weai_time_4_personal_m___4 10:45-11:00<br>5 weai_time_4_personal_m___5 11:00-11:15<br>6 weai_time_4_personal_m___6 11:15-11:30<br>7 weai_time_4_personal_m___7 11:30-11:45<br>8 weai_time_4_personal_m___8 11:45-12:00
1445	weai_time_4_school_m	School (also homework)	checkbox<br>1 weai_time_4_school_m___1 10:00-10:15<br>2 weai_time_4_school_m___2 10:15-10:30<br>3 weai_time_4_school_m___3 10:30-10:45<br>4 weai_time_4_school_m___4 10:45-11:00<br>5 weai_time_4_school_m___5 11:00-11:15<br>6 weai_time_4_school_m___6 11:15-11:30<br>7 weai_time_4_school_m___7 11:30-11:45<br>8 weai_time_4_school_m___8 11:45-12:00

1446	weai_time_4_work_m	Work as employed	checkbox<br>1 weai_time_4_work_m___1 10:00-10:15<br>2 weai_time_4_work_m___2 10:15-10:30<br>3 weai_time_4_work_m___3 10:30-10:45<br>4 weai_time_4_work_m___4 10:45-11:00<br>5 weai_time_4_work_m___5 11:00-11:15<br>6 weai_time_4_work_m___6 11:15-11:30<br>7 weai_time_4_work_m___7 11:30-11:45<br>8 weai_time_4_work_m___8 11:45-12:00
1447	weai_time_4_buisness_m	Own business work	checkbox<br>1 weai_time_4_buisness_m___1 10:00-10:15<br>2 weai_time_4_buisness_m___2 10:15-10:30<br>3 weai_time_4_buisness_m___3 10:30-10:45<br>4 weai_time_4_buisness_m___4 10:45-11:00<br>5 weai_time_4_buisness_m___5 11:00-11:15<br>6 weai_time_4_buisness_m___6 11:15-11:30<br>7 weai_time_4_buisness_m___7 11:30-11:45<br>8 weai_time_4_buisness_m___8 11:45-12:00
1448	weai_time_4_farm_m	Farming/livestock/fishing	checkbox<br>1 weai_time_4_farm_m___1 10:00-10:15<br>2 weai_time_4_farm_m___2 10:15-10:30<br>3 weai_time_4_farm_m___3 10:30-10:45<br>4 weai_time_4_farm_m___4 10:45-11:00<br>5 weai_time_4_farm_m___5 11:00-11:15<br>6 weai_time_4_farm_m___6 11:15-11:30<br>7 weai_time_4_farm_m___7 11:30-11:45<br>8 weai_time_4_farm_m___8 11:45-12:00
1449	weai_time_4_shop_m	Shopping/getting service (incl health services)	checkbox<br>1 weai_time_4_shop_m___1 10:00-10:15<br>2 weai_time_4_shop_m___2 10:15-10:30<br>3 weai_time_4_shop_m___3 10:30-10:45<br>4 weai_time_4_shop_m___4 10:45-11:00<br>5 weai_time_4_shop_m___5 11:00-11:15<br>6 weai_time_4_shop_m___6 11:15-11:30<br>7 weai_time_4_shop_m___7 11:30-11:45<br>8 weai_time_4_shop_m___8 11:45-12:00
1450	weai_time_4_weave_m	Weaving, sewing, textile care	checkbox<br>1 weai_time_4_weave_m___1 10:00-10:15<br>2 weai_time_4_weave_m___2 10:15-10:30<br>3 weai_time_4_weave_m___3 10:30-10:45<br>4 weai_time_4_weave_m___4 10:45-11:00<br>5 weai_time_4_weave_m___5 11:00-11:15<br>6 weai_time_4_weave_m___6 11:15-11:30<br>7 weai_time_4_weave_m___7 11:30-11:45<br>8 weai_time_4_weave_m___8 11:45-12:00

Havelaar AH, *et al. BMJ Open* 2022; 12:e061311. doi: 10.1136/bmjopen-2022-061311

1451	weai_time_4_cook_m	Cooking	checkbox<br>1 weai_time_4_cook_m___1 10:00-10:15<br>2 weai_time_4_cook_m___2 10:15-10:30<br>3 weai_time_4_cook_m___3 10:30-10:45<br>4 weai_time_4_cook_m___4 10:45-11:00<br>5 weai_time_4_cook_m___5 11:00-11:15<br>6 weai_time_4_cook_m___6 11:15-11:30<br>7 weai_time_4_cook_m___7 11:30-11:45<br>8 weai_time_4_cook_m___8 11:45-12:00
1452	weai_time_4_domestic_m	Domestic work (incl fetching wood and water)	checkbox<br>1 weai_time_4_domestic_m___1 10:00-10:15<br>2 weai_time_4_domestic_m___2 10:15-10:30<br>3 weai_time_4_domestic_m___3 10:30-10:45<br>4 weai_time_4_domestic_m___4 10:45-11:00<br>5 weai_time_4_domestic_m___5 11:00-11:15<br>6 weai_time_4_domestic_m___6 11:15-11:30<br>7 weai_time_4_domestic_m___7 11:30-11:45<br>8 weai_time_4_domestic_m___8 11:45-12:00
1453	weai_time_4_care_m	Care for children/adults/elderly	checkbox<br>1 weai_time_4_care_m___1 10:00-10:15<br>2 weai_time_4_care_m___2 10:15-10:30<br>3 weai_time_4_care_m___3 10:30-10:45<br>4 weai_time_4_care_m___4 10:45-11:00<br>5 weai_time_4_care_m___5 11:00-11:15<br>6 weai_time_4_care_m___6 11:15-11:30<br>7 weai_time_4_care_m___7 11:30-11:45<br>8 weai_time_4_care_m___8 11:45-12:00
1454	weai_time_4_travel_m	Travelling and communiting	checkbox<br>1 weai_time_4_travel_m___1 10:00-10:15<br>2 weai_time_4_travel_m___2 10:15-10:30<br>3 weai_time_4_travel_m___3 10:30-10:45<br>4 weai_time_4_travel_m___4 10:45-11:00<br>5 weai_time_4_travel_m___5 11:00-11:15<br>6 weai_time_4_travel_m___6 11:15-11:30<br>7 weai_time_4_travel_m___7 11:30-11:45<br>8 weai_time_4_travel_m___8 11:45-12:00
1455	weai_time_4_tv_m	Watching TV/listening to radio/reading	checkbox<br>1 weai_time_4_tv_m___1 10:00-10:15<br>2 weai_time_4_tv_m___2 10:15-10:30<br>3 weai_time_4_tv_m___3 10:30-10:45<br>4 weai_time_4_tv_m___4 10:45-11:00<br>5 weai_time_4_tv_m___5 11:00-11:15<br>6 weai_time_4_tv_m___6 11:15-11:30<br>7 weai_time_4_tv_m___7 11:30-11:45<br>8 weai_time_4_tv_m___8 11:45-12:00

1456	weai_time_4_exercising_m	Exercising	checkbox<br>1 weai_time_4_exercising_m___1 10:00-10:15<br>2 weai_time_4_exercising_m___2 10:15-10:30<br>3 weai_time_4_exercising_m___3 10:30-10:45<br>4 weai_time_4_exercising_m___4 10:45-11:00<br>5 weai_time_4_exercising_m___5 11:00-11:15<br>6 weai_time_4_exercising_m___6 11:15-11:30<br>7 weai_time_4_exercising_m___7 11:30-11:45<br>8 weai_time_4_exercising_m___8 11:45-12:00
1457	weai_time_4_social_m	Social activities and hobbies	checkbox<br>1 weai_time_4_social_m___1 10:00-10:15<br>2 weai_time_4_social_m___2 10:15-10:30<br>3 weai_time_4_social_m___3 10:30-10:45<br>4 weai_time_4_social_m___4 10:45-11:00<br>5 weai_time_4_social_m___5 11:00-11:15<br>6 weai_time_4_social_m___6 11:15-11:30<br>7 weai_time_4_social_m___7 11:30-11:45<br>8 weai_time_4_social_m___8 11:45-12:00
1458	weai_time_4_religious_m	Religious activities	checkbox<br>1 weai_time_4_religious_m___1 10:00-10:15<br>2 weai_time_4_religious_m___2 10:15-10:30<br>3 weai_time_4_religious_m___3 10:30-10:45<br>4 weai_time_4_religious_m___4 10:45-11:00<br>5 weai_time_4_religious_m___5 11:00-11:15<br>6 weai_time_4_religious_m___6 11:15-11:30<br>7 weai_time_4_religious_m___7 11:30-11:45<br>8 weai_time_4_religious_m___8 11:45-12:00
1459	weai_time_4_other_m	Other (specify below)	checkbox<br>1 weai_time_4_other_m___1 10:00-10:15<br>2 weai_time_4_other_m___2 10:15-10:30<br>3 weai_time_4_other_m___3 10:30-10:45<br>4 weai_time_4_other_m___4 10:45-11:00<br>5 weai_time_4_other_m___5 11:00-11:15<br>6 weai_time_4_other_m___6 11:15-11:30<br>7 weai_time_4_other_m___7 11:30-11:45<br>8 weai_time_4_other_m___8 11:45-12:00
1460	weai_time_4_other_specify_m<br>Show the field ONLY if:<br>[weai_time_4_other_m(1)] or<br>[weai_time_4_other_m(2)] or<br>[weai_time_4_other_m(3)] or<br>[weai_time_4_other_m(4)] or<br>[weai_time_4_other_m(5)] or<br>[weai_time_4_other_m(6)] or<br>[weai_time_4_other_m(7)] or<br>[weai_time_4_other_m(8)]	Specify Other	text<br>Custom alignment: LV

1461	weai_time_5_sleep_m	Section Header: *Early Evening*<br>Sleeping and resting	checkbox<br>1 weai_time_5_sleep_m___1 12:00-12:15<br>2 weai_time_5_sleep_m___2 12:15-12:30<br>3 weai_time_5_sleep_m___3 12:30-12:45<br>4 weai_time_5_sleep_m___4 12:45-13:00<br>5 weai_time_5_sleep_m___5 13:00-13:15<br>6 weai_time_5_sleep_m___6 13:15-13:30<br>7 weai_time_5_sleep_m___7 13:30-13:45<br>8 weai_time_5_sleep_m___8 13:45-14:00
1462	weai_time_5_eat_m	Eating and drinking	checkbox<br>1 weai_time_5_eat_m___1 12:00-12:15<br>2 weai_time_5_eat_m___2 12:15-12:30<br>3 weai_time_5_eat_m___3 12:30-12:45<br>4 weai_time_5_eat_m___4 12:45-13:00<br>5 weai_time_5_eat_m___5 13:00-13:15<br>6 weai_time_5_eat_m___6 13:15-13:30<br>7 weai_time_5_eat_m___7 13:30-13:45<br>8 weai_time_5_eat_m___8 13:45-14:00
1463	weai_time_5_personal_m	Personal Care	checkbox<br>1 weai_time_5_personal_m___1 12:00-12:15<br>2 weai_time_5_personal_m___2 12:15-12:30<br>3 weai_time_5_personal_m___3 12:30-12:45<br>4 weai_time_5_personal_m___4 12:45-13:00<br>5 weai_time_5_personal_m___5 13:00-13:15<br>6 weai_time_5_personal_m___6 13:15-13:30<br>7 weai_time_5_personal_m___7 13:30-13:45<br>8 weai_time_5_personal_m___8 13:45-14:00
1464	weai_time_5_school_m	School (also homework)	checkbox<br>1 weai_time_5_school_m___1 12:00-12:15<br>2 weai_time_5_school_m___2 12:15-12:30<br>3 weai_time_5_school_m___3 12:30-12:45<br>4 weai_time_5_school_m___4 12:45-13:00<br>5 weai_time_5_school_m___5 13:00-13:15<br>6 weai_time_5_school_m___6 13:15-13:30<br>7 weai_time_5_school_m___7 13:30-13:45<br>8 weai_time_5_school_m___8 13:45-14:00
1465	weai_time_5_work_m	Work as employed	checkbox<br>1 weai_time_5_work_m___1 12:00-12:15<br>2 weai_time_5_work_m___2 12:15-12:30<br>3 weai_time_5_work_m___3 12:30-12:45<br>4 weai_time_5_work_m___4 12:45-13:00<br>5 weai_time_5_work_m___5 13:00-13:15<br>6 weai_time_5_work_m___6 13:15-13:30<br>7 weai_time_5_work_m___7 13:30-13:45<br>8 weai_time_5_work_m___8 13:45-14:00

Havelaar AH, *et al. BMJ Open* 2022; 12:e061311. doi: 10.1136/bmjopen-2022-061311

1466	weai_time_5_buisness_m	Own business work	checkbox<br>1 weai_time_5_buisness_m___1 12:00-12:15<br>2 weai_time_5_buisness_m___2 12:15-12:30<br>3 weai_time_5_buisness_m___3 12:30-12:45<br>4 weai_time_5_buisness_m___4 12:45-13:00<br>5 weai_time_5_buisness_m___5 13:00-13:15<br>6 weai_time_5_buisness_m___6 13:15-13:30<br>7 weai_time_5_buisness_m___7 13:30-13:45<br>8 weai_time_5_buisness_m___8 13:45-14:00
1467	weai_time_5_farm_m	Farming/livestock/fishing	checkbox<br>1 weai_time_5_farm_m___1 12:00-12:15<br>2 weai_time_5_farm_m___2 12:15-12:30<br>3 weai_time_5_farm_m___3 12:30-12:45<br>4 weai_time_5_farm_m___4 12:45-13:00<br>5 weai_time_5_farm_m___5 13:00-13:15<br>6 weai_time_5_farm_m___6 13:15-13:30<br>7 weai_time_5_farm_m___7 13:30-13:45<br>8 weai_time_5_farm_m___8 13:45-14:00
1468	weai_time_5_shop_m	Shopping/getting service (incl health services)	checkbox<br>1 weai_time_5_shop_m___1 12:00-12:15<br>2 weai_time_5_shop_m___2 12:15-12:30<br>3 weai_time_5_shop_m___3 12:30-12:45<br>4 weai_time_5_shop_m___4 12:45-13:00<br>5 weai_time_5_shop_m___5 13:00-13:15<br>6 weai_time_5_shop_m___6 13:15-13:30<br>7 weai_time_5_shop_m___7 13:30-13:45<br>8 weai_time_5_shop_m___8 13:45-14:00
1469	weai_time_5_weave_m	Weaving, sewing, textile care	checkbox<br>1 weai_time_5_weave_m___1 12:00-12:15<br>2 weai_time_5_weave_m___2 12:15-12:30<br>3 weai_time_5_weave_m___3 12:30-12:45<br>4 weai_time_5_weave_m___4 12:45-13:00<br>5 weai_time_5_weave_m___5 13:00-13:15<br>6 weai_time_5_weave_m___6 13:15-13:30<br>7 weai_time_5_weave_m___7 13:30-13:45<br>8 weai_time_5_weave_m___8 13:45-14:00
1470	weai_time_5_cook_m	Cooking	checkbox<br>1 weai_time_5_cook_m___1 12:00-12:15<br>2 weai_time_5_cook_m___2 12:15-12:30<br>3 weai_time_5_cook_m___3 12:30-12:45<br>4 weai_time_5_cook_m___4 12:45-13:00<br>5 weai_time_5_cook_m___5 13:00-13:15<br>6 weai_time_5_cook_m___6 13:15-13:30<br>7 weai_time_5_cook_m___7 13:30-13:45<br>8 weai_time_5_cook_m___8 13:45-14:00

1471	weai_time_5_domestic_m	Domestic work (incl fetching wood and water)	checkbox<br>1 weai_time_5_domestic_m___1 12:00-12:15<br>2 weai_time_5_domestic_m___2 12:15-12:30<br>3 weai_time_5_domestic_m___3 12:30-12:45<br>4 weai_time_5_domestic_m___4 12:45-13:00<br>5 weai_time_5_domestic_m___5 13:00-13:15<br>6 weai_time_5_domestic_m___6 13:15-13:30<br>7 weai_time_5_domestic_m___7 13:30-13:45<br>8 weai_time_5_domestic_m___8 13:45-14:00
1472	weai_time_5_care_m	Care for children/adults/elderly	checkbox<br>1 weai_time_5_care_m___1 12:00-12:15<br>2 weai_time_5_care_m___2 12:15-12:30<br>3 weai_time_5_care_m___3 12:30-12:45<br>4 weai_time_5_care_m___4 12:45-13:00<br>5 weai_time_5_care_m___5 13:00-13:15<br>6 weai_time_5_care_m___6 13:15-13:30<br>7 weai_time_5_care_m___7 13:30-13:45<br>8 weai_time_5_care_m___8 13:45-14:00
1473	weai_time_5_travel_m	Travelling and communiting	checkbox<br>1 weai_time_5_travel_m___1 12:00-12:15<br>2 weai_time_5_travel_m___2 12:15-12:30<br>3 weai_time_5_travel_m___3 12:30-12:45<br>4 weai_time_5_travel_m___4 12:45-13:00<br>5 weai_time_5_travel_m___5 13:00-13:15<br>6 weai_time_5_travel_m___6 13:15-13:30<br>7 weai_time_5_travel_m___7 13:30-13:45<br>8 weai_time_5_travel_m___8 13:45-14:00
1474	weai_time_5_tv_m	Watching TV/listening to radio/reading	checkbox<br>1 weai_time_5_tv_m___1 12:00-12:15<br>2 weai_time_5_tv_m___2 12:15-12:30<br>3 weai_time_5_tv_m___3 12:30-12:45<br>4 weai_time_5_tv_m___4 12:45-13:00<br>5 weai_time_5_tv_m___5 13:00-13:15<br>6 weai_time_5_tv_m___6 13:15-13:30<br>7 weai_time_5_tv_m___7 13:30-13:45<br>8 weai_time_5_tv_m___8 13:45-14:00
1475	weai_time_5_exercising_m	Exercising	checkbox<br>1 weai_time_5_exercising_m___1 12:00-12:15<br>2 weai_time_5_exercising_m___2 12:15-12:30<br>3 weai_time_5_exercising_m___3 12:30-12:45<br>4 weai_time_5_exercising_m___4 12:45-13:00<br>5 weai_time_5_exercising_m___5 13:00-13:15<br>6 weai_time_5_exercising_m___6 13:15-13:30<br>7 weai_time_5_exercising_m___7 13:30-13:45<br>8 weai_time_5_exercising_m___8 13:45-14:00

1476	weai_time_5_social_m	Social activities and hobbies	checkbox<br>1 weai_time_5_social_m___1 12:00-12:15<br>2 weai_time_5_social_m___2 12:15-12:30<br>3 weai_time_5_social_m___3 12:30-12:45<br>4 weai_time_5_social_m___4 12:45-13:00<br>5 weai_time_5_social_m___5 13:00-13:15<br>6 weai_time_5_social_m___6 13:15-13:30<br>7 weai_time_5_social_m___7 13:30-13:45<br>8 weai_time_5_social_m___8 13:45-14:00
1477	weai_time_5_religious_m	Religious activities	checkbox<br>1 weai_time_5_religious_m___1 12:00-12:15<br>2 weai_time_5_religious_m___2 12:15-12:30<br>3 weai_time_5_religious_m___3 12:30-12:45<br>4 weai_time_5_religious_m___4 12:45-13:00<br>5 weai_time_5_religious_m___5 13:00-13:15<br>6 weai_time_5_religious_m___6 13:15-13:30<br>7 weai_time_5_religious_m___7 13:30-13:45<br>8 weai_time_5_religious_m___8 13:45-14:00
1478	weai_time_5_other_m	Other (specify below)	checkbox<br>1 weai_time_5_other_m___1 12:00-12:15<br>2 weai_time_5_other_m___2 12:15-12:30<br>3 weai_time_5_other_m___3 12:30-12:45<br>4 weai_time_5_other_m___4 12:45-13:00<br>5 weai_time_5_other_m___5 13:00-13:15<br>6 weai_time_5_other_m___6 13:15-13:30<br>7 weai_time_5_other_m___7 13:30-13:45<br>8 weai_time_5_other_m___8 13:45-14:00
1479	weai_time_5_other_specify_m<br>Show the field ONLY if:<br>[weai_time_5_other_m(1)] or [weai_time_5_other_m(2)] or [weai_time_5_other_m(3)] or [weai_time_5_other_m(4)] or [weai_time_5_other_m(5)] or [weai_time_5_other_m(6)] or [weai_time_5_other_m(7)] or [weai_time_5_other_m(8)]	Specify Other	text<br>Custom alignment: LV
1480	weai_time_6_sleep_m	Section Header: *Late Evening/Night*<br>Sleeping and resting	checkbox<br>1 weai_time_6_sleep_m___1 14:00-14:15<br>2 weai_time_6_sleep_m___2 14:15-14:30<br>3 weai_time_6_sleep_m___3 14:30-14:45<br>4 weai_time_6_sleep_m___4 14:45-15:00<br>5 weai_time_6_sleep_m___5 15:00-15:15<br>6 weai_time_6_sleep_m___6 15:15-15:30<br>7 weai_time_6_sleep_m___7 15:30-15:45<br>8 weai_time_6_sleep_m___8 15:45-16:00

Havelaar AH, *et al. BMJ Open* 2022; 12:e061311. doi: 10.1136/bmjopen-2022-061311

1481	weai_time_6_eat_m	Eating and drinking	checkbox<br>1 weai_time_6_eat_m___1 14:00-14:15<br>2 weai_time_6_eat_m___2 14:15-14:30<br>3 weai_time_6_eat_m___3 14:30-14:45<br>4 weai_time_6_eat_m___4 14:45-15:00<br>5 weai_time_6_eat_m___5 15:00-15:15<br>6 weai_time_6_eat_m___6 15:15-15:30<br>7 weai_time_6_eat_m___7 15:30-15:45<br>8 weai_time_6_eat_m___8 15:45-16:00
1482	weai_time_6_personal_m	Personal Care	checkbox<br>1 weai_time_6_personal_m___1 14:00-14:15<br>2 weai_time_6_personal_m___2 14:15-14:30<br>3 weai_time_6_personal_m___3 14:30-14:45<br>4 weai_time_6_personal_m___4 14:45-15:00<br>5 weai_time_6_personal_m___5 15:00-15:15<br>6 weai_time_6_personal_m___6 15:15-15:30<br>7 weai_time_6_personal_m___7 15:30-15:45<br>8 weai_time_6_personal_m___8 15:45-16:00
1483	weai_time_6_school_m	School (also homework)	checkbox<br>1 weai_time_6_school_m___1 14:00-14:15<br>2 weai_time_6_school_m___2 14:15-14:30<br>3 weai_time_6_school_m___3 14:30-14:45<br>4 weai_time_6_school_m___4 14:45-15:00<br>5 weai_time_6_school_m___5 15:00-15:15<br>6 weai_time_6_school_m___6 15:15-15:30<br>7 weai_time_6_school_m___7 15:30-15:45<br>8 weai_time_6_school_m___8 15:45-16:00
1484	weai_time_6_work_m	Work as employed	checkbox<br>1 weai_time_6_work_m___1 14:00-14:15<br>2 weai_time_6_work_m___2 14:15-14:30<br>3 weai_time_6_work_m___3 14:30-14:45<br>4 weai_time_6_work_m___4 14:45-15:00<br>5 weai_time_6_work_m___5 15:00-15:15<br>6 weai_time_6_work_m___6 15:15-15:30<br>7 weai_time_6_work_m___7 15:30-15:45<br>8 weai_time_6_work_m___8 15:45-16:00
1485	weai_time_6_buisness_m	Own business work	checkbox<br>1 weai_time_6_buisness_m___1 14:00-14:15<br>2 weai_time_6_buisness_m___2 14:15-14:30<br>3 weai_time_6_buisness_m___3 14:30-14:45<br>4 weai_time_6_buisness_m___4 14:45-15:00<br>5 weai_time_6_buisness_m___5 15:00-15:15<br>6 weai_time_6_buisness_m___6 15:15-15:30<br>7 weai_time_6_buisness_m___7 15:30-15:45<br>8 weai_time_6_buisness_m___8 15:45-16:00

1486	weai_time_6_farm_m	Farming/livestock/fishing	checkbox<br>1 weai_time_6_farm_m___1 14:00-14:15<br>2 weai_time_6_farm_m___2 14:15-14:30<br>3 weai_time_6_farm_m___3 14:30-14:45<br>4 weai_time_6_farm_m___4 14:45-15:00<br>5 weai_time_6_farm_m___5 15:00-15:15<br>6 weai_time_6_farm_m___6 15:15-15:30<br>7 weai_time_6_farm_m___7 15:30-15:45<br>8 weai_time_6_farm_m___8 15:45-16:00
1487	weai_time_6_shop_m	Shopping/getting service (incl health services)	checkbox<br>1 weai_time_6_shop_m___1 14:00-14:15<br>2 weai_time_6_shop_m___2 14:15-14:30<br>3 weai_time_6_shop_m___3 14:30-14:45<br>4 weai_time_6_shop_m___4 14:45-15:00<br>5 weai_time_6_shop_m___5 15:00-15:15<br>6 weai_time_6_shop_m___6 15:15-15:30<br>7 weai_time_6_shop_m___7 15:30-15:45<br>8 weai_time_6_shop_m___8 15:45-16:00
1488	weai_time_6_weave_m	Weaving, sewing, textile care	checkbox<br>1 weai_time_6_weave_m___1 14:00-14:15<br>2 weai_time_6_weave_m___2 14:15-14:30<br>3 weai_time_6_weave_m___3 14:30-14:45<br>4 weai_time_6_weave_m___4 14:45-15:00<br>5 weai_time_6_weave_m___5 15:00-15:15<br>6 weai_time_6_weave_m___6 15:15-15:30<br>7 weai_time_6_weave_m___7 15:30-15:45<br>8 weai_time_6_weave_m___8 15:45-16:00
1489	weai_time_6_cook_m	Cooking	checkbox<br>1 weai_time_6_cook_m___1 14:00-14:15<br>2 weai_time_6_cook_m___2 14:15-14:30<br>3 weai_time_6_cook_m___3 14:30-14:45<br>4 weai_time_6_cook_m___4 14:45-15:00<br>5 weai_time_6_cook_m___5 15:00-15:15<br>6 weai_time_6_cook_m___6 15:15-15:30<br>7 weai_time_6_cook_m___7 15:30-15:45<br>8 weai_time_6_cook_m___8 15:45-16:00
1490	weai_time_6_domestic_m	Domestic work (incl fetching wood and water)	checkbox<br>1 weai_time_6_domestic_m___1 14:00-14:15<br>2 weai_time_6_domestic_m___2 14:15-14:30<br>3 weai_time_6_domestic_m___3 14:30-14:45<br>4 weai_time_6_domestic_m___4 14:45-15:00<br>5 weai_time_6_domestic_m___5 15:00-15:15<br>6 weai_time_6_domestic_m___6 15:15-15:30<br>7 weai_time_6_domestic_m___7 15:30-15:45<br>8 weai_time_6_domestic_m___8 15:45-16:00

Supplemental material BMJ Publishing Group Limited (BMJ) disclaims all liability and responsibility arising from any reliance placed on this supplemental material which has been supplied by the author(s) BMJ Open

1491	weai_time_6_care_m	Care for children/adults/elderly	checkbox<br>1 | weai_time_6_care_m___1 | 14:00-14:15<br>2 | weai_time_6_care_m___2 | 14:15-14:30<br>3 | weai_time_6_care_m___3 | 14:30-14:45<br>4 | weai_time_6_care_m___4 | 14:45-15:00<br>5 | weai_time_6_care_m___5 | 15:00-15:15<br>6 | weai_time_6_care_m___6 | 15:15-15:30<br>7 | weai_time_6_care_m___7 | 15:30-15:45<br>8 | weai_time_6_care_m___8 | 15:45-16:00
1492	weai_time_6_travel_m	Travelling and communiting	checkbox<br>1 | weai_time_6_travel_m___1 | 14:00-14:15<br>2 | weai_time_6_travel_m___2 | 14:15-14:30<br>3 | weai_time_6_travel_m___3 | 14:30-14:45<br>4 | weai_time_6_travel_m___4 | 14:45-15:00<br>5 | weai_time_6_travel_m___5 | 15:00-15:15<br>6 | weai_time_6_travel_m___6 | 15:15-15:30<br>7 | weai_time_6_travel_m___7 | 15:30-15:45<br>8 | weai_time_6_travel_m___8 | 15:45-16:00
1493	weai_time_6_tv_m	Watching TV/listening to radio/reading	checkbox<br>1 | weai_time_6_tv_m___1 | 14:00-14:15<br>2 | weai_time_6_tv_m___2 | 14:15-14:30<br>3 | weai_time_6_tv_m___3 | 14:30-14:45<br>4 | weai_time_6_tv_m___4 | 14:45-15:00<br>5 | weai_time_6_tv_m___5 | 15:00-15:15<br>6 | weai_time_6_tv_m___6 | 15:15-15:30<br>7 | weai_time_6_tv_m___7 | 15:30-15:45<br>8 | weai_time_6_tv_m___8 | 15:45-16:00
1494	weai_time_6_exercising_m	Exercising	checkbox<br>1 | weai_time_6_exercising_m___1 | 14:00-14:15<br>2 | weai_time_6_exercising_m___2 | 14:15-14:30<br>3 | weai_time_6_exercising_m___3 | 14:30-14:45<br>4 | weai_time_6_exercising_m___4 | 14:45-15:00<br>5 | weai_time_6_exercising_m___5 | 15:00-15:15<br>6 | weai_time_6_exercising_m___6 | 15:15-15:30<br>7 | weai_time_6_exercising_m___7 | 15:30-15:45<br>8 | weai_time_6_exercising_m___8 | 15:45-16:00
1495	weai_time_6_social_m	Social activities and hobbies	checkbox<br>1 | weai_time_6_social_m___1 | 14:00-14:15<br>2 | weai_time_6_social_m___2 | 14:15-14:30<br>3 | weai_time_6_social_m___3 | 14:30-14:45<br>4 | weai_time_6_social_m___4 | 14:45-15:00<br>5 | weai_time_6_social_m___5 | 15:00-15:15<br>6 | weai_time_6_social_m___6 | 15:15-15:30<br>7 | weai_time_6_social_m___7 | 15:30-15:45<br>8 | weai_time_6_social_m___8 | 15:45-16:00

Havelaar AH, *et al. BMJ Open* 2022; 12:e061311. doi: 10.1136/bmjopen-2022-061311

1496	weai_time_6_religious_m	Religious activities	checkbox<br>1 weai_time_6_religious_m___1 14:00-14:15<br>2 weai_time_6_religious_m___2 14:15-14:30<br>3 weai_time_6_religious_m___3 14:30-14:45<br>4 weai_time_6_religious_m___4 14:45-15:00<br>5 weai_time_6_religious_m___5 15:00-15:15<br>6 weai_time_6_religious_m___6 15:15-15:30<br>7 weai_time_6_religious_m___7 15:30-15:45<br>8 weai_time_6_religious_m___8 15:45-16:00
1497	weai_time_6_other_m	Other (specify below)	checkbox<br>1 weai_time_6_other_m___1 14:00-14:15<br>2 weai_time_6_other_m___2 14:15-14:30<br>3 weai_time_6_other_m___3 14:30-14:45<br>4 weai_time_6_other_m___4 14:45-15:00<br>5 weai_time_6_other_m___5 15:00-15:15<br>6 weai_time_6_other_m___6 15:15-15:30<br>7 weai_time_6_other_m___7 15:30-15:45<br>8 weai_time_6_other_m___8 15:45-16:00
1498	weai_time_6_other_specify_m<br>Show the field ONLY if:<br>[weai_time_6_other_m(1)] or<br>[weai_time_6_other_m(2)] or<br>[weai_time_6_other_m(3)] or<br>[weai_time_6_other_m(4)] or<br>[weai_time_6_other_m(5)] or<br>[weai_time_6_other_m(6)] or<br>[weai_time_6_other_m(7)] or<br>[weai_time_6_other_m(8)]	Specify Other	text<br>Custom alignment: LV
1499	weai_time_7_sleep_m	Section Header: *Night/Early morning*<br>Sleeping and resting	checkbox<br>1 weai_time_7_sleep_m___1 16:00-16:15<br>2 weai_time_7_sleep_m___2 16:15-16:30<br>3 weai_time_7_sleep_m___3 16:30-16:45<br>4 weai_time_7_sleep_m___4 16:45-17:00<br>5 weai_time_7_sleep_m___5 17:00-17:15<br>6 weai_time_7_sleep_m___6 17:15-17:30<br>7 weai_time_7_sleep_m___7 17:30-17:45<br>8 weai_time_7_sleep_m___8 17:45-18:00
1500	weai_time_7_eat_m	Eating and drinking	checkbox<br>1 weai_time_7_eat_m___1 16:00-16:15<br>2 weai_time_7_eat_m___2 16:15-16:30<br>3 weai_time_7_eat_m___3 16:30-16:45<br>4 weai_time_7_eat_m___4 16:45-17:00<br>5 weai_time_7_eat_m___5 17:00-17:15<br>6 weai_time_7_eat_m___6 17:15-17:30<br>7 weai_time_7_eat_m___7 17:30-17:45<br>8 weai_time_7_eat_m___8 17:45-18:00

1501	weai_time_7_personal_m	Personal Care	checkbox<br>1 weai_time_7_personal_m___1 16:00-16:15<br>2 weai_time_7_personal_m___2 16:15-16:30<br>3 weai_time_7_personal_m___3 16:30-16:45<br>4 weai_time_7_personal_m___4 16:45-17:00<br>5 weai_time_7_personal_m___5 17:00-17:15<br>6 weai_time_7_personal_m___6 17:15-17:30<br>7 weai_time_7_personal_m___7 17:30-17:45<br>8 weai_time_7_personal_m___8 17:45-18:00
1502	weai_time_7_school_m	School (also homework)	checkbox<br>1 weai_time_7_school_m___1 16:00-16:15<br>2 weai_time_7_school_m___2 16:15-16:30<br>3 weai_time_7_school_m___3 16:30-16:45<br>4 weai_time_7_school_m___4 16:45-17:00<br>5 weai_time_7_school_m___5 17:00-17:15<br>6 weai_time_7_school_m___6 17:15-17:30<br>7 weai_time_7_school_m___7 17:30-17:45<br>8 weai_time_7_school_m___8 17:45-18:00
1503	weai_time_7_work_m	Work as employed	checkbox<br>1 weai_time_7_work_m___1 16:00-16:15<br>2 weai_time_7_work_m___2 16:15-16:30<br>3 weai_time_7_work_m___3 16:30-16:45<br>4 weai_time_7_work_m___4 16:45-17:00<br>5 weai_time_7_work_m___5 17:00-17:15<br>6 weai_time_7_work_m___6 17:15-17:30<br>7 weai_time_7_work_m___7 17:30-17:45<br>8 weai_time_7_work_m___8 17:45-18:00
1504	weai_time_7_buisness_m	Own business work	checkbox<br>1 weai_time_7_buisness_m___1 16:00-16:15<br>2 weai_time_7_buisness_m___2 16:15-16:30<br>3 weai_time_7_buisness_m___3 16:30-16:45<br>4 weai_time_7_buisness_m___4 16:45-17:00<br>5 weai_time_7_buisness_m___5 17:00-17:15<br>6 weai_time_7_buisness_m___6 17:15-17:30<br>7 weai_time_7_buisness_m___7 17:30-17:45<br>8 weai_time_7_buisness_m___8 17:45-18:00
1505	weai_time_7_farm_m	Farming/livestock/fishing	checkbox<br>1 weai_time_7_farm_m___1 16:00-16:15<br>2 weai_time_7_farm_m___2 16:15-16:30<br>3 weai_time_7_farm_m___3 16:30-16:45<br>4 weai_time_7_farm_m___4 16:45-17:00<br>5 weai_time_7_farm_m___5 17:00-17:15<br>6 weai_time_7_farm_m___6 17:15-17:30<br>7 weai_time_7_farm_m___7 17:30-17:45<br>8 weai_time_7_farm_m___8 17:45-18:00

Havelaar AH, *et al. BMJ Open* 2022; 12:e061311. doi: 10.1136/bmjopen-2022-061311

1506	weai_time_7_shop_m	Shopping/getting service (incl health services)	checkbox<br>1 weai_time_7_shop_m___1 16:00-16:15<br>2 weai_time_7_shop_m___2 16:15-16:30<br>3 weai_time_7_shop_m___3 16:30-16:45<br>4 weai_time_7_shop_m___4 16:45-17:00<br>5 weai_time_7_shop_m___5 17:00-17:15<br>6 weai_time_7_shop_m___6 17:15-17:30<br>7 weai_time_7_shop_m___7 17:30-17:45<br>8 weai_time_7_shop_m___8 17:45-18:00
1507	weai_time_7_weave_m	Weaving, sewing, textile care	checkbox<br>1 weai_time_7_weave_m___1 16:00-16:15<br>2 weai_time_7_weave_m___2 16:15-16:30<br>3 weai_time_7_weave_m___3 16:30-16:45<br>4 weai_time_7_weave_m___4 16:45-17:00<br>5 weai_time_7_weave_m___5 17:00-17:15<br>6 weai_time_7_weave_m___6 17:15-17:30<br>7 weai_time_7_weave_m___7 17:30-17:45<br>8 weai_time_7_weave_m___8 17:45-18:00
1508	weai_time_7_cook_m	Cooking	checkbox<br>1 weai_time_7_cook_m___1 16:00-16:15<br>2 weai_time_7_cook_m___2 16:15-16:30<br>3 weai_time_7_cook_m___3 16:30-16:45<br>4 weai_time_7_cook_m___4 16:45-17:00<br>5 weai_time_7_cook_m___5 17:00-17:15<br>6 weai_time_7_cook_m___6 17:15-17:30<br>7 weai_time_7_cook_m___7 17:30-17:45<br>8 weai_time_7_cook_m___8 17:45-18:00
1509	weai_time_7_domestic_m	Domestic work (incl fetching wood and water)	checkbox<br>1 weai_time_7_domestic_m___1 16:00-16:15<br>2 weai_time_7_domestic_m___2 16:15-16:30<br>3 weai_time_7_domestic_m___3 16:30-16:45<br>4 weai_time_7_domestic_m___4 16:45-17:00<br>5 weai_time_7_domestic_m___5 17:00-17:15<br>6 weai_time_7_domestic_m___6 17:15-17:30<br>7 weai_time_7_domestic_m___7 17:30-17:45<br>8 weai_time_7_domestic_m___8 17:45-18:00
1510	weai_time_7_care_m	Care for children/adults/elderly	checkbox<br>1 weai_time_7_care_m___1 16:00-16:15<br>2 weai_time_7_care_m___2 16:15-16:30<br>3 weai_time_7_care_m___3 16:30-16:45<br>4 weai_time_7_care_m___4 16:45-17:00<br>5 weai_time_7_care_m___5 17:00-17:15<br>6 weai_time_7_care_m___6 17:15-17:30<br>7 weai_time_7_care_m___7 17:30-17:45<br>8 weai_time_7_care_m___8 17:45-18:00

1511	weai_time_7_travel_m	Travelling and communiting	checkbox<br>1 weai_time_7_travel_m___1 16:00-16:15<br>2 weai_time_7_travel_m___2 16:15-16:30<br>3 weai_time_7_travel_m___3 16:30-16:45<br>4 weai_time_7_travel_m___4 16:45-17:00<br>5 weai_time_7_travel_m___5 17:00-17:15<br>6 weai_time_7_travel_m___6 17:15-17:30<br>7 weai_time_7_travel_m___7 17:30-17:45<br>8 weai_time_7_travel_m___8 17:45-18:00
1512	weai_time_7_tv_m	Watching TV/listening to radio/reading	checkbox<br>1 weai_time_7_tv_m___1 16:00-16:15<br>2 weai_time_7_tv_m___2 16:15-16:30<br>3 weai_time_7_tv_m___3 16:30-16:45<br>4 weai_time_7_tv_m___4 16:45-17:00<br>5 weai_time_7_tv_m___5 17:00-17:15<br>6 weai_time_7_tv_m___6 17:15-17:30<br>7 weai_time_7_tv_m___7 17:30-17:45<br>8 weai_time_7_tv_m___8 17:45-18:00
1513	weai_time_7_exercising_m	Exercising	checkbox<br>1 weai_time_7_exercising_m___1 16:00-16:15<br>2 weai_time_7_exercising_m___2 16:15-16:30<br>3 weai_time_7_exercising_m___3 16:30-16:45<br>4 weai_time_7_exercising_m___4 16:45-17:00<br>5 weai_time_7_exercising_m___5 17:00-17:15<br>6 weai_time_7_exercising_m___6 17:15-17:30<br>7 weai_time_7_exercising_m___7 17:30-17:45<br>8 weai_time_7_exercising_m___8 17:45-18:00
1514	weai_time_7_social_m	Social activities and hobbies	checkbox<br>1 weai_time_7_social_m___1 16:00-16:15<br>2 weai_time_7_social_m___2 16:15-16:30<br>3 weai_time_7_social_m___3 16:30-16:45<br>4 weai_time_7_social_m___4 16:45-17:00<br>5 weai_time_7_social_m___5 17:00-17:15<br>6 weai_time_7_social_m___6 17:15-17:30<br>7 weai_time_7_social_m___7 17:30-17:45<br>8 weai_time_7_social_m___8 17:45-18:00
1515	weai_time_7_religious_m	Religious activities	checkbox<br>1 weai_time_7_religious_m___1 16:00-16:15<br>2 weai_time_7_religious_m___2 16:15-16:30<br>3 weai_time_7_religious_m___3 16:30-16:45<br>4 weai_time_7_religious_m___4 16:45-17:00<br>5 weai_time_7_religious_m___5 17:00-17:15<br>6 weai_time_7_religious_m___6 17:15-17:30<br>7 weai_time_7_religious_m___7 17:30-17:45<br>8 weai_time_7_religious_m___8 17:45-18:00

1516	weai_time_7_other_m	Other (specify below)	checkbox<br>1 weai_time_7_other_m___1 16:00-16:15<br>2 weai_time_7_other_m___2 16:15-16:30<br>3 weai_time_7_other_m___3 16:30-16:45<br>4 weai_time_7_other_m___4 16:45-17:00<br>5 weai_time_7_other_m___5 17:00-17:15<br>6 weai_time_7_other_m___6 17:15-17:30<br>7 weai_time_7_other_m___7 17:30-17:45<br>8 weai_time_7_other_m___8 17:45-18:00
1517	weai_time_7_other_specify_m<br><br>Show the field ONLY if:<br>[weai_time_7_other_m(1)] or<br>[weai_time_7_other_m(2)] or<br>[weai_time_7_other_m(3)] or<br>[weai_time_7_other_m(4)] or<br>[weai_time_7_other_m(5)] or<br>[weai_time_7_other_m(6)] or<br>[weai_time_7_other_m(7)] or<br>[weai_time_7_other_m(8)]	Specify Other	text<br>Custom alignment: LV
1518	weai_time_8_sleep_m	Section Header: *Night/Early morning*<br>Sleeping and resting	checkbox<br>1 weai_time_8_sleep_m___1 18:00-18:15<br>2 weai_time_8_sleep_m___2 18:15-18:30<br>3 weai_time_8_sleep_m___3 18:30-18:45<br>4 weai_time_8_sleep_m___4 18:45-19:00<br>5 weai_time_8_sleep_m___5 19:00-19:15<br>6 weai_time_8_sleep_m___6 19:15-19:30<br>7 weai_time_8_sleep_m___7 19:30-19:45<br>8 weai_time_8_sleep_m___8 19:45-20:00
1519	weai_time_8_eat_m	Eating and drinking	checkbox<br>1 weai_time_8_eat_m___1 18:00-18:15<br>2 weai_time_8_eat_m___2 18:15-18:30<br>3 weai_time_8_eat_m___3 18:30-18:45<br>4 weai_time_8_eat_m___4 18:45-19:00<br>5 weai_time_8_eat_m___5 19:00-19:15<br>6 weai_time_8_eat_m___6 19:15-19:30<br>7 weai_time_8_eat_m___7 19:30-19:45<br>8 weai_time_8_eat_m___8 19:45-20:00
1520	weai_time_8_personal_m	Personal Care	checkbox<br>1 weai_time_8_personal_m___1 18:00-18:15<br>2 weai_time_8_personal_m___2 18:15-18:30<br>3 weai_time_8_personal_m___3 18:30-18:45<br>4 weai_time_8_personal_m___4 18:45-19:00<br>5 weai_time_8_personal_m___5 19:00-19:15<br>6 weai_time_8_personal_m___6 19:15-19:30<br>7 weai_time_8_personal_m___7 19:30-19:45<br>8 weai_time_8_personal_m___8 19:45-20:00

1521	weai_time_8_school_m	School (also homework)	checkbox<br>1 weai_time_8_school_m___1 18:00-18:15<br>2 weai_time_8_school_m___2 18:15-18:30<br>3 weai_time_8_school_m___3 18:30-18:45<br>4 weai_time_8_school_m___4 18:45-19:00<br>5 weai_time_8_school_m___5 19:00-19:15<br>6 weai_time_8_school_m___6 19:15-19:30<br>7 weai_time_8_school_m___7 19:30-19:45<br>8 weai_time_8_school_m___8 19:45-20:00
1522	weai_time_8_work_m	Work as employed	checkbox<br>1 weai_time_8_work_m___1 18:00-18:15<br>2 weai_time_8_work_m___2 18:15-18:30<br>3 weai_time_8_work_m___3 18:30-18:45<br>4 weai_time_8_work_m___4 18:45-19:00<br>5 weai_time_8_work_m___5 19:00-19:15<br>6 weai_time_8_work_m___6 19:15-19:30<br>7 weai_time_8_work_m___7 19:30-19:45<br>8 weai_time_8_work_m___8 19:45-20:00
1523	weai_time_8_buisness_m	Own business work	checkbox<br>1 weai_time_8_buisness_m___1 18:00-18:15<br>2 weai_time_8_buisness_m___2 18:15-18:30<br>3 weai_time_8_buisness_m___3 18:30-18:45<br>4 weai_time_8_buisness_m___4 18:45-19:00<br>5 weai_time_8_buisness_m___5 19:00-19:15<br>6 weai_time_8_buisness_m___6 19:15-19:30<br>7 weai_time_8_buisness_m___7 19:30-19:45<br>8 weai_time_8_buisness_m___8 19:45-20:00
1524	weai_time_8_farm_m	Farming/livestock/fishing	checkbox<br>1 weai_time_8_farm_m___1 18:00-18:15<br>2 weai_time_8_farm_m___2 18:15-18:30<br>3 weai_time_8_farm_m___3 18:30-18:45<br>4 weai_time_8_farm_m___4 18:45-19:00<br>5 weai_time_8_farm_m___5 19:00-19:15<br>6 weai_time_8_farm_m___6 19:15-19:30<br>7 weai_time_8_farm_m___7 19:30-19:45<br>8 weai_time_8_farm_m___8 19:45-20:00
1525	weai_time_8_shop_m	Shopping/getting service (incl health services)	checkbox<br>1 weai_time_8_shop_m___1 18:00-18:15<br>2 weai_time_8_shop_m___2 18:15-18:30<br>3 weai_time_8_shop_m___3 18:30-18:45<br>4 weai_time_8_shop_m___4 18:45-19:00<br>5 weai_time_8_shop_m___5 19:00-19:15<br>6 weai_time_8_shop_m___6 19:15-19:30<br>7 weai_time_8_shop_m___7 19:30-19:45<br>8 weai_time_8_shop_m___8 19:45-20:00

Havelaar AH, *et al. BMJ Open* 2022; 12:e061311. doi: 10.1136/bmjopen-2022-061311

1526	weai_time_8_weave_m	Weaving, sewing, textile care	checkbox<br>1 weai_time_8_weave_m___1 18:00-18:15<br>2 weai_time_8_weave_m___2 18:15-18:30<br>3 weai_time_8_weave_m___3 18:30-18:45<br>4 weai_time_8_weave_m___4 18:45-19:00<br>5 weai_time_8_weave_m___5 19:00-19:15<br>6 weai_time_8_weave_m___6 19:15-19:30<br>7 weai_time_8_weave_m___7 19:30-19:45<br>8 weai_time_8_weave_m___8 19:45-20:00
1527	weai_time_8_cook_m	Cooking	checkbox<br>1 weai_time_8_cook_m___1 18:00-18:15<br>2 weai_time_8_cook_m___2 18:15-18:30<br>3 weai_time_8_cook_m___3 18:30-18:45<br>4 weai_time_8_cook_m___4 18:45-19:00<br>5 weai_time_8_cook_m___5 19:00-19:15<br>6 weai_time_8_cook_m___6 19:15-19:30<br>7 weai_time_8_cook_m___7 19:30-19:45<br>8 weai_time_8_cook_m___8 19:45-20:00
1528	weai_time_8_domestic_m	Domestic work (incl fetching wood and water)	checkbox<br>1 weai_time_8_domestic_m___1 18:00-18:15<br>2 weai_time_8_domestic_m___2 18:15-18:30<br>3 weai_time_8_domestic_m___3 18:30-18:45<br>4 weai_time_8_domestic_m___4 18:45-19:00<br>5 weai_time_8_domestic_m___5 19:00-19:15<br>6 weai_time_8_domestic_m___6 19:15-19:30<br>7 weai_time_8_domestic_m___7 19:30-19:45<br>8 weai_time_8_domestic_m___8 19:45-20:00
1529	weai_time_8_care_m	Care for children/adults/elderly	checkbox<br>1 weai_time_8_care_m___1 18:00-18:15<br>2 weai_time_8_care_m___2 18:15-18:30<br>3 weai_time_8_care_m___3 18:30-18:45<br>4 weai_time_8_care_m___4 18:45-19:00<br>5 weai_time_8_care_m___5 19:00-19:15<br>6 weai_time_8_care_m___6 19:15-19:30<br>7 weai_time_8_care_m___7 19:30-19:45<br>8 weai_time_8_care_m___8 19:45-20:00
1530	weai_time_8_travel_m	Travelling and communiting	checkbox<br>1 weai_time_8_travel_m___1 18:00-18:15<br>2 weai_time_8_travel_m___2 18:15-18:30<br>3 weai_time_8_travel_m___3 18:30-18:45<br>4 weai_time_8_travel_m___4 18:45-19:00<br>5 weai_time_8_travel_m___5 19:00-19:15<br>6 weai_time_8_travel_m___6 19:15-19:30<br>7 weai_time_8_travel_m___7 19:30-19:45<br>8 weai_time_8_travel_m___8 19:45-20:00

Havelaar AH, *et al. BMJ Open* 2022; 12:e061311. doi: 10.1136/bmjopen-2022-061311

1531	weai_time_8_tv_m	Watching TV/listening to radio/reading	checkbox<br>1 weai_time_8_tv_m___1 18:00-18:15<br>2 weai_time_8_tv_m___2 18:15-18:30<br>3 weai_time_8_tv_m___3 18:30-18:45<br>4 weai_time_8_tv_m___4 18:45-19:00<br>5 weai_time_8_tv_m___5 19:00-19:15<br>6 weai_time_8_tv_m___6 19:15-19:30<br>7 weai_time_8_tv_m___7 19:30-19:45<br>8 weai_time_8_tv_m___8 19:45-20:00
1532	weai_time_8_exercising_m	Exercising	checkbox<br>1 weai_time_8_exercising_m___1 18:00-18:15<br>2 weai_time_8_exercising_m___2 18:15-18:30<br>3 weai_time_8_exercising_m___3 18:30-18:45<br>4 weai_time_8_exercising_m___4 18:45-19:00<br>5 weai_time_8_exercising_m___5 19:00-19:15<br>6 weai_time_8_exercising_m___6 19:15-19:30<br>7 weai_time_8_exercising_m___7 19:30-19:45<br>8 weai_time_8_exercising_m___8 19:45-20:00
1533	weai_time_8_social_m	Social activities and hobbies	checkbox<br>1 weai_time_8_social_m___1 18:00-18:15<br>2 weai_time_8_social_m___2 18:15-18:30<br>3 weai_time_8_social_m___3 18:30-18:45<br>4 weai_time_8_social_m___4 18:45-19:00<br>5 weai_time_8_social_m___5 19:00-19:15<br>6 weai_time_8_social_m___6 19:15-19:30<br>7 weai_time_8_social_m___7 19:30-19:45<br>8 weai_time_8_social_m___8 19:45-20:00
1534	weai_time_8_religious_m	Religious activities	checkbox<br>1 weai_time_8_religious_m___1 18:00-18:15<br>2 weai_time_8_religious_m___2 18:15-18:30<br>3 weai_time_8_religious_m___3 18:30-18:45<br>4 weai_time_8_religious_m___4 18:45-19:00<br>5 weai_time_8_religious_m___5 19:00-19:15<br>6 weai_time_8_religious_m___6 19:15-19:30<br>7 weai_time_8_religious_m___7 19:30-19:45<br>8 weai_time_8_religious_m___8 19:45-20:00
1535	weai_time_8_other_m	Other (specify below)	checkbox<br>1 weai_time_8_other_m___1 18:00-18:15<br>2 weai_time_8_other_m___2 18:15-18:30<br>3 weai_time_8_other_m___3 18:30-18:45<br>4 weai_time_8_other_m___4 18:45-19:00<br>5 weai_time_8_other_m___5 19:00-19:15<br>6 weai_time_8_other_m___6 19:15-19:30<br>7 weai_time_8_other_m___7 19:30-19:45<br>8 weai_time_8_other_m___8 19:45-20:00

Havelaar AH, *et al. BMJ Open* 2022; 12:e061311. doi: 10.1136/bmjopen-2022-061311

1536	weai_time_8_other_specify_m<br>Show the field ONLY if:<br>[weai_time_8_other_m(1)] or<br>[weai_time_8_other_m(2)] or<br>[weai_time_8_other_m(3)] or<br>[weai_time_8_other_m(4)] or<br>[weai_time_8_other_m(5)] or<br>[weai_time_8_other_m(6)] or<br>[weai_time_8_other_m(7)] or<br>[weai_time_8_other_m(8)]	Specify Other	text<br>Custom alignment: LV
1537	weai_time_9_sleep_m	Section Header: *Night/Early morning*<br>Sleeping and resting	checkbox<br>1 weai_time_9_sleep_m___1 20:00-20:15<br>2 weai_time_9_sleep_m___2 20:15-20:30<br>3 weai_time_9_sleep_m___3 20:30-20:45<br>4 weai_time_9_sleep_m___4 20:45-21:00<br>5 weai_time_9_sleep_m___5 21:00-21:15<br>6 weai_time_9_sleep_m___6 21:15-21:30<br>7 weai_time_9_sleep_m___7 21:30-21:45<br>8 weai_time_9_sleep_m___8 21:45-22:00
1538	weai_time_9_eat_m	Eating and drinking	checkbox<br>1 weai_time_9_eat_m___1 20:00-20:15<br>2 weai_time_9_eat_m___2 20:15-20:30<br>3 weai_time_9_eat_m___3 20:30-20:45<br>4 weai_time_9_eat_m___4 20:45-21:00<br>5 weai_time_9_eat_m___5 21:00-21:15<br>6 weai_time_9_eat_m___6 21:15-21:30<br>7 weai_time_9_eat_m___7 21:30-21:45<br>8 weai_time_9_eat_m___8 21:45-22:00
1539	weai_time_9_personal_m	Personal Care	checkbox<br>1 weai_time_9_personal_m___1 20:00-20:15<br>2 weai_time_9_personal_m___2 20:15-20:30<br>3 weai_time_9_personal_m___3 20:30-20:45<br>4 weai_time_9_personal_m___4 20:45-21:00<br>5 weai_time_9_personal_m___5 21:00-21:15<br>6 weai_time_9_personal_m___6 21:15-21:30<br>7 weai_time_9_personal_m___7 21:30-21:45<br>8 weai_time_9_personal_m___8 21:45-22:00
1540	weai_time_9_school_m	School (also homework)	checkbox<br>1 weai_time_9_school_m___1 20:00-20:15<br>2 weai_time_9_school_m___2 20:15-20:30<br>3 weai_time_9_school_m___3 20:30-20:45<br>4 weai_time_9_school_m___4 20:45-21:00<br>5 weai_time_9_school_m___5 21:00-21:15<br>6 weai_time_9_school_m___6 21:15-21:30<br>7 weai_time_9_school_m___7 21:30-21:45<br>8 weai_time_9_school_m___8 21:45-22:00

1541	weai_time_9_work_m	Work as employed	checkbox<br>1 weai_time_9_work_m___1 20:00-20:15<br>2 weai_time_9_work_m___2 20:15-20:30<br>3 weai_time_9_work_m___3 20:30-20:45<br>4 weai_time_9_work_m___4 20:45-21:00<br>5 weai_time_9_work_m___5 21:00-21:15<br>6 weai_time_9_work_m___6 21:15-21:30<br>7 weai_time_9_work_m___7 21:30-21:45<br>8 weai_time_9_work_m___8 21:45-22:00
1542	weai_time_9_buisness_m	Own business work	checkbox<br>1 weai_time_9_buisness_m___1 20:00-20:15<br>2 weai_time_9_buisness_m___2 20:15-20:30<br>3 weai_time_9_buisness_m___3 20:30-20:45<br>4 weai_time_9_buisness_m___4 20:45-21:00<br>5 weai_time_9_buisness_m___5 21:00-21:15<br>6 weai_time_9_buisness_m___6 21:15-21:30<br>7 weai_time_9_buisness_m___7 21:30-21:45<br>8 weai_time_9_buisness_m___8 21:45-22:00
1543	weai_time_9_farm_m	Farming/livestock/fishing	checkbox<br>1 weai_time_9_farm_m___1 20:00-20:15<br>2 weai_time_9_farm_m___2 20:15-20:30<br>3 weai_time_9_farm_m___3 20:30-20:45<br>4 weai_time_9_farm_m___4 20:45-21:00<br>5 weai_time_9_farm_m___5 21:00-21:15<br>6 weai_time_9_farm_m___6 21:15-21:30<br>7 weai_time_9_farm_m___7 21:30-21:45<br>8 weai_time_9_farm_m___8 21:45-22:00
1544	weai_time_9_shop_m	Shopping/getting service (incl health services)	checkbox<br>1 weai_time_9_shop_m___1 20:00-20:15<br>2 weai_time_9_shop_m___2 20:15-20:30<br>3 weai_time_9_shop_m___3 20:30-20:45<br>4 weai_time_9_shop_m___4 20:45-21:00<br>5 weai_time_9_shop_m___5 21:00-21:15<br>6 weai_time_9_shop_m___6 21:15-21:30<br>7 weai_time_9_shop_m___7 21:30-21:45<br>8 weai_time_9_shop_m___8 21:45-22:00
1545	weai_time_9_weave_m	Weaving, sewing, textile care	checkbox<br>1 weai_time_9_weave_m___1 20:00-20:15<br>2 weai_time_9_weave_m___2 20:15-20:30<br>3 weai_time_9_weave_m___3 20:30-20:45<br>4 weai_time_9_weave_m___4 20:45-21:00<br>5 weai_time_9_weave_m___5 21:00-21:15<br>6 weai_time_9_weave_m___6 21:15-21:30<br>7 weai_time_9_weave_m___7 21:30-21:45<br>8 weai_time_9_weave_m___8 21:45-22:00

Havelaar AH, *et al. BMJ Open* 2022; 12:e061311. doi: 10.1136/bmjopen-2022-061311

1546	weai_time_9_cook_m	Cooking	checkbox<br>1 weai_time_9_cook_m___1 20:00-20:15<br>2 weai_time_9_cook_m___2 20:15-20:30<br>3 weai_time_9_cook_m___3 20:30-20:45<br>4 weai_time_9_cook_m___4 20:45-21:00<br>5 weai_time_9_cook_m___5 21:00-21:15<br>6 weai_time_9_cook_m___6 21:15-21:30<br>7 weai_time_9_cook_m___7 21:30-21:45<br>8 weai_time_9_cook_m___8 21:45-22:00
1547	weai_time_9_domestic_m	Domestic work (incl fetching wood and water)	checkbox<br>1 weai_time_9_domestic_m___1 20:00-20:15<br>2 weai_time_9_domestic_m___2 20:15-20:30<br>3 weai_time_9_domestic_m___3 20:30-20:45<br>4 weai_time_9_domestic_m___4 20:45-21:00<br>5 weai_time_9_domestic_m___5 21:00-21:15<br>6 weai_time_9_domestic_m___6 21:15-21:30<br>7 weai_time_9_domestic_m___7 21:30-21:45<br>8 weai_time_9_domestic_m___8 21:45-22:00
1548	weai_time_9_care_m	Care for children/adults/elderly	checkbox<br>1 weai_time_9_care_m___1 20:00-20:15<br>2 weai_time_9_care_m___2 20:15-20:30<br>3 weai_time_9_care_m___3 20:30-20:45<br>4 weai_time_9_care_m___4 20:45-21:00<br>5 weai_time_9_care_m___5 21:00-21:15<br>6 weai_time_9_care_m___6 21:15-21:30<br>7 weai_time_9_care_m___7 21:30-21:45<br>8 weai_time_9_care_m___8 21:45-22:00
1549	weai_time_9_travel_m	Travelling and communiting	checkbox<br>1 weai_time_9_travel_m___1 20:00-20:15<br>2 weai_time_9_travel_m___2 20:15-20:30<br>3 weai_time_9_travel_m___3 20:30-20:45<br>4 weai_time_9_travel_m___4 20:45-21:00<br>5 weai_time_9_travel_m___5 21:00-21:15<br>6 weai_time_9_travel_m___6 21:15-21:30<br>7 weai_time_9_travel_m___7 21:30-21:45<br>8 weai_time_9_travel_m___8 21:45-22:00
1550	weai_time_9_tv_m	Watching TV/listening to radio/reading	checkbox<br>1 weai_time_9_tv_m___1 20:00-20:15<br>2 weai_time_9_tv_m___2 20:15-20:30<br>3 weai_time_9_tv_m___3 20:30-20:45<br>4 weai_time_9_tv_m___4 20:45-21:00<br>5 weai_time_9_tv_m___5 21:00-21:15<br>6 weai_time_9_tv_m___6 21:15-21:30<br>7 weai_time_9_tv_m___7 21:30-21:45<br>8 weai_time_9_tv_m___8 21:45-22:00

1551	weai_time_9_exercising_m	Exercising	checkbox<br>1 weai_time_9_exercising_m___1 20:00-20:15<br>2 weai_time_9_exercising_m___2 20:15-20:30<br>3 weai_time_9_exercising_m___3 20:30-20:45<br>4 weai_time_9_exercising_m___4 20:45-21:00<br>5 weai_time_9_exercising_m___5 21:00-21:15<br>6 weai_time_9_exercising_m___6 21:15-21:30<br>7 weai_time_9_exercising_m___7 21:30-21:45<br>8 weai_time_9_exercising_m___8 21:45-22:00
1552	weai_time_9_social_m	Social activities and hobbies	checkbox<br>1 weai_time_9_social_m___1 20:00-20:15<br>2 weai_time_9_social_m___2 20:15-20:30<br>3 weai_time_9_social_m___3 20:30-20:45<br>4 weai_time_9_social_m___4 20:45-21:00<br>5 weai_time_9_social_m___5 21:00-21:15<br>6 weai_time_9_social_m___6 21:15-21:30<br>7 weai_time_9_social_m___7 21:30-21:45<br>8 weai_time_9_social_m___8 21:45-22:00
1553	weai_time_9_religious_m	Religious activities	checkbox<br>1 weai_time_9_religious_m___1 20:00-20:15<br>2 weai_time_9_religious_m___2 20:15-20:30<br>3 weai_time_9_religious_m___3 20:30-20:45<br>4 weai_time_9_religious_m___4 20:45-21:00<br>5 weai_time_9_religious_m___5 21:00-21:15<br>6 weai_time_9_religious_m___6 21:15-21:30<br>7 weai_time_9_religious_m___7 21:30-21:45<br>8 weai_time_9_religious_m___8 21:45-22:00
1554	weai_time_9_other_m	Other (specify below)	checkbox<br>1 weai_time_9_other_m___1 20:00-20:15<br>2 weai_time_9_other_m___2 20:15-20:30<br>3 weai_time_9_other_m___3 20:30-20:45<br>4 weai_time_9_other_m___4 20:45-21:00<br>5 weai_time_9_other_m___5 21:00-21:15<br>6 weai_time_9_other_m___6 21:15-21:30<br>7 weai_time_9_other_m___7 21:30-21:45<br>8 weai_time_9_other_m___8 21:45-22:00
1555	weai_time_9_other_specify_m<br>Show the field ONLY if:<br>[weai_time_9_other_m(1)] or<br>[weai_time_9_other_m(2)] or<br>[weai_time_9_other_m(3)] or<br>[weai_time_9_other_m(4)] or<br>[weai_time_9_other_m(5)] or<br>[weai_time_9_other_m(6)] or<br>[weai_time_9_other_m(7)] or<br>[weai_time_9_other_m(8)]	Specify Other	text<br>Custom alignment: LV

1556	weai_time_10_sleep_m	Section Header: *Night/Early morning*<br>Sleeping and resting	checkbox<br>1 weai_time_10_sleep_m___1 22:00-22:15<br>2 weai_time_10_sleep_m___2 22:15-22:30<br>3 weai_time_10_sleep_m___3 22:30-22:45<br>4 weai_time_10_sleep_m___4 22:45-23:00<br>5 weai_time_10_sleep_m___5 23:00-23:15<br>6 weai_time_10_sleep_m___6 23:15-23:30<br>7 weai_time_10_sleep_m___7 23:30-23:45<br>8 weai_time_10_sleep_m___8 23:45-00:00
1557	weai_time_10_eat_m	Eating and drinking	checkbox<br>1 weai_time_10_eat_m___1 22:00-22:15<br>2 weai_time_10_eat_m___2 22:15-22:30<br>3 weai_time_10_eat_m___3 22:30-22:45<br>4 weai_time_10_eat_m___4 22:45-23:00<br>5 weai_time_10_eat_m___5 23:00-23:15<br>6 weai_time_10_eat_m___6 23:15-23:30<br>7 weai_time_10_eat_m___7 23:30-23:45<br>8 weai_time_10_eat_m___8 23:45-00:00
1558	weai_time_10_personal_m	Personal Care	checkbox<br>1 weai_time_10_personal_m___1 22:00-22:15<br>2 weai_time_10_personal_m___2 22:15-22:30<br>3 weai_time_10_personal_m___3 22:30-22:45<br>4 weai_time_10_personal_m___4 22:45-23:00<br>5 weai_time_10_personal_m___5 23:00-23:15<br>6 weai_time_10_personal_m___6 23:15-23:30<br>7 weai_time_10_personal_m___7 23:30-23:45<br>8 weai_time_10_personal_m___8 23:45-00:00
1559	weai_time_10_school_m	School (also homework)	checkbox<br>1 weai_time_10_school_m___1 22:00-22:15<br>2 weai_time_10_school_m___2 22:15-22:30<br>3 weai_time_10_school_m___3 22:30-22:45<br>4 weai_time_10_school_m___4 22:45-23:00<br>5 weai_time_10_school_m___5 23:00-23:15<br>6 weai_time_10_school_m___6 23:15-23:30<br>7 weai_time_10_school_m___7 23:30-23:45<br>8 weai_time_10_school_m___8 23:45-00:00
1560	weai_time_10_work_m	Work as employed	checkbox<br>1 weai_time_10_work_m___1 22:00-22:15<br>2 weai_time_10_work_m___2 22:15-22:30<br>3 weai_time_10_work_m___3 22:30-22:45<br>4 weai_time_10_work_m___4 22:45-23:00<br>5 weai_time_10_work_m___5 23:00-23:15<br>6 weai_time_10_work_m___6 23:15-23:30<br>7 weai_time_10_work_m___7 23:30-23:45<br>8 weai_time_10_work_m___8 23:45-00:00

Havelaar AH, *et al. BMJ Open* 2022; 12:e061311. doi: 10.1136/bmjopen-2022-061311

Supplemental material BMJ Publishing Group Limited (BMJ) disclaims all liability and responsibility arising from any reliance placed on this supplemental material which has been supplied by the author(s) BMJ Open

1561	weai_time_10_buisness_m	Own business work	checkbox<br>1 | weai_time_10_buisness_m___1 | 22:00-22:15<br>2 | weai_time_10_buisness_m___2 | 22:15-22:30<br>3 | weai_time_10_buisness_m___3 | 22:30-22:45<br>4 | weai_time_10_buisness_m___4 | 22:45-23:00<br>5 | weai_time_10_buisness_m___5 | 23:00-23:15<br>6 | weai_time_10_buisness_m___6 | 23:15-23:30<br>7 | weai_time_10_buisness_m___7 | 23:30-23:45<br>8 | weai_time_10_buisness_m___8 | 23:45-00:00
1562	weai_time_10_farm_m	Farming/livestock/fishing	checkbox<br>1 | weai_time_10_farm_m___1 | 22:00-22:15<br>2 | weai_time_10_farm_m___2 | 22:15-22:30<br>3 | weai_time_10_farm_m___3 | 22:30-22:45<br>4 | weai_time_10_farm_m___4 | 22:45-23:00<br>5 | weai_time_10_farm_m___5 | 23:00-23:15<br>6 | weai_time_10_farm_m___6 | 23:15-23:30<br>7 | weai_time_10_farm_m___7 | 23:30-23:45<br>8 | weai_time_10_farm_m___8 | 23:45-00:00
1563	weai_time_10_shop_m	Shopping/getting service (incl health services)	checkbox<br>1 | weai_time_10_shop_m___1 | 22:00-22:15<br>2 | weai_time_10_shop_m___2 | 22:15-22:30<br>3 | weai_time_10_shop_m___3 | 22:30-22:45<br>4 | weai_time_10_shop_m___4 | 22:45-23:00<br>5 | weai_time_10_shop_m___5 | 23:00-23:15<br>6 | weai_time_10_shop_m___6 | 23:15-23:30<br>7 | weai_time_10_shop_m___7 | 23:30-23:45<br>8 | weai_time_10_shop_m___8 | 23:45-00:00
1564	weai_time_10_weave_m	Weaving, sewing, textile care	checkbox<br>1 | weai_time_10_weave_m___1 | 22:00-22:15<br>2 | weai_time_10_weave_m___2 | 22:15-22:30<br>3 | weai_time_10_weave_m___3 | 22:30-22:45<br>4 | weai_time_10_weave_m___4 | 22:45-23:00<br>5 | weai_time_10_weave_m___5 | 23:00-23:15<br>6 | weai_time_10_weave_m___6 | 23:15-23:30<br>7 | weai_time_10_weave_m___7 | 23:30-23:45<br>8 | weai_time_10_weave_m___8 | 23:45-00:00
1565	weai_time_10_cook_m	Cooking	checkbox<br>1 | weai_time_10_cook_m___1 | 22:00-22:15<br>2 | weai_time_10_cook_m___2 | 22:15-22:30<br>3 | weai_time_10_cook_m___3 | 22:30-22:45<br>4 | weai_time_10_cook_m___4 | 22:45-23:00<br>5 | weai_time_10_cook_m___5 | 23:00-23:15<br>6 | weai_time_10_cook_m___6 | 23:15-23:30<br>7 | weai_time_10_cook_m___7 | 23:30-23:45<br>8 | weai_time_10_cook_m___8 | 23:45-00:00

Havelaar AH, *et al. BMJ Open* 2022; 12:e061311. doi: 10.1136/bmjopen-2022-061311

1566	weai_time_10_domestic_m	Domestic work (incl fetching wood and water)	checkbox<br>1 weai_time_10_domestic_m___1 22:00-22:15<br>2 weai_time_10_domestic_m___2 22:15-22:30<br>3 weai_time_10_domestic_m___3 22:30-22:45<br>4 weai_time_10_domestic_m___4 22:45-23:00<br>5 weai_time_10_domestic_m___5 23:00-23:15<br>6 weai_time_10_domestic_m___6 23:15-23:30<br>7 weai_time_10_domestic_m___7 23:30-23:45<br>8 weai_time_10_domestic_m___8 23:45-00:00
1567	weai_time_10_care_m	Care for children/adults/elderly	checkbox<br>1 weai_time_10_care_m___1 22:00-22:15<br>2 weai_time_10_care_m___2 22:15-22:30<br>3 weai_time_10_care_m___3 22:30-22:45<br>4 weai_time_10_care_m___4 22:45-23:00<br>5 weai_time_10_care_m___5 23:00-23:15<br>6 weai_time_10_care_m___6 23:15-23:30<br>7 weai_time_10_care_m___7 23:30-23:45<br>8 weai_time_10_care_m___8 23:45-00:00
1568	weai_time_10_travel_m	Travelling and communiting	checkbox<br>1 weai_time_10_travel_m___1 22:00-22:15<br>2 weai_time_10_travel_m___2 22:15-22:30<br>3 weai_time_10_travel_m___3 22:30-22:45<br>4 weai_time_10_travel_m___4 22:45-23:00<br>5 weai_time_10_travel_m___5 23:00-23:15<br>6 weai_time_10_travel_m___6 23:15-23:30<br>7 weai_time_10_travel_m___7 23:30-23:45<br>8 weai_time_10_travel_m___8 23:45-00:00
1569	weai_time_10_tv_m	Watching TV/listening to radio/reading	checkbox<br>1 weai_time_10_tv_m___1 22:00-22:15<br>2 weai_time_10_tv_m___2 22:15-22:30<br>3 weai_time_10_tv_m___3 22:30-22:45<br>4 weai_time_10_tv_m___4 22:45-23:00<br>5 weai_time_10_tv_m___5 23:00-23:15<br>6 weai_time_10_tv_m___6 23:15-23:30<br>7 weai_time_10_tv_m___7 23:30-23:45<br>8 weai_time_10_tv_m___8 23:45-00:00
1570	weai_time_10_exercising_m	Exercising	checkbox<br>1 weai_time_10_exercising_m___1 22:00-22:15<br>2 weai_time_10_exercising_m___2 22:15-22:30<br>3 weai_time_10_exercising_m___3 22:30-22:45<br>4 weai_time_10_exercising_m___4 22:45-23:00<br>5 weai_time_10_exercising_m___5 23:00-23:15<br>6 weai_time_10_exercising_m___6 23:15-23:30<br>7 weai_time_10_exercising_m___7 23:30-23:45<br>8 weai_time_10_exercising_m___8 23:45-00:00

1571	weai_time_10_social_m	Social activities and hobbies	checkbox<br>1 weai_time_10_social_m___1 22:00-22:15<br>2 weai_time_10_social_m___2 22:15-22:30<br>3 weai_time_10_social_m___3 22:30-22:45<br>4 weai_time_10_social_m___4 22:45-23:00<br>5 weai_time_10_social_m___5 23:00-23:15<br>6 weai_time_10_social_m___6 23:15-23:30<br>7 weai_time_10_social_m___7 23:30-23:45<br>8 weai_time_10_social_m___8 23:45-00:00
1572	weai_time_10_religious_m	Religious activities	checkbox<br>1 weai_time_10_religious_m___1 22:00-22:15<br>2 weai_time_10_religious_m___2 22:15-22:30<br>3 weai_time_10_religious_m___3 22:30-22:45<br>4 weai_time_10_religious_m___4 22:45-23:00<br>5 weai_time_10_religious_m___5 23:00-23:15<br>6 weai_time_10_religious_m___6 23:15-23:30<br>7 weai_time_10_religious_m___7 23:30-23:45<br>8 weai_time_10_religious_m___8 23:45-00:00
1573	weai_time_10_other_m	Other (specify below)	checkbox<br>1 weai_time_10_other_m___1 22:00-22:15<br>2 weai_time_10_other_m___2 22:15-22:30<br>3 weai_time_10_other_m___3 22:30-22:45<br>4 weai_time_10_other_m___4 22:45-23:00<br>5 weai_time_10_other_m___5 23:00-23:15<br>6 weai_time_10_other_m___6 23:15-23:30<br>7 weai_time_10_other_m___7 23:30-23:45<br>8 weai_time_10_other_m___8 23:45-00:00
1574	weai_time_10_other_specify_m<br>Show the field ONLY if:<br>[weai_time_10_other_m(1)] or [weai_time_10_other_m(2)] or [weai_time_10_other_m(3)] or [weai_time_10_other_m(4)] or [weai_time_10_other_m(5)] or [weai_time_10_other_m(6)] or [weai_time_10_other_m(7)] or [weai_time_10_other_m(8)]	Specify Other	text<br>Custom alignment: LV
1575	weai_time_11_sleep_m	Section Header: *Night/Early morning*<br>Sleeping and resting	checkbox<br>1 weai_time_11_sleep_m___1 00:00-00:15<br>2 weai_time_11_sleep_m___2 00:15-00:30<br>3 weai_time_11_sleep_m___3 00:30-00:45<br>4 weai_time_11_sleep_m___4 00:45-01:00<br>5 weai_time_11_sleep_m___5 01:00-01:15<br>6 weai_time_11_sleep_m___6 01:15-01:30<br>7 weai_time_11_sleep_m___7 01:30-01:45<br>8 weai_time_11_sleep_m___8 01:45-02:00

Havelaar AH, *et al. BMJ Open* 2022; 12:e061311. doi: 10.1136/bmjopen-2022-061311

Supplemental material BMJ Publishing Group Limited (BMJ) disclaims all liability and responsibility arising from any reliance placed on this supplemental material which has been supplied by the author(s) BMJ Open

1576	weai_time_11_eat_m	Eating and drinking	checkbox<br>1 weai_time_11_eat_m___1 00:00-00:15<br>2 weai_time_11_eat_m___2 00:15-00:30<br>3 weai_time_11_eat_m___3 00:30-00:45<br>4 weai_time_11_eat_m___4 00:45-01:00<br>5 weai_time_11_eat_m___5 01:00-01:15<br>6 weai_time_11_eat_m___6 01:15-01:30<br>7 weai_time_11_eat_m___7 01:30-01:45<br>8 weai_time_11_eat_m___8 01:45-02:00
1577	weai_time_11_personal_m	Personal Care	checkbox<br>1 weai_time_11_personal_m___1 00:00-00:15<br>2 weai_time_11_personal_m___2 00:15-00:30<br>3 weai_time_11_personal_m___3 00:30-00:45<br>4 weai_time_11_personal_m___4 00:45-01:00<br>5 weai_time_11_personal_m___5 01:00-01:15<br>6 weai_time_11_personal_m___6 01:15-01:30<br>7 weai_time_11_personal_m___7 01:30-01:45<br>8 weai_time_11_personal_m___8 01:45-02:00
1578	weai_time_11_school_m	School (also homework)	checkbox<br>1 weai_time_11_school_m___1 00:00-00:15<br>2 weai_time_11_school_m___2 00:15-00:30<br>3 weai_time_11_school_m___3 00:30-00:45<br>4 weai_time_11_school_m___4 00:45-01:00<br>5 weai_time_11_school_m___5 01:00-01:15<br>6 weai_time_11_school_m___6 01:15-01:30<br>7 weai_time_11_school_m___7 01:30-01:45<br>8 weai_time_11_school_m___8 01:45-02:00
1579	weai_time_11_work_m	Work as employed	checkbox<br>1 weai_time_11_work_m___1 00:00-00:15<br>2 weai_time_11_work_m___2 00:15-00:30<br>3 weai_time_11_work_m___3 00:30-00:45<br>4 weai_time_11_work_m___4 00:45-01:00<br>5 weai_time_11_work_m___5 01:00-01:15<br>6 weai_time_11_work_m___6 01:15-01:30<br>7 weai_time_11_work_m___7 01:30-01:45<br>8 weai_time_11_work_m___8 01:45-02:00
1580	weai_time_11_buisness_m	Own business work	checkbox<br>1 weai_time_11_buisness_m___1 00:00-00:15<br>2 weai_time_11_buisness_m___2 00:15-00:30<br>3 weai_time_11_buisness_m___3 00:30-00:45<br>4 weai_time_11_buisness_m___4 00:45-01:00<br>5 weai_time_11_buisness_m___5 01:00-01:15<br>6 weai_time_11_buisness_m___6 01:15-01:30<br>7 weai_time_11_buisness_m___7 01:30-01:45<br>8 weai_time_11_buisness_m___8 01:45-02:00

Havelaar AH, *et al. BMJ Open* 2022; 12:e061311. doi: 10.1136/bmjopen-2022-061311

1581	weai_time_11_farm_m	Farming/livestock/fishing	checkbox<br>1 | weai_time_11_farm_m___1 | 00:00-00:15<br>2 | weai_time_11_farm_m___2 | 00:15-00:30<br>3 | weai_time_11_farm_m___3 | 00:30-00:45<br>4 | weai_time_11_farm_m___4 | 00:45-01:00<br>5 | weai_time_11_farm_m___5 | 01:00-01:15<br>6 | weai_time_11_farm_m___6 | 01:15-01:30<br>7 | weai_time_11_farm_m___7 | 01:30-01:45<br>8 | weai_time_11_farm_m___8 | 01:45-02:00
1582	weai_time_11_shop_m	Shopping/getting service (incl health services)	checkbox<br>1 | weai_time_11_shop_m___1 | 00:00-00:15<br>2 | weai_time_11_shop_m___2 | 00:15-00:30<br>3 | weai_time_11_shop_m___3 | 00:30-00:45<br>4 | weai_time_11_shop_m___4 | 00:45-01:00<br>5 | weai_time_11_shop_m___5 | 01:00-01:15<br>6 | weai_time_11_shop_m___6 | 01:15-01:30<br>7 | weai_time_11_shop_m___7 | 01:30-01:45<br>8 | weai_time_11_shop_m___8 | 01:45-02:00
1583	weai_time_11_weave_m	Weaving, sewing, textile care	checkbox<br>1 | weai_time_11_weave_m___1 | 00:00-00:15<br>2 | weai_time_11_weave_m___2 | 00:15-00:30<br>3 | weai_time_11_weave_m___3 | 00:30-00:45<br>4 | weai_time_11_weave_m___4 | 00:45-01:00<br>5 | weai_time_11_weave_m___5 | 01:00-01:15<br>6 | weai_time_11_weave_m___6 | 01:15-01:30<br>7 | weai_time_11_weave_m___7 | 01:30-01:45<br>8 | weai_time_11_weave_m___8 | 01:45-02:00
1584	weai_time_11_cook_m	Cooking	checkbox<br>1 | weai_time_11_cook_m___1 | 00:00-00:15<br>2 | weai_time_11_cook_m___2 | 00:15-00:30<br>3 | weai_time_11_cook_m___3 | 00:30-00:45<br>4 | weai_time_11_cook_m___4 | 00:45-01:00<br>5 | weai_time_11_cook_m___5 | 01:00-01:15<br>6 | weai_time_11_cook_m___6 | 01:15-01:30<br>7 | weai_time_11_cook_m___7 | 01:30-01:45<br>8 | weai_time_11_cook_m___8 | 01:45-02:00
1585	weai_time_11_domestic_m	Domestic work (incl fetching wood and water)	checkbox<br>1 | weai_time_11_domestic_m___1 | 00:00-00:15<br>2 | weai_time_11_domestic_m___2 | 00:15-00:30<br>3 | weai_time_11_domestic_m___3 | 00:30-00:45<br>4 | weai_time_11_domestic_m___4 | 00:45-01:00<br>5 | weai_time_11_domestic_m___5 | 01:00-01:15<br>6 | weai_time_11_domestic_m___6 | 01:15-01:30<br>7 | weai_time_11_domestic_m___7 | 01:30-01:45<br>8 | weai_time_11_domestic_m___8 | 01:45-02:00

<table>
<tr><td>1586</td><td>weai_time_11_care_m</td><td>Care for children/adults/elderly</td><td>checkbox<table>
<tr><td>1</td><td>weai_time_11_care_m___1</td><td>00:00-00:15</td></tr>
<tr><td>2</td><td>weai_time_11_care_m___2</td><td>00:15-00:30</td></tr>
<tr><td>3</td><td>weai_time_11_care_m___3</td><td>00:30-00:45</td></tr>
<tr><td>4</td><td>weai_time_11_care_m___4</td><td>00:45-01:00</td></tr>
<tr><td>5</td><td>weai_time_11_care_m___5</td><td>01:00-01:15</td></tr>
<tr><td>6</td><td>weai_time_11_care_m___6</td><td>01:15-01:30</td></tr>
<tr><td>7</td><td>weai_time_11_care_m___7</td><td>01:30-01:45</td></tr>
<tr><td>8</td><td>weai_time_11_care_m___8</td><td>01:45-02:00</td></tr>
</table></td></tr>
<tr><td>1587</td><td>weai_time_11_travel_m</td><td>Travelling and communiting</td><td>checkbox<table>
<tr><td>1</td><td>weai_time_11_travel_m___1</td><td>00:00-00:15</td></tr>
<tr><td>2</td><td>weai_time_11_travel_m___2</td><td>00:15-00:30</td></tr>
<tr><td>3</td><td>weai_time_11_travel_m___3</td><td>00:30-00:45</td></tr>
<tr><td>4</td><td>weai_time_11_travel_m___4</td><td>00:45-01:00</td></tr>
<tr><td>5</td><td>weai_time_11_travel_m___5</td><td>01:00-01:15</td></tr>
<tr><td>6</td><td>weai_time_11_travel_m___6</td><td>01:15-01:30</td></tr>
<tr><td>7</td><td>weai_time_11_travel_m___7</td><td>01:30-01:45</td></tr>
<tr><td>8</td><td>weai_time_11_travel_m___8</td><td>01:45-02:00</td></tr>
</table></td></tr>
<tr><td>1588</td><td>weai_time_11_tv_m</td><td>Watching TV/listening to radio/reading</td><td>checkbox<table>
<tr><td>1</td><td>weai_time_11_tv_m___1</td><td>00:00-00:15</td></tr>
<tr><td>2</td><td>weai_time_11_tv_m___2</td><td>00:15-00:30</td></tr>
<tr><td>3</td><td>weai_time_11_tv_m___3</td><td>00:30-00:45</td></tr>
<tr><td>4</td><td>weai_time_11_tv_m___4</td><td>00:45-01:00</td></tr>
<tr><td>5</td><td>weai_time_11_tv_m___5</td><td>01:00-01:15</td></tr>
<tr><td>6</td><td>weai_time_11_tv_m___6</td><td>01:15-01:30</td></tr>
<tr><td>7</td><td>weai_time_11_tv_m___7</td><td>01:30-01:45</td></tr>
<tr><td>8</td><td>weai_time_11_tv_m___8</td><td>01:45-02:00</td></tr>
</table></td></tr>
<tr><td>1589</td><td>weai_time_11_exercising_m</td><td>Exercising</td><td>checkbox<table>
<tr><td>1</td><td>weai_time_11_exercising_m___1</td><td>00:00-00:15</td></tr>
<tr><td>2</td><td>weai_time_11_exercising_m___2</td><td>00:15-00:30</td></tr>
<tr><td>3</td><td>weai_time_11_exercising_m___3</td><td>00:30-00:45</td></tr>
<tr><td>4</td><td>weai_time_11_exercising_m___4</td><td>00:45-01:00</td></tr>
<tr><td>5</td><td>weai_time_11_exercising_m___5</td><td>01:00-01:15</td></tr>
<tr><td>6</td><td>weai_time_11_exercising_m___6</td><td>01:15-01:30</td></tr>
<tr><td>7</td><td>weai_time_11_exercising_m___7</td><td>01:30-01:45</td></tr>
<tr><td>8</td><td>weai_time_11_exercising_m___8</td><td>01:45-02:00</td></tr>
</table></td></tr>
<tr><td>1590</td><td>weai_time_11_social_m</td><td>Social activities and hobbies</td><td>checkbox<table>
<tr><td>1</td><td>weai_time_11_social_m___1</td><td>00:00-00:15</td></tr>
<tr><td>2</td><td>weai_time_11_social_m___2</td><td>00:15-00:30</td></tr>
<tr><td>3</td><td>weai_time_11_social_m___3</td><td>00:30-00:45</td></tr>
<tr><td>4</td><td>weai_time_11_social_m___4</td><td>00:45-01:00</td></tr>
<tr><td>5</td><td>weai_time_11_social_m___5</td><td>01:00-01:15</td></tr>
<tr><td>6</td><td>weai_time_11_social_m___6</td><td>01:15-01:30</td></tr>
<tr><td>7</td><td>weai_time_11_social_m___7</td><td>01:30-01:45</td></tr>
<tr><td>8</td><td>weai_time_11_social_m___8</td><td>01:45-02:00</td></tr>
</table></td></tr>
</table>

1591	weai_time_11_religious_m	Religious activities	checkbox<br>1 weai_time_11_religious_m___1 00:00-00:15<br>2 weai_time_11_religious_m___2 00:15-00:30<br>3 weai_time_11_religious_m___3 00:30-00:45<br>4 weai_time_11_religious_m___4 00:45-01:00<br>5 weai_time_11_religious_m___5 01:00-01:15<br>6 weai_time_11_religious_m___6 01:15-01:30<br>7 weai_time_11_religious_m___7 01:30-01:45<br>8 weai_time_11_religious_m___8 01:45-02:00
1592	weai_time_11_other_m	Other (specify below)	checkbox<br>1 weai_time_11_other_m___1 00:00-00:15<br>2 weai_time_11_other_m___2 00:15-00:30<br>3 weai_time_11_other_m___3 00:30-00:45<br>4 weai_time_11_other_m___4 00:45-01:00<br>5 weai_time_11_other_m___5 01:00-01:15<br>6 weai_time_11_other_m___6 01:15-01:30<br>7 weai_time_11_other_m___7 01:30-01:45<br>8 weai_time_11_other_m___8 01:45-02:00
1593	weai_time_11_other_specify_m<br>Show the field ONLY if:<br>[weai_time_11_other_m(1)] or<br>[weai_time_11_other_m(2)] or<br>[weai_time_11_other_m(3)] or<br>[weai_time_11_other_m(4)] or<br>[weai_time_11_other_m(5)] or<br>[weai_time_11_other_m(6)] or<br>[weai_time_11_other_m(7)] or<br>[weai_time_11_other_m(8)]	Specify Other	text<br>Custom alignment: LV
1594	weai_time_12_sleep_m	Section Header: *Night/Early morning*<br>Sleeping and resting	checkbox<br>1 weai_time_12_sleep_m___1 02:00-02:15<br>2 weai_time_12_sleep_m___2 02:15-02:30<br>3 weai_time_12_sleep_m___3 02:30-02:45<br>4 weai_time_12_sleep_m___4 02:45-03:00<br>5 weai_time_12_sleep_m___5 03:00-03:15<br>6 weai_time_12_sleep_m___6 03:15-03:30<br>7 weai_time_12_sleep_m___7 03:30-03:45<br>8 weai_time_12_sleep_m___8 03:45-04:00
1595	weai_time_12_eat_m	Eating and drinking	checkbox<br>1 weai_time_12_eat_m___1 02:00-02:15<br>2 weai_time_12_eat_m___2 02:15-02:30<br>3 weai_time_12_eat_m___3 02:30-02:45<br>4 weai_time_12_eat_m___4 02:45-03:00<br>5 weai_time_12_eat_m___5 03:00-03:15<br>6 weai_time_12_eat_m___6 03:15-03:30<br>7 weai_time_12_eat_m___7 03:30-03:45<br>8 weai_time_12_eat_m___8 03:45-04:00

1596	weai_time_12_personal_m	Personal Care	checkbox<br>1 weai_time_12_personal_m___1 02:00-02:15<br>2 weai_time_12_personal_m___2 02:15-02:30<br>3 weai_time_12_personal_m___3 02:30-02:45<br>4 weai_time_12_personal_m___4 02:45-03:00<br>5 weai_time_12_personal_m___5 03:00-03:15<br>6 weai_time_12_personal_m___6 03:15-03:30<br>7 weai_time_12_personal_m___7 03:30-03:45<br>8 weai_time_12_personal_m___8 03:45-04:00
1597	weai_time_12_school_m	School (also homework)	checkbox<br>1 weai_time_12_school_m___1 02:00-02:15<br>2 weai_time_12_school_m___2 02:15-02:30<br>3 weai_time_12_school_m___3 02:30-02:45<br>4 weai_time_12_school_m___4 02:45-03:00<br>5 weai_time_12_school_m___5 03:00-03:15<br>6 weai_time_12_school_m___6 03:15-03:30<br>7 weai_time_12_school_m___7 03:30-03:45<br>8 weai_time_12_school_m___8 03:45-04:00
1598	weai_time_12_work_m	Work as employed	checkbox<br>1 weai_time_12_work_m___1 02:00-02:15<br>2 weai_time_12_work_m___2 02:15-02:30<br>3 weai_time_12_work_m___3 02:30-02:45<br>4 weai_time_12_work_m___4 02:45-03:00<br>5 weai_time_12_work_m___5 03:00-03:15<br>6 weai_time_12_work_m___6 03:15-03:30<br>7 weai_time_12_work_m___7 03:30-03:45<br>8 weai_time_12_work_m___8 03:45-04:00
1599	weai_time_12_buisness_m	Own business work	checkbox<br>1 weai_time_12_buisness_m___1 02:00-02:15<br>2 weai_time_12_buisness_m___2 02:15-02:30<br>3 weai_time_12_buisness_m___3 02:30-02:45<br>4 weai_time_12_buisness_m___4 02:45-03:00<br>5 weai_time_12_buisness_m___5 03:00-03:15<br>6 weai_time_12_buisness_m___6 03:15-03:30<br>7 weai_time_12_buisness_m___7 03:30-03:45<br>8 weai_time_12_buisness_m___8 03:45-04:00
1600	weai_time_12_farm_m	Farming/livestock/fishing	checkbox<br>1 weai_time_12_farm_m___1 02:00-02:15<br>2 weai_time_12_farm_m___2 02:15-02:30<br>3 weai_time_12_farm_m___3 02:30-02:45<br>4 weai_time_12_farm_m___4 02:45-03:00<br>5 weai_time_12_farm_m___5 03:00-03:15<br>6 weai_time_12_farm_m___6 03:15-03:30<br>7 weai_time_12_farm_m___7 03:30-03:45<br>8 weai_time_12_farm_m___8 03:45-04:00

1601	weai_time_12_shop_m	Shopping/getting service (incl health services)	checkbox<br>1 weai_time_12_shop_m___1 02:00-02:15<br>2 weai_time_12_shop_m___2 02:15-02:30<br>3 weai_time_12_shop_m___3 02:30-02:45<br>4 weai_time_12_shop_m___4 02:45-03:00<br>5 weai_time_12_shop_m___5 03:00-03:15<br>6 weai_time_12_shop_m___6 03:15-03:30<br>7 weai_time_12_shop_m___7 03:30-03:45<br>8 weai_time_12_shop_m___8 03:45-04:00
1602	weai_time_12_weave_m	Weaving, sewing, textile care	checkbox<br>1 weai_time_12_weave_m___1 02:00-02:15<br>2 weai_time_12_weave_m___2 02:15-02:30<br>3 weai_time_12_weave_m___3 02:30-02:45<br>4 weai_time_12_weave_m___4 02:45-03:00<br>5 weai_time_12_weave_m___5 03:00-03:15<br>6 weai_time_12_weave_m___6 03:15-03:30<br>7 weai_time_12_weave_m___7 03:30-03:45<br>8 weai_time_12_weave_m___8 03:45-04:00
1603	weai_time_12_cook_m	Cooking	checkbox<br>1 weai_time_12_cook_m___1 02:00-02:15<br>2 weai_time_12_cook_m___2 02:15-02:30<br>3 weai_time_12_cook_m___3 02:30-02:45<br>4 weai_time_12_cook_m___4 02:45-03:00<br>5 weai_time_12_cook_m___5 03:00-03:15<br>6 weai_time_12_cook_m___6 03:15-03:30<br>7 weai_time_12_cook_m___7 03:30-03:45<br>8 weai_time_12_cook_m___8 03:45-04:00
1604	weai_time_12_domestic_m	Domestic work (incl fetching wood and water)	checkbox<br>1 weai_time_12_domestic_m___1 02:00-02:15<br>2 weai_time_12_domestic_m___2 02:15-02:30<br>3 weai_time_12_domestic_m___3 02:30-02:45<br>4 weai_time_12_domestic_m___4 02:45-03:00<br>5 weai_time_12_domestic_m___5 03:00-03:15<br>6 weai_time_12_domestic_m___6 03:15-03:30<br>7 weai_time_12_domestic_m___7 03:30-03:45<br>8 weai_time_12_domestic_m___8 03:45-04:00
1605	weai_time_12_care_m	Care for children/adults/elderly	checkbox<br>1 weai_time_12_care_m___1 02:00-02:15<br>2 weai_time_12_care_m___2 02:15-02:30<br>3 weai_time_12_care_m___3 02:30-02:45<br>4 weai_time_12_care_m___4 02:45-03:00<br>5 weai_time_12_care_m___5 03:00-03:15<br>6 weai_time_12_care_m___6 03:15-03:30<br>7 weai_time_12_care_m___7 03:30-03:45<br>8 weai_time_12_care_m___8 03:45-04:00

Havelaar AH, *et al. BMJ Open* 2022; 12:e061311. doi: 10.1136/bmjopen-2022-061311

<table>
<tr><td>1606</td><td>weai_time_12_travel_m</td><td>Travelling and communiting</td><td>checkbox
<table>
<tr><td>1</td><td>weai_time_12_travel_m___1</td><td>02:00-02:15</td></tr>
<tr><td>2</td><td>weai_time_12_travel_m___2</td><td>02:15-02:30</td></tr>
<tr><td>3</td><td>weai_time_12_travel_m___3</td><td>02:30-02:45</td></tr>
<tr><td>4</td><td>weai_time_12_travel_m___4</td><td>02:45-03:00</td></tr>
<tr><td>5</td><td>weai_time_12_travel_m___5</td><td>03:00-03:15</td></tr>
<tr><td>6</td><td>weai_time_12_travel_m___6</td><td>03:15-03:30</td></tr>
<tr><td>7</td><td>weai_time_12_travel_m___7</td><td>03:30-03:45</td></tr>
<tr><td>8</td><td>weai_time_12_travel_m___8</td><td>03:45-04:00</td></tr>
</table></td></tr>
<tr><td>1607</td><td>weai_time_12_tv_m</td><td>Watching TV/listening to radio/reading</td><td>checkbox
<table>
<tr><td>1</td><td>weai_time_12_tv_m___1</td><td>02:00-02:15</td></tr>
<tr><td>2</td><td>weai_time_12_tv_m___2</td><td>02:15-02:30</td></tr>
<tr><td>3</td><td>weai_time_12_tv_m___3</td><td>02:30-02:45</td></tr>
<tr><td>4</td><td>weai_time_12_tv_m___4</td><td>02:45-03:00</td></tr>
<tr><td>5</td><td>weai_time_12_tv_m___5</td><td>03:00-03:15</td></tr>
<tr><td>6</td><td>weai_time_12_tv_m___6</td><td>03:15-03:30</td></tr>
<tr><td>7</td><td>weai_time_12_tv_m___7</td><td>03:30-03:45</td></tr>
<tr><td>8</td><td>weai_time_12_tv_m___8</td><td>03:45-04:00</td></tr>
</table></td></tr>
<tr><td>1608</td><td>weai_time_12_exercising_m</td><td>Exercising</td><td>checkbox
<table>
<tr><td>1</td><td>weai_time_12_exercising_m___1</td><td>02:00-02:15</td></tr>
<tr><td>2</td><td>weai_time_12_exercising_m___2</td><td>02:15-02:30</td></tr>
<tr><td>3</td><td>weai_time_12_exercising_m___3</td><td>02:30-02:45</td></tr>
<tr><td>4</td><td>weai_time_12_exercising_m___4</td><td>02:45-03:00</td></tr>
<tr><td>5</td><td>weai_time_12_exercising_m___5</td><td>03:00-03:15</td></tr>
<tr><td>6</td><td>weai_time_12_exercising_m___6</td><td>03:15-03:30</td></tr>
<tr><td>7</td><td>weai_time_12_exercising_m___7</td><td>03:30-03:45</td></tr>
<tr><td>8</td><td>weai_time_12_exercising_m___8</td><td>03:45-04:00</td></tr>
</table></td></tr>
<tr><td>1609</td><td>weai_time_12_social_m</td><td>Social activities and hobbies</td><td>checkbox
<table>
<tr><td>1</td><td>weai_time_12_social_m___1</td><td>02:00-02:15</td></tr>
<tr><td>2</td><td>weai_time_12_social_m___2</td><td>02:15-02:30</td></tr>
<tr><td>3</td><td>weai_time_12_social_m___3</td><td>02:30-02:45</td></tr>
<tr><td>4</td><td>weai_time_12_social_m___4</td><td>02:45-03:00</td></tr>
<tr><td>5</td><td>weai_time_12_social_m___5</td><td>03:00-03:15</td></tr>
<tr><td>6</td><td>weai_time_12_social_m___6</td><td>03:15-03:30</td></tr>
<tr><td>7</td><td>weai_time_12_social_m___7</td><td>03:30-03:45</td></tr>
<tr><td>8</td><td>weai_time_12_social_m___8</td><td>03:45-04:00</td></tr>
</table></td></tr>
<tr><td>1610</td><td>weai_time_12_religious_m</td><td>Religious activities</td><td>checkbox
<table>
<tr><td>1</td><td>weai_time_12_religious_m___1</td><td>02:00-02:15</td></tr>
<tr><td>2</td><td>weai_time_12_religious_m___2</td><td>02:15-02:30</td></tr>
<tr><td>3</td><td>weai_time_12_religious_m___3</td><td>02:30-02:45</td></tr>
<tr><td>4</td><td>weai_time_12_religious_m___4</td><td>02:45-03:00</td></tr>
<tr><td>5</td><td>weai_time_12_religious_m___5</td><td>03:00-03:15</td></tr>
<tr><td>6</td><td>weai_time_12_religious_m___6</td><td>03:15-03:30</td></tr>
<tr><td>7</td><td>weai_time_12_religious_m___7</td><td>03:30-03:45</td></tr>
<tr><td>8</td><td>weai_time_12_religious_m___8</td><td>03:45-04:00</td></tr>
</table></td></tr>
</table>

Havelaar AH, *et al. BMJ Open* 2022; 12:e061311. doi: 10.1136/bmjopen-2022-061311

1611	weai_time_12_other_m	Other (specify below)	checkbox<br>1 weai_time_12_other_m___1 02:00-02:15<br>2 weai_time_12_other_m___2 02:15-02:30<br>3 weai_time_12_other_m___3 02:30-02:45<br>4 weai_time_12_other_m___4 02:45-03:00<br>5 weai_time_12_other_m___5 03:00-03:15<br>6 weai_time_12_other_m___6 03:15-03:30<br>7 weai_time_12_other_m___7 03:30-03:45<br>8 weai_time_12_other_m___8 03:45-04:00
1612	weai_time_12_other_specify_m<br>Show the field ONLY if:<br>[weai_time_12_other_m(1)] or [weai_time_12_other_m(2)] or [weai_time_12_other_m(3)] or [weai_time_12_other_m(4)] or [weai_time_12_other_m(5)] or [weai_time_12_other_m(6)] or [weai_time_12_other_m(7)] or [weai_time_12_other_m(8)]	Specify Other	text<br>Custom alignment: LV
1613	weai_24hrs_work_m	2) In the last 24 hours did you work (at home or outside of the home) more than usual, about the same as usual, or less than usual?	radio<br>1 More than usual<br>2 About the same as usual<br>3 Less than usual<br>Custom alignment: LV
1614	weai_group_ag_m	Section Header: *"Now I'm going to ask you about groups in the community. These can be either formal or informal and customary groups."*<br>3) Is there an Agricultural / livestock/ fisheries producer's group (including marketing groups) in your community?	radio<br>1 Yes<br>2 No<br>97 Don't know<br>Custom alignment: LV
1615	weai_group_ag_active_m<br>Show the field ONLY if:<br>[weai_group_ag_m] = '1'	3a) Are you an active member of this [Agricultural / livestock/ fisheries producer's group (including marketing groups)]?	radio<br>1 Yes<br>2 No<br>Custom alignment: LV
1616	weai_group_water_m	4) Is there a Water users' group in your community?	radio<br>1 Yes<br>2 No<br>97 Don't know<br>Custom alignment: LV
1617	weai_group_water_active_m<br>Show the field ONLY if:<br>[weai_group_water_m] = '1'	4a) Are you an active member of this Water users' group?	radio<br>1 Yes<br>2 No<br>Custom alignment: LV
1618	weai_group_forest_m	5) Is there a Forest users' group in your community?	radio<br>1 Yes<br>2 No<br>97 Don't know<br>Custom alignment: LV

1619	weai_group_forest_active_m<br>Show the field ONLY if:<br>[weai_group_forest_m] = '1'	5a) Are you an active member of this Forest users' group?	radio<br>1 Yes<br>2 No<br>Custom alignment: LV
1620	weai_group_credit_m	6) Is there a Credit or microfinance group (including SACCOs/merry-go-rounds/ VSLAs) in your community?	radio<br>1 Yes<br>2 No<br>97 Don't know<br>Custom alignment: LV
1621	weai_group_credit_active_m<br>Show the field ONLY if:<br>[weai_group_credit_m] = '1'	6a) Are you an active member of this Credit or microfinance group (including SACCOs/merry-go-rounds/ VSLAs)?	radio<br>1 Yes<br>2 No<br>Custom alignment: LV
1622	weai_group_mutual_m	7) Is there a Mutual help or insurance group (including burial societies) in your community?	radio<br>1 Yes<br>2 No<br>97 Don't know<br>Custom alignment: LV
1623	weai_group_mutual_active_m<br>Show the field ONLY if:<br>[weai_group_mutual_m] = '1'	7a) Are you an active member of this Mutual help or insurance group (including burial societies)?	radio<br>1 Yes<br>2 No<br>Custom alignment: LV
1624	weai_group_trade_m	8) Is there a Trade and business association group in your community?	radio<br>1 Yes<br>2 No<br>97 Don't know<br>Custom alignment: LV
1625	weai_group_trade_active_m<br>Show the field ONLY if:<br>[weai_group_trade_m] = '1'	8a) Are you an active member of this Trade and business association group?	radio<br>1 Yes<br>2 No<br>Custom alignment: LV
1626	weai_group_civics_m	9) Is there a Civic groups (improving community) or charitable group (helping others) in your community?	radio<br>1 Yes<br>2 No<br>97 Don't know<br>Custom alignment: LV
1627	weai_group_civics_active_m<br>Show the field ONLY if:<br>[weai_group_civics_m] = '1'	9a) Are you an active member of this Civic groups (improving community) or charitable group (helping others)?	radio<br>1 Yes<br>2 No<br>Custom alignment: LV
1628	weai_group_religious_m	10) Is there a Religious group in your community?	radio<br>1 Yes<br>2 No<br>97 Don't know<br>Custom alignment: LV

1629	weai_group_religion_active_m<br>Show the field ONLY if:<br>[weai_group_religious_m] = '1'	10a) Are you an active member of this Religious group?	radio<br>1 Yes<br>2 No<br>Custom alignment: LV
1630	weai_group_other_m	11) Is there an Other [women's/men's] group (only if it does not fit into one of the other categories) in your community?	radio<br>1 Yes<br>2 No<br>97 Don't know<br>Custom alignment: LV
1631	weai_group_other_active_m<br>Show the field ONLY if:<br>[weai_group_other_m] = '1'	11a) Are you an active member of this Other [women's/men's] group?	radio<br>1 Yes<br>2 No<br>Custom alignment: LV
1632	weai_group_other2_m	12) Is there an Other [specify type below] group in your community?	radio<br>1 Yes<br>2 No<br>97 Don't know<br>Custom alignment: LV
1633	weai_group_other2_specify_m<br>Show the field ONLY if:<br>[weai_group_other2_m] = '1'	12a) Specify Other group:	text<br>Custom alignment: LV
1634	weai_group_other2_active_m<br>Show the field ONLY if:<br>[weai_group_other2_m] = '1'	12b) Are you an active member of this Other group?	radio<br>1 Yes<br>2 No<br>Custom alignment: LV
1635	weai_outcome_m	2) OUTCOME OF INTERVIEW:	radio<br>1 COMPLETED<br>2 HOUSEHOLD MEMBER TOO ILL TO RESPOND/COGNITIVELY IMPAIRED<br>3 RESPONDENT NOT AT HOME/TEMPORARILY UNAVAILABLE<br>4 RESPONDENT NOT AT HOME/EXTENDED ABSENCE<br>5 REFUSED<br>6 COULD NOT LOCATE<br>Custom alignment: LV
1636	aweai_male_complete	Section Header: *Form Status*<br>Complete?	dropdown<br>0 Incomplete<br>1 Unverified<br>2 Complete
Instrument: **Anthropometrics Female Only** (anthropometrics_female_only)			Collapse
1637	an_child_dom	Date of Measurement	text (date_dmy), Required<br>Custom alignment: LV
1638	an_child_age	AN1) Child Date of Birth [enrollment_arm_1][en_child_dob]	text (date_dmy), Required<br>Custom alignment: LV<br>Field Annotation: @HIDEBUTTON
1639	an_month_calc	Calculated age in months for this survey	calc<br>Calculation: datediff ([an_child_dom], [an_child_age], "M")

1640	an_child_sex	AN2) Child sex [enrollment_arm_1][en_child_sex]	radio<br>1 Male<br>2 Female<br>Custom alignment: LV
1641	an_muac	Section Header: *Anthropometrics*<br>AN3) MUAC (mm)	text (number, Min: 9, Max: 20)<br>Custom alignment: LV
1642	an_head_circumf	AN3.1) Head circumferences (cm)	text (number, Min: 35, Max: 50)<br>Custom alignment: LV
1643	an_weight	AN4) Weight (kg)	text (number)<br>Custom alignment: LV
1644	an_height1	AN5) Height 1 (cm)	text (number)<br>Custom alignment: LV
1645	an_height2	AN6) Height 2 (cm)	text (number)<br>Custom alignment: LV
1646	an_height3	AN7) Height 3 (cm)	text (number)<br>Custom alignment: LV
1647	an_vaccine_card_pres	Section Header:<br>AN8) Vaccine card present?	yesno<br>1 Yes<br>0 No<br>Custom alignment: LV
1648	an_diarrhea_current	Section Header: *Currently:*<br>AN9) Does child currently have diarrhea?	yesno<br>1 Yes<br>0 No<br>Custom alignment: LV
1649	an_fever_current	AN10) Does child currently have fever?	yesno<br>1 Yes<br>0 No<br>Custom alignment: LV
1650	an_nursing_current	AN11) Is child currently nursing?	yesno<br>1 Yes<br>0 No<br>Custom alignment: LV
1651	an_referred	AN12) Was the child referred to a health clinic on the basis of information gathered during the data collection process?	yesno<br>1 Yes<br>0 No<br>Custom alignment: LV
1652	an_referred_purpose<br>Show the field ONLY if:<br>[an_referred] = '1'	AN12a) For what purpose?	notes<br>Custom alignment: LV
1653	an_edema	AN13) Pitting Edema?	yesno, Required<br>1 Yes<br>0 No<br>Custom alignment: LV
1654	anthropometrics_female_only_complete	Section Header: *Form Status*<br>Complete?	dropdown<br>0 Incomplete<br>1 Unverified<br>2 Complete

Instrument: **Coronavirus checker** (coronavirus_checker) Collapse

1655	cor_qualtrics	Qualtrics questionnaire ID:	text<br>Custom alignment: LV
1656	work_id	Workplace ID:	text
1657	cor_staff_name	1. Name	dropdown (autocomplete)<br>1 Elizabeth Schieber<br>2 Amanda Ojeda<br>3 Yitagele Terefe Mokonnon<br>4 Bahar Mummed Hassen<br>5 Kedir Abdi<br>6 Yenenesh Demisse W/Senbet<br>7 Sada Muktar Mohammed<br>8 Jafer Kedir Amin<br>9 Ibsa Abduselam Ahmed<br>10 Ibsa Aliyi Usman<br>11 Abdulmuen Mohammed<br>12 Meri Mahammed Usmail<br>13 Halengo Tamane Game<br>14 Ballo Abrahim Mummed<br>15 Fayo Idris Abrahim<br>16 Loic Deblais<br>17 Amme Yousuf Hassen<br>18 Kedir Teji Roba<br>Custom alignment: LV
1658	cor_pos_result_date	2. Date of positive screening result	text (date_dmy)<br>Custom alignment: LV
1659	cor_self_iso_date	3. Date of starting self-isolation	text (date_dmy)<br>Custom alignment: LV
1660	cor_self_iso_date_end	4. Date of ending self-isolation	text (date_dmy)<br>Custom alignment: LV
1661	cor_samp_submit	5. Was a sample submitted?	yesno<br>1 Yes<br>0 No<br>Custom alignment: LV
1662	cor_samp_submit_date<br>Show the field ONLY if:<br>[cor_samp_submit] = '1'	When was the sample was submitted?	text (date_dmy)<br>Custom alignment: LV
1663	cor_samp_test_result_date<br>Show the field ONLY if:<br>[cor_samp_submit] = '1'	When was the test result received?	text (date_dmy)<br>Custom alignment: LV
1664	cor_samp_test_result<br>Show the field ONLY if:<br>[cor_samp_submit] = '1'	What was the test result?	radio<br>1 Positive<br>0 Negative<br>Custom alignment: LV
1665	cor_samp_pos_repeat<br>Show the field ONLY if:<br>[cor_samp_test_result] = '1'	(If positive) Was a repeat test sample submitted?	yesno<br>1 Yes<br>0 No<br>Custom alignment: LV
1666	cor_samp_repeat_date<br>Show the field ONLY if:<br>[cor_samp_pos_repeat] = '1'	(If yes) When was the test result received?	text (date_dmy)<br>Custom alignment: LV

1667	cor_samp_repeat_result<br>Show the field ONLY if:<br>[cor_samp_pos_repeat] = '1'	What was the retest result?	radio<br>1 Positive<br>0 Negative<br>Custom alignment: LV
1668	cor_return_work	6. Has [cor_staff_name] returned to work?	yesno<br>1 Yes<br>0 No<br>Custom alignment: LV
1669	cor_return_work_when<br>Show the field ONLY if:<br>[cor_return_work] = '1'	(If yes) When did [cor_staff_name] return to work?	text (date_dmy)<br>Custom alignment: LV
1670	cor_treated_md	7. Was [cor_staff_name] treated by a medical doctor?	yesno<br>1 Yes<br>0 No<br>Custom alignment: LV
1671	cor_symptom_free	8. (To be completed 10 days after first symptoms) is [cor_staff_name] free of symptoms?	yesno<br>1 Yes<br>0 No<br>Custom alignment: LV
1672	coronavirus_checker_complete	Section Header: *Form Status*<br>Complete?	dropdown<br>0 Incomplete<br>1 Unverified<br>2 Complete
Instrument: **Campylobacter Culture Feces** (campylobacter_culture_feces)			Collapse
1673	ch_campy_sample_type	Section Header: *Campylobacter culture*<br>The Questions below are for the Child sample type by plating	descriptive
1674	ch_campy_c_lod	Is unconfirmed Campylobacter concentration below limit of detection (LoD)?	yesno<br>1 Yes<br>0 No<br>Custom alignment: LV
1675	ch_campy_c_unconfirm_conc<br>Show the field ONLY if:<br>[ch_campy_c_lod] = '0'	Unconfirmed Campylobacter concentration<br>*cfu/g*	text (number)<br>Custom alignment: LV
1676	ch_campy_c_colonies<br>Show the field ONLY if:<br>[ch_campy_c_lod] = '0'	Number of colonies for pure culturing	text (integer)<br>Custom alignment: LV
1677	ch_campy_c_confirm1<br>Show the field ONLY if:<br>[ch_campy_c_colonies] > 0	Confirmation result per colony	radio<br>0 Absent<br>1 Present<br>Custom alignment: LV
1678	ch_campy_c_confirm2<br>Show the field ONLY if:<br>[ch_campy_c_colonies] > 1	Confirmation result per colony	radio<br>0 Absent<br>1 Present<br>Custom alignment: LV
1679	ch_campy_c_confirm3<br>Show the field ONLY if:<br>[ch_campy_c_colonies] > 2	Confirmation result per colony	radio<br>0 Absent<br>1 Present<br>Custom alignment: LV

Havelaar AH, *et al. BMJ Open* 2022; 12:e061311. doi: 10.1136/bmjopen-2022-061311

1680	ch_campy_c_confirm4<br>Show the field ONLY if:<br>[ch_campy_c_colonies] > 2	Confirmation result per colony	radio<br>0 Absent<br>1 Present<br>Custom alignment: LV
1681	ch_campy_c_confirm5<br>Show the field ONLY if:<br>[ch_campy_c_colonies] > 2	Confirmation result per colony	radio<br>0 Absent<br>1 Present<br>Custom alignment: LV
1682	ch_campy_c_species<br>Show the field ONLY if:<br>[ch_campy_c_lod] = '0'	Campylobacter species for confirmed colonies<br>*check all that apply*	checkbox<br>1 ch_campy_c_species___1 Cj<br>2 ch_campy_c_species___2 Cc<br>3 ch_campy_c_species___3 Co<br>Custom alignment: LV
1683	ch_campy_c_cj<br>Show the field ONLY if:<br>[ch_campy_c_species(1)] = '1'	Number of Cj colonies	text (integer)<br>Custom alignment: LV
1684	ch_campy_c_cc<br>Show the field ONLY if:<br>[ch_campy_c_species(2)] = '1'	Number of Cc colonies	text (integer)<br>Custom alignment: LV
1685	ch_campy_c_co<br>Show the field ONLY if:<br>[ch_campy_c_species(3)] = '1'	Number of Co colonies	text (integer)<br>Custom alignment: LV
1686	ch_campy_enrichment<br>Show the field ONLY if:<br>[ch_campy_c_lod] = '1'	Section Header: *Campylobacter enrichment*<br>Result of enrichment	radio<br>0 Absent<br>1 Present<br>Custom alignment: LV
1687	ch_campy_colony_pr<br>Show the field ONLY if:<br>[ch_campy_enrichment] = '1'	Number of colonies for pure culturing	text (integer, Min: 0, Max: 3)<br>Custom alignment: LV
1688	ch_campy_result_pr1<br>Show the field ONLY if:<br>[ch_campy_colony_pr] > 0	Confirmation result per colony	radio<br>0 Absent<br>1 Present<br>Custom alignment: LV
1689	ch_campy_result_pr2<br>Show the field ONLY if:<br>[ch_campy_colony_pr] > 1	Confirmation result per colony	radio<br>0 Absent<br>1 Present<br>Custom alignment: LV
1690	ch_campy_result_pr3<br>Show the field ONLY if:<br>[ch_campy_colony_pr] > 2	Confirmation result per colony	radio<br>0 Absent<br>1 Present<br>Custom alignment: LV
1691	ch_campy_species_pr<br>Show the field ONLY if:<br>[ch_campy_enrichment] = '1'	Campylobacter species for confirmed colonies<br>*check all that apply*	checkbox<br>1 ch_campy_species_pr___1 Cj<br>2 ch_campy_species_pr___2 Cc<br>3 ch_campy_species_pr___3 Co<br>Custom alignment: LV

1692	ch_campy_cj_pr<br>Show the field ONLY if:<br>[ch_campy_species_pr(1)] = '1'	Number of Cj colonies	text (integer)<br>Custom alignment: LV
1693	ch_campy_cc_pr<br>Show the field ONLY if:<br>[ch_campy_species_pr(2)] = '1'	Number of Cc colonies	text (integer)<br>Custom alignment: LV
1694	ch_campy_co_pr<br>Show the field ONLY if:<br>[ch_campy_species_pr(3)] = '1'	Number of Co colonies	text (integer)<br>Custom alignment: LV
1695	ch_campy_conc_pcr_lod	Section Header: *Campylobacter PCR*<br>Is the Campylobacter PCR concentration below the limit of detection (LoD)?	yesno<br>1 Yes<br>0 No<br>Custom alignment: LV
1696	ch_campy_conc_pcr<br>Show the field ONLY if:<br>[ch_campy_conc_pcr_lod] = '0'	Campylobacter concentration<br>*gene copies/g*	text (number)<br>Custom alignment: LV
1697	p_campy_sample_type	Section Header: *Campylobacter culture*<br>The Questions below are for the Poultry sample type by plating	descriptive
1698	p_campy_c_lod	Is unconfirmed Campylobacter concentration below limit of detection (LoD)?	yesno<br>1 Yes<br>0 No<br>Custom alignment: LV
1699	p_campy_c_unconfirm_conc<br>Show the field ONLY if:<br>[p_campy_c_lod] = '0'	Unconfirmed Campylobacter concentration<br>*cfu/g*	text (number)<br>Custom alignment: LV
1700	p_campy_c_colonies<br>Show the field ONLY if:<br>[p_campy_c_lod] = '0'	Number of colonies for pure culturing	text (integer)<br>Custom alignment: LV
1701	p_campy_c_confirm1<br>Show the field ONLY if:<br>[p_campy_c_colonies] > 0	Confirmation result per colony	radio<br>0 Absent<br>1 Present<br>Custom alignment: LV
1702	p_campy_c_confirm2<br>Show the field ONLY if:<br>[p_campy_c_colonies] > 1	Confirmation result per colony	radio<br>0 Absent<br>1 Present<br>Custom alignment: LV
1703	p_campy_c_confirm3<br>Show the field ONLY if:<br>[p_campy_c_colonies] > 2	Confirmation result per colony	radio<br>0 Absent<br>1 Present<br>Custom alignment: LV
1704	p_campy_c_confirm4<br>Show the field ONLY if:<br>[p_campy_c_colonies] > 2	Confirmation result per colony	radio<br>0 Absent<br>1 Present<br>Custom alignment: LV
1705	p_campy_c_confirm5<br>Show the field ONLY if:<br>[p_campy_c_colonies] > 2	Confirmation result per colony	radio<br>0 Absent<br>1 Present<br>Custom alignment: LV

1706	p_campy_c_species<br><br>Show the field ONLY if:<br>[p_campy_c_lod] = '0'	Campylobacter species for confirmed colonies<br>*check all that apply*	checkbox<br>1 p_campy_c_species___1 Cj<br>2 p_campy_c_species___2 Cc<br>3 p_campy_c_species___3 Co<br><br>Custom alignment: LV
1707	p_campy_c_cj<br><br>Show the field ONLY if:<br>[p_campy_c_species(1)] = '1'	Number of Cj colonies	text (integer)<br>Custom alignment: LV
1708	p_campy_c_cc<br><br>Show the field ONLY if:<br>[p_campy_c_species(2)] = '1'	Number of Cc colonies	text (integer)<br>Custom alignment: LV
1709	p_campy_c_co<br><br>Show the field ONLY if:<br>[p_campy_c_species(3)] = '1'	Number of Co colonies	text (integer)<br>Custom alignment: LV
1710	p_campy_enrichment<br><br>Show the field ONLY if:<br>[p_campy_c_lod] = '1'	Section Header: *Campylobacter enrichment*<br>Result of enrichment	radio<br>0 Absent<br>1 Present<br><br>Custom alignment: LV
1711	p_campy_colony_pr<br><br>Show the field ONLY if:<br>[p_campy_enrichment] = '1'	Number of colonies for pure culturing	text (integer, Min: 0, Max: 3)<br>Custom alignment: LV
1712	p_campy_result_pr1<br><br>Show the field ONLY if:<br>[p_campy_colony_pr] > 0	Confirmation result per colony	radio<br>0 Absent<br>1 Present<br><br>Custom alignment: LV
1713	p_campy_result_pr2<br><br>Show the field ONLY if:<br>[p_campy_colony_pr] > 1	Confirmation result per colony	radio<br>0 Absent<br>1 Present<br><br>Custom alignment: LV
1714	p_campy_result_pr3<br><br>Show the field ONLY if:<br>[p_campy_colony_pr] > 2	Confirmation result per colony	radio<br>0 Absent<br>1 Present<br><br>Custom alignment: LV
1715	p_campy_species_pr<br><br>Show the field ONLY if:<br>[p_campy_enrichment] = '1'	Campylobacter species for confirmed colonies<br>*check all that apply*	checkbox<br>1 p_campy_species_pr___1 Cj<br>2 p_campy_species_pr___2 Cc<br>3 p_campy_species_pr___3 Co<br><br>Custom alignment: LV
1716	p_campy_cj_pr<br><br>Show the field ONLY if:<br>[p_campy_species_pr(1)] = '1'	Number of Cj colonies	text (integer)<br>Custom alignment: LV
1717	p_campy_cc_pr<br><br>Show the field ONLY if:<br>[p_campy_species_pr(2)] = '1'	Number of Cc colonies	text (integer)<br>Custom alignment: LV
1718	p_campy_co_pr<br><br>Show the field ONLY if:<br>[p_campy_species_pr(3)] = '1'	Number of Co colonies	text (integer)<br>Custom alignment: LV

Havelaar AH, *et al. BMJ Open* 2022; 12:e061311. doi: 10.1136/bmjopen-2022-061311

1719	p_campy_conc_pcr_lod	Section Header: *Campylobacter PCR*<br>Is the Campylobacter PCR concentration below the limit of detection (LoD)?	yesno<br>1 Yes<br>0 No<br>Custom alignment: LV
1720	p_campy_conc_pcr<br>Show the field ONLY if:<br>[p_campy_conc_pcr_lod] = '0'	Campylobacter concentration<br>*gene copies/g*	text (number)<br>Custom alignment: LV
1721	b_campy_sample_type	Section Header: *Campylobacter culture*<br>The Questions below are for the Bovine sample type by plating	descriptive
1722	b_campy_c_lod	Is unconfirmed Campylobacter concentration below limit of detection (LoD)?	yesno<br>1 Yes<br>0 No<br>Custom alignment: LV
1723	b_campy_c_unconfirm_conc<br>Show the field ONLY if:<br>[b_campy_c_lod] = '0'	Unconfirmed Campylobacter concentration<br>*cfu/g*	text (number)<br>Custom alignment: LV
1724	b_campy_c_colonies<br>Show the field ONLY if:<br>[b_campy_c_lod] = '0'	Number of colonies for pure culturing	text (integer)<br>Custom alignment: LV
1725	b_campy_c_confirm1<br>Show the field ONLY if:<br>[b_campy_c_colonies] > 0	Confirmation result per colony	radio<br>0 Absent<br>1 Present<br>Custom alignment: LV
1726	b_campy_c_confirm2<br>Show the field ONLY if:<br>[b_campy_c_colonies] > 1	Confirmation result per colony	radio<br>0 Absent<br>1 Present<br>Custom alignment: LV
1727	b_campy_c_confirm3<br>Show the field ONLY if:<br>[b_campy_c_colonies] > 2	Confirmation result per colony	radio<br>0 Absent<br>1 Present<br>Custom alignment: LV
1728	b_campy_c_confirm4<br>Show the field ONLY if:<br>[b_campy_c_colonies] > 2	Confirmation result per colony	radio<br>0 Absent<br>1 Present<br>Custom alignment: LV
1729	b_campy_c_confirm5<br>Show the field ONLY if:<br>[b_campy_c_colonies] > 2	Confirmation result per colony	radio<br>0 Absent<br>1 Present<br>Custom alignment: LV
1730	b_campy_c_species<br>Show the field ONLY if:<br>[b_campy_c_lod] = '0'	Campylobacter species for confirmed colonies<br>*check all that apply*	checkbox<br>1 b_campy_c_species___1 Cj<br>2 b_campy_c_species___2 Cc<br>3 b_campy_c_species___3 Co<br>Custom alignment: LV
1731	b_campy_c_cj<br>Show the field ONLY if:<br>[b_campy_c_species(1)] = '1'	Number of Cj colonies	text (integer)<br>Custom alignment: LV

Supplemental material
BMJ Publishing Group Limited (BMJ) disclaims all liability and responsibility arising from any reliance placed on this supplemental material which has been supplied by the author(s)
BMJ Open

1732	b_campy_c_cc<br>Show the field ONLY if:<br>[b_campy_c_species(2)] = '1'	Number of Cc colonies	text (integer)<br>Custom alignment: LV
1733	b_campy_c_co<br>Show the field ONLY if:<br>[b_campy_c_species(3)] = '1'	Number of Co colonies	text (integer)<br>Custom alignment: LV
1734	b_campy_enrichment<br>Show the field ONLY if:<br>[b_campy_c_lod] = '1'	Section Header: *Campylobacter enrichment*<br>Result of enrichment	radio<br>0 Absent<br>1 Present<br>Custom alignment: LV
1735	b_campy_colony_pr<br>Show the field ONLY if:<br>[b_campy_enrichment] = '1'	Number of colonies for pure culturing	text (integer, Min: 0, Max: 3)<br>Custom alignment: LV
1736	b_campy_result_pr1<br>Show the field ONLY if:<br>[b_campy_colony_pr] > 0	Confirmation result per colony	radio<br>0 Absent<br>1 Present<br>Custom alignment: LV
1737	b_campy_result_pr2<br>Show the field ONLY if:<br>[b_campy_colony_pr] > 1	Confirmation result per colony	radio<br>0 Absent<br>1 Present<br>Custom alignment: LV
1738	b_campy_result_pr3<br>Show the field ONLY if:<br>[b_campy_colony_pr] > 2	Confirmation result per colony	radio<br>0 Absent<br>1 Present<br>Custom alignment: LV
1739	b_campy_species_pr<br>Show the field ONLY if:<br>[b_campy_enrichment] = '1'	Campylobacter species for confirmed colonies<br>*check all that apply*	checkbox<br>1 b_campy_species_pr___1 Cj<br>2 b_campy_species_pr___2 Cc<br>3 b_campy_species_pr___3 Co<br>Custom alignment: LV
1740	b_campy_cj_pr<br>Show the field ONLY if:<br>[b_campy_species_pr(1)] = '1'	Number of Cj colonies	text (integer)<br>Custom alignment: LV
1741	b_campy_cc_pr<br>Show the field ONLY if:<br>[b_campy_species_pr(2)] = '1'	Number of Cc colonies	text (integer)<br>Custom alignment: LV
1742	b_campy_co_pr<br>Show the field ONLY if:<br>[b_campy_species_pr(3)] = '1'	Number of Co colonies	text (integer)<br>Custom alignment: LV
1743	b_campy_conc_pcr_lod	Section Header: *Campylobacter PCR*<br>Is the Campylobacter PCR concentration below the limit of detection (LoD)?	yesno<br>1 Yes<br>0 No<br>Custom alignment: LV
1744	b_campy_conc_pcr<br>Show the field ONLY if:<br>[b_campy_conc_pcr_lod] = '0'	Campylobacter concentration<br>*gene copies/g*	text (number)<br>Custom alignment: LV
1745	g_campy_sample_type	Section Header: *Campylobacter culture*<br>The Questions below are for the Goat sample type by plating	descriptive

Havelaar AH, *et al. BMJ Open* 2022; 12:e061311. doi: 10.1136/bmjopen-2022-061311

1746	g_campy_c_lod	Is unconfirmed Campylobacter concentration below limit of detection (LoD)?	yesno<br>1 Yes<br>0 No<br>Custom alignment: LV
1747	g_campy_c_unconfirm_conc<br>Show the field ONLY if:<br>[g_campy_c_lod] = '0'	Unconfirmed Campylobacter concentration<br>*cfu/g*	text (number)<br>Custom alignment: LV
1748	g_campy_c_colonies<br>Show the field ONLY if:<br>[g_campy_c_lod] = '0'	Number of colonies for pure culturing	text (integer)<br>Custom alignment: LV
1749	g_campy_c_confirm1<br>Show the field ONLY if:<br>[g_campy_c_colonies] > 0	Confirmation result per colony	radio<br>0 Absent<br>1 Present<br>Custom alignment: LV
1750	g_campy_c_confirm2<br>Show the field ONLY if:<br>[g_campy_c_colonies] > 1	Confirmation result per colony	radio<br>0 Absent<br>1 Present<br>Custom alignment: LV
1751	g_campy_c_confirm3<br>Show the field ONLY if:<br>[g_campy_c_colonies] > 2	Confirmation result per colony	radio<br>0 Absent<br>1 Present<br>Custom alignment: LV
1752	g_campy_c_confirm4<br>Show the field ONLY if:<br>[g_campy_c_colonies] > 2	Confirmation result per colony	radio<br>0 Absent<br>1 Present<br>Custom alignment: LV
1753	g_campy_c_confirm5<br>Show the field ONLY if:<br>[g_campy_c_colonies] > 2	Confirmation result per colony	radio<br>0 Absent<br>1 Present<br>Custom alignment: LV
1754	g_campy_c_species<br>Show the field ONLY if:<br>[g_campy_c_lod] = '0'	Campylobacter species for confirmed colonies<br>*check all that apply*	checkbox<br>1 g_campy_c_species___1 Cj<br>2 g_campy_c_species___2 Cc<br>3 g_campy_c_species___3 Co<br>Custom alignment: LV
1755	g_campy_c_cj<br>Show the field ONLY if:<br>[g_campy_c_species(1)] = '1'	Number of Cj colonies	text (integer)<br>Custom alignment: LV
1756	g_campy_c_cc<br>Show the field ONLY if:<br>[g_campy_c_species(2)] = '1'	Number of Cc colonies	text (integer)<br>Custom alignment: LV
1757	g_campy_c_co<br>Show the field ONLY if:<br>[g_campy_c_species(3)] = '1'	Number of Co colonies	text (integer)<br>Custom alignment: LV
1758	g_campy_enrichment<br>Show the field ONLY if:<br>[g_campy_c_lod] = '1'	Section Header: *Campylobacter enrichment*<br>Result of enrichment	radio<br>0 Absent<br>1 Present<br>Custom alignment: LV

1759	g_campy_colony_pr<br>Show the field ONLY if:<br>[g_campy_enrichment] = '1'	Number of colonies for pure culturing	text (integer, Min: 0, Max: 3)<br>Custom alignment: LV
1760	g_campy_result_pr1<br>Show the field ONLY if:<br>[g_campy_colony_pr] > 0	Confirmation result per colony	radio<br>0 Absent<br>1 Present<br>Custom alignment: LV
1761	g_campy_result_pr2<br>Show the field ONLY if:<br>[g_campy_colony_pr] > 1	Confirmation result per colony	radio<br>0 Absent<br>1 Present<br>Custom alignment: LV
1762	g_campy_result_pr3<br>Show the field ONLY if:<br>[g_campy_colony_pr] > 2	Confirmation result per colony	radio<br>0 Absent<br>1 Present<br>Custom alignment: LV
1763	g_campy_species_pr<br>Show the field ONLY if:<br>[g_campy_enrichment] = '1'	Campylobacter species for confirmed colonies<br>*check all that apply*	checkbox<br>1 g_campy_species_pr___1 Cj<br>2 g_campy_species_pr___2 Cc<br>3 g_campy_species_pr___3 Co<br>Custom alignment: LV
1764	g_campy_cj_pr<br>Show the field ONLY if:<br>[g_campy_species_pr(1)] = '1'	Number of Cj colonies	text (integer)<br>Custom alignment: LV
1765	g_campy_cc_pr<br>Show the field ONLY if:<br>[g_campy_species_pr(2)] = '1'	Number of Cc colonies	text (integer)<br>Custom alignment: LV
1766	g_campy_co_pr<br>Show the field ONLY if:<br>[g_campy_species_pr(3)] = '1'	Number of Co colonies	text (integer)<br>Custom alignment: LV
1767	g_campy_conc_pcr_lod	Section Header: *Campylobacter PCR*<br>Is the Campylobacter PCR concentration below the limit of detection (LoD)?	yesno<br>1 Yes<br>0 No<br>Custom alignment: LV
1768	g_campy_conc_pcr<br>Show the field ONLY if:<br>[g_campy_conc_pcr_lod] = '0'	Campylobacter concentration<br>*gene copies/g*	text (number)<br>Custom alignment: LV
1769	campylobacter_culture_feces_complete	Section Header: *Form Status*<br>Complete?	dropdown<br>0 Incomplete<br>1 Unverified<br>2 Complete
Instrument: **Eed Analysis** (eed_analysis)			Collapse
1770	urine_lr	L/R ratio	text (number, Min: 0, Max: 1)<br>Custom alignment: LV
1771	urine_l	% L	text (number, Min: 1, Max: 100)<br>Custom alignment: LV
1772	urine_r	% R	text (number, Min: 1, Max: 100)<br>Custom alignment: LV
1773	fecal_mpo	Fecal MPO<br>*ng/mL*	text (number)<br>Custom alignment: LV

1774	eed_analysis_complete	Section Header: *Form Status*<br>Complete?	dropdown<br>0 Incomplete<br>1 Unverified<br>2 Complete
	Instrument: **Campylobacter Culture Drag Samples** (campylobacter_culture_drag_samples)		Collapse
1775	drag_location_id	Location ID	text<br>Custom alignment: LV
1776	drag_sample_date	Sampling date	text (date_ymd)<br>Custom alignment: LV
1777	campy_enrichment_drag	Section Header: *Campylobacter enrichment*<br>Result of enrichment	radio<br>0 0<br>1 1<br>Custom alignment: LV
1778	campy_colony_pr_drag<br>Show the field ONLY if:<br>[campy_enrichment_drag] = '1'	Number of colonies for pure culturing	text (integer)<br>Custom alignment: LV
1779	campy_result_pr1_drag<br>Show the field ONLY if:<br>[campy_colony_pr_drag] > 0	Confirmation result per colony	radio<br>0 0<br>1 1<br>Custom alignment: LV
1780	campy_result_pr2_drag<br>Show the field ONLY if:<br>[campy_colony_pr_drag] > 1	Confirmation result per colony	radio<br>0 0<br>1 1<br>Custom alignment: LV
1781	campy_result_pr3_drag<br>Show the field ONLY if:<br>[campy_colony_pr_drag] > 2	Confirmation result per colony	radio<br>0 0<br>1 1<br>Custom alignment: LV
1782	campy_species_pr_drag	Campylobacter species for confirmed colonies<br>*check all that apply*	checkbox<br>1 campy_species_pr_drag___1 Cj<br>2 campy_species_pr_drag___2 Cc<br>3 campy_species_pr_drag___3 Co<br>Custom alignment: LV
1783	campy_cj_pr_drag<br>Show the field ONLY if:<br>[campy_species_pr_drag(1)] = '1'	Number of Cj colonies	text (integer)<br>Custom alignment: LV
1784	campy_cc_pr_drag<br>Show the field ONLY if:<br>[campy_species_pr_drag(2)] = '1'	Number of Cc colonies	text (integer)<br>Custom alignment: LV
1785	campy_co_pr_drag<br>Show the field ONLY if:<br>[campy_species_pr_drag(3)] = '1'	Number of Co colonies	text (integer)<br>Custom alignment: LV
1786	campylobacter_culture_drag_samples_complete	Section Header: *Form Status*<br>Complete?	dropdown<br>0 Incomplete<br>1 Unverified<br>2 Complete
	Instrument: **Protozoan Parasites** (protozoan_parasites)		Collapse

1787	feces_crypto_detect	Was Cryptosporidium detected?	yesno<br>1 Yes<br>0 No<br><br>Custom alignment: LV
1788	feces_crypto_conc<br><br>Show the field ONLY if:<br>[feces_crypto_detect] = '1'	Cryptosporidium concentration<br>*oocyst/g*	text (number)<br>Custom alignment: LV
1789	feces_crypto_species<br><br>Show the field ONLY if:<br>[feces_crypto_detect] = '1'	Cryptosporidium species<br>*check all that apply*	checkbox<br>1 feces_crypto_species___1 parvum<br>2 feces_crypto_species___2 hominis<br>88 feces_crypto_species___88 other<br><br>Custom alignment: LV
1790	feces_crypto_species_oth<br><br>Show the field ONLY if:<br>[feces_crypto_species(88)] = '1'	Specify Other:	text<br>Custom alignment: LV
1791	feces_giardia_detect	Was Giardia detected?	yesno<br>1 Yes<br>0 No<br><br>Custom alignment: LV
1792	feces_giardia_conc<br><br>Show the field ONLY if:<br>[feces_giardia_detect] = '1'	Giardia concentration<br>*cyst/g*	text (number)<br>Custom alignment: LV
1793	feces_giardia_ass	Giardia assemblage<br>*check all that apply*	checkbox<br>1 feces_giardia_ass___1 A<br>2 feces_giardia_ass___2 B<br>88 feces_giardia_ass___88 Other<br><br>Custom alignment: LV
1794	feces_giardia_ass_other<br><br>Show the field ONLY if:<br>[feces_giardia_ass(88)] = '1'	Specify Other:	text<br>Custom alignment: LV
1795	protozoan_parasites_complete	Section Header: *Form Status*<br>Complete?	dropdown<br>0 Incomplete<br>1 Unverified<br>2 Complete
Instrument: **Participant Dropout Interview DRAFT** (participant_dropout_interview_draft)			Collapse
1796	dropout_participate_v2	1. I understand that you have decided not to participate in our research project any longer. I want to thank you for your engagement up to this point. I would also like to ask you a few questions that will help me understand your decision to leave the study. Would you be willing to answer a few questions? We accept your decision to leave the study and will not try to change your mind; this information will simply help us understand the context of your decision to leave.	yesno<br>1 Yes<br>0 No<br><br>Custom alignment: LV
1797	dropout_reason_v2<br><br>Show the field ONLY if:<br>[dropout_participate_v2] = '1'	2. What is the major reason you have decided to no longer participate in the study?	text<br>Custom alignment: LV
1798	dropout_done_differently_v2<br><br>Show the field ONLY if:<br>[dropout_participate_v2] = '1'	3. Is there anything we could have done differently as researchers to keep you engaged in the study?	text<br>Custom alignment: LV
1799	dropout_experience_v2<br><br>Show the field ONLY if:<br>[dropout_participate_v2] = '1'	4. Is there anything you'd like for us to know or understand about your experience as a participant up to this point?	text<br>Custom alignment: LV

1800	dropout_sample_issue	5. Is there concern about sharing your already collected data or samples?	yesno<br>1 Yes<br>0 No<br>Custom alignment: LV
1801	dropout_destroy_data<br>Show the field ONLY if: [dropout_sample_issue] = '1'	If yes, may we retain and analyze your already collected data up to the time of withdrawal?	radio<br>1 Yes, we will retain and analyze already collected data up to the time of subject withdrawal<br>0 No, destroy the subject's data, excluding the subject's data from any further analysis.<br>Custom alignment: LV
1802	participant_dropout_interview_draft_complete	Section Header: *Form Status*<br>Complete?	dropdown<br>0 Incomplete<br>1 Unverified<br>2 Complete
Instrument: **C5.2A DNA** (c52a_dna)			Collapse
1803	analyst	Section Header: *Raw Data management*<br>Name of the person who conducted the experiment	text<br>1 yt<br>2 bm<br>3 ao<br>4 ld<br>Custom alignment: LV
1804	date_exp	Date when the experiment was conducted	text (date_ymd)<br>Custom alignment: LV
1805	sample_id	ID utilized for CAGED & EXCAM sample<br>*Unique ID for connecting data*	text<br>Custom alignment: LV
1806	paper_date	The date the data was entered in paper log	text (date_ymd)<br>Custom alignment: LV
1807	dna_conc	Section Header: *Experimental data*<br>The DNA concentration in (ng/ul<br>*an exception code of 1234 should be entered in an error or anomaly is found in the data*	text (number, Min: 0, Max: 1000)<br>Custom alignment: LV
1808	dna_conc_com	Provide any explanation for the anomilty that was observed that required the utilization of the exception code	text<br>Custom alignment: LV
1809	lab_260_280	260/280 ratio<br>*an exception code of 1234 should be entered in an error or anomaly is found in the data*	text (number, Min: 0, Max: 3)<br>Custom alignment: LV
1810	lab_260_280_com	Provide any explanation for the anomilty that was observed that required the utilization of the exception code	text<br>Custom alignment: LV
1811	lab_260_230	260/230 ratio<br>*an exception code of 1234 should be entered in an error or anomaly is found in the data*	text (number, Min: 0, Max: 3)<br>Custom alignment: LV
1812	lab_260_230_com	Provide any explanation for the anomilty that was observed that required the utilization of the exception code	text<br>Custom alignment: LV
1813	extract_box	box # for where the extracted DNA is stored	text (number, Min: 1, Max: 200)<br>Custom alignment: LV
1814	elec_date	Section Header: *Data verification*<br>date the data was entered in the electronic template	text (date_ymd)<br>Custom alignment: LV
1815	verified_by	name of who verified the data	text<br>1 ao<br>2 ld<br>Custom alignment: LV
1816	verified_date	date the data was verified	text (date_ymd)<br>Custom alignment: LV

1817	c52a_dna_complete	Section Header: *Form Status*<br>Complete?	dropdown<br>0 Incomplete<br>1 Unverified<br>2 Complete
Instrument: **Food Security** (food_security)			Collapse
1818	ls_fs_enough_food	Section Header: *Food Security*<br>1. In the past four weeks, did you worry that your household would not have enough food?	yesno<br>1 Yes<br>0 No
1819	ls_fs_enough_food_y<br>Show the field ONLY if:<br>[ls_fs_enough_food] = '1'	If yes, how often did this happen?	dropdown, Required<br>1 Rarely (once or twice in the past four weeks)<br>2 Sometimes (three to ten times in the past four weeks)<br>3 Often (more than ten times in the past four weeks)
1820	ls_fs_food_preferred	2. In the past four weeks, were you or any household member not able to eat the kinds of<br>foods you preferred because of a lack of resources?	yesno, Required<br>1 Yes<br>0 No
1821	ls_fs_food_preferred_y<br>Show the field ONLY if:<br>[ls_fs_food_preferred] = '1'	If yes, how often did this happen?	dropdown, Required<br>1 Rarely (once or twice in the past four weeks)<br>2 Sometimes (three to ten times in the past four weeks)<br>3 Often (more than ten times in the past four weeks)
1822	ls_fs_food_variety	3. In the past four weeks, did you or any household member have to eat a limited variety of foods due to a lack of resources?	yesno, Required<br>1 Yes<br>0 No
1823	ls_fs_food_variety_y<br>Show the field ONLY if:<br>[ls_fs_food_variety] = '1'	If yes, how often did this happen?	dropdown, Required<br>1 Rarely (once or twice in the past four weeks)<br>2 Sometimes (three to ten times in the past four weeks)<br>3 Often (more than ten times in the past four weeks)
1824	ls_fs_food_same	4. In the past four weeks, did you or any household member have to eat some foods that<br>you really did not want to eat because of a lack of resources to obtain other types of<br>food?	yesno, Required<br>1 Yes<br>0 No
1825	ls_fs_food_same_y<br>Show the field ONLY if:<br>[ls_fs_food_same] = '1'	If yes, how often did this happen?	dropdown, Required<br>1 Rarely (once or twice in the past four weeks)<br>2 Sometimes (three to ten times in the past four weeks)<br>3 Often (more than ten times in the past four weeks)
1826	ls_fs_food_small	5. In the past four weeks, did you or any household member have to eat a smaller meal than<br>you felt you needed because there was not enough food?	yesno, Required<br>1 Yes<br>0 No
1827	ls_fs_food_small_y<br>Show the field ONLY if:<br>[ls_fs_food_small] = '1'	If yes, how often did this happen?	dropdown, Required<br>1 Rarely (once or twice in the past four weeks)<br>2 Sometimes (three to ten times in the past four weeks)<br>3 Often (more than ten times in the past four weeks)
1828	ls_fs_food_meals	6. In the past four weeks, did you or any household member have to eat fewer meals in a<br>day because there was not enough food?	yesno, Required<br>1 Yes<br>0 No

1829	ls_fs_food_meals_y<br>Show the field ONLY if: [ls_fs_food_meals] = '1'	If yes, how often did this happen?	dropdown, Required<br>1 Rarely (once or twice in the past four weeks)<br>2 Sometimes (three to ten times in the past four weeks)<br>3 Often (more than ten times in the past four weeks)
1830	ls_fs_food_none	7. In the past four weeks, was there ever no food to eat of any kind in your household<br>because of lack of resources to get food?	yesno, Required<br>1 Yes<br>0 No
1831	ls_fs_food_none_y<br>Show the field ONLY if: [ls_fs_food_none] = '1'	If yes, how often did this happen?	dropdown, Required<br>1 Rarely (once or twice in the past four weeks)<br>2 Sometimes (three to ten times in the past four weeks)<br>3 Often (more than ten times in the past four weeks)
1832	ls_fs_food_hungry_nt	8. In the past four weeks, did you or any household member go to sleep at night hungry<br>because there was not enough food?	yesno, Required<br>1 Yes<br>0 No
1833	ls_fs_food_hungry_nt_y<br>Show the field ONLY if: [ls_fs_food_hungry_nt] = '1'	If yes, how often did this happen?	dropdown, Required<br>1 Rarely (once or twice in the past four weeks)<br>2 Sometimes (three to ten times in the past four weeks)<br>3 Often (more than ten times in the past four weeks)
1834	ls_fs_food_hungry_day	9. In the past four weeks, did you or any household member go a whole day and night<br>without eating anything because there was not enough food?	yesno, Required<br>1 Yes<br>0 No
1835	ls_fs_food_hungry_day_y<br>Show the field ONLY if: [ls_fs_food_hungry_day] = '1'	If yes, how often did this happen?	dropdown, Required<br>1 Rarely (once or twice in the past four weeks)<br>2 Sometimes (three to ten times in the past four weeks)<br>3 Often (more than ten times in the past four weeks)
1836	food_security_complete	Section Header: *Form Status*<br>Complete?	dropdown<br>0 Incomplete<br>1 Unverified<br>2 Complete
Instrument: **Campy Lab Test** (campy_lab_test)			Collapse
1837	camp_script	Campylobacter genus-specific qPCR - Note: The below information should be entered for all observations undergo a genus-specific qPCR test.	descriptive
1838	camp_samp_date	Sample Date:	text (date_mdy)
1839	camp_lab_id	1. Lab ID<br>*(lab ID is comprised of a three-digit code which represents the household, two letters representing sample category and host, followed by two digits representing timepoint)*	text
1840	camp_category	2. Sample category	radio<br>1 Human<br>2 Livestock<br>3 Environmental

Havelaar AH, *et al. BMJ Open* 2022; 12:e061311. doi: 10.1136/bmjopen-2022-061311

1841	camp_host	3. Sample Host	radio<br>1 Child<br>2 Sibling<br>3 Mother<br>4 Cattle<br>5 Sheep<br>6 Goat<br>7 Chicken<br>8 Drinking water<br>9 Soil
1842	camp_result_avail	4. Is the genus-specific qPCR test result available?	yesno<br>1 Yes<br>0 No
1843	camp_cycle_threshold<br>Show the field ONLY if:<br>[camp_result_avail] = '1'	What is the cycle threshold value (unit)?	text (number)
1844	camp_qpcr_result<br>Show the field ONLY if:<br>[camp_result_avail] = '1'	What is the result for the genus-specific qPCR test?	radio<br>1 Positive<br>0 Negative
1845	camp_lab_threshold<br>Show the field ONLY if:<br>[camp_result_avail] = '1'	What is the Lab threshold used for the positive/negative assignment?	text (number)
1846	camp_qpcr_unavailable<br>Show the field ONLY if:<br>[camp_result_avail] = '0'	Specify a reason that the result of the genus-specific qPCR test is not available:	text
1847	campy_lab_test_complete	Section Header: *Form Status*<br>Complete?	dropdown<br>0 Incomplete<br>1 Unverified<br>2 Complete

Instrument: **Campylobacter Genusspecific Qpcr** (campylobacter_genusspecific_qpcr) Collapse

1848	source	Source	text
1849	dna_extract_code	DNA extraction code	text
1850	code	CAGED sample code	text
1851	type	type of QPCR performed	text
1852	ct	cycle threshold for sample	text
1853	thermophilic_campy	cycle threshold for positive control using thermoplilic DNA	text
1854	nonthermopilic_campy	cycle threshold for positive control non thermoplilic DNA	text
1855	e_coli_1	cycle threshold for negative control E. coli DNA	text
1856	e_coli_2	cycle threshold for negative control E. coli DNA	text
1857	e_coli_3	cycle threshold for negative control E. coli DNA	text
1858	salmonella_1	cycle threshold for negative control Salmonella DNA	text
1859	salmonella_2	cycle threshold for negative control Salmonella DNA	text
1860	nfw_1	cycle threshold for nuclease free water control	text
1861	nfw_2	cycle threshold for nuclease free water control	text
1862	positive_for_campy	Presence or absence of Campylobacter in the sample based on the cut off (Ct< control-1 and 35)	radio<br>1 Presence<br>0 Absence

Havelaar AH, *et al. BMJ Open* 2022; 12:e061311. doi: 10.1136/bmjopen-2022-061311

1863	campylobacter_genusspecific_qpcr_complete	Section Header: *Form Status*<br>Complete?	dropdown<br>0 Incomplete<br>1 Unverified<br>2 Complete

Havelaar AH, *et al. BMJ Open* 2022; 12:e061311. doi: 10.1136/bmjopen-2022-061311
